# Decarboxylative stereoretentive C–N coupling by harnessing aminating reagent

**DOI:** 10.1038/s41467-024-48075-w

**Published:** 2024-05-06

**Authors:** Jeonguk Kweon, Bumsu Park, Dongwook Kim, Sukbok Chang

**Affiliations:** 1 Center for Catalytic Hydrocarbon Functionalizations, Institute for Basic Science (IBS), Daejeon, 34141 South Korea; 2 Department of Chemistry, Korea Advanced Institute of Science and Technology (KAIST), Daejeon, 34141 South Korea

**Keywords:** Synthetic chemistry methodology, Stereochemistry, Reaction mechanisms

## Abstract

In recent decades, strategies involving transition-metal catalyzed carbon-carbon or carbon-heteroatom bond coupling have emerged as potent synthetic tools for constructing intricate molecular architectures. Among these, decarboxylative carbon-nitrogen bond formation using abundant carboxylic acids or their derivatives has garnered notable attention for accessing alkyl- or arylamines, one of key pharmacophores. While several decarboxylative amination methods have been developed, the involvement of a common carboradical intermediate currently poses challenges in achieving stereospecific transformation toward chiral alkylamines. Herein, we present a base-mediated, stereoretentive decarboxylative amidation by harnessing 1,4,2-dioxazol-5-one as a reactive and robust amidating reagent under transition-metal-free ambient conditions, encompassing all types of primary, secondary and tertiary carboxylic acids, thereby providing access to the important pharmacophore, α-chiral amines. This method exhibits high functional group tolerance, convenient scalability, and ease of applicability for ^15^N-isotope labeling, thus accentuating its synthetic utilities. Experimental and computational mechanistic investigations reveal a sequence of elementary steps: i) nucleophilic addition of carboxylate to dioxazolone, ii) rearrangement to form a dicarbonyl N-hydroxy intermediate, iii) conversion to hydroxamate, followed by a Lossen-type rearrangement, and finally, iv) reaction of the in situ generated isocyanate with carboxylate leading to C–N bond formation in a stereoretentive manner.

## Abstract (web summary)

Accessing chiral alkylamines is highly important due to their ubiquity in bioactive molecules. Here, the authors present the development of stereoretentive decarboxylative amidation of carboxylic acids using dioxazolone under ambient conditions.

## Introduction

**Fig. 1:**
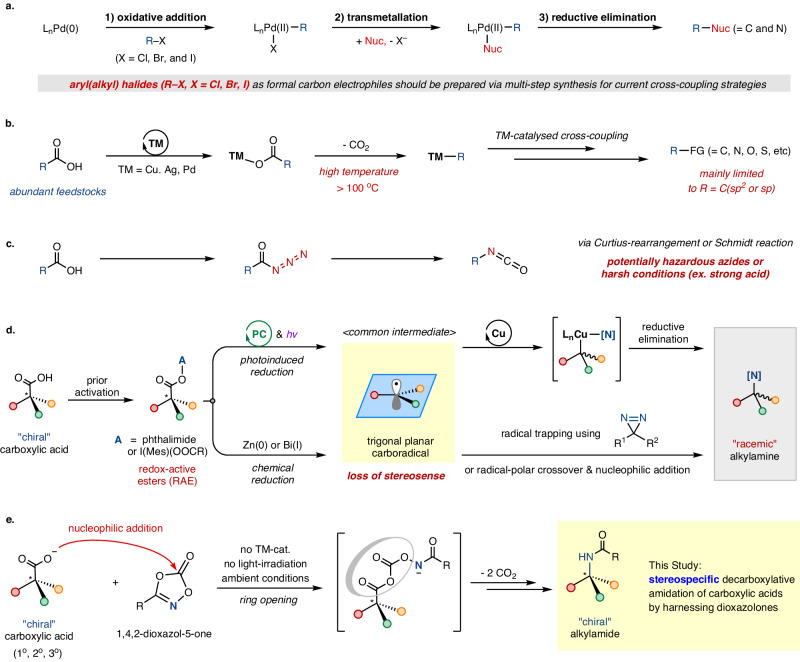
Decarboxylative C–N bond couplings. **a** Transition-metal catalyzed cross-coupling reactions. **b** Transition-metal catalyzed decarboxylative cross-coupling reactions. **c** Conventional acylazide-mediated formal decarboxylative amination. **d** Previous approaches for the decarboxylative amination methods. **e** Current work: transition-metal-free stereospecific decarboxylative amidation of carboxylic acids using 1,4,2-dioxazol-5-one as an amino source.

In the preceding decades, scientific breakthroughs in the field of organic chemistry have paved the way for innovative synthetic methodologies, thereby unlocking the potential to create complex molecular skeletons. One remarkable avenue in this journey has been the development of transition-metal catalyzed cross-coupling reactions, positioning them as a versatile synthetic toolbox for building chemical architectures^1,2^. Noteworthy examples include catalytic carbon-carbon or carbon-nitrogen bond-forming reactions, now applied for both academic research and industrial chemical synthesis (Fig. 1a)^3,4^. Despite the notable advances in these cross-coupling reactions, prefunctionalized aryl(alkyl) halides (or their pseudohalides) are conventionally employed to initiate the required oxidative addition, leading to the following bond-forming steps. In this context, there has been a demand for the development of new coupling protocols to utilize copious and readily obtainable aryl(alkyl) sources.

In response to this desire, a decarboxylative cross-coupling strategy using abundant carboxylic acid feedstocks has arisen as an appealing alternative^5,6^. In fact, transition-metal catalyzed decarboxylative functionalizations were developed, while they were limited mainly to aryl- or alkynylcarboxylic acid substrates under rather harsh reaction conditions (>100 ^o^C), presumably due to the thermodynamically demanding CO_2_ extrusion (Fig. 1b)^5^. To overcome these constraints, a redox-mediated one-electron process for the decarboxylation of carboxylic acids or their derivatives, such as redox-active esters (RAEs), has been actively investigated to achieve milder cross-coupling procedures, especially to enable decarboxylative C(sp^3^)–N bond formation^6,7^, accessing one of essential biorelevant motifs prevalent in natural products, agrochemicals, and pharmaceuticals^8–10^. As note, while the conventional Curtius rearrangement or Schmidt reaction has been utilized for the formal decarboxylative amination, a significant drawback is the requirement of potentially hazardous compounds, such as azides or strong acids (Fig. 1c)^11,12^.

In recent years, harnessing redox-active esters, derived from carboxylic acids, has proven to be effective in facilitating C(sp^3^)–N bond formation under reductive decarboxylative conditions (Fig. 1d)^13–17^. While Fu and Peters pioneered a photocatalyzed intramolecular decarboxylative amidation of N-hydroxyphthalimide (NHP) esters^13^, MacMillan and Hu independently showcased metallaphotoredox catalysis to forge C(sp^3^)–N bonds in an intermolecular fashion^14,15^. Additionally, Lopchuck and Cornella independently reported the facile chemical reduction of RAEs using Zn(0) or Bi(I), inducing decarboxylative carboradical generation for subsequent trapping with a diazirine reagent or coupling with N-nucleophiles via radical-polar crossover, respectively^16,17^. Notably, Yoon unveiled an elegant decarboxylative amination procedure employing carboxylic acids directly without prior activation, wherein photoinduced ligand-to-metal charge transfer (LMCT) was utilized to engender carboradical species^18,19^. Despite these remarkable recent achievements, the involvement of trigonal planar carboradicals as an indispensable intermediate poses an insurmountable challenge in preserving the stereochemical information of starting materials, especially when chiral carboxylic acids are employed.

In our continuous exploration of transition-metal catalyzed nitrenoid group transfer reactions, we have demonstrated a multitude of amidation protocols utilizing 1,4,2-dioxazol-5-ones (dioxazolones), readily accessible from abundant carboxylic acids, as an efficient acylnitrenoid precursor^20–22^. Nevertheless, the use of this robust amidation reagent under metal-free conditions, bypassing the elaborated nitrenoid-involved pathways, has remained rather limited^23^. Drawing inspiration from the electrophilic reactivity of dioxazolones^24^, we hypothesized that nucleophilic addition into this amide precursor may lead to the formation of a ring-opened intermediate, to induce a subsequent rearrangement giving rise to alkyl(aryl)amides in a stereospecific manner (Fig. 1d). This conceptualization was envisaged to furnish a transition-metal-free decarboxylative amidation procedure accessing α-chiral amines when chiral carboxylic acids are used.

Herein, we present a base-mediated decarboxylative amidation of carboxylic acids to provide synthetically versatile N-alkyl(aryl)amides using easily procurable dioxazolones. This approach proved amenable to all types of carboxylic acid substrates (primary, secondary, and tertiary) under mild and ambient transition-metal-free conditions, most significantly enabling a stereoretentive transformation to access medicinally important chiral N-alkylamides. The synthetic applicability of the current reaction was underscored by high functional group tolerance as well as its scalability and ability to afford ^15^N-labeled compounds. Both experimental and computational mechanistic studies unveiled details on the pathway involving nucleophilic addition of carboxylate to dioxazolone, followed by sequential rearrangement and decarboxylation, eventually enabling stereoretentive amidation.

## Results

### Reaction optimization of transition-metal-free decarboxylative amidation

**Fig. 2:**
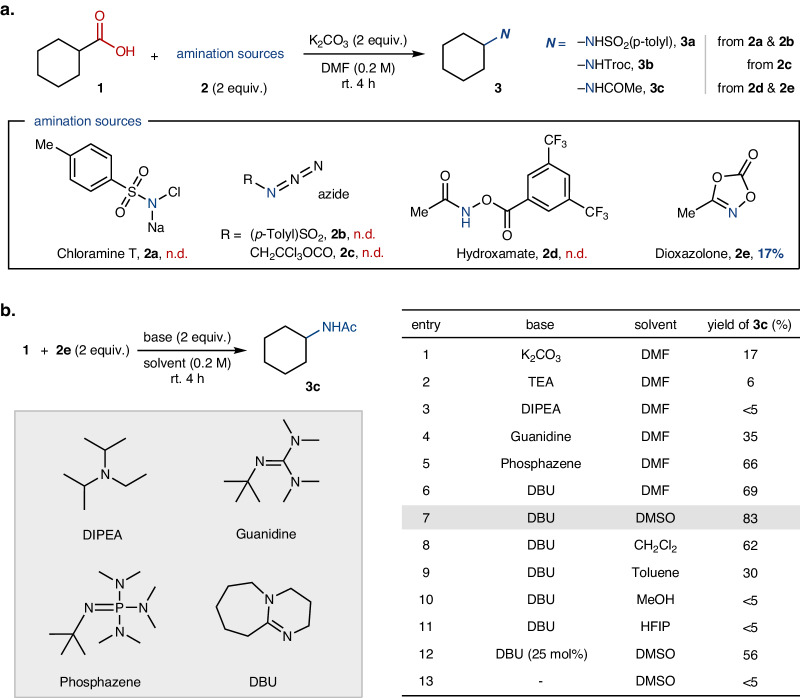
Reaction optimization of transition-metal-free decarboxylative amidation. **a** Reaction tests using a series of amination sources for decarboxylative amidation. **b** Reaction optimization of decarboxylative amidation using dioxazolone. Reaction conditions: cyclohexanecarboxylic acid (**1**, 0.1 mmol), 3-methyl-1,4,2-dioxazol-5-one (**2e**, 2 equiv.) and indicated base (2 equiv.) in indicated solvent (0.2 M) at room temperature for 4 h. The product yield was measured by ^1^H-NMR analysis of the crude reaction mixture in the presence of internal standard (1,3,5-trimethoxybenzene). DMF *N*,*N*-dimethylformamide, DMSO dimethylsulfoxide, HFIP 1,1,1,3,3,3-hexafluoroisopropanol, TEA triethylamine, DIPEA diisopropylethylamine, and DBU 1,8-diazabicyclo[5.4.0]undec-7-ene.

We commenced our study using cyclohexanecarboxylic acid **1** as a model substrate and assessed the reactivity of various aminating reagents (Fig. 2a). As a note, selected N-sources such as chloramine T (**2a**), organic azides RN_3_ (e.g., R = Ts; **2b** and Troc; **2c**), hydroxamates (e.g., **2d**), and 1,4,2-dioxazol-5-ones (e.g., **2e**) have been fruitfully employed in previously established amination reactions^25–30^. The decarboxylative amidation of **1** was not viable with such amino precursors as chloramine **2a**, azides (**2b,** **2c**) and hydroxamate **2d**. In contrast, using dioxazolone **2e**, the corresponding *N*-cyclohexylacetamide **3c** was obtained, albeit in low yield (17%), in the presence of potassium carbonate (K_2_CO_3_) in *N*,*N*-dimethylformamide (DMF) solvent.

Given the initial observation of the promising reactivity of the dioxazolone with carboxylic acid to yield N-alkylamide, we endeavored to optimize conditions by modulating reaction parameters (Fig. 2b, also see the Supplementary Information for details). The use of weak organic bases, including triethylamine (TEA) and diisopropylethylamine (DIPEA), proved ineffective in enhancing the reactivity (entries 2 and 3). However, significant enhancement of reactivity was observed using stronger bases (2 equiv.) such as guanidine, phosphazene, or 1,8-diazabicyclo[5.4.0]undec-7-ene (DBU) as shown in entries 4–6, respectively. Furthermore, switching the solvent to dimethylsulfoxide (DMSO) improved the yield to 83% for the corresponding N-alkylamide **3c** (entry 7). Additional screening revealed that polar aprotic solvents gave rise to moderate to high product yields (entries 6–8), while nonpolar or polar protic solvents resulted significantly reduced productivity (entries 9–11). Notably, a reaction with 25 mol% of DBU led to a moderate yield of **3c** (56%, entry 12), indicative of a potential practical utility of the current amidation reaction under base-catalyzed conditions, while no reactivity observed in the absence of a base additive underscored its critical role (entry 13). Consequently, we identified the optimal amidation conditions to employ 2 equiv. of each dioxazolone and DBU in DMSO (0.2 M) at room temperature for 4 h.

### Substrate scope investigations with achiral carboxylic acids

**Fig. 3:**
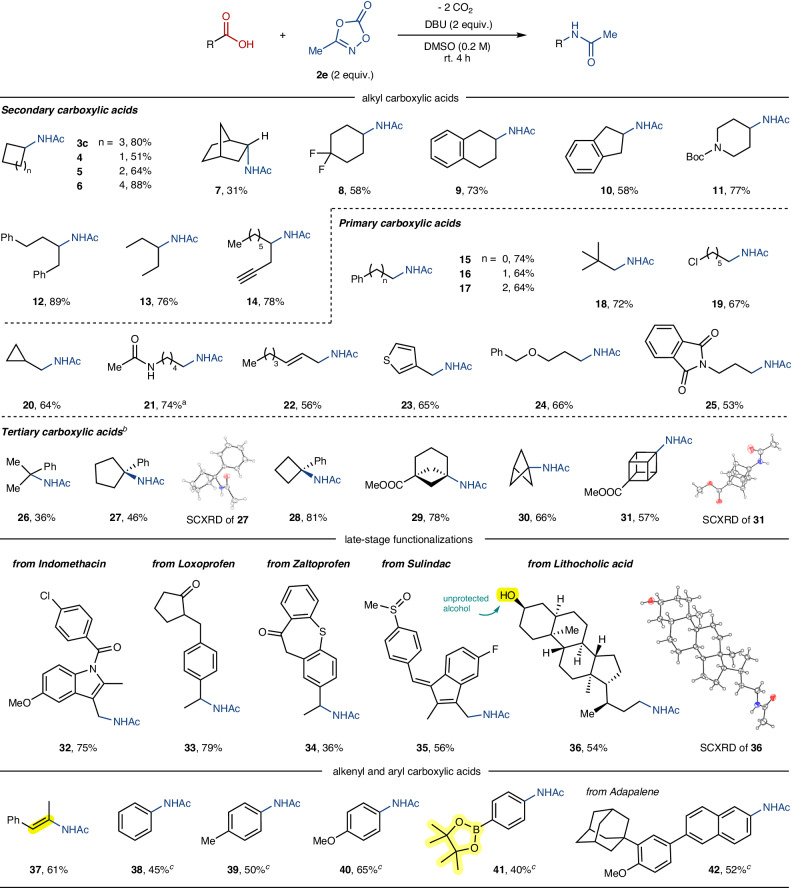
Substrate scope of decarboxylative C–N bond coupling of achiral carboxylic acids. Reaction conditions: carboxylic acid (0.2 mmol), 3-methyl-1,4,2-dioxazol-5-one (**2e**, 2 equiv.) and DBU (2 equiv.) at room temperature for 4 h. Isolated yields are reported. ^a^Run in DMF (0.2 M). ^*b*^Run for 12 h. ^*c*^Run with 3 equiv. of **2e** at 60 ^o^C. SCXRD stands for single crystal X-ray diffraction.

We next explored the scope of achiral carboxylic acids in the present decarboxylative amidation reaction (Fig. 3). Encouragingly, cyclic 2°-carboxylic acids readily converted into the corresponding N-cycloalkylamides under the standard conditions (**3c,** **4**–**11**). Notably, cycloalkanecarboxylic acids with varying ring sizes posed no difficulty for the efficiency of the decarboxylative amidation (**4**–**6**). The sterically hindered bicyclo[2.2.1]heptan-2-yl group was also amenable, albeit in low yield (**7**). An N-Alkylamide incorporating a difluoromethylene moiety was efficiently obtained (**8**). Carboxylic acids featuring benzofused rings were smoothly converted to the desired amide products (**9** and **10**) and a Boc-protected amino group was well tolerated, providing the corresponding alkylamide in good yield (**11**). Additionally, secondary acyclic carboxylic acids proved to be viable under the current amidation conditions (**12**–**14**). It is noteworthy that the terminal alkynyl moiety, one of sensitive functional groups under basic conditions (pK_a_ ~ 26), remained intact in this conversion giving rise to **14** in high yield.

Subsequently, our substrate scope investigation was extended to encompass primary carboxylic acids to obtain terminal amide compounds under otherwise identical reaction conditions (**15**–**25**). It was shown that the alkyl chain length did not significantly affect the current amidation efficiency (**15**–**17**), and an α-*tert*-butyl group was well accommodated (**18**). Notably, the carbon-chloride bond, prone to cleave under radical-involved conditions via halide atom abstraction^31^, presented no impediment and afforded the corresponding product **19** in high yield. The successful conversion of α-cyclopropylacetic acid to *N*-(cyclopropylmethyl)acetamide **20** occurred without ring-opening, implying that the current decarboxylative amidation may follow a two-electron pathway rather than a radical process (*vide infra*)^32^. Intriguingly, 1,5-diacetamide (**21**) was also synthesized in high yield from 6-acetamidohexanoic acid in DMF solvent. Furthermore, synthetically versatile allylamide (**22**) was obtained from the corresponding allylic carboxylic acid^33^. A series of substrates containing heteroatom-functional groups, such as thiophenyl (**23**), benzylic ether (**24**), and N-phthalimide (**25**), were readily amenable to the reaction conditions. Furthermore, the current decarboxylative amidation approach was proven to be applicable for the synthesis of α-tertiary amides (**26**–**31**), which are usually inaccessible through the conventional reductive amination approach^34,35^. Notably, strained skeletons such as bicyclo[3.1.1]heptane (BCH), bicyclo[1.1.1]pentane (BCP), and cubane, extensively explored as aryl bioisosteres in the pharmaceutical community^36,37^, underwent smooth conversion into the desired amidated products (**29**–**31**). As a note, the intact cubane skeleton of **31** was unequivocally confirmed by a single crystal X-ray diffraction (SCXRD) analysis.

We then demonstrated that the current decarboxylative amidation could be applied for the late-stage transformation of biorelevant molecules in reaction with methyl dioxazolone **2e**. A variety of non-steroidal anti-inflammatory drugs (NSAIDs), including Indomethacin (**32**), Loxoprofen (**33**), Zaltoprofen (**34**), and Sulindac (**35**), underwent successful transformations, yielding their respective N-acetamide products. Furthermore, an amidated product **36** was obtained from a carboxylic acid tethered with a steroidal backbone, lithocholic acid, and its structure was confirmed through SCXRD analysis. It is noteworthy that the unprotected alcohol, considered as a potential nucleophile, was tolerated under the current reaction conditions. We also found that α,β-unsaturated carboxylic acid readily reacted to furnish synthetically versatile enamide product **37**^38^. Moreover, the current decarboxylative amidation was shown to be viable for establishing C(aryl)–N bonds from aryl carboxylic acids, albeit at slightly elevated temperature (**38**–**42**). Notably, the successful decarboxylative coupling of a benzoic acid derivative bearing a pinacol borate (**41**) holds potential for the programmable sequential C(aryl)–N couplings, by applying a Chan-Evans-Lam-type amination at the borate moiety of **41**^39^. Decarboxylative amidation of biorelevant Adapalene (**42**) was also amenable.

### Substrate scope investigations with chiral carboxylic acids

**Fig. 4:**
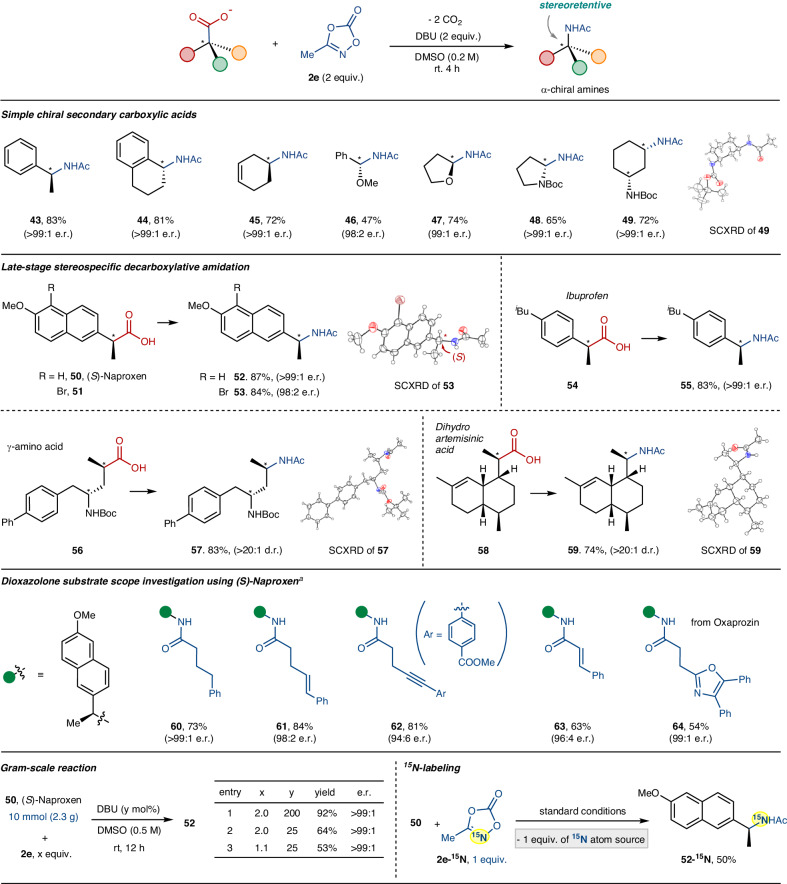
Substrate scope of decarboxylative C(sp^3^)–N bond coupling of chiral carboxylic acids. Reaction conditions: chiral carboxylic acid (0.2 mmol), dioxazolone (2 equiv.) and DBU (2 equiv.) in DMSO (0.2 M) at room temperature for 4 h. Isolated yields are reported. ^*a*^Run in DMF (0.2 M) at 0 ^o^C for 12 h. Enantiomeric ratio (e.r.) was determined by high-performance liquid chromatography (HPLC). Diastereomeric ratio (d.r.) was determined by ^1^H-NMR of the crude reaction mixture.

Optically active α-chiral amines serve as important building blocks in organic synthesis and medicinal chemistry^40,41^. This pharmacophore can be synthetically accessed by enantioselective reductive amination or hydrogenation of ketimine precursors^40,41^. In this context, we wondered whether our current decarboxylative amidation procedure could also be applied to abundant chiral carboxylic acids to obtain optically active α-chiral amines. Given that there is no precedent for stereospecific one-electron redox-mediated decarboxylative amination^13–19^, we anticipated that our envisaged stereo-controlled conversion, if applicable, would open a new avenue to access highly valuable α-chiral amines. Gratifyingly, a wide range of chiral carboxylic acids were smoothly converted into N-alkylamides in a completely stereoretentive manner (Fig. 4, **43**–**49**). This stereospecific transformation proved effective for a broad range of carboxylic acids bearing a chiral center at the benzylic or remote alkyl position, irrespective of cyclic or acyclic molecular skeleton. Notably, chiral hemiaminal (**46,** **47**) and aminal (**48**) products, typically prone to racemize easily under basic conditions due to the acidic heteroatom α-C–H bonds, could be readily obtained from the parent chiral carboxylic acids with excellent stereospecificity. Additionally, chiral diamine **49** bearing two stereogenic centers at the 1,3-position was successfully prepared from the corresponding γ-amino acid derivative (>99:1 e.r.), with the diastereomeric structure being confirmed by SCXRD.

Following this, we explored the synthetic versatility of the current stereospecific decarboxylative amidation procedure by applying it to biorelevant chiral carboxylic acids. (*S*)-Naproxen (**50**) and its brominated derivative (**51**) effectively underwent decarboxylative amidation using methyl dioxazolone **2e**, yielding the corresponding chiral alkylamides (**52** and **53**, respectively). The absolute stereochemistry of **53** was unambiguously confirmed by a single-crystal X-ray diffraction analysis. Ibuprofen (**54**), another class of anti-inflammatory drug, was readily transformed to the desired chiral N-alkylamide with excellent stereospecificity (**55**, >99:1 e.r.). Chiral γ-amino acid (**56**) and dihydroartemisinic acid (**58**), each harboring multiple stereocenters, were also viable, thus producing the desired alkylamides **57** and **59**, respectively, with excellent diastereoselectivity (>20:1 d.r.).

Next, we examined the scope of dioxazolones as the amino source within the framework of the current stereospecific decarboxylative amidation by employing (*S*)-Naproxen **50** as a model carboxylic acid (see the Supplementary Information for additional optimization studies). Notably, considering the fact that dioxazolones are easily prepared in high yields from corresponding carboxylic acids in two steps^21^, the present amidation can be regarded as a formal dual decarboxylative transformation of two carboxylic acid reactants. When slightly modified conditions were applied, being still mild and convenient, a range of N-alkylamides bearing various amide derivatives were readily obtained in a stereoretentive manner with high chemical yields (**60**–**64**). Notably, 3-phenylpropyl dioxazolone, previously known to transform to the 5-membered γ-lactam skeleton via metal-nitrenoid-mediated γ-C–H insertion reported by our group^27^, was successfully incorporated in the current decarboxylative reaction, thereby yielding the corresponding N-alkylamide with >99:1 e.r. (**60**). Additional types of functionalized dioxazolones featuring alkene (**61**) and alkyne (**62**) in their alkyl backbone smoothly underwent the present amidation in a stereoretentive way. As observed, the variation in enantiomeric ratio (**61**–**63**) is likely attributed to the partial involvement of decarboxylative carbanion formation in the benzylic secondary carboxylic acids^42^. Significantly, a dioxazolone derived from α,β-unsaturated carboxylic acid was also viable and provided the desired *N*-cinnamoylamide **63**. Furthermore, a dioxazolone derived from oxazole containing Oxaprozin, one of the NSAIDs, was successfully subjected to the current reaction conditions, providing the corresponding product **64** (99:1 e.r.). It is noteworthy that the classical Curtius rearrangement requires additional subsequent reactions, such as the acylation of alkylamines, to prepare the above amide products tethered with a series of alkyl groups^43^.

Subsequently, we briefly explored the synthetic applicability of the current transition-metal-free dual-decarboxylative amidation method (Fig. 4, bottom). Firstly, a gram scale reaction was examined using (*S*)-Naproxen **50** with dioxazolone **2e**, and similar amidation efficiency was obtained from a 10 mmol scale reaction (2 equiv. of DBU) to afford excellent product yield (92%) and stereospecificity (>99:1 e.r., entry 1). Significantly, the reaction was found to be amenable to some extent with the use of catalytic amounts of DBU and/or in nearly equimolar ratio of two reactants of **50** and **2e** although chemical yields were slightly decreased, but maintaining the stereospecificity (entries 2 and 3). Next, we examined an isotopic ^15^N-incorporation in the amidated product given the applications of isotope-labeled compounds in metabolic mechanistic studies^44,45^ or in vivo imaging techniques^46^. A reaction of **50** with 1 equiv. of ^15^N-labeled methyl dioxazolone (**2e-**^**15**^**N**) readily provided the corresponding ^15^N-alkylamide **52-**^**15**^**N** ( > 99:1 e.r.). It should be noted that the Curtius rearrangement requires 3 equivalents of ^15^N atoms (^15^N_3_^–^) for similar isotope labeling of carboxylic acid substrates.

### Mechanistic Investigations of present transition-metal-free decarboxylative amidation using dioxazolone

**Fig. 5:**
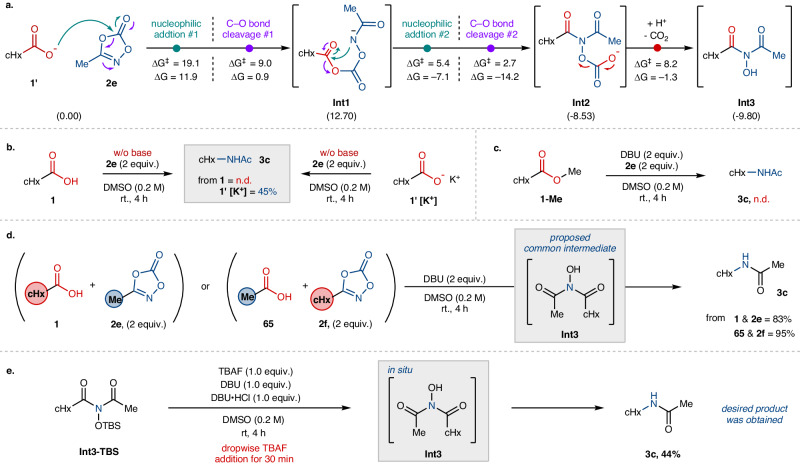
Mechanistic investigations of the transition-metal-free decarboxylative amidation. **a** DFT computed Gibbs energy and activation barrier of nucleophilic addition followed by sequential rearrangement (unit = kcal/mol). M06-2X/6-311 + G**/SMD (solvent = DMSO, ε = 46.826)//M06-2X/6-31 G**. Structure of DBUH^+^ and DBU is omitted for clear presentation. **b** Control experiment using carboxylic acid and its deprotonated form in the absence of base additive. **c** Reaction test using methyl cyclohexylcarboxylate (**1-Me**). **d** Crossover experiment. **e** Test on a symmetric intermediate **Int3**. cHx cyclohexyl, TBAF tetrabutylammonium fluoride, and TBS *tert*-butyldimethylsilyl.

Following the successful development of a transition-metal-free decarboxylative amidation under mild and ambient conditions, we further carried out mechanistic investigations of this stereoretentive transformation (Fig. 5). To elucidate the reaction pathway, density functional theory (DFT) calculations were performed (Fig. 5a, see the Supplementary Information for computational details). Computational studies indicated that nucleophilic attack of cyclohexanecarboxylate **1′** at the carbonyl carbon of dioxazolone **2e** followed by carbonyl substitution via C–O bond cleavage constitutes a kinetically viable pathway {ΔG^‡^ (nucleophilic addition #1) = 19.1 kcal/mol and ΔG^‡^ (C–O bond cleavage #1) = 9.0 kcal/mol}, thus initiating the sequential processes to form a ring-opened intermediate **Int1**. A control experiment, employing cyclohexanecarboxylic acid **1** or its potassium salt **1′[K**^**+**^**]**, showed no reactivity with acid itself in the absence of an external base, while the use of salt gave 45% of the desired product **3c** under the identical conditions, indicative of critical effect of deprotonation in the initial nucleophilic addition (Fig. 5b). Along this line, a reaction with ester **1-Me** instead of carboxylic acid gave no product formation under the standard conditions (Fig. 5c).

Computational studies further revealed that the subsequent intramolecular nucleophilic attack of the postulated amide anion of **Int1** into the tethered carbonyl carbon (Fig. 5a) is highly facile kinetically and thermodynamically (nucleophilic addition #2: ΔG^‡^ = 5.4 kcal/mol, ΔG = –7.1 kcal/mol), thereby inducing the second C–O bond cleavage to afford *N*,*N*-dicarbonyl *N*-carbonate **Int2** (C–O bond cleavage #2: ΔG^‡^ = 2.7 kcal/mol, ΔG = –14.2 kcal/mol). Subsequently, this intermediate **Int2** extrudes CO_2_ to generate a symmetrical dicarbonyl-*N*-hydroxy species **Int3** (ΔG^‡^ = 8.2 kcal/mol, ΔG = –1.3 kcal/mol). To substantiate the existence of the computationally estimated **Int3**, we designed an experiment with two different combinations of carboxylic acids and dioxazolones, namely cyclohexanecarboxylic acid (**1**)/3-methyl 1,4,2-dioxazol-5-one (**2e**) and acetic acid (**65**)/3-cyclohexyl 1,4,2-dioxazol-5-one (**2f**), to see whether a common intermediate **Int3** would be formed eventually to lead to the same product **3c**. Indeed, *N*-cyclohexylacetamide **3c** was obtained exclusively from each reaction with similar efficiency. Furthermore, when *tert*-butyldimethylsilyl (TBS)-protected species **Int3-TBS** was subjected to the reaction conditions with DBU (1 equiv.) and DBU·HCl (1 equiv.), the expected product **3c** was obtained by the presence of tetrabutylammonium fluoride (TBAF), likely via the in situ formation of **Int3** (Fig. 5e). These combined results strongly support our working hypothesis involving **Int3** as a key intermediate during the reaction course.

**Fig. 6:**
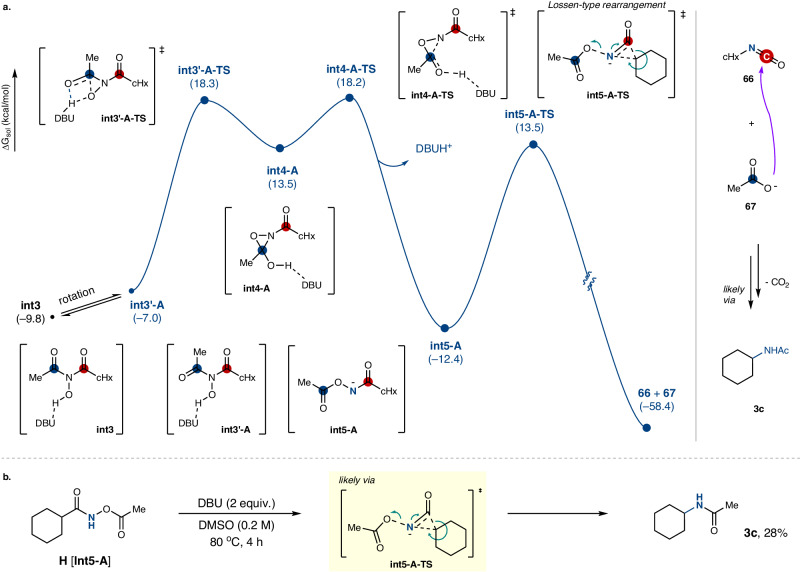
Mechanistic Investigations of the C–N Coupled Product Formation. **a** Computed potential energy surface of plausible rearrangements to access N-alkylamide product (unit = kcal/mol). M06-2X/6-311 + G**/SMD (solvent = DMSO, ε = 46.826)//M06-2X/6-31 G**. **b** Control experiment using a postulated hydroxamate intermediate.

DFT-assisted investigations were further performed to shed light on details underpinning a plausible following rearrangement of the N-hydroxy intermediate (Fig. 6a). The hydroxy group of **Int3′-A** was identified as a kinetically plausible nucleophile to attack at the carbonyl carbon of the tethered acetyl moiety, thereby resulting in the formation of oxaziridine **Int4-A** via **Int3′-A-TS (**ΔG^‡^ = 25.4 kcal/mol). It is noteworthy that an alternative addition of the same hydroxy group of **Int3** to the carbonyl carbon of the cyclohexyl instead of acetyl was found to be disfavored, presumably due to steric hindrance (see the Supplementary Information for more details). Considering that the total activation energy from **Int3** to **Int3′-A-TS** was 28.1 kcal/mol, this step was regarded as the rate-determining step. Upon the formation of **Int4-A**, it was shown that the subsequent ring-opening of oxaziridine to form hydroxamate **Int5-A** is highly facile (ΔG^‡^ = 4.7 kcal/mol, ΔG = –25.9 kcal/mol). In addition, a Lossen-type rearrangement of **Int5-A** to lead cyclohexyl isocyanate **66** along with acetate **67** is also plausible (ΔG^‡^ = 25.9 kcal/mol, ΔG = –46.1 kcal/mol). As the final process, a coupling of isocyanate with carboxylate will take place to release CO_2_ via a Hofmann-type rearrangement to provide alkylamide product, importantly in a stereoretentive manner (Fig. 6a, right)^47–49^. In fact, in line with our proposed pathway, when an additional control experiment was conducted using hydroxamate **H[Int5-A]** under standard conditions, *N*-cyclohexylacetamide **3c** was observed to form albeit at elevated temperature (Fig. 6b). It should be noted that our reaction pathway does not involve any carboradical (or carbocation) intermediates during the course of the whole processes, thus attributing to the observed excellent stereospecificity in the present decarboxylative amidation reaction (Fig. 4).

In summary, we have successfully developed a stereospecific decarboxylative amidation of abundant carboxylic acids by employing 1,4,2-dioxazol-5-ones as a robust amidating reagent under mild and transition-metal-free conditions. This methodology enables a facile access to synthetically versatile N-alkylamides in a stereoretentive manner, preserving the stereochemical information of chiral carboxylic acids to provide α-chiral amines, an important pharmacophore. Through combined experimental and computational studies, we unveiled a mechanistic scaffold operative for the dioxazolone activation, and this pathway is distinctive from the previously established metal-acylnitrenoid-mediated processes. The present amidation reaction is initiated by a nucleophilic addition of carboxylate to the carbonyl of dioxazolones, followed by cascade rearrangements to offer stereospecific dual-decarboxylative pathway.

## Methods

### General procedure for transition-metal-free decarboxylative amination using dioxazolones

To an oven-dried 4 mL screw-capped vial equipped with oval-shaped stirring bar were added carboxylic acids (0.200 mmol), 1,8-diazabicyclo[5.4.0]undec-7-ene (DBU, 2.00 equiv., 0.400 mmol, 60.9 mg), and anhydrous dimethylsulfoxide (DMSO, 1.00 mL, 0.200 M) under atmospheric conditions. To the reaction mixture was added 3-alkyl-1,4,2-dioxazol-5-one (2.00 equiv., 0.400 mmol, 40.4 mg) and stirred for 4 h at room temperature. After reaction completion, the crude reaction mixture was diluted with dichloromethane (DCM, 5.0 mL), added 1 N HCl aqueous solution (10 mL), and extracted with DCM (5 mL × 3 times). The combined organic layer was dried over MgSO_4_, filtered, and concentrated under the reduced pressure. The crude mixture was subjected to silica column chromatography to provide the purified desired N-alkylamide products (eluent: Dichloromethane/Acetone, 100:0–50:50).

### Supplementary information

Supplementary Information

Peer Review File

Description of Additional Supplementary Files

Supplementary Data 1

## Data Availability

All data are available in the main text or the supplementary materials and from the Cambridge Crystallographic Data Center (CCDC; https://www.ccdc.cam.ac.uk/structures/). Crystallographic data are available under CCDC reference numbers 2321815 (**27**), 2321816 (**31**), 2321817 (**36**), 2321818 (**49**), 2321819 (**53**), 2321820 (**57**), and 2321821 (**59**). Computational output files obtained in this study are available in the Zenodo database (10.5281/zenodo.10897788). Cartesian coordinates of computationally optimized geometries are available in Supplementary Data 1. All data are available from the corresponding author upon request.
