# Supplementary material for: Decarboxylative stereoretentive C–N coupling by harnessing aminating reagent
Source: Nat Commun. 2024 May 6;15:3788. doi: 10.1038/s41467-024-48075-w (PMC11074145; doi:10.1038/s41467-024-48075-w)
Supplement: Supplementary file 1 — Supplementary Information [file 41467_2024_48075_MOESM1_ESM.pdf]

Supplementary Information

Decarboxylative Stereoretentive C–N Coupling by Harnessing Aminating Reagent

Jeonguk Kweon,[1,2,‡] Bumsu Park,[2,‡] Dongwook Kim,[2,1] and Sukbok Chang[2,1,*]

[1]Center for Catalytic Hydrocarbon Functionalizations, Institute for Basic Science (IBS), Daejeon 34141, South Korea

[2]Department of Chemistry, Korea Advanced Institute of Science and Technology (KAIST), Daejeon 34141, South Korea

[‡]These authors contributed equally in this work.

*Emails: sbchang@kaist.ac.kr (S.C.)

Table of Contents

I. General Information S2

II. Reaction Tests of Decarboxylative Amination Using a Series of Amination Sources S3

III. Reaction Optimization of Decarboxylative Amidation S5

IV. Control Experiments using Transition-Metal Salts S6

V. Substrate Scope of Decarboxylative Amidation with Achiral Carboxylic Acids S7

VI. Substrate Scope of Decarboxylative Amidation with Aryl Carboxylic Acids S17

VII. Substrate Scope of Decarboxylative Amidation with Chiral Carboxylic Acids S20

VIII. Dioxazolone Scope of Decarboxylative Amidation of (*S*)-Naproxen S33

IX. Synthetic Applicability S40

X. Experimental Mechanistic Investigation S44

XI. Computation Details S51

Spectral Copies of ^{1}H, ^{13}C, and ^{19}F NMR of Compounds Obtained in This Study S56

Crystallographic Data of *27, 31, 36, 49, 53, 57, and 59* S127

Supplementary References S135

Supplementary Methods

I. General Information

Unless otherwise noted, all commercial reagents and solvents were used directly without additional purification. Analytical and preparative thin-layer chromatography (TLC) was performed on Merck pre-coated silica gel 60 F_{254} plates. Visualization on TLC plates was achieved under UV light (254 nm) or treatment with potassium permanganate stain followed by heating. Flash column chromatography was undertaken on silica gel Combi*Flash*® R_f+ system with Redi*Sep*® R_f silica columns (230 – 400 mesh) using an indicated eluent system. Concentration of solution was carried out by using a rotary evaporator and generally followed by removal of residual solvents on a vacuum line held at 0.1–1 torr. ^{1}H NMR spectra was recorded on Agilent Technologies DD2 (600 MHz), Bruker Avance Neo (500 MHz) or AVANCE NEO Nanobay (400 MHz) spectrometers at room temperature. Chemical shifts were quoted in parts per million (ppm) referenced to the residual solvent peak ($CHCl_3$ in $CDCl_3$: 7.26 ppm, $(CD_3)SO(CD_2H)$ in DMSO-d_6: 2.50 ppm). The following abbreviations were used to describe peak splitting patterns when appropriate: s = singlet, d = doublet, t = triplet, q = quartet, dd = doublet of doublet, dq = doublet of quartet, dt = doublet of triplet, td = triplet of doublet, tt = triplet of triplet, m = multiplet. Coupling constants, *J*, were reported in hertz (Hz). ^{13}C NMR was obtained on Bruker AVANCE III HD (100 MHz), AVANCE NEO (125 MHz), or Agilent Technologies DD2 (150 MHz) and was fully decoupled by broad band proton decoupling. Chemical shifts were reported in ppm referenced to the residual solvent peak. ^{19}F NMR was recorded on Agilent Technologies DD2 (564 MHz), Bruker Avance 500 (471 MHz), and AVANCE III HD (376 MHz). Infrared (IR) spectra were obtained on a Bruker Alpha FT-IR Spectrometer equipped with an iD5 ATR accessory, and frequencies are given in wave numbers (cm^{-1}) and only selected absorbance is reported. Melting point was measured with Buchi Melting Point M-565. High resolution mass spectra (HRMS) were obtained from the Korea Basic Science Institute (Daegu) by using electron ionization (EI) or fast atom bombardment (FAB) method. X-ray diffraction data was collected on a Bruker SMART APEX II coated with Paraton-N oil under a stream of N_2 (g) at 120 K. High pressure liquid chromatography (HPLC) analysis was performed with Shimadzu Prominence HPLC system composed of LC20A pump, and SPD-M20A photodiode array detector. Optical rotaions were recorded with Jasco P-2000 Polarimeter equipped with a temperature controller.

II. Reaction Tests of Decarboxylative Amination using a Series of Amination Sources

1. Preparation of amidation sources

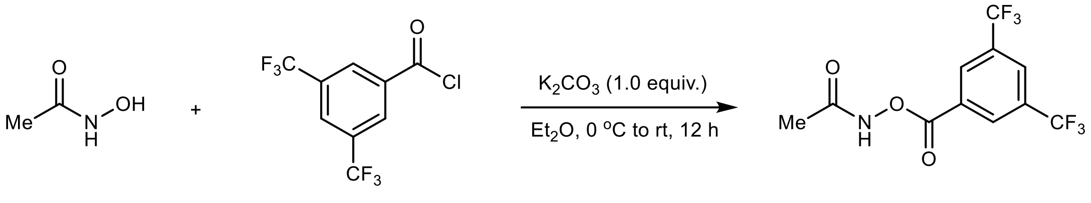

Chloramine T trihydrate was purchased from Sigma Aldrich. Tosyl azide (TsN_3, **2b**), 2,2,2-trichloroethyl carbonazidate ($TrocN_3$, **2c**), and 3-methyl-1,4,2-dioxazole-5-one (**2e**) were prepared following the literature procedure.[1–3] *N*-[{3,5-bis(trifluoromethyl)benzoyl}oxy]acetamide (**2d**) was prepared by modified procedure of previous reports.[4] To an oven-dried 250 mL round bottom flask equipped with oval-shaped stirring bar were added methyl hydroxamic acid (10.0 mmol, 751 mg), potassium carbonate (1.00 equiv., 10.0 mmol, 1.38 g), and diethyl ether (Et_2O, 100 mL, 0.1 M). To a resulting solution was added 3,5-bis(trifluoromethylbenzoyl chloride (1.00 equiv. 10.0 mmol, 2.77 g) in dropwise manner at 0 °C. The reaction mixture was warmed to room temperature and stirred for 12 h. After reaction completion, the mixture was poured into water (100 mL) and extracted with Et_2O (100 mL x 2 times). The combined organic mixture was then dried over $MgSO_4$ and concentrated under the reduced pressure. The crude product was purifed by recrystallization (dichloromethane/n-pentane).

***N*-[{3,5-Bis(trifluoromethyl)benzoyl}oxy]acetamide (2d)**

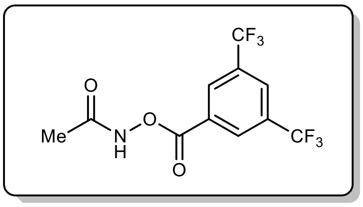

Colorless solid (2.24 g, 71%); **m.p.** = 105 – 107 °C; **^{1}H NMR** (500 MHz, DMSO-d_6) δ 12.19 (s, 1H), 8.56 (s, 1H), 8.53 (s, 2H), 1.98 (s, 3H). **^{13}C NMR** (125 MHz, DMSO-d_6) δ 167.3, 162.0, 131.3 (q, J = 33.9 Hz), 130.1 – 129.3 (m), 127.8, 122.7 (q, J = 273.0 Hz), 19.1; **^{19}F NMR** (471 MHz, DMSO-d_6) δ -61.71; **IR** (cm^{-1}) 3895, 1779, 1660, 1277, 1125, 913, 698, 435; **HRMS** (FAB) m/z calcd. For $C_{11}H_8F_6NO_3$ $[M+H]^+$: 316.0408, found: 316.0413.

2. Reaction tests using a series of amination sources

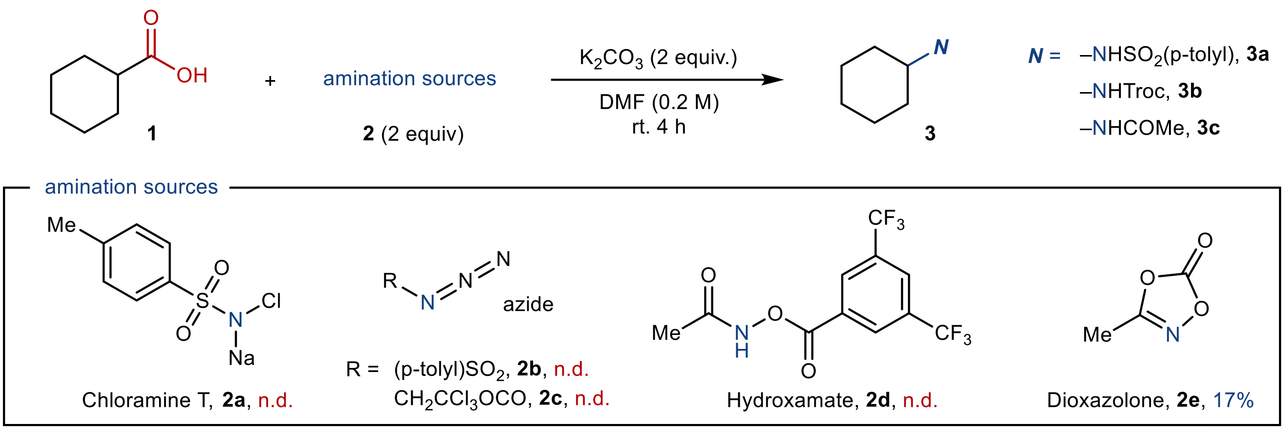

To an oven-dried 4 mL screw-capped vial equipped with oval-shaped stirring bar were added cyclohexanecarboxylic acids (0.100 mmol), potassium carbonate (K_2CO_3, 2.00 equiv., 0.200 mmol, 27.6 mg), and anhydrous *N*,*N*-dimethylformamide (DMF, 0.500 mL, 0.200 M) under atmospheric conditions. To the reaction mixture was added indicated amination sources (2.00 equiv.) and stirred for 4 h at room temperature. Product yield was measured by ^{1}H-NMR analysis of the crude mixture in the presence of internal standard (1,3,5-trimethoxybenzene) in DMSO-d_6. While chloramine **2a**, azides **2b**/**2c**, hydroxamate **2d** were not provided desired amidated product (**3a**, **3b** and **3c**, respectively), dioxazolone **2e** displayed 17% yield of corresponding alkyl amide product **3c**.

III. Reaction optimization of decarboxylative amidation

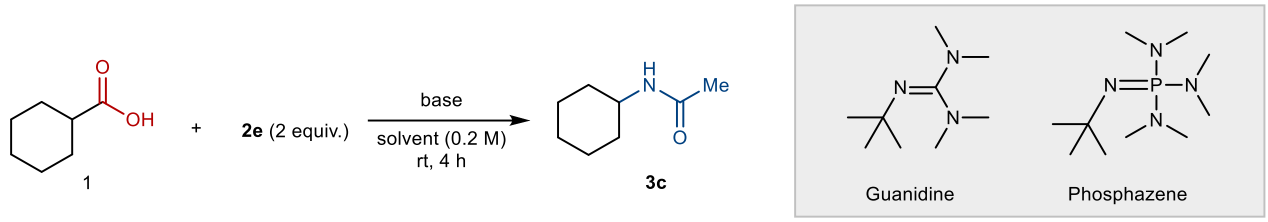

To an oven-dried 4 mL screw-capped vial equipped with oval-shaped stirring bar were added cyclohexyl carboxylic acid (**1**, 0.100 mmol, 12.8 mg), bases (as indicated), and solvent (0.500 mL, 0.200 M) under atmospheric conditions. To the reaction mixture was added dioxazolone **2e** (0.200 mmol, 20.2 mg) and stirred for 4 h at room temperature. Product yield was measured by ^{1}H-NMR analysis of the crude mixture in the presence of internal standard (1,3,5-trimethoxybenzene) in DMSO-d_6.

Supplementary Table 1 | Optimization of the transition metal-free decarboxylative amidation with dioxazolone

entry	base (equiv)	solvent	yield (%)
1	K_2CO_3 (2.0)	DMF	17
2	TEA (2.0)	DMF	6
3	DIPEA (2.0)	DMF	<5
4	Guanidine (2.0)	DMF	35
5	Phosphazene (2.0)	DMF	66
6	DBU (2.0)	DMF	69
7	TEA (2.0)	DMSO	26
8	DIPEA (2.0)	DMSO	19
9	Guanidine (2.0)	DMSO	51
10	Phosphazene (2.0)	DMSO	75
11	DBU (2.0)	DMSO	83
12[a]	DBU (2.0)	DMSO	81
13	DBU (0.25)	DMSO	56
14	-	DMSO	<5
15	DBU (2.0)	DCM	62
16	DBU (2.0)	MeCN	<5
17	DBU (2.0)	Toluene	30
18	DBU (2.0)	MeOH	<5
19	DBU (2.0)	HFIP	<5

[a]Run the experiment under completely dark conditions after wrapping the vial with aluminum foil. TEA = triethylamine, DIPEA = diisopropylethylamine, DBU = 1,8-Diazabicyclo[5.4.0]undec-7-ene, DMF = *N*,*N*-Dimethylformamide, DMSO = Dimethylsulfoxide, DCM = Dichloromethane, HFIP = 1,1,1,3,3,3-Hexafluoroisopropanol.

IV. Control Experiments using Transition-Metal Salts

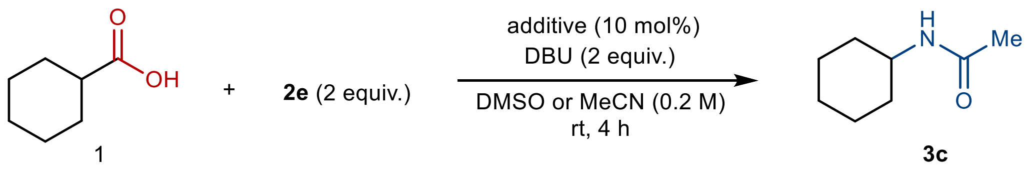

To confirm the no involvement of transition-metal impurities, a series of control experiment using various metal salts were conducted. To an oven-dried 4 mL screw-capped vial equipped with oval-shaped stirring bar were added cyclohexyl carboxylic acid (**1**, 0.100 mmol, 12.8 mg), 1,8-Diazabicyclo[5.4.0]undec-7-ene (DBU, 2.00 equiv, 0.200 mmol, 30.4 mg), indicated additives (10 mol%), and solvent (0.500 mL, 0.200 M) under atmospheric conditions. To the reaction mixture was added dioxazolone **2e** (0.200 mmol, 20.2 mg) and stirred for 4 h at room temperature. Product yield was measured by ^{1}H-NMR analysis of the crude mixture in the presence of internal standard (1,3,5-trimethoxybenzene) in DMSO-d_6. Based on the inhibitory effect of metal salts observed in DMSO solvent and the lack of significantly enhanced reactivity in MeCN, we conclude that the current decarboxylative amidation reactivity with dioxazolone is unlikely to be originated from a small amount of transition metal impurity.

Supplementary Table 2 | Optimization of the transition metal-free decarboxylative amidation with dioxazolone

entry	additive	solvent	yield (%)
1	-	DMSO	83
2	$PdCl_2$		69
3	$FeCl_3$ $6H_2O$		26
4	$CoCl_2$		85
5	$CuCl_2$		64
6	$NiCl_2$ $6H_2O$		57
7	-	MeCN	<5
8	$PdCl_2$		9
9	$FeCl_3$ $6H_2O$		<5
10	$CoCl_2$		24
11	$CuCl_2$		27
12	$NiCl_2$ $6H_2O$		6

V. Substrate Scope of Decarboxylative Amidation with Achiral Carboxylic Acids

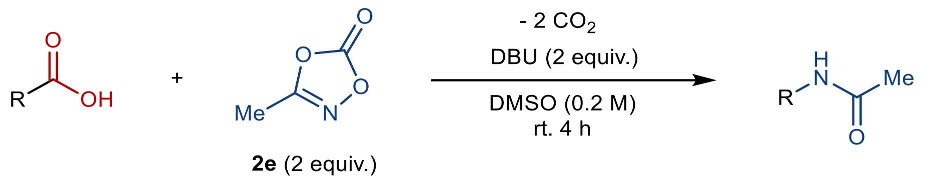

To an oven-dried 4 mL screw-capped vial equipped with oval-shaped stirring bar were added carboxylic acids (0.200 mmol), 1,8-Diazabicyclo[5.4.0]undec-7-ene (DBU, 2.00 equiv., 0.400 mmol, 60.9 mg), and anhydrous dimethylsulfoxide (DMSO, 1.00 mL, 0.200 M) under atmospheric conditions. To the reaction mixture was added 3-methyl-1,4,2-dioxazol-5-one (**2e**, 2.00 equiv., 0.400 mmol, 40.4 mg) and stirred for 4 h at room temperature. After reaction completion, the crude reaction mixture was diluted with dichloromethane (DCM, 5.0 mL), added 1N HCl aqueous solution (10 mL), and extracted with DCM (5 mL x 3 times). The combined organic layer was dried over $MgSO_4$, filtered, and concentrated under the reduced pressure. The crude mixture was subjected to silica column chromatography to provide the purified desired N-alkylamide products (eluent: Dichloromethane/Acetone, 100:0 ~ 50:50).

_N_-Cyclohexylacetamide (3c)

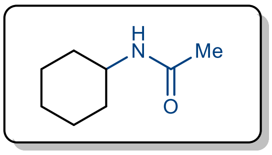

Isolated with DCM/Acetone = 85:15.; Colorless solid (22.6 mg, 80%); **^{1}H NMR** (600 MHz, $CDCl_3$) δ 5.40 (s, 1H), 3.79 – 3.71 (m, 1H), 1.96 (s, 3H), 1.94 – 1.88 (m, 2H), 1.73 – 1.66 (m, 2H), 1.64 – 1.58 (m, 1H), 1.40 – 1.32 (m, 2H), 1.21 – 1.06 (m, 3H); **^{13}C NMR** (150 MHz, $CDCl_3$) δ 169.2, 48.4, 33.4, 25.7, 25.0, 23.8. *Data consistent with those previously reported.*[5]

_N_-Cyclobutylacetamide (4)

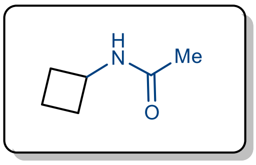

Isolated with DCM/Acetone = 60:40.; Colorless solid (11.6 mg, 51%); **m.p.** = 50 – 52 °C; **^{1}H NMR** (600 MHz, $CDCl_3$) δ 5.63 (s, 1H), 4.44 – 4.34 (m, 1H), 2.39 – 2.29 (m, 2H), 1.94 (s, 3H), 1.88 – 1.79 (m, 2H), 1.74 – 1.66 (m, 2H); **^{13}C NMR** (150 MHz, $CDCl_3$) δ 169.2, 44.9, 31.5, 23.5, 15.2; **IR** (cm^{-1}) 3250, 3062, 2973, 1624, 1544, 1293; **HRMS** (EI) m/z calcd. For $C_6H_{11}NO$ $[M]^+$: 113.0841, found: 113.0842.

N-Cyclopentylacetamide (5)

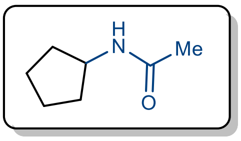

Isolated with DCM/Acetone = 80:20.; Colorless solid (16.2 mg, 64%); **^{1}H NMR** (600 MHz, $CDCl_3$) δ 5.45 (s, 1H), 4.19 (h, J = 7.0 Hz, 1H), 2.02 – 1.96 (m, 2H), 1.95 (s, 3H), 1.66 (tdd, J = 10.3, 8.6, 5.2 Hz, 2H), 1.59 (ddddd, J = 11.3, 9.8, 8.7, 5.6, 4.0 Hz, 2H), 1.39 – 1.31 (m, 2H); **^{13}C NMR** (150 MHz, $CDCl_3$) δ 169.7, 51.4, 33.3, 23.8, 23.6. *Data consistent with those previously reported.*[5]

N-Cycloheptylacetamide (6)

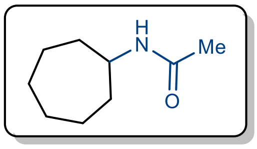

Isolated with DCM/Acetone = 85:15.; Colorless solid (27.4 mg, 88%); **^{1}H NMR** (600 MHz, $CDCl_3$) δ 5.46 (s, 1H), 3.93 (ddq, J = 13.3, 8.9, 4.4 Hz, 1H), 1.93 (s, 3H), 1.90 (dt, J = 7.9, 5.6 Hz, 2H), 1.64 – 1.56 (m, 4H), 1.54 – 1.46 (m, 4H), 1.43 – 1.35 (m, 2H); **^{13}C NMR** (150 MHz, $CDCl_3$) δ 168.9, 50.6, 35.3, 28.2, 24.2, 23.8. *Data consistent with those previously reported.*[5]

N-(Bicyclo[2.2.1]heptan-2-yl)acetamide (7)

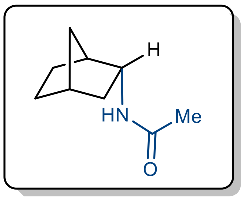

Isolated with DCM/Acetone = 80:20.; Colorless solid (9.4 mg, 31%); **^{1}H NMR** (600 MHz, $CDCl_3$) δ 5.46 (s, 1H), 4.12 (dddd, J = 11.6, 8.8, 5.2, 3.0 Hz, 1H), 2.43 (d, J = 4.1 Hz, 1H), 2.21 (t, J = 4.5 Hz, 1H), 2.09 (dddd, J = 13.0, 11.4, 4.7, 3.2 Hz, 1H), 1.98 (s, 3H), 1.62 – 1.55 (m, 1H), 1.51 – 1.40 (m, 3H), 1.32 (ddt, J = 10.0, 3.3, 1.7 Hz, 1H), 1.21 (dddd, J = 11.5, 8.5, 4.7, 2.3 Hz, 1H), 0.69 (ddd, J = 12.9, 4.7, 3.1 Hz, 1H). **^{13}C NMR** (150 MHz, $CDCl_3$) δ 170.0, 50.9, 40.2, 38.2, 37.8, 36.6, 29.9, 23.5, 21.5. *Data consistent with those previously reported.*[6]

N-(4,4-Difluorocyclohexyl)acetamide (8)

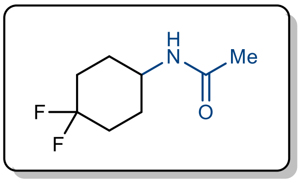

Isolated with DCM/Acetone = 75:25.; Colorless solid (20.6 mg, 58%); **m.p.** = 153 – 155 °C; **^{1}H NMR** (600 MHz, $CDCl_3$) δ 5.35 (s, 1H), 3.94 – 3.84 (m, 1H), 2.14 – 2.04 (m, 2H), 2.03 – 1.95 (m, 5H), 1.85 (dtt, J = 31.3, 13.5, 4.4 Hz, 2H), 1.50 (qd, J = 12.8, 4.0 Hz, 2H); **^{13}C NMR** (150 MHz, $CDCl_3$) δ 169.6, 122.6 (dd, J = 242.8, 239.9 Hz), 46.4, 32.4 (t, J = 25.1 Hz), 28.8 (d, J = 9.6 Hz), 23.6; **^{19}F NMR** (471 MHz, $CDCl_3$) δ -94.78 (d, J = 237.5 Hz), -101.54 (d, J = 237.9 Hz); **IR** (cm^{-1}) 3300, 2944, 1638, 1552, 1123; **HRMS** (EI) m/z calcd. For $C_8H_{13}F_2NO$ $[M]^+$: 177.0965, found: 177.0968.

N-(1,2,3,4-Tetrahydronaphthalen-2-yl)acetamide (9)

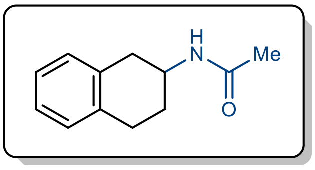

Isolated with DCM/Acetone = 80:20; Colorless solid (27.8 mg, 73%); **^{1}H NMR** (600 MHz, $CDCl_3$) δ 7.16 – 7.08 (m, 3H), 7.08 – 7.04 (m, 1H), 5.55 (s, 1H), 4.34 – 4.26 (m, 1H), 3.12 (dd, J = 16.3, 5.2 Hz, 1H), 2.88 (tdt, J = 17.2, 12.1, 6.6 Hz, 2H), 2.65 (dd, J = 16.3, 7.9 Hz, 1H), 2.09 – 2.01 (m, 1H), 1.98 (d, J = 0.9 Hz, 3H), 1.79 (tdd, J = 10.3, 8.4, 4.3 Hz, 1H); **^{13}C NMR** (150 MHz, $CDCl_3$) δ 169.7, 135.7, 134.2, 130.1, 129.0, 126.8, 126.1, 43.3, 36.8, 29.6, 26.7, 23.7. *Data consistent with those previously reported.*[7]

N-(2,3-Dihydro-*1H*-inden-2-yl)acetamide (10)

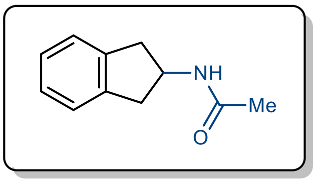

Isolated with DCM/Acetone = 80:20.; Colorless solid (20.3 mg, 58%); **^{1}H NMR** (600 MHz, $CDCl_3$) δ 7.26 – 7.21 (m. 2H), 7.20 – 7.16 (m, 2H), 5.72 (s, 1H), 4.73 (tq, J = 7.4, 4.2, 3.8 Hz, 1H), 3.30 (dd, J = 16.1, 7.0 Hz, 2H), 2.80 (dd, J = 16.1, 4.2 Hz, 2H), 1.94 (s, 3H); **^{13}C NMR** (150 MHz, $CDCl_3$) δ 169.8, 141.0, 126.9, 125.0, 50.8, 40.3, 23.6. *Data consistent with those previously reported.*[8]

***tert*-Butyl 4-acetamidopiperidine-1-carboxylate (11)**

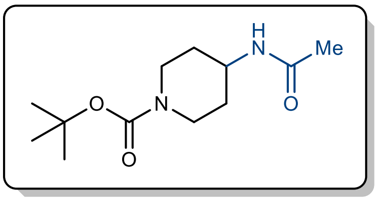

Isolated with DCM/Acetone = 60:40.; Colorless solid (37.1 mg, 77%); **^{1}H NMR** (600 MHz, $CDCl_3$) δ 5.48 (d, J = 8.0 Hz, 1H), 4.18 – 3.94 (m, 2H), 3.90 (tdt, J = 11.6, 8.2, 4.1 Hz, 1H), 2.90 – 2.73 (m, 2H), 1.96 (s, 3H), 1.92 – 1.86 (m, 2H), 1.44 (s, 9H), 1.32 – 1.21 (m, 2H); **^{13}C NMR** (150 MHz, $CDCl_3$) δ 169.5, 154.8, 79.8, 46.9, 42.9, 32.2, 28.6, 23.6. *Data consistent with those previously reported.*[9]

N-(1,4-Diphenylbutan-2-yl)acetamide (12)

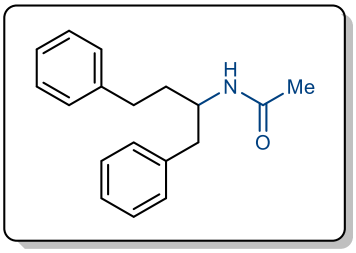

Isolated with DCM/Acetone = 90:10.; Colorless solid (47.8 mg, 89%); **^{1}H NMR** (600 MHz, $CDCl_3$) δ 7.31 – 7.25 (m, 4H), 7.24 – 7.21 (m, 1H), 7.20 – 7.13 (m, 5H), 5.16 (d, J = 8.4 Hz, 1H), 4.27 (dddd, J = 15.3, 9.0, 6.3, 4.6 Hz, 1H), 2.87 – 2.79 (m, 2H), 2.66 (ddd, J = 9.4, 6.6, 3.0 Hz, 2H), 1.91 (s, 3H), 1.89 – 1.82 (m, 1H), 1.70 – 1.62 (m, 1H); **^{13}C NMR** (150 MHz, $CDCl_3$) δ 169.7, 141.8, 137.9, 129.6, 128.5(9), 128.5(7), 128.4(5), 126.7, 126.1, 50.2, 41.0, 35.8, 32.7, 23.6; **IR** (cm^{-1}) 3318, 3022, 2939, 1630, 1531; **HRMS** (EI) m/z calcd. For $C_{18}H_{21}NO$ $[M]^+$: 267.1623, found: 267.1620.

N-(Pentan-3-yl)acetamide (13)

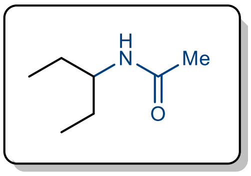

Isolated with DCM/Acetone = 80:20.; Colorless solid (19.7 mg, 76%); **^{1}H NMR** (600 MHz, $CDCl_3$) δ 5.23 (s, 1H), 3.77 (dddd, *J* = 13.1, 9.1, 7.8, 5.4 Hz, 1H), 1.98 (s, 3H), 1.53 (dddd, *J* = 14.9, 12.9, 7.4, 5.3 Hz, 2H), 1.35 (dt, *J* = 13.8, 7.5 Hz, 2H), 0.88 (t, *J* = 7.5 Hz, 6H); **^{13}C NMR** (150 MHz, $CDCl_3$) δ 169.9, 52.1, 27.5, 23.7, 10.3.

Data consistent with those previously reported.[10]

N-(Hex-5-yn-3-yl)acetamide (14)

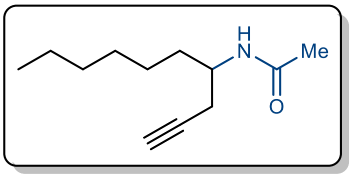

Isolated with DCM/Acetone = 85:15.; Colorless solid (30.3 mg, 78%); **^{1}H NMR** (600 MHz, $CDCl_3$) δ 5.52 (d, *J* = 9.0 Hz, 1H), 4.04 (ddddd, *J* = 9.1, 8.2, 6.4, 5.2, 4.1 Hz, 1H), 2.49 (ddd, *J* = 16.9, 5.2, 2.7 Hz, 1H), 2.36 (ddd, *J* = 16.8, 4.1, 2.6 Hz, 1H), 2.01 – 1.97 (m, 4H), 1.63 – 1.48 (m, 2H), 1.35 – 1.23 (m, 8H), 0.90 – 0.85 (m, 3H); **^{13}C NMR** (150 MHz, $CDCl_3$) δ 169.7, 80.5, 70.9, 47.2, 33.5, 31.8, 29.2, 26.1, 24.2, 23.6, 22.7, 14.2; **IR** (cm^{-1}) 3287, 3263, 2950, 2921, 2852, 1644, 1555; **HRMS** (EI) m/z calcd. For $C_{12}H_{21}NO$ $[M]^+$: 195.1623, found: 195.1622.

N-Benzylacetamide (15)

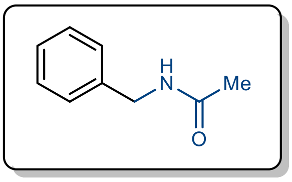

Isolated with DCM/Acetone = 80:20.; Colorless solid (22.1 mg, 74%); **^{1}H NMR** (600 MHz, $CDCl_3$) δ 7.35 – 7.31 (m, 2H), 7.30 – 7.25 (m, 3H), 5.84 (s, 1H), 4.42 (d, *J* = 5.7 Hz, 2H), 2.01 (s, 3H); **^{13}C NMR** (150 MHz, $CDCl_3$) δ 170.0, 138.4, 128.9, 128.0, 127.7, 43.9, 23.4.

Data consistent with those previously reported.[5]

N-Phenethylacetamide (16)

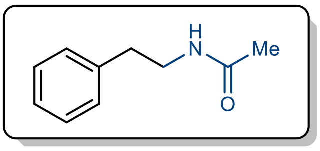

Isolated with DCM/Acetone = 80:20.; Colorless solid (20.9 mg, 64%); **^{1}H NMR** (600 MHz, $CDCl_3$) δ 7.33 – 7.30 (m, 2H), 7.25 – 7.22 (m, 1H), 7.21 – 7.18 (m, 2H), 5.48 (s, 1H), 3.52 (q, *J* = 6.8 Hz, 2H) 2.82 (t, *J* = 7.0 Hz, 2H), 1.94 (s, 3H); **^{13}C NMR** (150 MHz, $CDCl_3$) δ 170.2, 139.0, 128.9, 128.8, 126.7, 40.8, 35.8, 23.5.

Data consistent with those previously reported.[8]

N-(3-Phenylpropyl)acetamide (17)

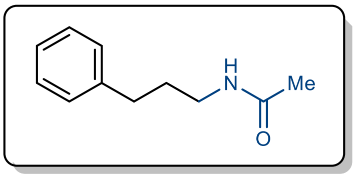

Isolated with DCM/Acetone = 80:20.; Colorless liquid (22.8 mg, 64%); **^{1}H NMR** (600 MHz, $CDCl_3$) δ 7.31 – 7.25 (m, 2H), 7.21 – 7.16 (m, 3H), 5.45 (s, 1H), 3.31 – 3.26 (m, 2H), 2.65 (t, J = 7.7 Hz, 2H), 1.93 (s, 3H), 1.84 (p, J = 7.3 Hz, 2H); **^{13}C NMR** (150 MHz, $CDCl_3$) δ 170.2, 141.6, 128.6, 128.5, 126.2, 39.5, 33.5, 31.3, 23.5.

Data consistent with those previously reported.[11]

N-Neopentylacetamide (18)

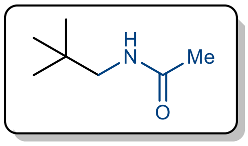

Isolated with DCM/Acetone = 75:25.; Colorless solid (18.5 mg, 72%); **m.p.** = 63 – 65 °C; **^{1}H NMR** (600 MHz, $CDCl_3$) δ 5.51 (s, 1H), 3.06 (d, J = 6.3 Hz, 2H), 2.00 (s, 3H), 0.90 (s, 9H); **^{13}C NMR** (150 MHz, $CDCl_3$) δ 170.2, 50.8, 31.9, 27.3, 23.6; **IR** (cm^{-1}) 3261, 3086, 2957, 2864, 1636, 1583; **HRMS** (EI) m/z calcd. For $C_7H_{15}NO$ $[M]^+$: 129.1154, found: 129.1150.

N-(5-Chloropentyl)acetamide (19)

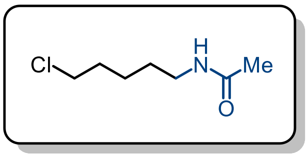

Isolated with DCM/Acetone = 70:30.; Colorless liquid (22.0 mg, 67%); **^{1}H NMR** (600 MHz, $CDCl_3$) δ 5.49 (s, 1H), 3.54 (t, J = 6.6 Hz, 2H), 3.28 – 3.23 (m, 2H), 1.97 (s, 3H), 1.82 – 1.76 (m, 2H), 1.57 – 1.50 (m, 2H), 1.50 – 1.44 (m, 2H); **^{13}C NMR** (150 MHz, $CDCl_3$) δ 170.2, 45.0, 39.5, 32.2, 29.1, 24.3, 23.5; **IR** (cm^{-1}) 3282, 3086, 2933, 2863, 1647, 1553; **HRMS** (EI) m/z calcd. For $C_7H_{14}ClNO$ $[M]^+$: 163.0764, found: 163.0763.

N-(Cyclopropylmethyl)acetamide (20)

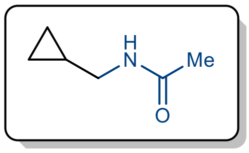

Isolated with DCM/Acetone = 75:25.; Colorless liquid (36.2 mg, 64%); **^{1}H NMR** (600 MHz, $CDCl_3$) δ 5.66 (s, 1H), 3.09 (dd, J = 7.2, 5.4 Hz, 2H), 1.98 (s, 3H), 0.97 – 0.90 (m, 1H), 0.51 – 0.47 (m, 2H), 0.20 – 0.17 (m, 2H); **^{13}C NMR** (150 MHz, $CDCl_3$) δ 170.1, 44.6, 23.5, 10.8, 3.5.

Data consistent with those previously reported.[12]

N,N'-(Pentane-1,5-diyl)diacetamide (21)

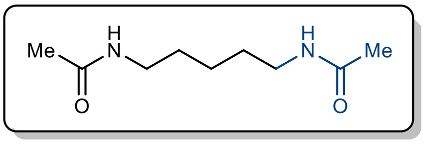

Reaction was conducted in DMF solvent. After reaction completion, the crude mixture was directly subjected to the silica coloum chromatography (eluent: DCM:MeOH, 90:10).; Colorless solid (27.7 mg, 74%); **^{1}H NMR** (600 MHz, $CDCl_3$) δ 5.73 (s, 2H), 3.24 (td, J = 6.9, 5.8 Hz, 4H), 1.98 (s, 6H), 1.52 (p, J = 7.1 Hz, 4H), 1.38 – 1.31 (m, 2H); **^{13}C NMR** (150 MHz, $CDCl_3$) δ 170.5, 39.3, 29.1, 23.8, 23.4. *Data consistent with those previously reported.*[13]

(E)-N-(Hept-2-en-1-yl)acetamide (22)

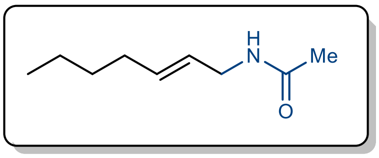

Isolated with DCM/Acetone = 80:20.; Colorless liquid (17.3 mg, 56%); **^{1}H NMR** (600 MHz, $CDCl_3$) δ 5.61 (dtt, J = 14.9, 6.7, 1.4 Hz, 1H), 5.53 – 5.36 (m, 2H), 3.82 – 3.78 (m, 2H), 2.03 – 1.99 (m, 2H), 1.98 (s, 3H), 1.37 – 1.26 (m, 4H), 0.89 (t, J = 7.1 Hz, 3H); **^{13}C NMR** (150 MHz, $CDCl_3$) δ 169.9, 134.2, 125.6, 41.8, 32.0, 31.4, 23.5, 22.3, 14.0; **IR** (cm^{-1}) 3280, 3077, 2956, 2924, 2856, 1648, 1546; **HRMS** (EI) m/z calcd. For $C_9H_{17}NO$ $[M]^+$: 155.1310, found: 155.1309.

N-(Thiophen-3-ylmethyl)acetamide (23)

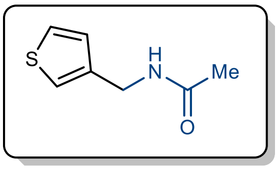

Isolated with DCM/Acetone = 80:20.; Colorless solid (20.2 mg, 65%); **^{1}H NMR** (600 MHz, $CDCl_3$) δ 7.30 (dd, J = 5.0, 3.0 Hz, 1H), 7.17 – 7.13 (m, 1H), 7.03 (dd, J = 5.0, 1.3 Hz, 1H), 5.72 (s, 1H), 4.44 (d, J = 5.6 Hz, 2H), 2.01 (s, 3H); **^{13}C NMR** (150 MHz, $CDCl_3$) δ 169.9, 139.1, 127.5, 126.6, 122.5, 39.0, 23.4. *Data consistent with those previously reported.*[14]

N-(3-(Benzyloxy)propyl)acetamide (24)

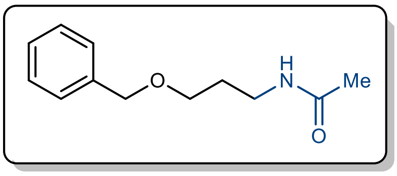

Isolated with DCM/Acetone = 65:35.; Colorless liquid (27.4 mg, 66%); **^{1}H NMR** (600 MHz, $CDCl_3$) δ 7.38 – 7.28 (m, 5H), 6.01 (s, 1H), 4.50 (s, 2H), 3.59 (t, J = 5.7 Hz, 2H), 3.37 (q, J = 6.0 Hz, 2H), 1.88 (s, 3H), 1.83 – 1.78 (m, 2H); **^{13}C NMR** (150 MHz, $CDCl_3$) δ 170.1, 138.3, 128.6, 127.9, 127.8, 73.3, 69.5, 38.4, 29.1, 23.4; **IR** (cm^{-1}) 3286, 3086, 2928, 2859, 1646, 1549, 1098; **HRMS** (FAB) m/z calcd. For $C_{12}H_{18}NO_2$ $[M+H]^+$: 208.1338, found: 208.1335.

N-{3-(1,3-Dioxoisoindolin-2-yl)propyl}acetamide (25)

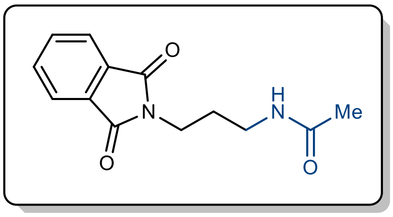

Isolated with DCM/Acetone = 80:20.; Colorless solid (25.9 mg, 53%); **m.p.** = 130 – 132 °C; **^{1}H NMR** (600 MHz, $CDCl_3$) δ 7.85 (dd, *J* = 5.4, 3.1 Hz, 2H), 7.73 (dd, *J* = 5.5, 3.0 Hz, 2H), 6.20 (s, 1H), 3.76 (t, *J* = 6.4 Hz, 2H), 3.23 (q, *J* = 6.3 Hz, 2H), 2.02 (s, 3H), 1.89 – 1.83 (m, 2H); **^{13}C NMR** (150 MHz, $CDCl_3$) δ 170.3, 168.9, 134.3, 132.1, 123.5, 36.2, 35.0, 28.4, 23.6; **IR** (cm^{-1}) 3313, 1767, 1694, 1634, 1552; **HRMS** (EI) m/z calcd. For $C_{13}H_{14}N_2O_3$ $[M]^+$: 246.1004, found: 246.1006.

N-(2-Phenylpropan-2-yl)acetamide (26)

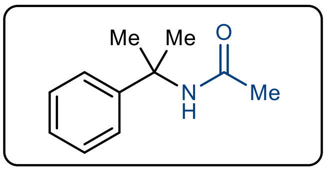

Reaction was conducted for 12 h, Isolated with DCM/Acetone = 80:20.; Colorless solid (12.8 mg, 36%); **^{1}H NMR** (500 MHz, $CDCl_3$) δ 7.42 – 7.35 (m, 2H), 7.37 – 7.29 (m, 2H), 7.25 – 7.20 (m, 1H), 5.74 (s, 1H), 1.97 (s, 3H), 1.70 (s, 6H); **^{13}C NMR** (125 MHz, $CDCl_3$) δ 169.2, 147.0, 128.5, 126.8, 124.9, 56.1, 29.2, 24.5. *Data consistent with those previously reported.*[5]

N-(1-Phenylcyclopentyl)acetamide (27)

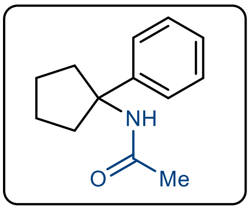

Reaction was conducted for 12 h. Isolated with DCM/Acetone = 90:10.; Colorless solid (18.7 mg, 46%); **m.p.** = 109 – 111 °C; **^{1}H NMR** (500 MHz, $CDCl_3$) δ 7.43 – 7.39 (m, 2H), 7.32 (t, *J* = 7.8 Hz, 2H), 7.24 – 7.19 (m, 1H), 5.86 (s, 1H), 2.42 – 2.33 (m, 2H), 2.14 – 2.03 (m, 2H), 1.96 (s, 3H), 1.88 – 1.77 (m, 4H). **^{13}C NMR** (125 MHz, $CDCl_3$) 169.5, 145.0, 128.1, 126.5, 125.7, 66.7, 39.2, 24.1, 23.2; **IR** (cm^{-1}) 3312, 2964, 1735, 1646, 1539, 1492, 1294, 757, 696, 593, 529; **HRMS** (EI) m/z calcd. For $C_{13}H_{17}NO$ $[M]^+$: 203.1310, found: 203.1308.

*The structure of the title compound was further confirmed by X-ray crystallographic analysis (see **Appendix II**)*

N-(1-Phenylcyclobutyl)acetamide (28)

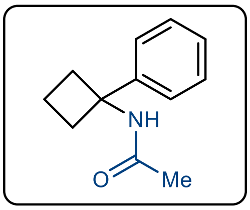

Reaction was conducted for 12 h. Isolated with DCM/Acetone = 85:15.; Colorless liquid (30.8 mg, 81%); **m.p.** = 161 – 163 °C; **^{1}H NMR** (500 MHz, $CDCl_3$) δ 7.47 – 7.41 (m, 2H), 7.37 – 7.29 (m, 2H), 7.25 – 7.18 (m, 1H), 6.09 (s, 1H), 2.64 – 2.55 (m, 4H), 2.15 – 2.01 (m, 1H), 1.92 (s. 3H), 1.90 – 1.79 (m, 1H); **^{13}C NMR** (125 MHz, $CDCl_3$) δ 169.2, 145.5, 128.3, 126.8, 125.7, 59.9, 34.2, 24.0, 15.6; **IR** (cm^{-1}) 3278, 2945, 1642, 1539, 1294, 1032, 754, 698, 599, 536; **HRMS** (EI) m/z calcd. For $C_{12}H_{15}NO$ $[M]^+$: 189.1154, found: 189.1152.

Methyl (1*r*,5*r*)-5-acetamidobicyclo[3.1.1]heptane-1-carboxylate (29)

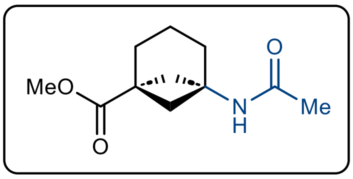

Reaction was conducted for 12 h. Isolated with DCM/Acetone = 85:15; Colorless solid (33.1 mg, 78%); **m.p.** = 114 – 116 °C; **^{1}H NMR** (500 MHz, $CDCl_3$) δ 5.78 (s, 1H), 3.65 (s, 3H), 2.39 (dt, J = 7.3, 3.6 Hz, 2H), 2.04 – 1.99 (m, 2H), 1.99 – 1.94 (m, 2H), 1.92 – 1.82 (m, 7H); **^{13}C NMR** (125 MHz, $CDCl_3$) δ 175.4, 169.5, 52.5, 51.9, 42.4, 41.1, 33.1, 29.2, 23.9, 16.7; **IR** (cm^{-1}) 3274, 3081, 2919, 2862, 1727, 1555, 1436, 1290, 1166, 1065, 731, 606, 526; **HRMS** (EI) m/z calcd. For $C_{11}H_{17}NO_3$ $[M]^+$: 211.1208, found: 211.1207.

***N*-(Bicyclo[1.1.1]pentan-1-yl)acetamide (30)**

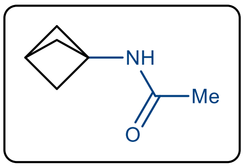

Reaction was conducted for 12 h. Isolated with DCM/Acetone = 90:10.; Colorless solid (15.8 mg, 66%); **m.p.** = 103 – 105 °C; **^{1}H NMR** (500 MHz, $CDCl_3$) δ 5.95 (s, 1H), 2.42 (s, 1H), 2.07 (s, 6H), 1.91 (s, 3H); **^{13}C NMR** (125 MHz, $CDCl_3$) δ 170.5, 52.8, 48.9, 24.9, 23.7; **IR** (cm^{-1}) 3242, 2994, 2913, 2876, 1636, 1548, 1373, 1297, 1194, 1022, 796, 749; **HRMS** (FAB) m/z calcd. For $C_7H_{12}NO$ $[M+H]^+$: 126.0919, found: 126.0917.

Methyl (2*r*,3*R*,4*s*,5*S*)-4-acetamidocubane-1-carboxylate (31)

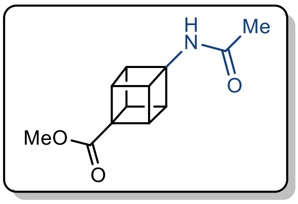

Reaction was conducted for 12 h. Isolated with DCM/Acetone = 90:10.; Colorless solid (25.1 mg, 57%); **m.p.** = 148 – 150 °C; **^{1}H NMR** (500 MHz, $CDCl_3$) δ 6.29 (s, 1H), 4.14 (tdd, J = 8.2, 3.8, 1.9 Hz, 6H), 3.71 (s, 3H), 2.01 (s, 3H); **^{13}C NMR** (125 MHz, $CDCl_3$) δ 172.7, 169.6, 66.6, 55.8, 51.6, 50.2, 45.0, 23.1; **IR** (cm^{-1}) 3247, 2992, 1715, 1634, 1525, 1313, 1214, 1092, 842, 603, 442; **HRMS** (FAB) m/z calcd. For $C_9H_{18}NO$ $[M+H]^+$: 220.0974, found: 220.0973.

*The structure of the title compound was further confirmed by X-ray crystallographic analysis (see **Appendix II**)*

***N*-[{1-(4-Chlorobenzoyl)-5-methoxy-2-methyl-*1H*-indol-3-yl}methyl]acetamide (32)**

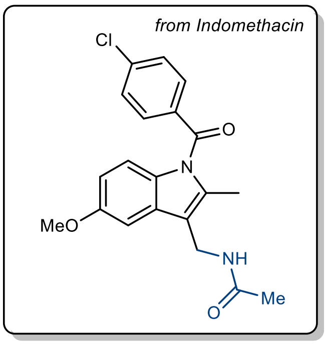

Isolated with DCM/Acetone = 80:20.; Colorless solid (55.3 mg, 75%); **m.p.** = 155 – 157 °C; **^{1}H NMR** (600 MHz, $CDCl_3$) δ 7.67 – 7.63 (m, 2H), 7.49 – 7.45 (m, 2H), 7.00 (d, J = 2.6 Hz, 1H), 6.82 (d, J = 9.0 Hz, 1H), 6.67 (dd, J = 9.0, 2.5 Hz, 1H), 5.60 (s, 1H), 4.53 (d, J = 5.1 Hz, 2H), 3.82 (s, 3H), 2.41 (s, 3H), 2.00 (s, 3H); **^{13}C NMR** (150 MHz, $CDCl_3$) δ 170.1, 168.5, 156.3, 139.7, 136.4, 133.8, 131.3, 131.0, 130.1, 129.3, 115.9, 115.2, 112.2, 101.2, 55.9, 33.7, 23.4, 13.2; **IR** (cm^{-1}) 3298, 1679, 1606, 1540, 1356, 1321; **HRMS** (EI) m/z calcd. For $C_{20}H_{19}ClN_2O_3$ $[M]^+$: 370.1084, found: 370.1086.

***N*-(1-[4-{(2-Oxocyclopentyl)methyl}phenyl]ethyl)acetamide (33)**

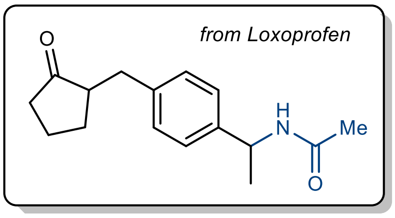

Isolated with DCM/Acetone = 80:20.; Colorless liquid (41.2 mg, 79%); **^{1}H NMR** (600 MHz, $CDCl_3$) δ 7.24 – 7.21 (m, 2H), 7.15 – 7.11 (m, 2H), 5.72 (d, *J* = 8.1 Hz, 1H), 5.10 (p, *J* = 7.2 Hz, 1H), 3.11 (dd, *J* = 14.0, 4.2 Hz, 1H), 2.52 (dd, *J* = 14.0, 9.4 Hz, 1H), 2.37 – 2.29 (m, 2H), 2.14 – 2.05 (m, 2H), 2.01 – 1.92 (m, 4H), 1.78 – 1.68 (m, 1H), 1.58 – 1.51 (m, 1H), 1.47 (d, *J* = 6.9 Hz, 3H); **^{13}C NMR** (150 MHz, $CDCl_3$) δ 220.2, 169.1, 141.2, 139.3, 129.3, 126.4, 51.1, 48.6, 38.3, 35.3, 29.3, 23.6, 21.8, 20.7; **IR** (cm^{-1}) 3284, 2967, 1734, 1643, 1539; **HRMS** (EI) m/z calcd. For $C_{16}H_{21}NO_2$ $[M]^+$: 259.1572, found: 259.1574.

***N*-{1-(10-Oxo-10,11-dihydrodibenzo[b,f]thiepin-2-yl)ethyl}acetamide (34)**

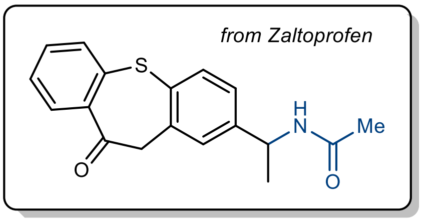

Isolated with DCM/Acetone = 80:20.; Colorless solid (22.6 mg, 36%); **m.p.** = 179 – 181 °C; **^{1}H NMR** (600 MHz, $CDCl_3$) δ 8.19 (dd, *J* = 8.0, 1.6 Hz, 1H), 7.60 (dd, *J* = 7.9, 1.2 Hz, 2H), 7.43 (ddd, *J* = 7.8, 7.2, 1.6 Hz, 1H), 7.38 (d, *J* = 2.0 Hz, 1H), 7.31 (ddd, *J* = 8.3, 7.3, 1.2 Hz, 1H), 7.15 (dd, *J* = 8.0, 2.0 Hz, 1H), 5.68 (d, *J* = 7.8 Hz, 1H), 5.10 (p, *J* = 7.1 Hz, 1H), 4.40 – 4.32 (m, 2H), 1.99 (s, 3H), 1.45 (d, *J* = 7.0 Hz, 3H); **^{13}C NMR** (150 MHz, $CDCl_3$) δ 191.6, 169.3, 145.7, 140.4, 138.1, 136.3, 133.5, 132.7, 131.7, 131.7, 131.0, 127.0, 126.9, 125.5, 51.3, 48.6, 23.6, 22.0; **IR** (cm^{-1}) 3246, 1670, 1632, 1548, 1284; **HRMS** (EI) m/z calcd. For $C_{18}H_{17}NO_2S$ $[M]^+$: 311.0980, found: 311.0976.

(*Z*)-*N*-([5-Fluoro-2-methyl-1-{4-(methylsulfinyl)benzylidene}-*1H*-inden-3-yl]methyl)acetamide (35)

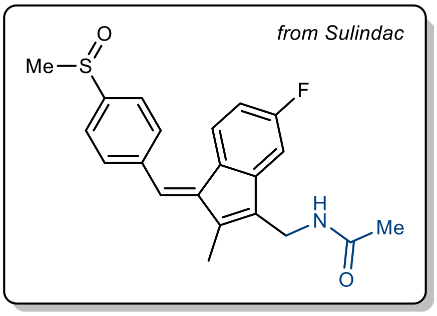

Isolated with DCM/Acetone = 60:40.; Yellow solid (41.5 mg, 56%); **m.p.** = 177 – 179 °C; **^{1}H NMR** (600 MHz, $CDCl_3$) δ 7.71 – 7.68 (m, 2H), 7.65 – 7.62 (m, 2H), 7.17 (s, 1H), 7.15 (dd, *J* = 8.4, 5.1 Hz, 1H), 6.92 (dd, *J* = 8.8, 2.5 Hz, 1H), 6.57 (td, *J* = 8.8, 2.5 Hz, 1H), 5.69 (s, 1H), 4.39 (d, *J* = 5.3 Hz, 2H), 2.79 (s, 3H), 2.23 (s, 3H), 2.01 (s, 3H); **^{13}C NMR** (150 MHz, $CDCl_3$) δ 170.2, 163.6 (d, *J* = 247.2 Hz), 146.2, 146.1, 145.8, 141.9, 139.6, 138.6, 135.1, 130.3, 129.7, 129.0, 124.0, 111.1 (d, *J* = 22.6 Hz), 106.4 (d, *J* = 24.1 Hz), 44.1, 34.5, 23.3, 10.5; **^{19}F NMR** (471 MHz, $CDCl_3$) δ -112.21; **IR** (cm^{-1}) 3254, 1659, 1546, 1463; **HRMS** (EI) m/z calcd. For $C_{21}H_{20}FNO_2S$ $[M]^+$: 369.1199, found: 369.1199.

N-[(R)-3-{(3R,5R,8R,9S,10S,13R,14S,17R)-3-Hydroxy-10,13-dimethylhexadecahydro-1H-cyclopenta[a]phenanthren-17-yl}butyl]acetamide (36)

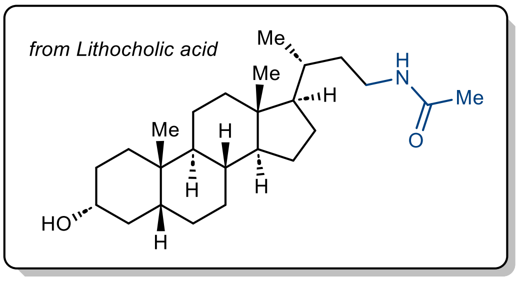

Isolated with DCM/Acetone = 60:40.; Colorless solid (42.3 mg, 54%); **m.p.** = 226 – 228 °C; **^{1}H NMR** (600 MHz, $CDCl_3$) δ 5.40 (s, 1H), 3.62 (tt, *J* = 11.1, 4.6 Hz, 1H), 3.36 – 3.28 (m, 1H), 3.20 – 3.11 (m, 1H), 1.99 – 1.92 (m, 4H), 1.88 – 1.71 (m, 4H), 1.70 – 1.53 (m, 5H), 1.53 – 1.47 (m, 1H), 1.46 – 1.34 (m, 5H), 1.35 – 1.27 (m, 1H), 1.27 – 1.17 (m, 4H), 1.16 – 0.96 (m, 6H), 0.94 (d, *J* = 6.6 Hz, 3H), 0.91 (s, 3H), 0.64 (s, 3H); **^{13}C NMR** (150 MHz, $CDCl_3$) δ 170.1, 72.0, 56.6, 56.3, 42.9, 42.2, 40.6, 40.3, 37.5, 36.6, 36.0, 35.9, 35.5, 34.7, 34.2, 30.7, 28.5, 27.3, 26.6, 24.3, 23.5, 23.5, 21.0, 18.8, 12.1; **IR** (cm^{-1}) 3436, 3320, 2927, 2880, 2861, 1656, 1550; **HRMS** (EI) m/z calcd. For $C_{25}H_{43}NO_2$ $[M]^+$: 389.3294, found: 389.3292.

The structure of the title compound was further confirmed by X-ray crystallographic analysis (see ***Appendix II****)*

(E)-N-(1-Phenylprop-1-en-2-yl)acetamide (37)

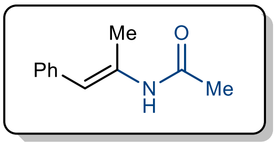

Column chromatography was conducted after washing with 1% TEA in dichloromethane. Isolated with DCM/Acetone = 90:10.; Colorless solid (21.3 mg, 61%); **m.p.** = 80 – 82 °C; **^{1}H NMR** (600 MHz, $CDCl_3$) δ 7.31 (t, *J* = 7.6 Hz, 2H), 7.22 (d, *J* = 7.7 Hz, 2H), 7.19 (t, J = 7.5 Hz, 1H), 7.01 (s, 1H), 6.68 (s, 1H), 2.10 (s, 3H), 2.09 (s, 3H); **^{13}C NMR** (150 MHz, $CDCl_3$) δ 168.7, 137.2, 132.9, 129.1, 128.2, 126.2, 116.3, 24.9, 18.1; **IR** (cm^{-1}) 3278, 3182, 3080, 1662, 1544, 1280, 856, 744, 697, 608, 506; **HRMS** (EI) m/z calcd. For $C_{11}H_{13}NO$ $[M]^+$: 175.0997, found: 175.0998.

VI. Substrate Scope of Decarboxylative Amidation with Aryl Carboxylic Acids

1. Additional Reaction Optimization using benzoic acid

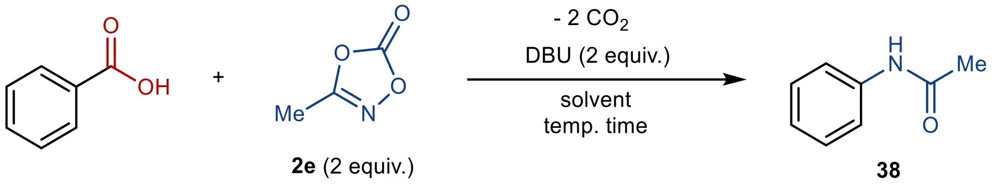

To an oven-dried 4 mL screw-capped vial equipped with oval-shaped stirring bar were added benzoic acid (0.100 mmol, 12.2 mg), 1,8-Diazabicyclo[5.4.0]undec-7-ene (DBU, 2.00 equiv., 0.200 mmol, 30.5 mg), and indicated solvent under atmospheric conditions. To the reaction mixture was added 3-methyl-1,4,2-dioxazol-5-one (**2e**, 2.00 equiv., 0.200 mmol, 20.2 mg) and stirred for 4 h at indicated temperature. Product yield was measured by ^{1}H-NMR analysis of the crude mixture in the presence of internal standard (1,3,5-trimethoxybenzene) in DMSO-d_6.

Supplementary Table 3 | Optimization of the transition metal-free decarboxylative amidation with benzoic acid

entry	solvent (conc.)	temp (°C)	time (h)	yield of 38 (%)
**1**	DMSO (0.2 M)	r.t.	4	19
**2**	DMSO (0.2 M)	60	4	45
**3**	DMSO (1.0 M)	60	4	41
**4**	DMSO (1.0 M)	60	12	32
**5**	DMF (0.2 M)	60	4	30
**6**	DCM (0.2 M)	60	4	37
**7**[a]	DMSO (0.2 M)	60	4	5
**8**[b]	**DMSO (0.2 M)**	**60**	**4**	**56**

[a]Run with 0.100 mmol of **2e** and 0.200 mmol of benzoic acid. [b]Run with 3 equiv of **2e**.

2. Substrate scope investigation using aryl carboxylic acid

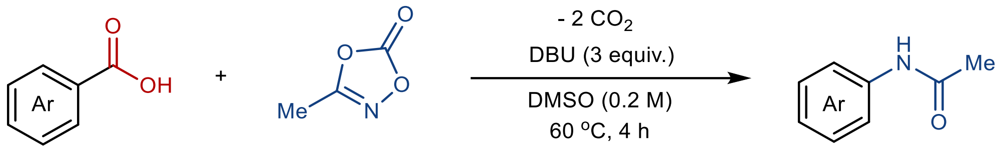

2e (3 equiv)

To an oven-dried 4 mL screw-capped vial equipped with oval-shaped stirring bar were added carboxylic acids (0.200 mmol), 1,8-Diazabicyclo[5.4.0]undec-7-ene (DBU, 2.00 equiv., 0.400 mmol, 60.9 mg), and dimethylsulfoxide (DMSO, 1.00 mL, 0.200 M) under atmospheric conditions. To the reaction mixture was added 3-methyl-1,4,2-dioxazol-5-one (**2e**, 3.00 equiv., 0.600 mmol, 60.6 mg) and stirred for 4 h at 60 °C. After reaction completion, the crude reaction mixture was diluted with dichloromethane (DCM, 5.0 mL), added 1N HCl aqueous solution (10 mL), and extracted with DCM (5 mL x 3 times). The combined organic layer washed with $KHSO_4$ aqueous solution (30 mL), dried over $MgSO_4$, filtered, and concentrated under the reduced pressure. The crude mixture was subjected to silica column chromatography to provide the purified desired N-arylamide products (eluent: Dichloromethane/Acetone, 95:5 ~ 90:10).

***N*-Phenylacetamide (38)**

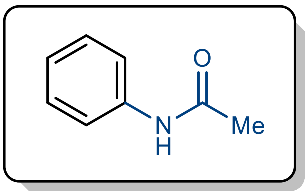

Isolated with DCM/Acetone = 90:10.; Colorless solid (12.1 mg, 45%); **^{1}H NMR** (500 MHz, $CDCl_3$) δ 7.53 – 7.47 (m, 2H), 7.42 (s, 1H), 7.31 (t, *J* = 8.0 Hz, 2H), 7.13 – 7.07 (m, 1H), 2.17 (s, 3H); **^{13}C NMR** (125 MHz, $CDCl_3$) δ 168.6, 138.0, 129.1, 124.4, 120.0, 24.7. *Data consistent with those previously reported.*[15]

***N*-(*p*-Tolyl)acetamide (39)**

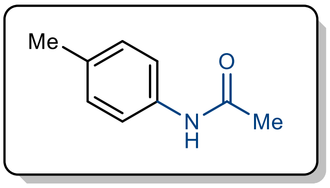

Isolated with DCM/Acetone = 90:10.; Colorless solid (14.9 mg, 50%); **^{1}H NMR** (500 MHz, $CDCl_3$) δ 7.42 – 7.36 (m, 2H), 7.28 (s, 1H), 7.13 (d, J = 8.1 Hz, 2H), 2.33 (s, 3H), 2.18 (s, 3H). **^{13}C NMR** (125 MHz, $CDCl_3$) δ 168.4, 135.4, 134.1, 129.6, 120.2, 24.7, 21.0. *Data consistent with those previously reported.*[15]

***N*-(3-Methoxyphenyl)acetamide (40)**

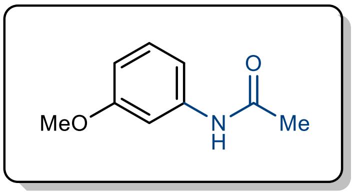

Isolated with DCM/Acetone = 95:5 without workup process; Colorless solid (21.6 mg, 65%); **^{1}H NMR** (500 MHz, $CDCl_3$) δ 8.35 (dd, *J* = 8.1, 1.6 Hz, 1H), 7.76 (s, 1H), 7.03 (td, *J* = 7.8, 1.7 Hz, 1H), 6.95 (td, *J* = 7.7, 1.4 Hz, 1H), 6.87 (dd, *J* = 8.1, 1.4 Hz, 1H), 3.88 (s, 3H), 2.20 (s, 3H); **^{13}C NMR** (125 MHz, $CDCl_3$) δ 168.3, 147.8, 127.8, 123.7, 121.2, 119.9, 110.0, 55.8, 25.1. *Data consistent with those previously reported.*[16]

N-{4-(4,4,5,5-Tetramethyl-1,3,2-dioxaborolan-2-yl)phenyl}acetamide (41)

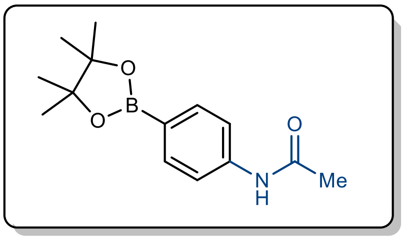

Isolated with DCM/Acetone = 90:10.; Colorless solid (20.8 mg, 40%); **^{1}H NMR** (500 MHz, $CDCl_3$) δ 7.76 (d, *J* = 8.1 Hz, 2H), 7.51 (d, *J* = 8.0 Hz, 2H), 7.35 (s, 1H), 2.17 (d, *J* = 1.7 Hz, 3H), 1.33 (s, 12H); **^{13}C NMR** (125 MHz, $CDCl_3$, *one carbon peak is missing due to boron coupling*) δ 168.6, 140.6, 135.9, 118.7, 83.9, 25.0, 24.9. *Data consistent with those previously reported.*[17]

N-[6-{3-(Adamantan-1-yl)-4-methoxyphenyl}naphthalen-2-yl]acetamide (42)

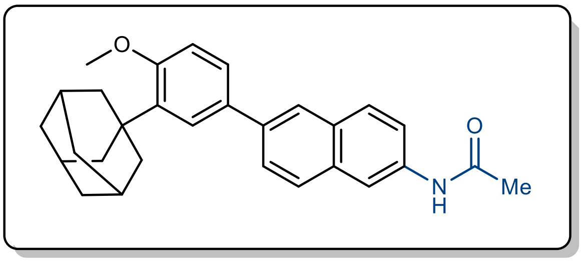

Isolated with DCM/Acetone = 95:5.; Colorless solid (44.3 mg, 52%); **m.p.** = 225 – 227 °C; **^{1}H NMR** (500 MHz, THF-d_8) δ 9.14 (s, 1H), 8.32 (d, *J* = 2.2 Hz, 1H), 7.93 (d, *J* = 1.8 Hz, 1H), 7.79 (s, 1H), 7.77 (s, 1H), 7.68 (dd, *J* = 8.5, 1.8 Hz, 1H), 7.58 (d, *J* = 2.3 Hz, 1H), 7.54 (dd, *J* = 8.8, 2.1 Hz, 1H), 7.51 (dd, *J* = 8.4, 2.3 Hz, 1H), 7.02 (d, *J* = 8.4 Hz, 1H), 3.87 (s, 3H), 2.22 (d, *J* = 2.9 Hz, 6H), 2.12 – 2.04 (m, 6H), 1.89 – 1.77 (m, 6H); **^{13}C NMR** (125 MHz, THF-d_8) δ 168.4, 159.4, 139.2, 138.4, 138.1, 134.2, 134.0, 131.6, 129.1, 128.6, 126.6, 126.2, 126.2, 125.2, 120.7, 115.8, 112.9, 55.4, 41.6, 38.1, 38.0, 30.4, 24.2; **IR** (cm^{-1}) 3307, 2901, 2850, 1683, 1543, 1503, 1394, 1235, 1025, 874, 803, 730, 591, 464; **HRMS** (EI) m/z calcd. For $C_{29}H_{31}NO_2$ $[M]^+$: 425.2355, found: 425.2357.

VII. Substrate Scope of Decarboxylative Amidation with Chiral Carboxylic Acids

(*S*)-2-Phenylpropanoic acid, (*S*)-cyclohex-3-ene-1-carboxylic acid, (*R*)-2-methoxy-2-phenylacetic acid, (*S*)-tetrahydrofuran-2-carboxylic acid, (*tert*-butoxycarbonyl)-*D*-proline, (1*S*,3*R*)-3-{(tert-butoxycarbonyl)amino} cyclohexane-1-carboxylic acid, and (*S*)-2-(6-methoxynaphthalen-2-yl)propanoic acid were purchased from TCI chemical. (*R*)-1,2,3,4-Tetrahydronaphthalene-1-carboxylic acid was purchased from Acros Organics. (*S*)-2-(5-Bromo-6-methoxynaphthalen-2-yl)propanoic acid was purchased from aablock. (*S*)-2-(4-Isobutylphenyl)propanoic acid was purchased from Sigma Aldrich. (2*R*,4*S*)-5-([1,1'-Biphenyl]-4-yl)-4-{(tert-butoxycarbonyl)amino}-2-methylpentanoic acid and (R)-2-{(1*R*,4*R*,4a*S*,8a*S*)-4,7-dimethyl-1,2,3,4,4a,5,6,8a-octahydronaphthalen-1-yl}propanoic acid were purchased from BLD Pharm. In the case of (*S*)-2-(6-methoxynaphthalen-2-yl)propanoic acid, enantiomeric ratio was measured by HPLC analysis. CHIRALPAK AD-H, 32 °C; *n*-hexane:*i*-PrOH = 80:20, 0.5 mL/min, 250 nm, t_{R1} (major) = 14.7 min, t_{R2} (minor) = 13.5 min, >99:1 e.r.;

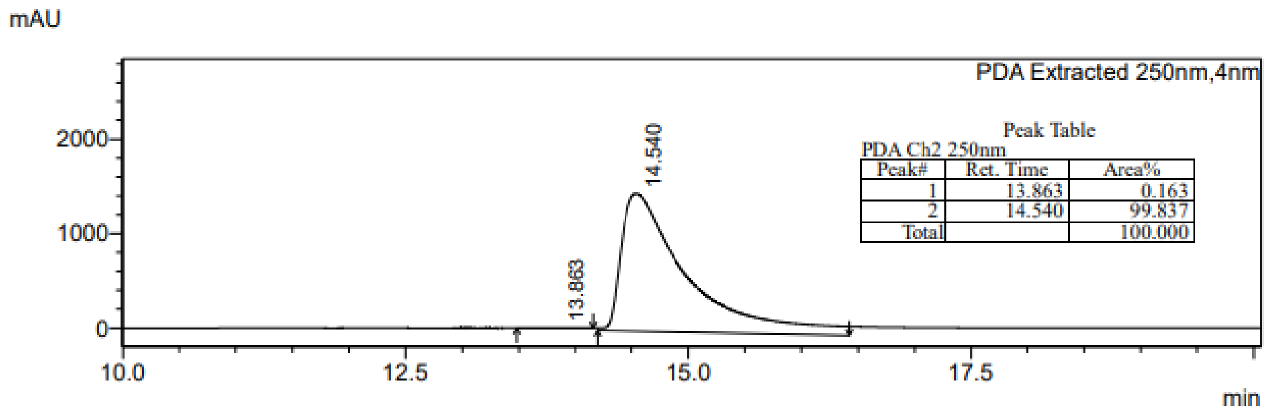

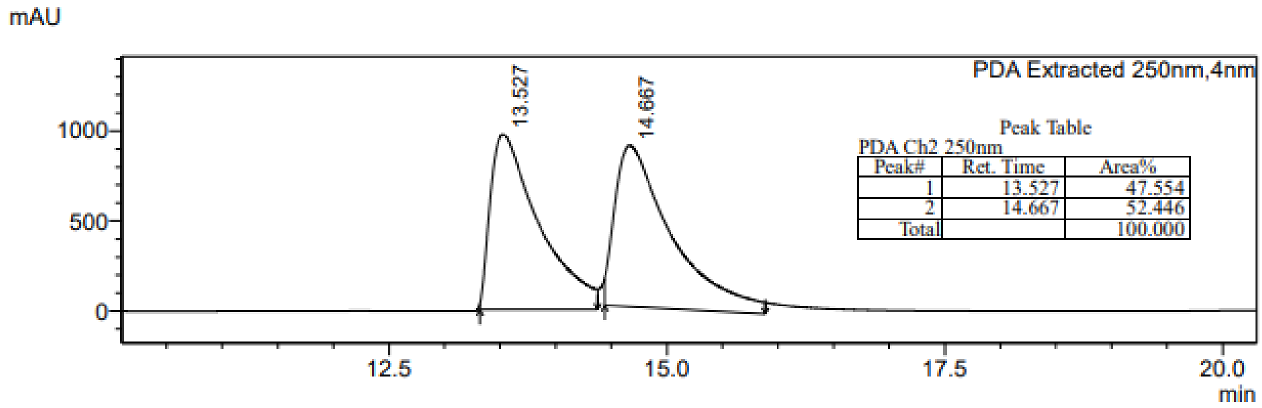

Supplementary Fig. 1 | HPLC traces of (*S*)-Naproxen (top) and corresponding racemic mixture (bottom).

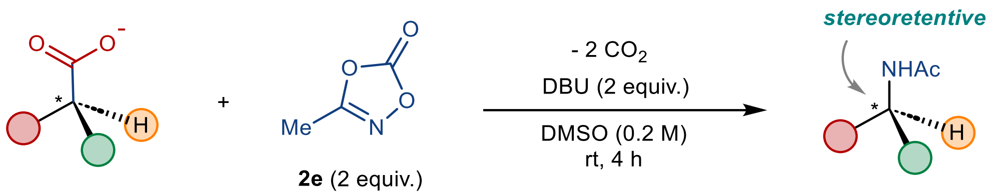

To an oven-dried 4 mL screw-capped vial equipped with oval-shaped stirring bar were added carboxylic acids (0.200 mmol), 1,8-Diazabicyclo[5.4.0]undec-7-ene (DBU, 2.00 equiv., 0.400 mmol, 60.9 mg), and anhydrous dimethylsulfoxide (DMSO, 1.00 mL, 0.200 M) under atmospheric conditions. To the reaction mixture was added 3-methyl-1,4,2-dioxazol-5-one (**2e**, 2.00 equiv., 0.400 mmol, 40.4 mg) and stirred for 4 h at room temperature. After reaction completion, the crude reaction mixture was diluted with dichloromethane (DCM, 5.0 mL), added 1N HCl aqueous solution (10 mL), and extracted with DCM (5 mL x 3 times). The combined organic layer was dried over $MgSO_4$, filtered, and concentrated under the reduced pressure. The crude mixture was subjected to silica column chromatography to provide the purified desired N-alkylamide products (eluent: Dichloromethane/Acetone, 100:0 ~ 50:50). Values of enantiomeric ratio (e.r.) were determined by HPLC analysis. The authentic samples of racemic products were synthesized by our present decarboxylative amination method using as racemic carboxylic acids.

(*S*)-*N*-(1-Phenylethyl)acetamide (43)

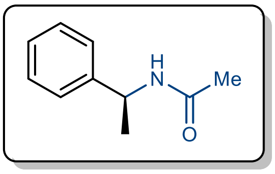

Isolated with DCM/Acetone = 85:15.; Colorless solid (26.7 mg, 83%); **^{1}H NMR** (600 MHz, $CDCl_3$) δ 7.37 – 7.30 (m, 4H), 7.29 – 7.24 (m, 1H), 5.73 (s, 1H), 5.13 (p, J = 7.1 Hz, 1H), 1.98 (s, 3H), 1.49 (d, J = 6.9 Hz, 3H); **^{13}C NMR** (150 MHz, $CDCl_3$) δ 169.2, 143.3, 128.8, 127.6, 126.3, 48.9, 23.6, 21.8; **Specific Rotation** $[\alpha]_D^{29}$ = –149.1° (c 0.51, CH_2Cl_2); **HPLC Analysis**. CHIRALPAK OD-H, 27 °C; *n*-hexane:*i*-PrOH = 90:10, 0.5 mL/min, 210 nm, t_{R1} (major) = 20.2 min, t_{R2} (minor) = 21.6 min, >99:1 e.r.; *Data consistent with those previously reported.*[18]

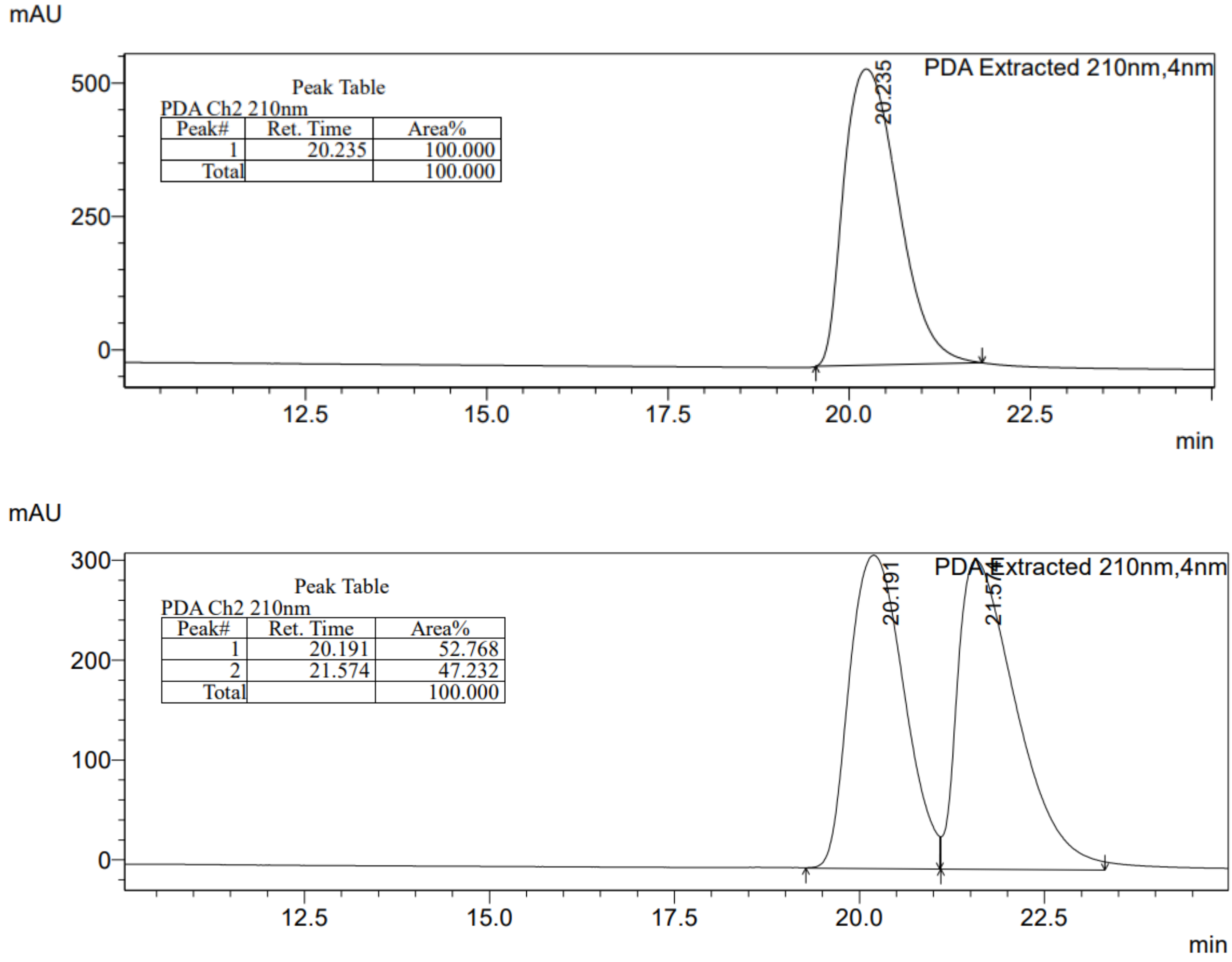

Supplementary Fig. 2 | HPLC traces of **43** (top) and corresponding racemic mixture (bottom).

(*R*)-*N*-(1,2,3,4-Tetrahydronaphthalen-1-yl)acetamide (44)

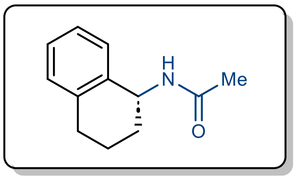

Isolated with DCM/Acetone = 80:20.; Colorless powder (30.5 mg, 81%); **^{1}H NMR** (600 MHz, $CDCl_3$) δ 7.29 – 7.26 (m, 1H), 7.20 – 7.14 (m, 2H), 7.12 – 7.08 (m, 1H), 5.67 (s, 1H), 5.22 – 5.14 (m, 1H), 2.78 (qt, J = 17.1, 5.8 Hz, 2H), 2.08 – 2.03 (m, 1H), 2.02 (s, 3H), 1.87 – 1.78 (m, 3H); **^{13}C NMR** (150 MHz, $CDCl_3$) δ 168.8, 139.1, 136.8, 129.3, 128.8, 127.4, 126.4, 47.6, 30.8, 29.3, 24.0, 20.4; **Specific Rotation** $[\alpha]_D^{29}$ = 90.8° (*c* 0.47, CH_2Cl_2); **HPLC Analysis**. CHIRALPAK AD-H, 32 °C; *n*-hexane:*i*-PrOH = 80:20, 0.5 mL/min, 210 nm, t_{R1} (major) = 7.8 min, t_{R2} (minor) = 8.9 min, >99:1 e.r.; *Data consistent with those previously reported*.[19]

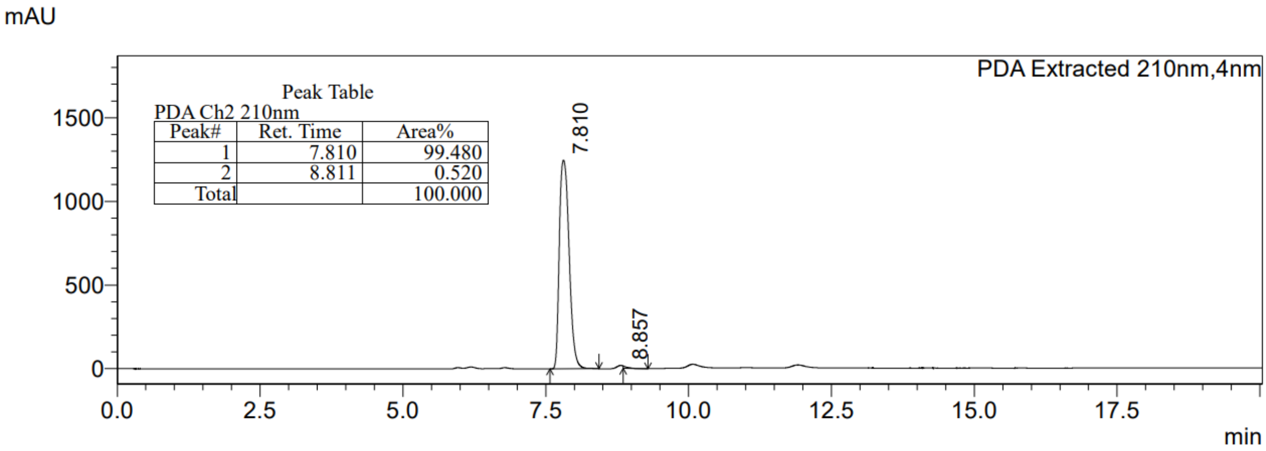

Peak#	Ret. Time	Area%
1	7.810	99.480
2	8.811	0.520
Total		100.000

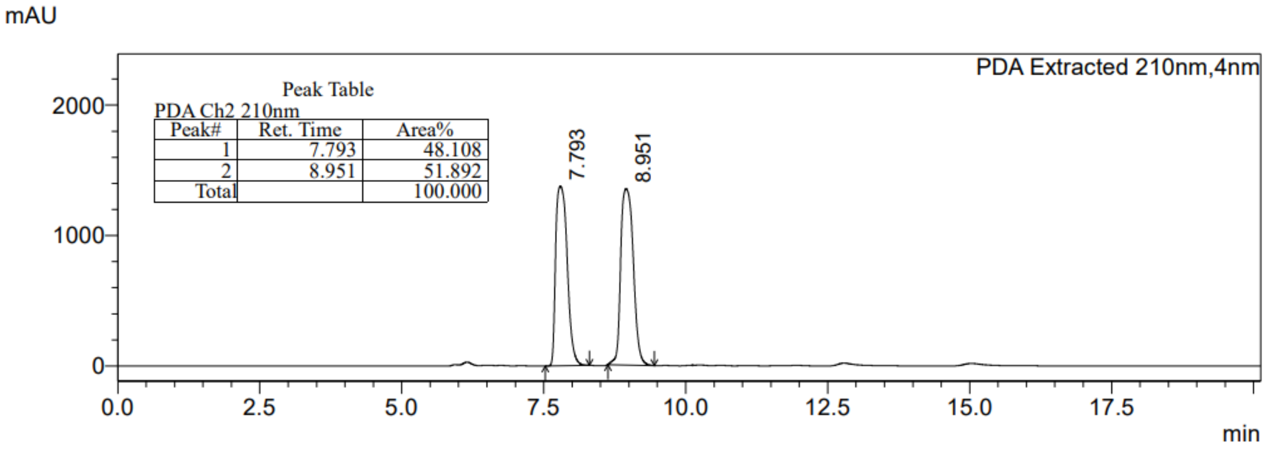

Peak#	Ret. Time	Area%
1	7.793	48.108
2	8.951	51.892
Total		100.000

Supplementary Fig. 3 | HPLC traces of **44** (top) and corresponding racemic mixture (bottom).

(*S*)-*N*-(Cyclohex-3-en-1-yl)acetamide (45)

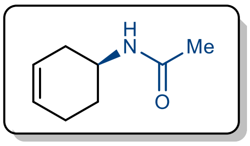

Isolated with DCM/Acetone = 70:30.; Colorless solid (20.1 mg, 72%); **m.p.** = 74 – 76 °C; **^{1}H NMR** (600 MHz, $CDCl_3$) δ 5.72 – 5.66 (m, 1H), 5.63 – 5.58 (m, 1H), 5.47 (s, 1H), 4.11 (dddd, *J* = 12.6, 8.2, 6.7, 3.0 Hz, 1H), 2.43 – 2.35 (m, 1H), 2.21 – 2.07 (m, 2H), 1.97 (s, 3H), 1.90 – 1.82 (m, 2H), 1.57 (dddd, *J* = 12.7, 9.5, 8.0, 5.9 Hz, 1H); **^{13}C NMR** (150 MHz, $CDCl_3$) δ 169.5, 127.2, 124.5, 44.6, 31.8, 28.0, 23.8, 23.5; **IR** (cm^{-1}) 3290, 3032, 2917, 2839, 1635, 1549, 1360, 1281, 651, 604, 511; **Specific Rotation** $[\alpha]_D^{29}$ = –14.4° (*c* 0.46, CH_2Cl_2); **HRMS** (FAB) m/z calcd. For $C_8H_{14}NO$ $[M+H]^+$: 140.1075, found: 140.1078; **HPLC Analysis**. CHIRALPAK AS-H, 32 °C; *n*-hexane:*i*-PrOH = 95:5, 0.5 mL/min, 210 nm, t_{R1} (minor) = 145.5 min, t_{R2} (major) = 150.2 min, 99:1 e.r.

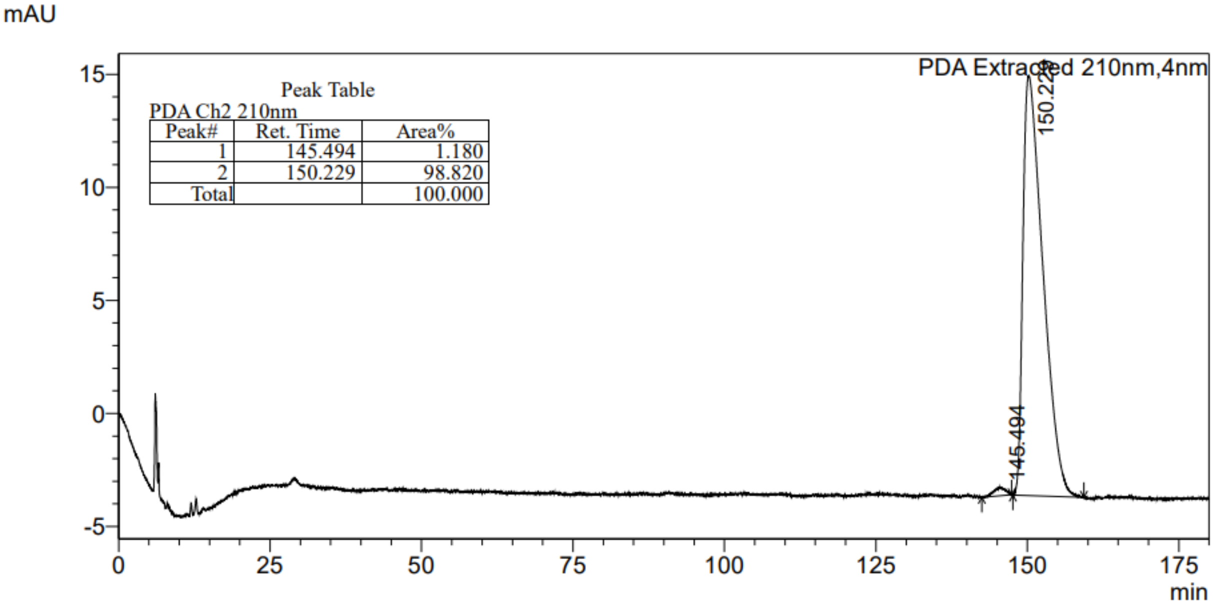

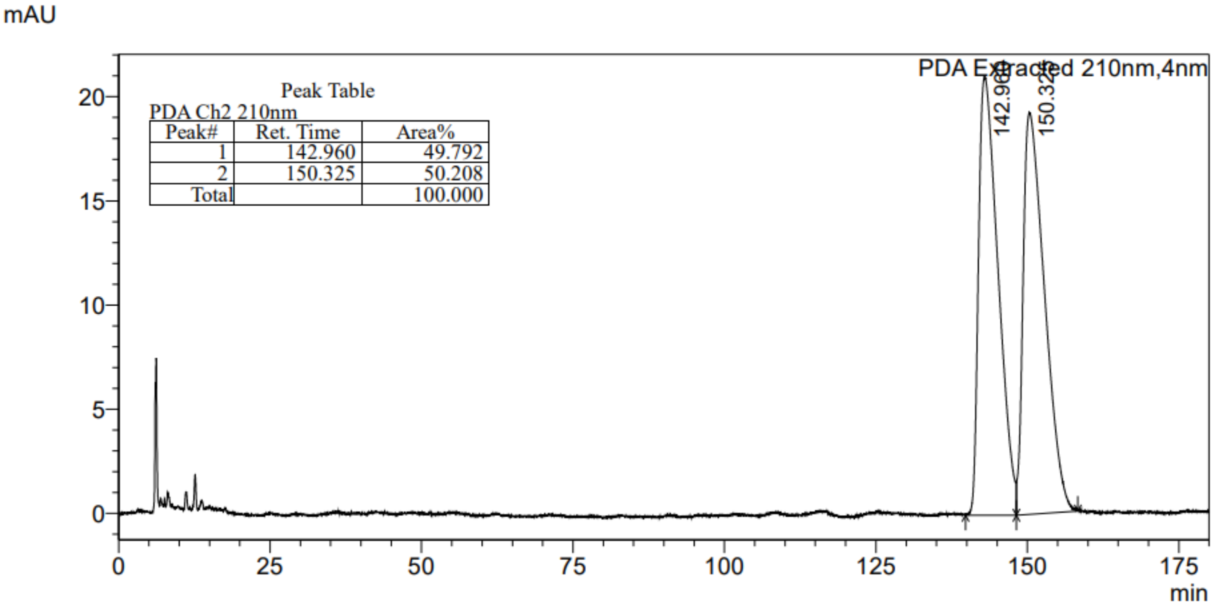

Supplementary Fig. 4 | HPLC traces of **45** (top) and corresponding racemic mixture (bottom).

(*R*)-*N*-{Methoxy(phenyl)methyl}acetamide (46)

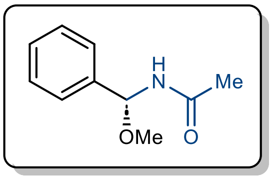

Washed with brine rather than 1N HCl. Isolated with DCM/Acetone = 90:10.; Colorless solid (17.0 mg, 47%); **^{1}H NMR** (600 MHz, $CDCl_3$) δ 7.44 – 7.40 (m, 2H), 7.40 – 7.36 (m, 2H), 7.35 – 7.32 (m, 1H), 6.12 (d, *J* = 9.5 Hz, 1H), 5.93 (d, *J* = 9.2 Hz, 1H), 3.46 (s, 3H), 2.05 (s, 3H); **^{13}C NMR** (150 MHz, $CDCl_3$) δ 170.2, 139.4, 128.8, 128.7, 126.0, 81.6, 56.2, 23.6; **Specific Rotation** $[\alpha]_D^{26}$ = 6.1° (*c* 0.07, CH_2Cl_2); **HPLC Analysis**. CHIRALPAK AD-H, 28 °C; *n*-hexane:*i*-PrOH = 90:10, 0.5 mL/min, 210 nm, t_{R1} (minor) = 11.5 min, t_{R2} (major) = 12.5 min, 98:2 e.r. *Data consistent with those previously reported.*[20]

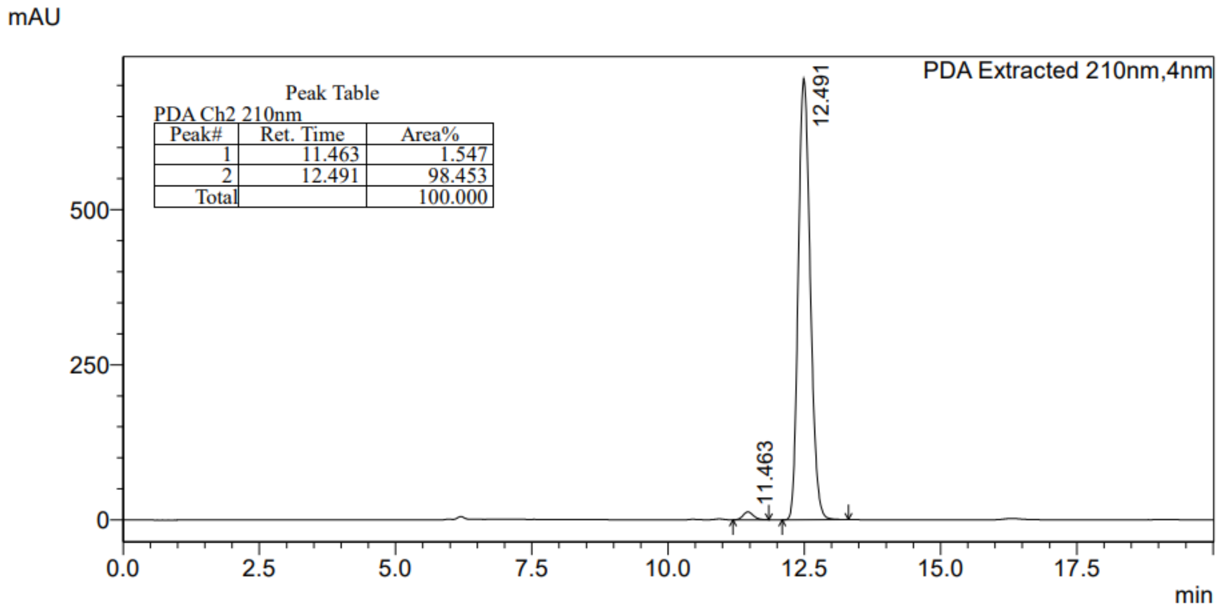

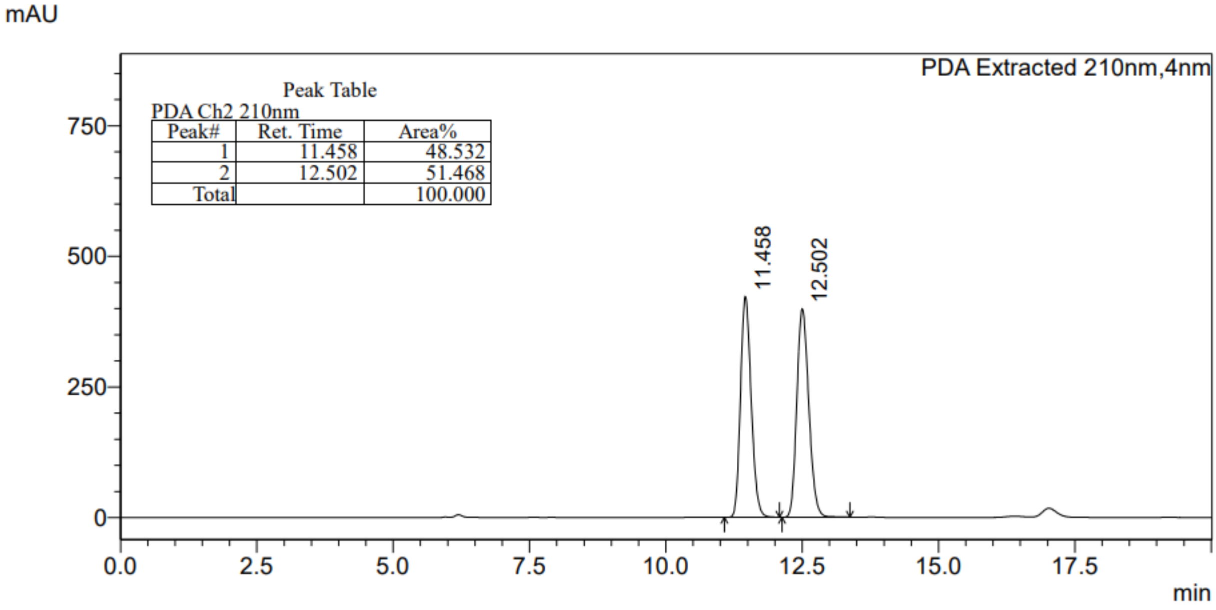

Supplementary Fig. 5 | HPLC traces of **46** (top) and corresponding racemic mixture (bottom).

(*S*)-*N*-(Tetrahydrofuran-2-yl)acetamide (47)

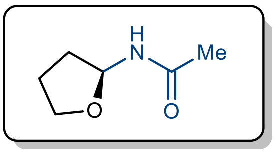

Isolated with DCM/Acetone 50:50 without workup process; Colorless liquid (19.1 mg, 74%); **^{1}H NMR** (600 MHz, $CDCl_3$) δ 5.91 (s, 1H), 5.70 (ddd, *J* = 8.3, 6.4, 4.8 Hz, 1H), 3.93 (dt, *J* = 8.5, 6.7 Hz, 1H), 3.80 (dt, *J* = 8.5, 6.9 Hz, 1H), 2.18 (ddt, *J* = 13.2, 8.3, 6.7 Hz, 1H), 1.98 (s, 3H), 1.94 (dtd, *J* = 13.3, 6.7, 1.7 Hz, 2H), 1.71 (dddd, *J* = 12.8, 7.9, 6.6, 4.7 Hz, 1H); **^{13}C NMR** (150 MHz, $CDCl_3$) δ 170.1, 81.2, 67.6, 32.2, 24.8, 23.6; **IR** (cm^{-1}) 3291, 2969, 1655, 1537, 1367, 1290, 1189, 1137, 1040, 922, 599, 515; **HRMS** (FAB) m/z calcd. For $C_6H_{12}NO_2$ $[M+H]^+$: 130.0868, found: 130.0866; **Specific Rotation** $[\alpha]_D^{29}$ = 75.4° (*c* 0.41, CH_2Cl_2); **HPLC Analysis**. CHIRALPAK AD-H, 32 °C; n-hexane:*i*-PrOH = 90:10, 0.5 mL/min, 210 nm, t_{R1} (major) = 12.8 min, t_{R2} (minor) = 13.6 min, 99:1 e.r.

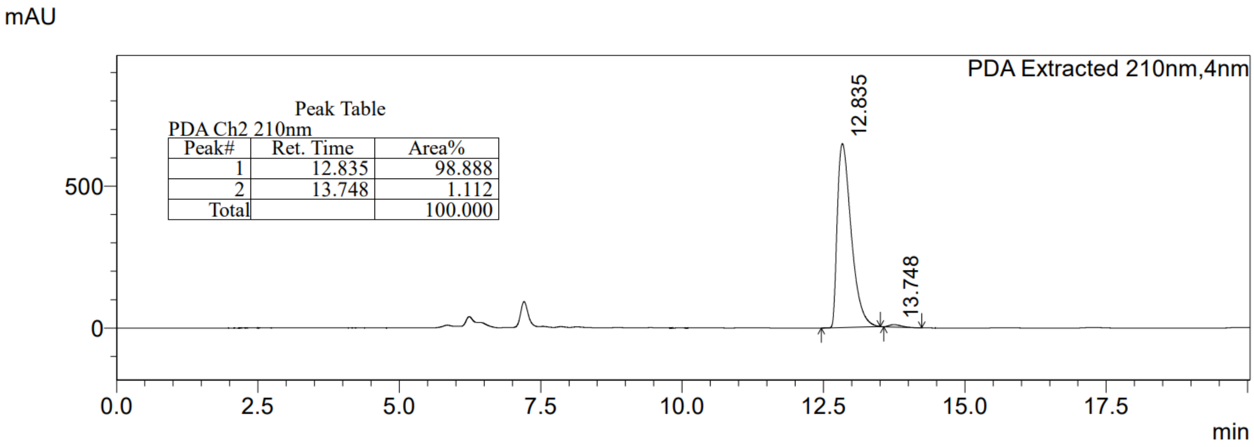

Peak Table
PDA Ch2 210nm

Peak#	Ret. Time	Area%
1	12.835	98.888
2	13.748	1.112
Total		100.000

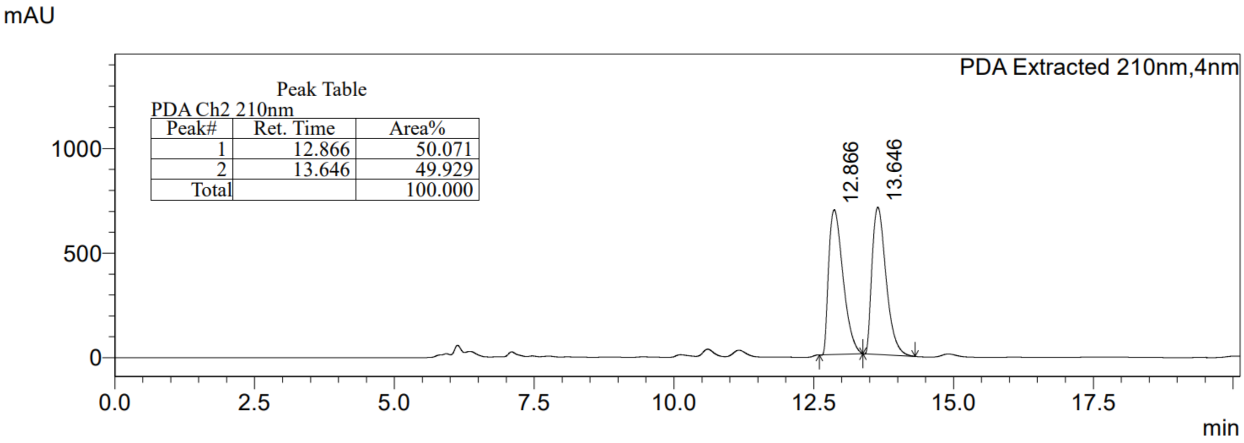

Peak Table
PDA Ch2 210nm

Peak#	Ret. Time	Area%
1	12.866	50.071
2	13.646	49.929
Total		100.000

Supplementary Fig. 6 | HPLC traces of **47** (top) and corresponding racemic mixture (bottom).

tert-Butyl (R)-2-acetamidopyrrolidine-1-carboxylate (48)

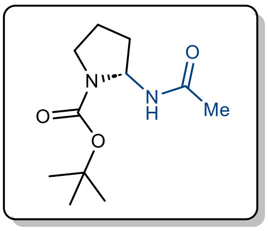

Washed with brine rather than 1N HCl.Isolated with DCM/Acetone = 70:30.; Colorless solid (29.7 mg, 65%); **^{1}H NMR** (600 MHz, $CDCl_3$) δ 5.77 (s, 1H), 5.69 – 5.40 (m, 1H), 3.50 – 3.39 (m, 1H), 3.34 – 3.20 (m, 1H), 2.11 – 1.79 (m, 7H), 1.43 (s, 9H); **^{13}C NMR** (100 MHz, $CDCl_3$) δ 168.9, 154.2, 80.3, 64.2, 46.1, 33.9, 28.5, 23.5, 22.5; **Specific Rotation** $[\alpha]_D^{29}$ = 29.6° (*c* 0.44, CH_2Cl_2); **HPLC Analysis**. CHIRALCEL OD-H, 28 °C; *n*-hexane:*i*-PrOH = 80:20, 0.5 mL/min, 210 nm, t_{R1} (minor) = 7.5 min, t_{R2} (major) = 8.8 min, >99:1 e.r. *Data consistent with those previously reported.*[21]

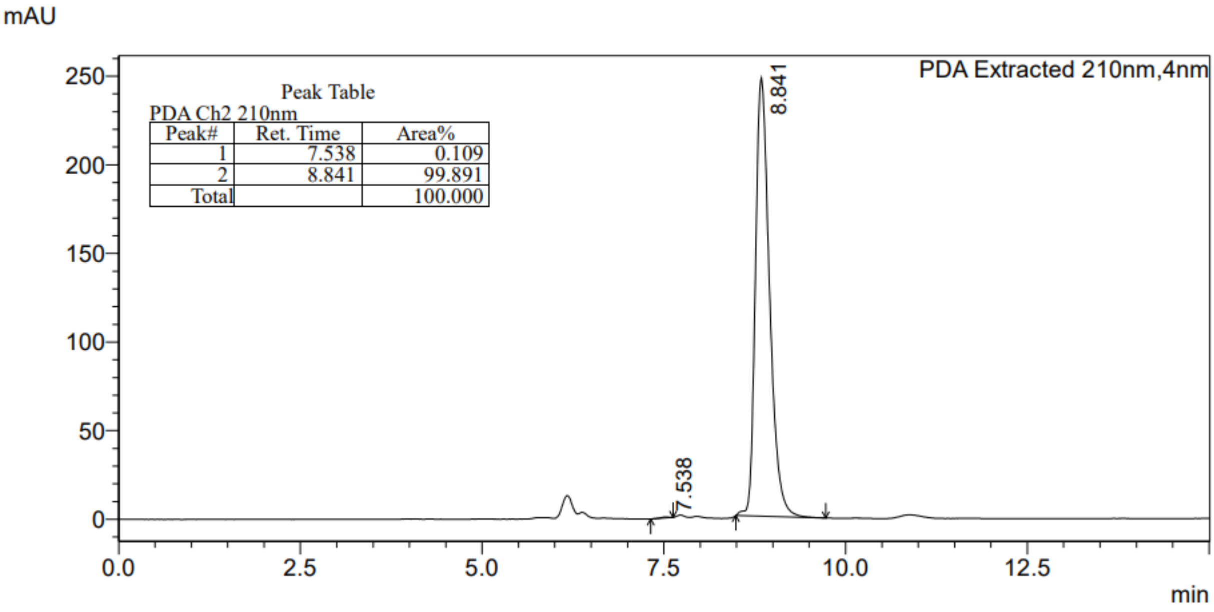

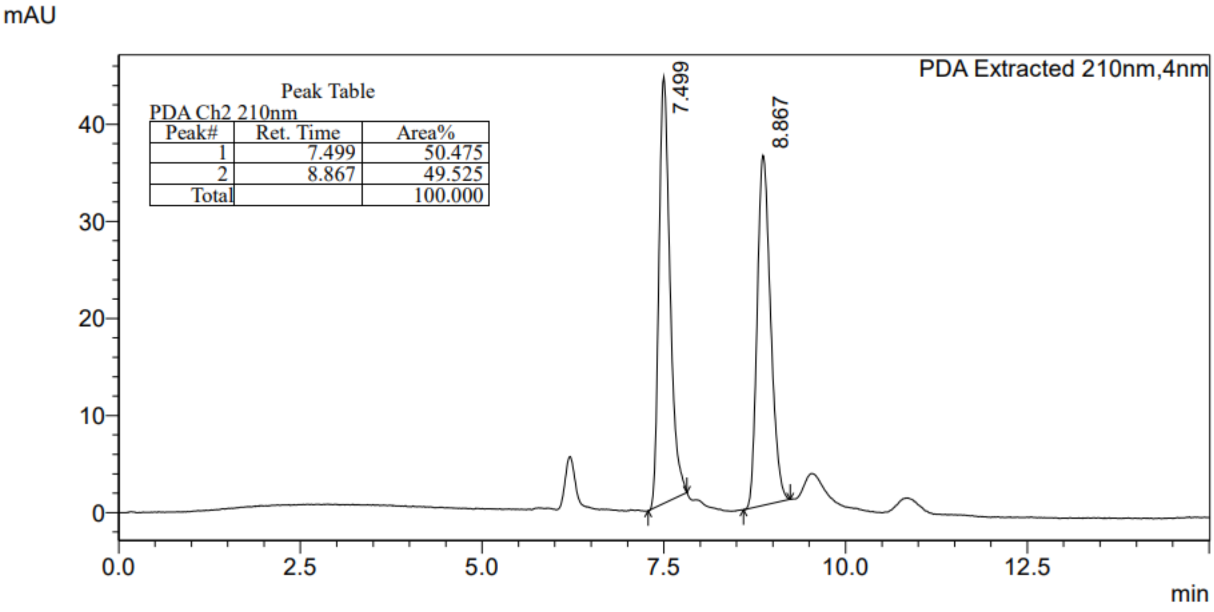

Supplementary Fig. 7 | HPLC traces of **48** (top) and corresponding racemic mixture (bottom).

tert-Butyl {(_1R,3S_)-3-acetamidocyclohexyl}carbamate (49)

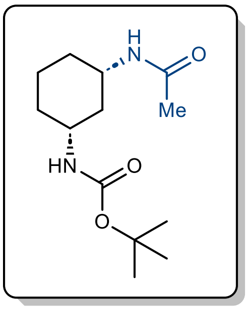

Isolated with DCM/Acetone = 60:40.; Colorless solid (36.9 mg, 72%); **m.p.** = 185 – 187 °C; **^{1}H NMR** (600 MHz, $CDCl_3$) δ 5.37 (d, *J* = 8.1 Hz, 1H), 4.43 (s, 1H), 3.80 (tdt, *J* = 11.9, 8.0, 4.0 Hz, 1H), 3.48 (s, 1H), 2.30 – 2.21 (m, 1H), 2.01 – 1.91 (m, 5H), 1.81 – 1.72 (m, 1H), 1.49 – 1.34 (m, 10H), 1.04 – 0.90 (m, 3H); **^{13}C NMR** (100 MHz, $CDCl_3$) δ 169.2, 155.1, 79.4, 48.8, 47.6, 40.2, 32.8, 32.5, 28.5, 23.6, 23.0; **IR** (cm^{-1}) 3330, 3270, 2973, 2933, 2858, 1684, 1655, 1536; **HRMS** (EI) m/z calcd. For $C_{13}H_{24}N_2O_3$ $[M]^+$: 256.1787, found: 256.1785; **Specific Rotation** $[\alpha]_D^{29}$ = –10.6° (*c* 0.59, CH_2Cl_2); **HPLC Analysis**. CHIRALPAK AD-H, 28 °C; *n*-hexane:*i*-PrOH = 90:10, 0.5 mL/min, 210 nm, t_{R1} (major) = 12.6 min, t_{R2} (minor) = 18.6 min, >99:1 e.r.

*The structure of the title compound was further confirmed by X-ray crystallographic analysis (see **Appendix II**)*

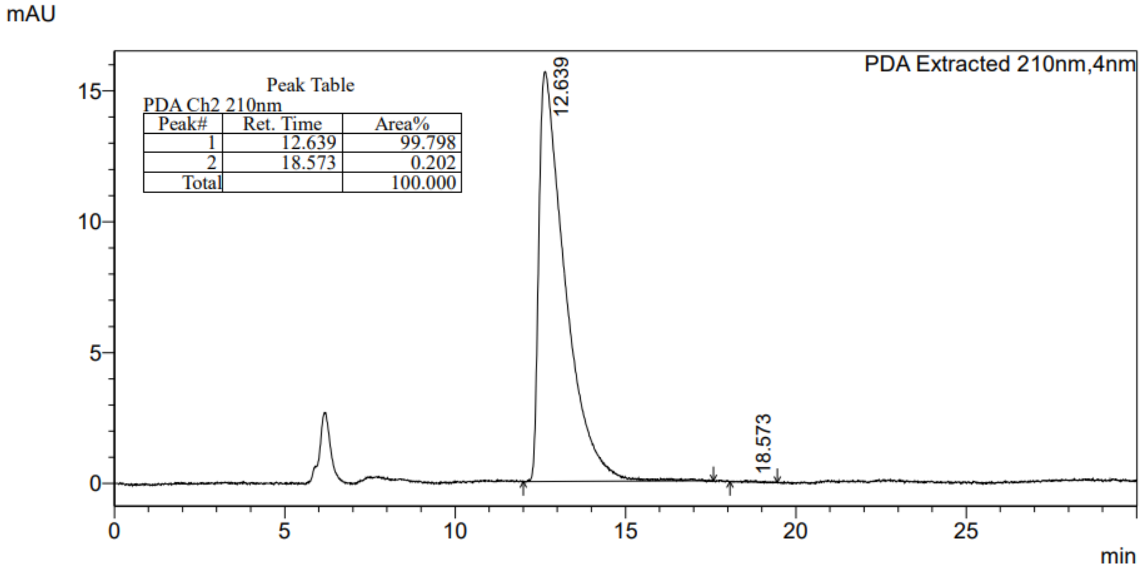

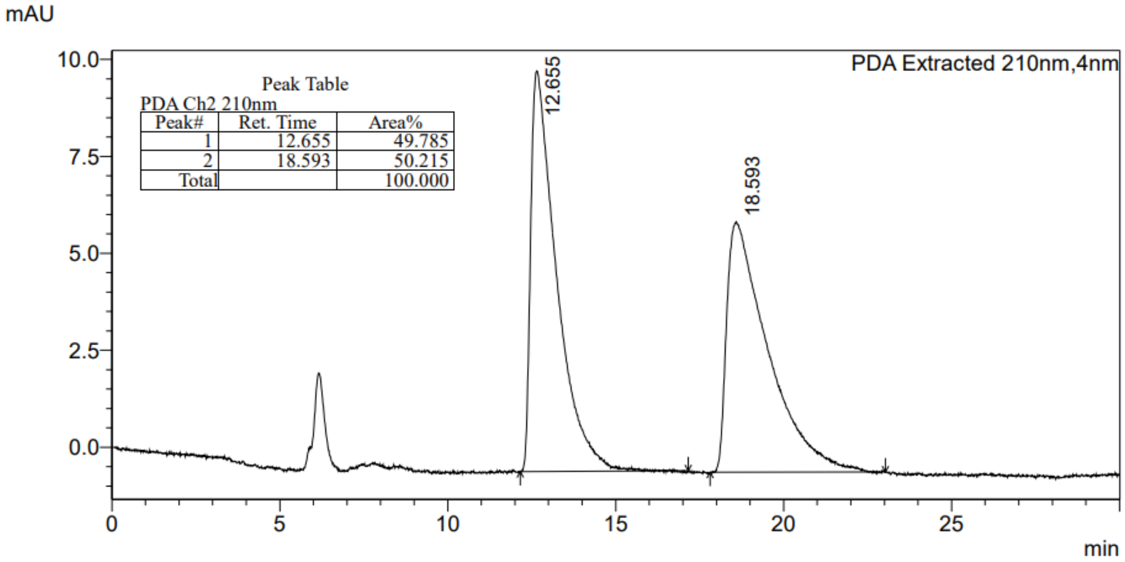

Supplementary Fig. 8 | HPLC traces of **49** (top) and corresponding racemic mixture (bottom).

(*S*)-*N*-{1-(6-Methoxynaphthalen-2-yl)ethyl}acetamide (52)

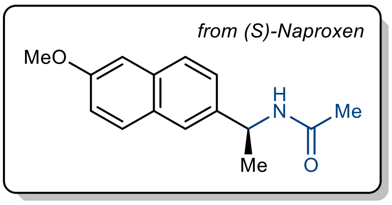

Isolated with DCM/Acetone = 85:15.; Colorless solid (42.1 mg, 87%); **m.p.** = 145 – 147 °C; **^{1}H NMR** (600 MHz, $CDCl_3$) δ 7.71 (dd, *J* = 8.7, 4.7 Hz, 2H), 7.69 – 7.67 (m, 1H), 7.40 (dd, *J* = 8.5, 1.8 Hz, 1H), 7.15 (dd, *J* = 8.9, 2.5 Hz, 1H), 7.12 – 7.10 (m, 1H), 5.75 (d, *J* = 7.5 Hz, 1H), 5.27 (p, *J* = 7.1 Hz, 1H), 3.92 (s, 3H), 2.00 (s, 3H), 1.57 (d, *J* = 6.9 Hz, 3H); **^{13}C NMR** (150 MHz, $CDCl_3$) δ 169.2, 157.9, 138.3, 134.0, 129.5, 128.9, 127.5, 125.5, 124.6, 119.2, 105.8, 55.5, 48.9, 23.7, 21.7; **IR** (cm^{-1}) 3272, 3061, 2971, 2928, 1630, 1604, 1542; **HRMS** (EI) m/z calcd. For $C_{15}H_{17}NO_2$ $[M]^+$: 243.1259, found: 243.1260; **Specific Rotation** $[\alpha]_D^{29}$ = –147.4° (*c* 0.47, CH_2Cl_2); **HPLC Analysis**. CHIRALPAK AD-H, 28 °C; *n*-hexane:*i*-PrOH = 80:20, 0.5 mL/min, 210 nm, t_{R1} (minor) = 9.1 min, t_{R2} (major) = 10.8 min, >99:1 *e.r.*

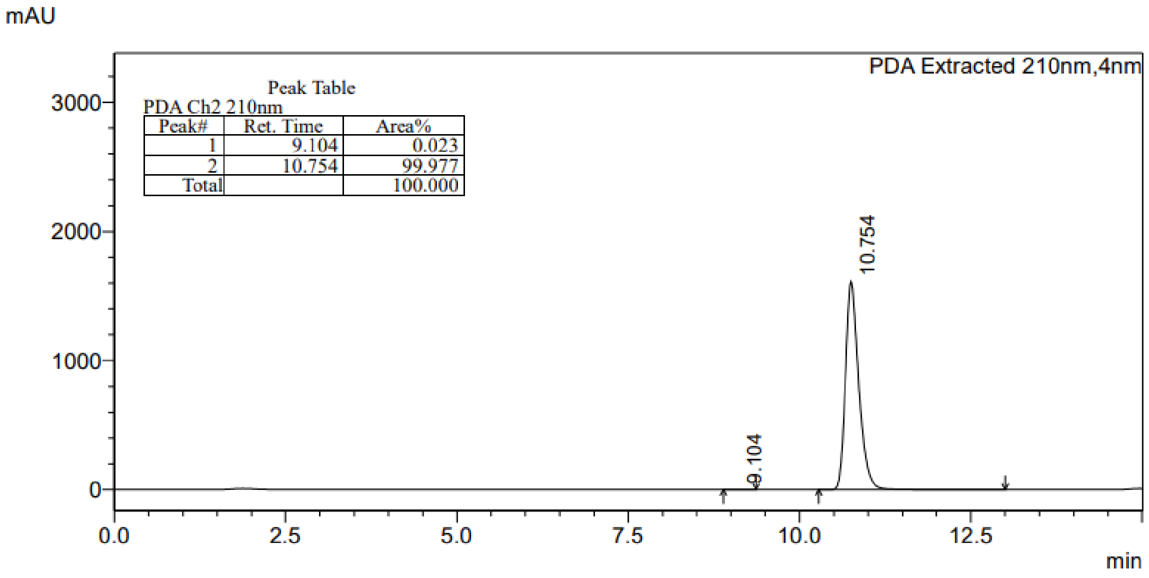

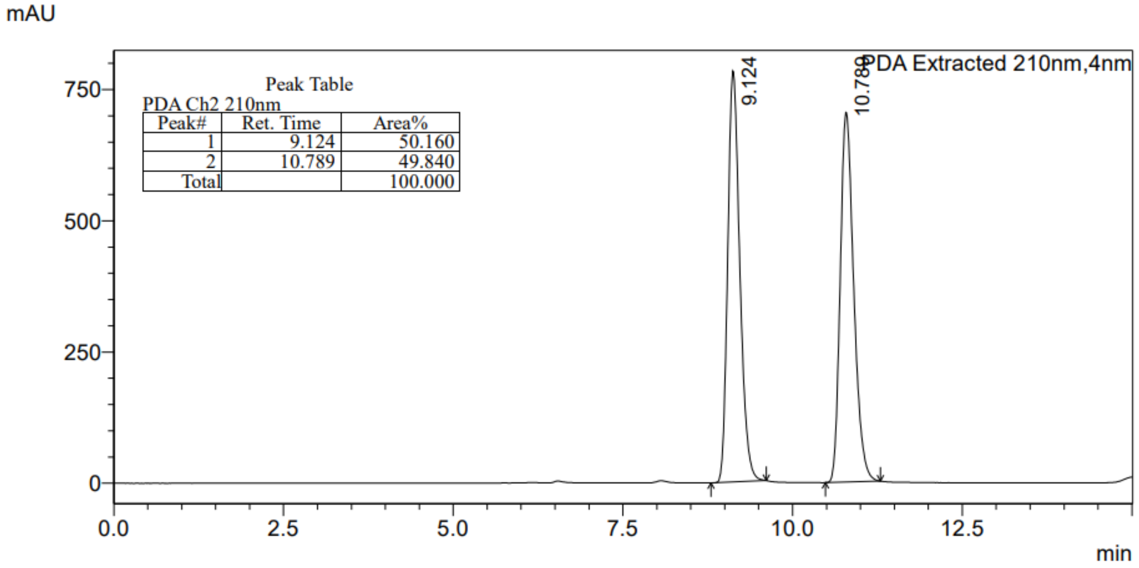

Supplementary Fig. 9 | HPLC traces of **52** (top) and corresponding racemic mixture (bottom).

(S)-*N*-{1-(5-Bromo-6-methoxynaphthalen-2-yl)ethyl}acetamide (53)

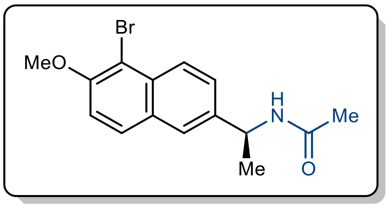

Isolated with DCM/Acetone = 85:15.; Colorless solid (54.1 mg, 84%); **m.p.** = 183 – 185 °C; **^{1}H NMR** (500 MHz, $CDCl_3$) δ 8.17 (d, *J* = 8.8 Hz, 1H), 7.76 (d, *J* = 9.0 Hz, 1H), 7.69 – 7.66 (m, 1H), 7.50 (dd, *J* = 8.8, 1.8 Hz, 1H), 7.25 (d, *J* = 9.0 Hz, 1H), 5.90 (d, *J* = 7.8 Hz, 1H), 5.27 (p, *J* = 7.1 Hz, 1H), 4.01 (s, 3H), 1.99 (s, 3H), 1.55 (d, *J* = 6.9 Hz, 3H); **^{13}C NMR** (125 MHz, $CDCl_3$) δ 169.3, 153.9, 139.2, 132.6, 129.8, 129.0, 127.0, 126.6, 125.0, 114.1, 108.6, 57.2, 48.6, 23.6, 21.7; **IR** (cm^{-1}) 3283, 1636, 1601, 1539, 1275, 1068, 820, 802, 603; **HRMS** (EI) m/z calcd. For $C_{15}H_{16}BrNO_2$ $[M]^+$: 321.0364, found: 321.0367; **Specific Rotation** $[\alpha]_D^{29}$ = –115.8° (*c* 0.47, CH_2Cl_2); **HPLC Analysis**. CHIRALPAK AD-H, 32 °C; n-hexane:*i*-PrOH = 80:20, 0.5 mL/min, 230 nm, t_{R1} (major) = 11.2 min, t_{R2} (minor) = 9.5 min, 98:2 e.r.

The structure of the title compound was further confirmed by X-ray crystallographic analysis (see ***Appendix II****)*

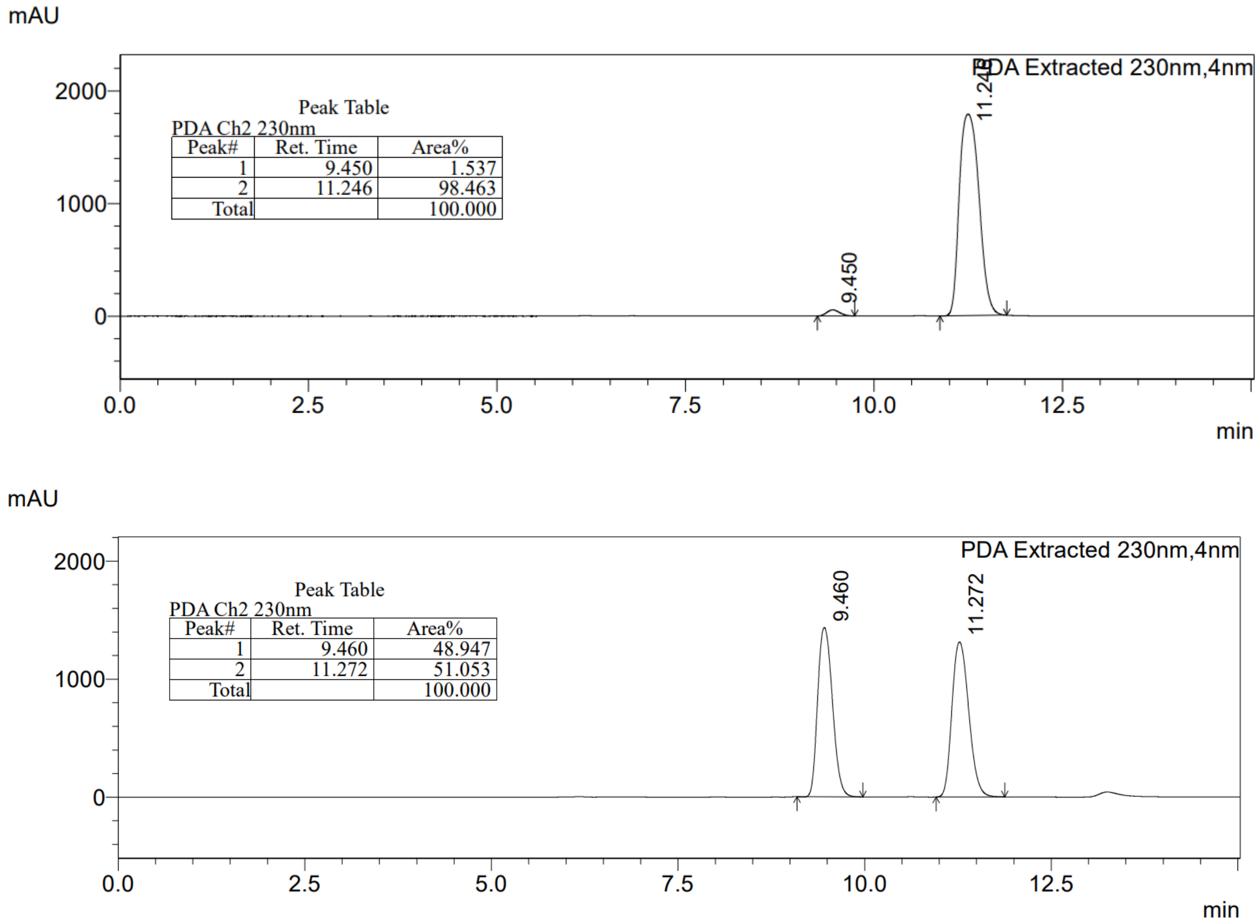

Supplementary Fig. 10 | HPLC traces of **53** (top) and corresponding racemic mixture (bottom).

(*S*)-*N*-{1-(4-Isobutylphenyl)ethyl}acetamide (55)

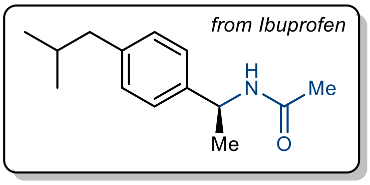

Isolated with DCM/Acetone = 85:15.; Colorless liquid (36.5 mg, 83%); **^{1}H NMR** (600 MHz, $CDCl_3$) δ 7.22 (dd, *J* = 6.1, 1.7 Hz, 2H), 7.11 (dd, *J* = 6.3, 1.8 Hz, 2H), 5.74 (d, *J* = 7.8 Hz, 1H), 5.10 (p, *J* = 7.1 Hz, 1H), 2.45 (d, *J* = 7.2 Hz, 2H), 1.97 (s, 3H), 1.89 – 1.80 (m, 1H), 1.47 (d, *J* = 6.9 Hz, 3H), 0.89 (d, *J* = 6.6 Hz, 6H); **^{13}C NMR** (150 MHz, $CDCl_3$) δ 169.1, 141.0, 140.5, 129.5, 126.1, 48.6, 45.2, 30.3, 23.6, 22.5, 21.7; **IR** (cm^{-1}) 3273, 2954, 1639, 1544, 1450, 1370; **HRMS** (EI) m/z calcd. For $C_{14}H_{21}NO$ $[M]^+$: 219.1623, found: 219.1620; **Specific Rotation** $[\alpha]_D^{29}$ = –101.9° (*c* 0.22, CH_2Cl_2); **HPLC Analysis**. CHIRALPAK AD-H, 28 °C; n-hexane:*i*-PrOH = 90:10, 0.5 mL/min, 210 nm, t_{R1} (minor) = 9.0 min, t_{R2} (major) = 10.3 min, >99:1 e.r.

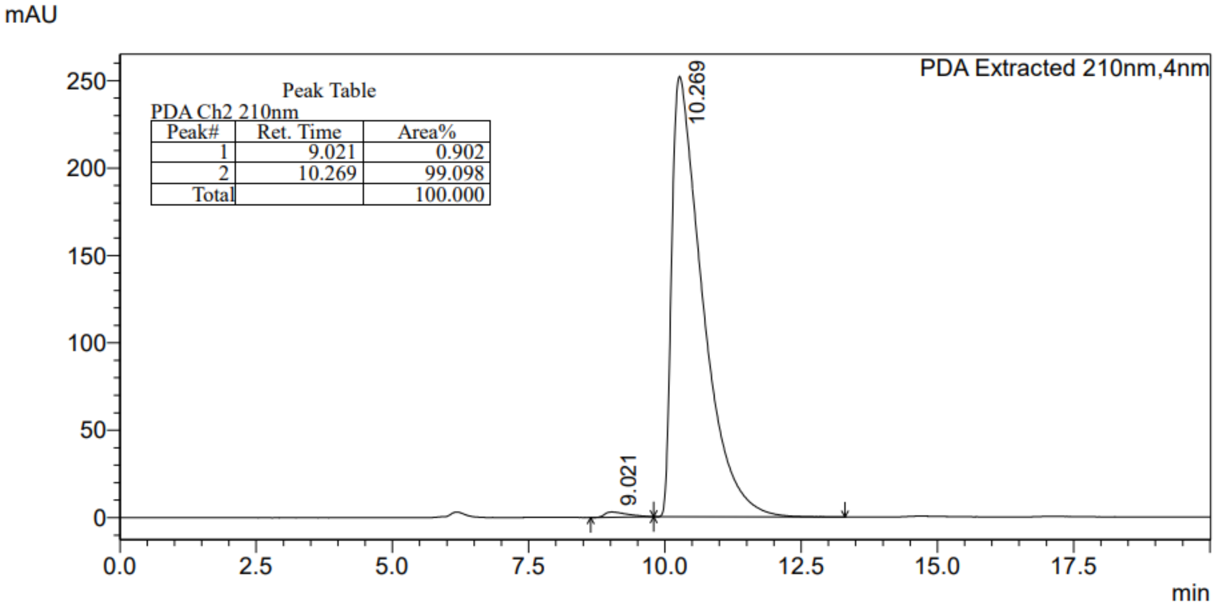

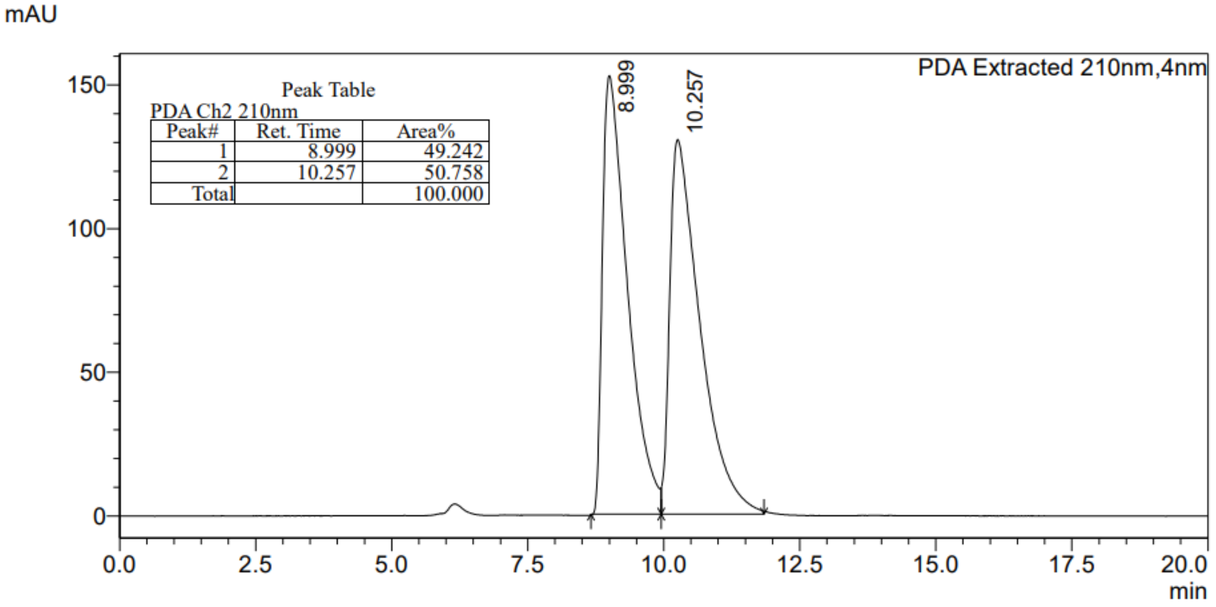

Supplementary Fig. 11 | HPLC traces of **55** (top) and corresponding racemic mixture (bottom).

tert-Butyl [(2S,4S)-1-{(1,1'-biphenyl)-4-yl}-4-acetamidopentan-2-yl]carbamate (57)

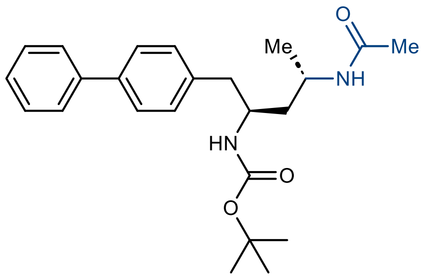

Isolated with DCM/Acetone = 60:40; Colorless solid (66.1 mg, 83%); **m.p.** = 170 – 172 °C; **¹H NMR** (600 MHz, $CDCl_3$) δ 7.59 – 7.55 (m, 2H), 7.53 (d, *J* = 7.9 Hz, 2H), 7.43 (t, *J* = 7.6 Hz, 2H), 7.33 (t, *J* = 7.4 Hz, 1H), 7.28 – 7.24 (m, 2H), 5.60 (s, 1H), 4.62 (d, *J* = 8.9 Hz, 1H), 4.09 (p, *J* = 6.9 Hz, 1H), 3.86 (s, 1H), 2.94 – 2.76 (m, 2H), 1.93 (s, 3H), 1.70 – 1.58 (m, 2H), 1.39 (s, 9H), 1.16 (d, *J* = 6.7 Hz, 3H); **¹³C NMR** (150 MHz, $CDCl_3$) δ 169.7, 155.6, 141.0, 139.5, 137.3, 130.0, 128.9, 127.3, 127.3, 127.1, 79.4, 49.0, 42.5, 41.2, 40.1, 28.5, 23.7, 20.6; **IR** (cm^{-1}) 3336, 2975, 1678, 1647, 1518, 1166, 755, 691, 593; **HRMS** (FAB) m/z calcd. For $C_{24}H_{33}N_2O_3$ $[M+H]^+$: 397.2491, found: 397.2516; **Specific Rotation** $[\alpha]_D^{29}$ = 27.2° (*c* 0.45, CH_2Cl_2).

The structure of the title compound was further confirmed by X-ray crystallographic analysis (see ***Appendix II****)*

N-[(R)-1-{(1R,4R,4aS,8aR)-4,7-Dimethyl-1,2,3,4,4a,5,6,8a-octahydronaphthalen-1-yl}ethyl]acetamide (59)

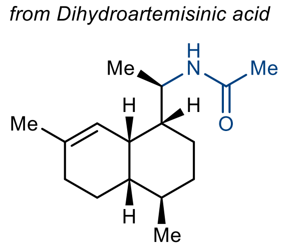

Isolated with DCM/Acetone = 80:20; Colorless solid (36.8 mg, 74%); **m.p.** = 166 – 168 °C; **¹H NMR** (400 MHz, $CDCl_3$) δ 5.29 – 5.16 (m, 2H), 4.02 (tq, *J* = 9.5, 6.5 Hz, 1H), 2.51 – 2.45 (m, 1H), 1.98 (s, 3H), 1.96 – 1.84 (m, 1H), 1.84 – 1.74 (m, 1H), 1.68 – 1.36 (m, 8H), 1.23 – 1.01 (m, 6H), 0.90 – 0.77 (m, 4H); **¹³C NMR** (100 MHz, $CDCl_3$) δ 169.6, 136.0, 119.8, 48.1, 47.4, 42.0, 37.8, 35.5, 27.8, 26.7, 25.8, 25.7, 23.8(4), 23.7(6), 19.9, 19.4; **IR** (cm^{-1}) 2905, 1730, 1550, 1434, 1370, 732, 608, 470, 432; **HRMS** (EI) m/z calcd. For $C_{16}H_{27}NO$ $[M]^+$: 249.2093, found: 249.2094; **Specific Rotation** $[\alpha]_D^{29}$ = –11.2° (*c* 0.61, CH_2Cl_2).

The structure of the title compound was further confirmed by X-ray crystallographic analysis (see ***Appendix II****)*

VIII. Dioxazolone Scope of Decaroboxylative Amidation of (*S*)-Naproxen

Preparation of 3-alkyl-1,4,2-dioxazol-5-ones

3-(3-Phenylpropyl)-1,4,2-dioxazol-5-one,[22] (*E*)-3-(4-phenylbut-3-en-1-yl)-1,4,2-dioxazol-5-one,[23] methyl 4-{4-(5-oxo-1,4,2-dioxazol-3-yl)but-1-yn-1-yl}benzoate,[24] (*E*)-3-styryl-1,4,2-dioxazol-5-one,[25] and 3-{2-(4,5-diphenyloxazol-2-yl)ethyl}-1,4,2-dioxazol-5-one[26] were prepared based on the reported procedures.

Additional optimization of transition-metal-free decarboxylative amidation for dioxazolone scope

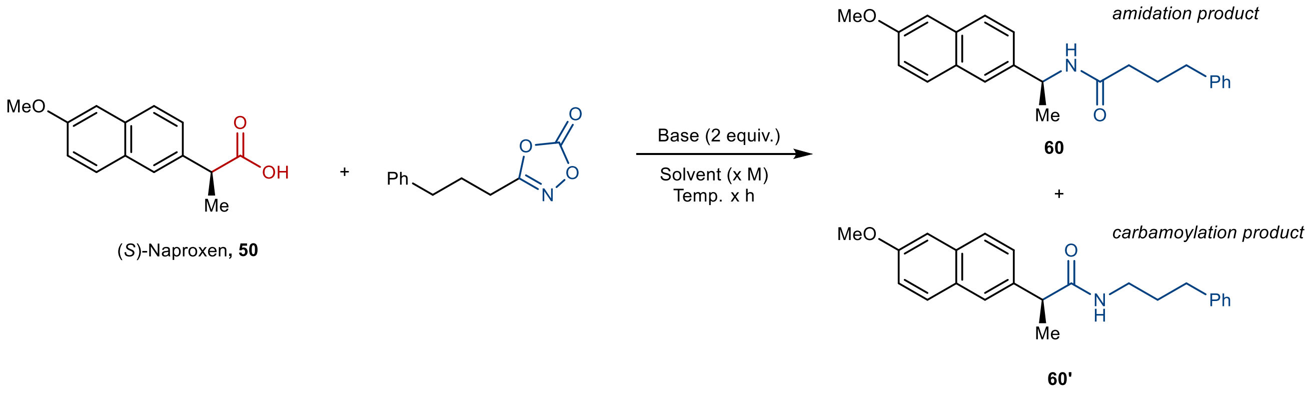

To an oven-dried 4 mL screw-capped vial equipped with oval-shaped stirring bar were added (*S*)-Naproxen (**50**, 0.100 mmol, 23.0 mg), bases (as indicated, 2 equiv.), and solvent (0.500 mL, 0.200 M) under atmospheric conditions. To the reaction mixture was added 3-(3-phenylpropyl)-1,4,2-dioxazol-5-one (0.200 mmol, 20.5 mg) and stirred at indicated temperature for indicated time. Product yield was measured by ^{1}H-NMR analysis of the crude mixture in the presence of internal standard (1,3,5-trimethoxybenzene) in DMSO-d_6. While standard reaction conditions (DMSO, r.t., 4 h) provided mixture of corresponding amidated product **60** (44%) and carbamoylated product **60′** (11%, **60** : **60′** = 4:1, entry 1), different types of super-bases, such as guanidine and phosphazene, were not effective to enhance the selectivity toward desired amidation reactions (entries 2 and 3). Eventually, when the reaction was conducted at lower temperature (= 0 °C) in *N*,*N*-dimethylformamide (DMF, melting point = –61 °C) rather than dimethylsulfoxide (DMSO, melting point = 19 °C), satisfiable selectivity was obtained with high yield of **60** (70%) and suppressed carbamoylated product formation (4%, **60** : **60′** = 18:1)

Supplementary Table 4 | Additional reaction optimization using 3-(3-phenylpropyl)-1,4,2-dioxazol-5-one and (*S*)-Naproxen

Entry	Base	Solvent	Temperature	Time (h)	**60** (%)	**60′** (%)	**60:60′**
1	DBU	DMSO	r.t. (= 25 °C)	4 h	44	11	4:1
2	Guanidine	DMSO	r.t. (= 25 °C)	4 h	9	20	1:2.2
3	Phosphazene	DMSO	r.t. (= 25 °C)	4 h	29	28	1:1
4	DBU	DMF	0 °C	12 h	70	4	18:1

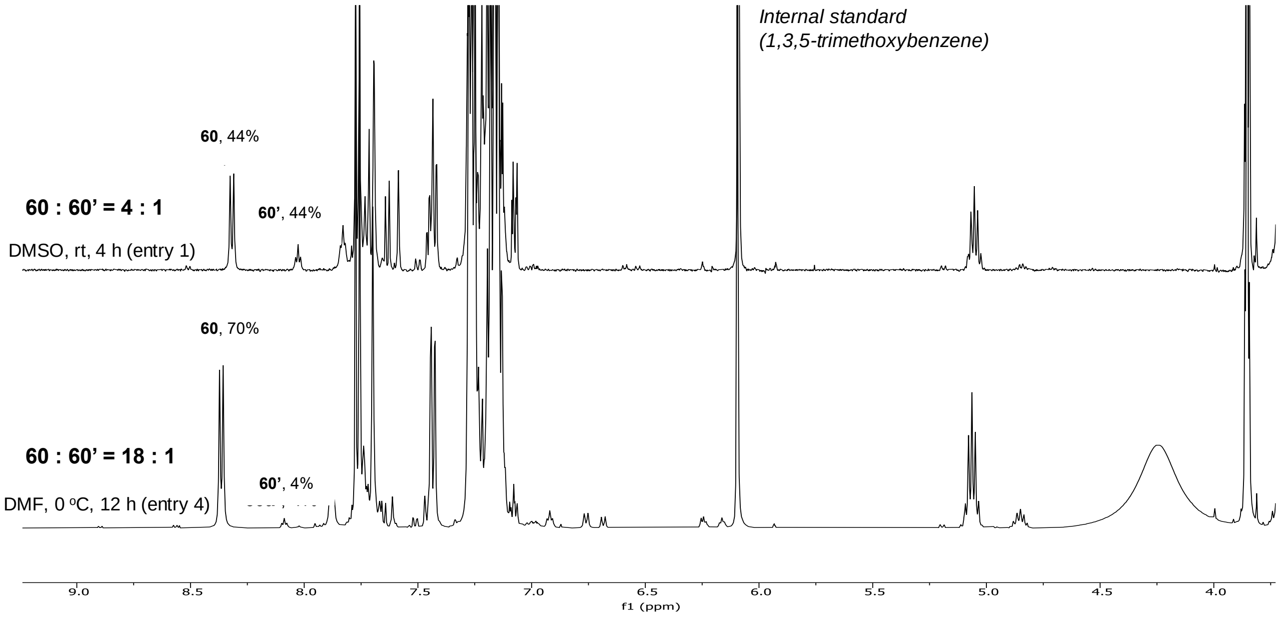

Supplementary Fig. 12 | ^{1}H NMR spectrum obtained from crude mixture (**Table S4**, top = entry 1, bottom = entry 4)

Dioxazolone substrate scope investigation of transition-metal-free decarboxylative amidation

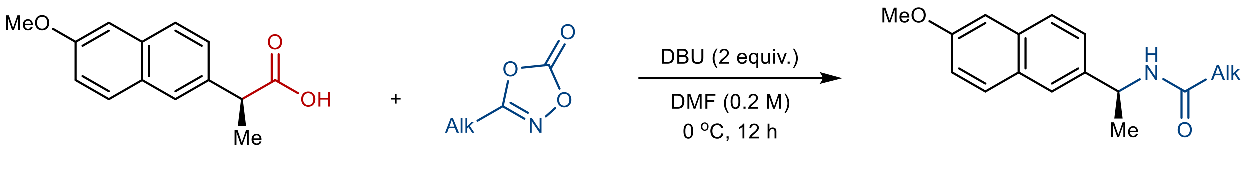

To an oven-dried 4 mL screw-capped vial equipped with oval-shaped stirring bar were added (S)-Naproxen (**50**, 0.200 mmol, 46.1 mg), 1,8-diazabicyclo[5.4.0]undec-7-ene (DBU, 2.00 equiv., 0.400 mmol, 60.9 mg), and anhydrous *N*,*N*-dimethylformamide (DMF, 1.00 mL, 0.200 M) under atmospheric conditions. To the reaction mixture was added 3-alkyl-1,4,2-dioxazol-5-ones (2.00 equiv., 0.400 mmol) and stirred at 0 °C for 12 h. After reaction completion, the crude reaction mixture was diluted with dichloromethane (DCM, 5.0 mL), added 1N HCl aqueous solution (10 mL), and extracted with DCM (5.0 mL x 3 times). The combined organic layer was dried over $MgSO_4$, filtered, and concentrated under the reduced pressure. The crude mixture was subjected to silica column chromatography to provide the purified desired N-alkylamide products (eluent: Dichloromethane/Acetonitrile, 95:5 ~ 90:10). Values of enantiomeric ratio (e.r.) were determined by HPLC analysis. The authentic samples of racemic products were synthesized by our present decarboxylative amination method using as (±)-Naproxen. As a note, when the reaction was conducted with phenyl dioxazolone and (*S*)-Naproxen, the corresponding amide product was formed in trace amounts (<5%).

(*S*)-*N*-{1-(6-Methoxynaphthalen-2-yl)ethyl}-4-phenylbutanamide (60)

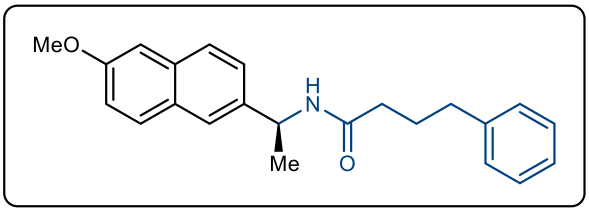

Isolated with DCM/Acetonitrile = 90:10.; Colorless solid (50.4 mg, 73%); **m.p.** = 109 – 111 °C; **^{1}H NMR** (500 MHz, $CDCl_3$) δ 7.77 – 7.67 (m, 3H), 7.42 (dd, *J* = 8.5, 1.9 Hz, 1H), 7.29 (dd, *J* = 8.2, 6.8 Hz, 2H), 7.25 – 7.12 (m, 5H), 5.83 (d, *J* = 8.1 Hz, 1H), 5.31 (p, *J* = 7.0 Hz, 1H), 3.94 (s, 3H), 2.66 (dd, *J* = 8.4, 6.7 Hz, 2H), 2.21 (td, *J* = 7.3, 2.7 Hz, 2H), 2.01 (p, *J* = 7.5 Hz, 2H), 1.58 (d, *J* = 6.9 Hz, 3H); **^{13}C NMR** (125 MHz, $CDCl_3$) δ 171.8, 157.8, 141.6, 138.4, 134.0, 129.5, 128.9, 128.6, 128.5, 127.4, 126.1, 125.4, 124.5, 119.2, 105.7, 55.4, 48.6, 36.1, 35.2, 27.2, 21.6. **IR** (cm^{-1}) 3300, 1638, 1534, 1261, 1029, 888, 697, 675, 473; **HRMS** (EI) m/z calcd. For $C_{23}H_{25}NO_2$ $[M]^+$: 347.1885, found: 347.1883; **Specific Rotation** $[\alpha]_D^{29}$ = –78.4° (*c* 0.63, CH_2Cl_2); **HPLC Analysis**. CHIRALPAK AD-H, 32 °C; *n*-hexane:*i*-PrOH = 90:10, 0.5 mL/min, 232 nm, t_{R1} (major) = 32.2 min, t_{R2} (minor) = 23.0 min, >99:1 e.r.

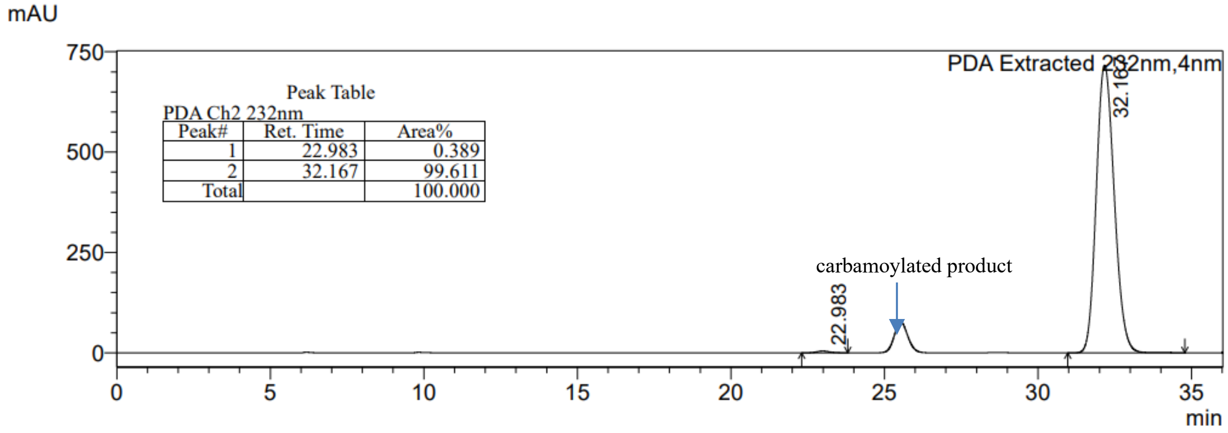

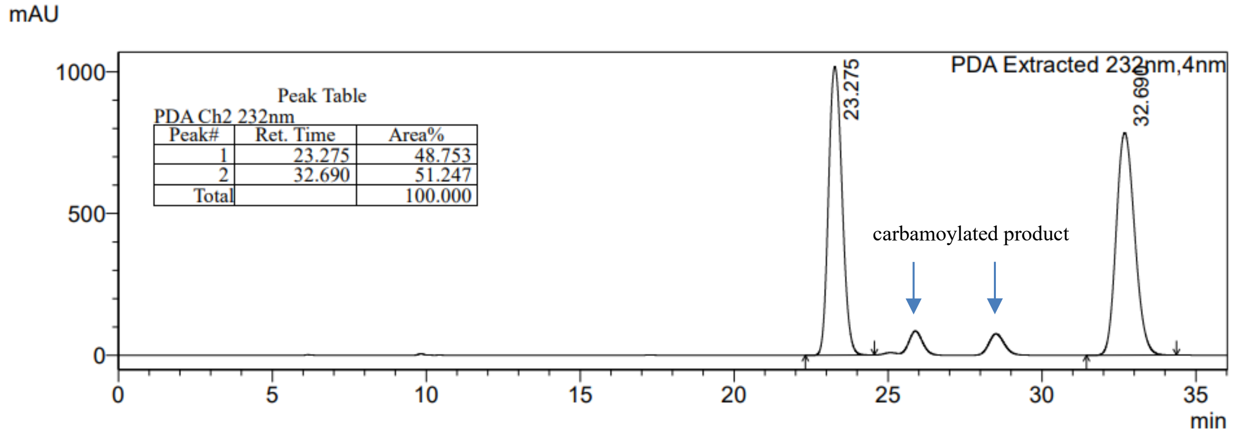

Supplementary Fig. 13 | HPLC traces of **60** (top) and corresponding racemic mixture (bottom).

(*S*,*E*)-*N*-{1-(6-Methoxynaphthalen-2-yl)ethyl}-5-phenylpent-4-enamide (61)

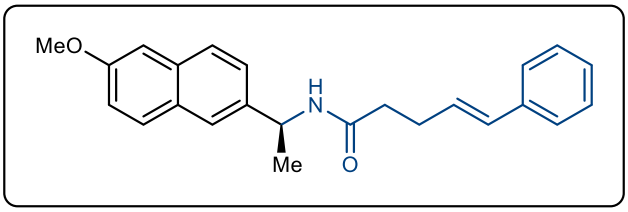

Isolated with DCM/Acetonitrile = 90:10.; Colorless solid (60.4 mg, 84%); **m.p.** = 144 – 146 °C; **^{1}H NMR** (400 MHz, $CDCl_3$) δ 7.67 – 7.58 (m, 3H), 7.32 – 7.24 (m, 4H), 7.23 – 7.17 (m, 2H), 7.15 – 7.04 (m, 2H), 6.42 (dt, *J* = 15.8, 1.5 Hz, 1H), 6.19 (dt, *J* = 15.8, 6.9 Hz, 1H), 5.78 (d, *J* = 8.1 Hz, 1H), 5.29 (dt, *J* = 14.0, 7.0 Hz, 1H), 3.91 (s, 3H), 2.62 – 2.52 (m, 2H), 2.44 – 2.28 (m, 2H), 1.56 (d, *J* = 6.9 Hz, 3H); **^{13}C NMR** (100 MHz, $CDCl_3$) δ 171.3, 157.8, 138.3, 137.4, 134.0, 131.3, 129.5, 128.9, 128.8, 128.7, 127.5, 127.3, 126.2, 125.5, 124.5, 119.2, 105.7, 55.5, 48.7, 36.7, 29.2, 21.7; **IR** (cm^{-1}) 3303, 2926, 1635, 1607, 1504, 1262, 1178, 1029, 962, 855, 812, 689; **HRMS** (EI) m/z calcd. For $C_{24}H_{25}NO_2$ $[M]^+$: 359.1885, found: 359.1882; **Specific Rotation** $[\alpha]_D^{29}$ = –42.6° (*c* 0.29, CH_2Cl_2); **HPLC Analysis**. CHIRALPAK AD-H, 32 °C; *n*-hexane:*i*-PrOH = 80:20, 0.5 mL/min, 250 nm, t_{R1} (major) = 15.2 min, t_{R2} (minor) = 12.2 min, 98:2 e.r.

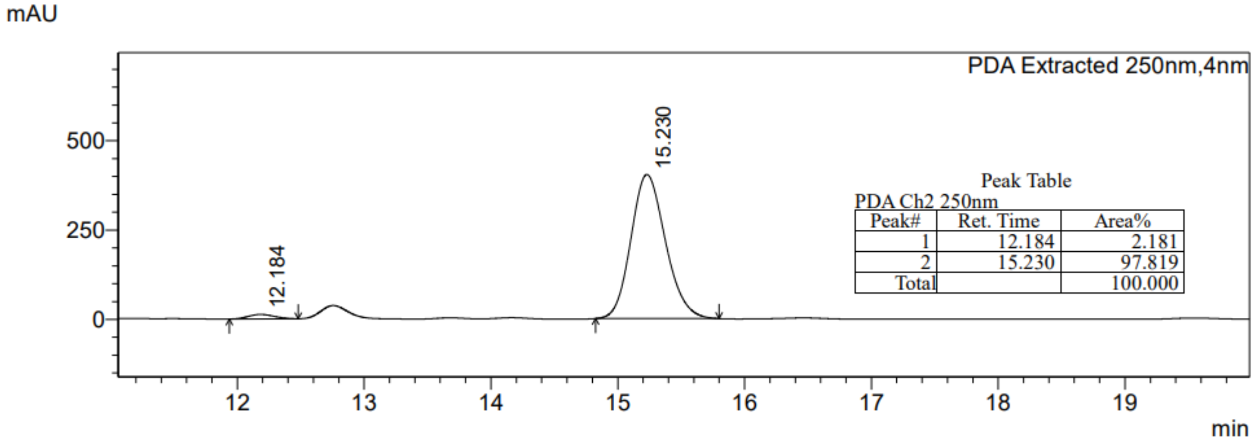

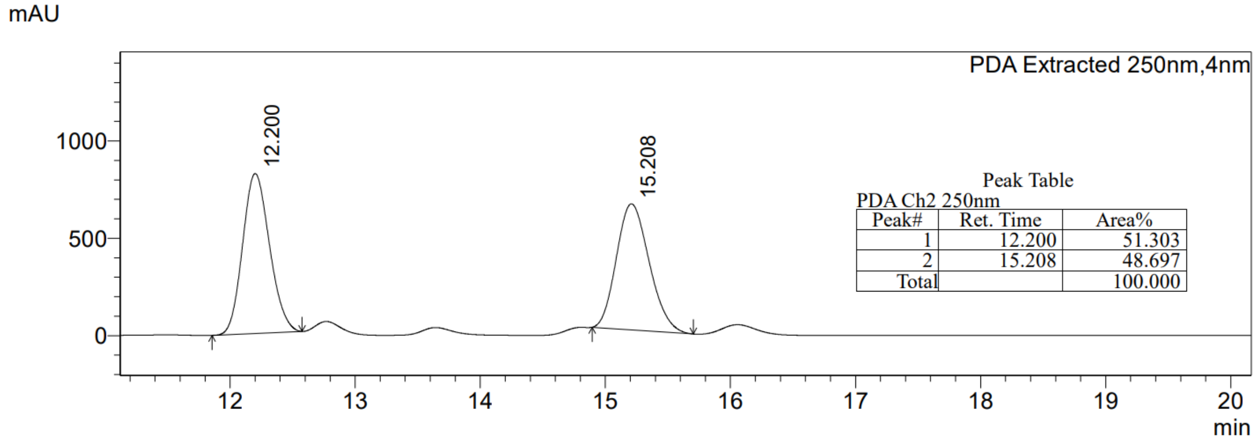

Supplementary Fig. 14 | HPLC traces of **61** (top) and corresponding racemic mixture (bottom).

Methyl (*S*)-4-(5-[{1-(6-methoxynaphthalen-2-yl)ethyl}amino]-5-oxopent-1-yn-1-yl)benzoate (62)

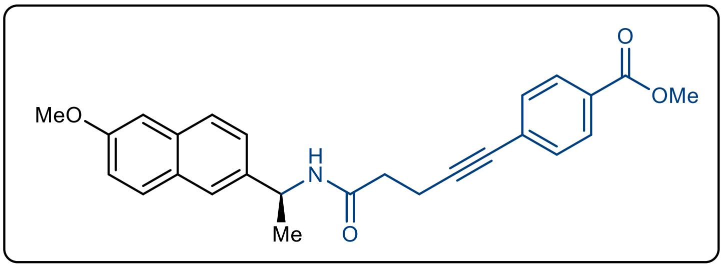

Isolated with DCM/Acetonitrile = 95:5.; Colorless solid (67.4 mg, 81%); **m.p.** = 146 – 1148 °C; **^{1}H NMR** (600 MHz, $CDCl_3$) δ 7.84 – 7.79 (m, 2H), 7.69 – 7.66 (m, 1H), 7.62 (d, J = 8.7 Hz, 2H), 7.39 (dd, J = 8.5, 1.9 Hz, 1H), 7.24 (d, J = 1.7 Hz, 1H), 7.23 (d, J = 1.7 Hz, 1H), 7.11 (dd, J = 8.9, 2.6 Hz, 1H), 7.07 (d, J = 2.6 Hz, 1H), 6.01 (d, J = 8.0 Hz, 1H), 5.35 – 5.27 (m, 1H), 3.92 (s, 3H), 3.91 (s, 3H), 2.84 – 2.73 (m, 2H), 2.58 – 2.42 (m, 2H), 1.58 (d, J = 6.9 Hz, 3H); **^{13}C NMR** (150 MHz, $CDCl_3$) δ 170.2, 166.7, 157.9, 138.1, 134.0, 131.5, 129.5, 129.3, 128.9, 128.2, 127.5, 125.4, 124.6, 119.2, 105.7, 91.8, 81.3, 55.4, 52.3, 49.0, 35.8, 21.7, 16.3; **IR** (cm^{-1}) 3182, 1715, 1664, 1435, 1276, 1110, 829, 768; **HRMS** (EI) m/z calcd. For $C_{26}H_{25}NO_4$ $[M]^+$: 415.1784, found: 415.1780; **Specific Rotation** $[\alpha]_D^{29}$ = –1.4° (c 0.66, CH_2Cl_2); **HPLC Analysis**. CHIRALPAK AD-H, 32 °C; n-hexane:i-PrOH = 80:20, 0.5 mL/min, 232 nm, t_{R1} (major) = 17.8 min, t_{R2} (minor) = 14.5 min, 94:6 e.r.

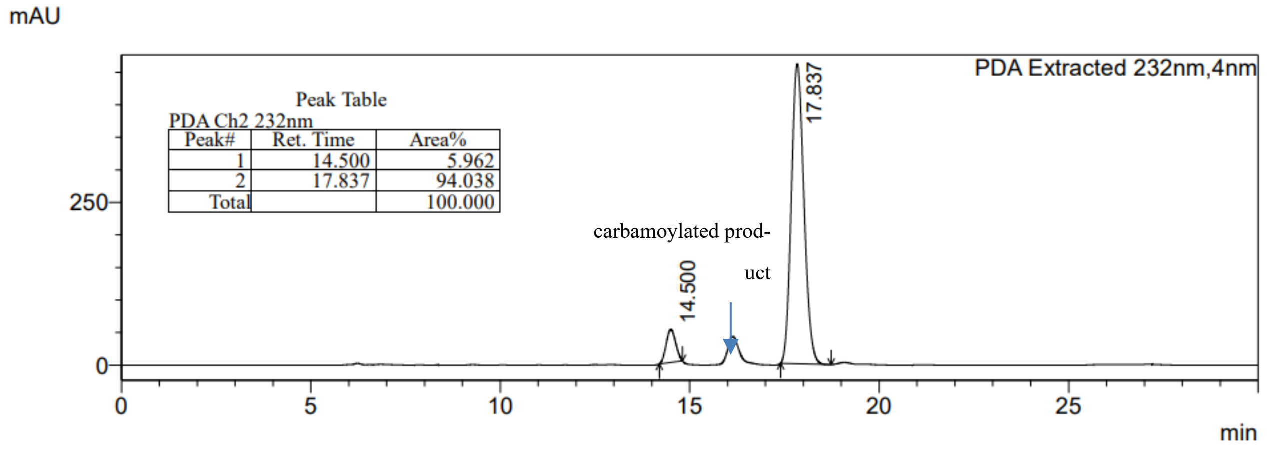

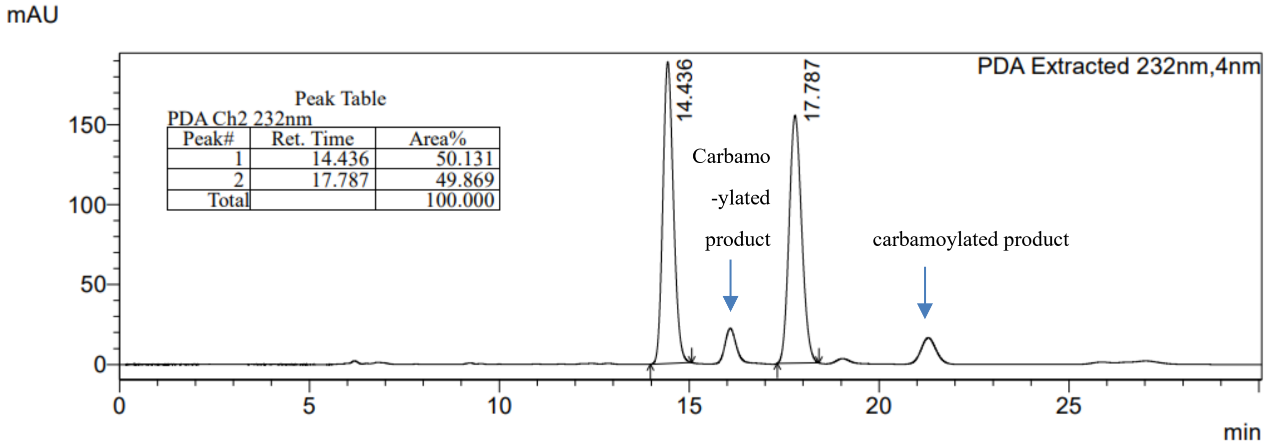

Supplementary Fig. 15 | HPLC traces of **62** (top) and corresponding racemic mixture (bottom).

(*S*)-*N*-{1-(6-methoxynaphthalen-2-yl)ethyl}cinnamamide (63)

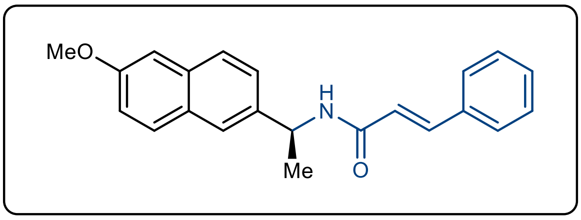

Isolated with DCM/Acetonitrile = 90:10.; Colorless solid (41.7 mg, 63%); **m.p.** = 176 – 178 °C; **^{1}H NMR** (500 MHz, $CDCl_3$) δ 7.73 – 7.66 (m, 3H), 7.64 (d, *J* = 15.6 Hz, 1H), 7.44 (td, *J* = 8.2, 7.7, 2.3 Hz, 3H), 7.37 – 7.28 (m, 3H), 7.13 (dd, *J* = 8.9, 2.6 Hz, 1H), 7.10 (d, *J* = 2.5 Hz, 1H), 6.43 (d, *J* = 15.6 Hz, 1H), 6.19 (d, *J* = 8.1 Hz, 1H), 5.40 (p, *J* = 7.1 Hz, 1H), 3.90 (s, 3H), 1.62 (d, *J* = 7.0 Hz, 3H); **^{13}C NMR** (125 MHz, $CDCl_3$, *one carbon peak overlaps with others*) δ 165.2, 157.8, 141.3, 138.3, 135.0, 134.0, 129.7, 129.5, 128.9, 127.9, 127.5, 125.5, 124.6, 120.9, 119.1, 105.7, 55.4, 49.0, 21.6; **IR** (cm^{-1}) 3299, 2974, 1650, 1533, 1448, 1163, 851, 672, 492; **HRMS** (EI) m/z calcd. For $C_{22}H_{21}NO_2$ $[M]^+$: 331.1572, found: 331.1576; **Specific Rotation** $[\alpha]_D^{29}$ = –3.8° (*c* 0.55, CH_2Cl_2); **HPLC Analysis**. CHIRALPAK AD-H, 32 °C; *n*-hexane:*i*-PrOH = 80:20, 0.5 mL/min, 280 nm, t_{R1} (major) = 36.3 min, t_{R2} (minor) = 16.7 min, 96:4 e.r.

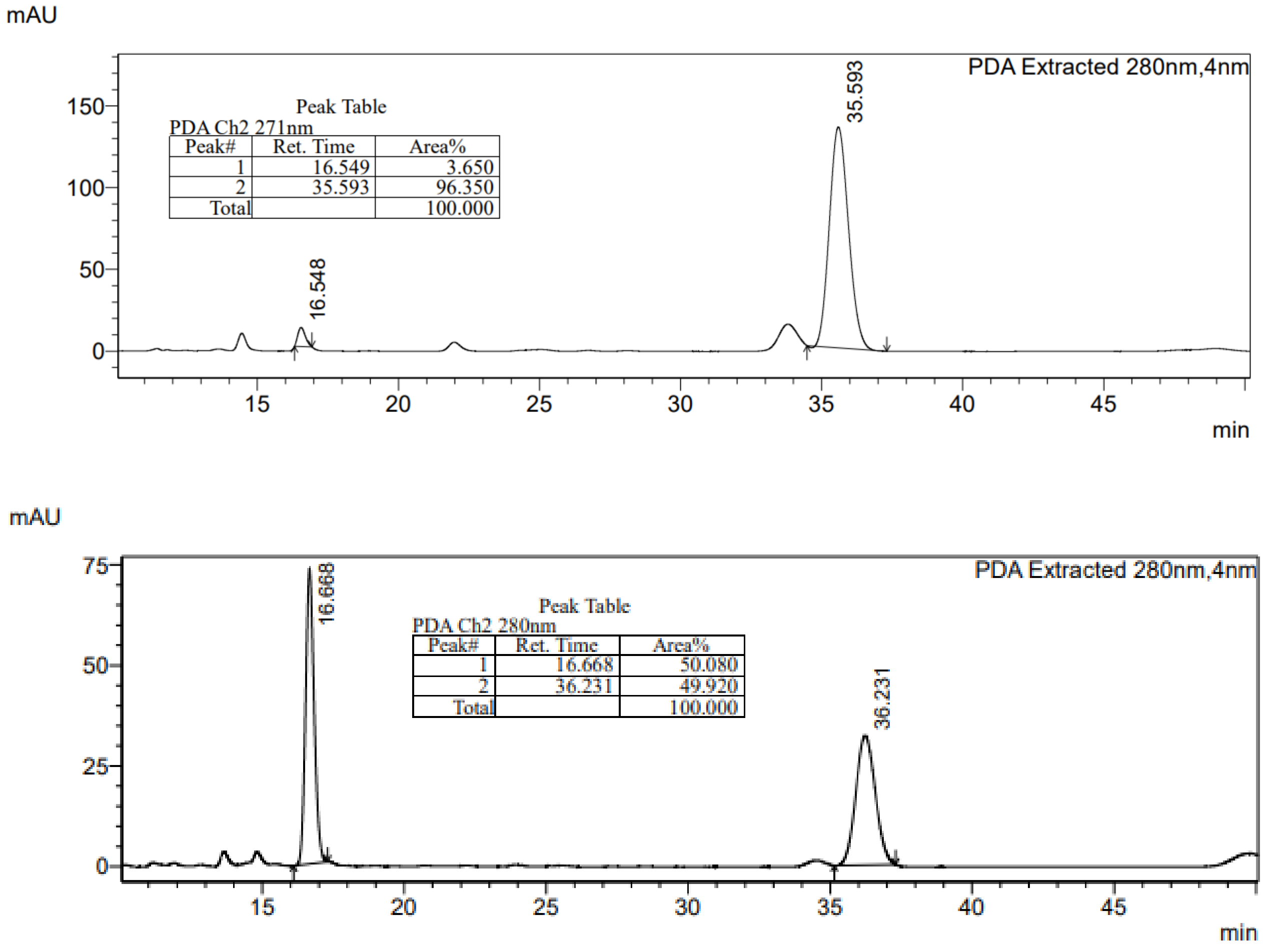

Supplementary Fig. 16 | HPLC traces of **63** (top) and corresponding racemic mixture (bottom).

(*S*)-3-(4,5-Diphenyloxazol-2-yl)-*N*-{1-(6-methoxynaphthalen-2-yl)ethyl}propanamide (64)

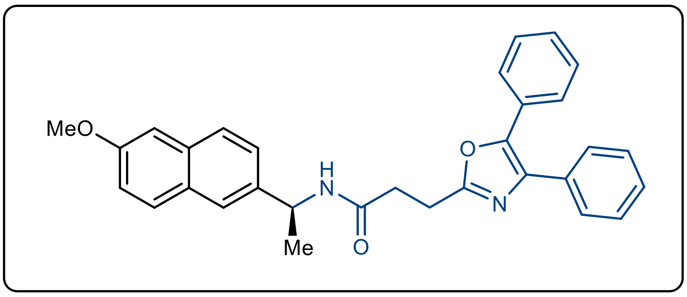

Isolated with DCM/Acetonitrile = 90:10.; Colorless solid (51.4 mg, 54%); **m.p.** = 122 – 124 °C; **^{1}H NMR** (600 MHz, $CDCl_3$) δ 7.63 (s, 1H), 7.59 (t, J = 8.2 Hz, 2H), 7.54 (dd, J = 7.5, 2.1 Hz, 4H), 7.37 – 7.26 (m, 7H), 7.10 (dd, J = 8.9, 2.5 Hz, 1H), 7.05 (d, J = 2.5 Hz, 1H), 6.55 (d, J = 8.0 Hz, 1H), 5.32 – 5.23 (m, 1H), 3.90 (s, 3H), 3.21 (t, J = 7.0 Hz, 2H), 2.85 – 2.75 (m, 2H), 1.54 (d, J = 6.9 Hz, 3H); **^{13}C NMR** (150 MHz, $CDCl_3$) δ 170.6, 162.6, 157.8, 145.7, 138.4, 135.0, 134.0, 132.4, 129.5, 129.0, 128.9, 128.8, 128.6(9), 128.6(6), 128.2, 128.0, 127.5, 126.6, 125.4, 124.5, 119.1, 105.8, 55.4, 49.0, 33.4, 24.3, 21.9; **IR** (cm^{-1}) 3360, 2964, 1641, 1529, 1195, 969, 858, 675, 483; **HRMS** (EI) m/z calcd. For $C_{31}H_{28}N_2O_3$ $[M]^+$: 476.2100, found: 476.2102; **Specific Rotation** $[\alpha]_D^{29}$ = –31.1° (c 0.46, CH_2Cl_2); **HPLC Analysis**. CHIRALPAK AD-H, 32 °C; n-hexane:i-PrOH = 80:20, 0.5 mL/min, 230 nm, t_{R1} (major) = 13.9 min, t_{R2} (minor) = 11.1 min, 99:1 e.r.

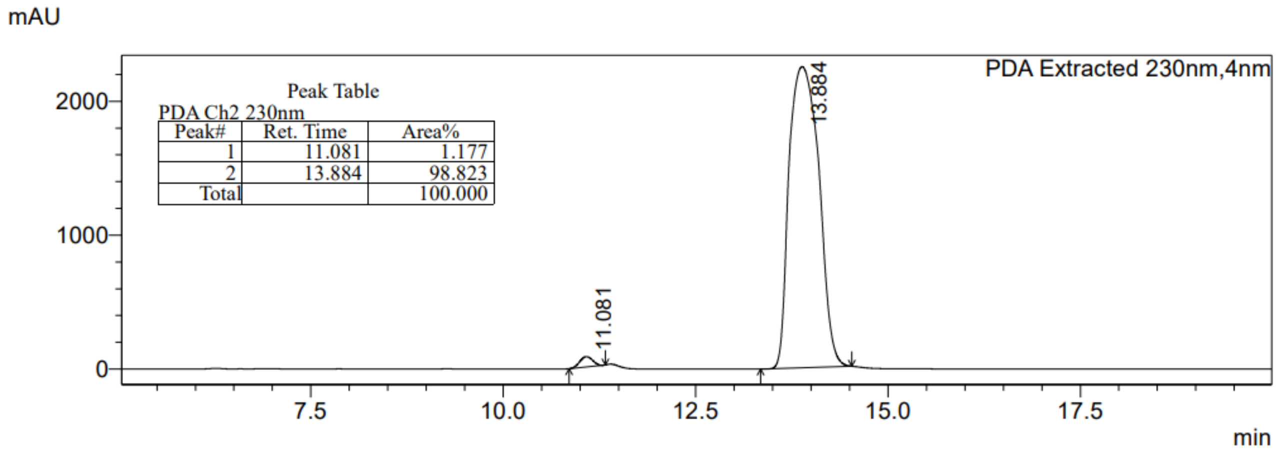

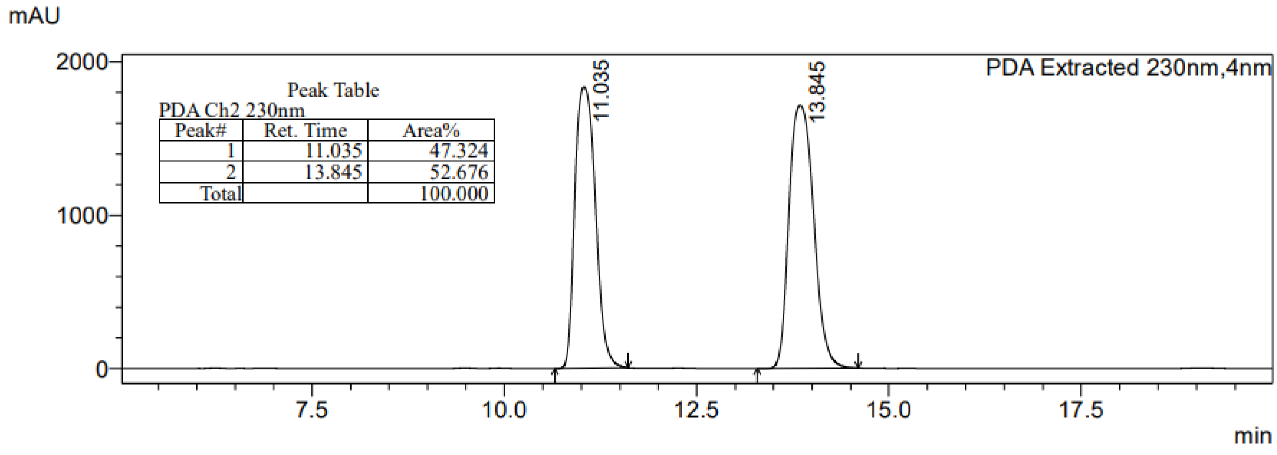

Supplementary Fig. 17 | HPLC traces of **64** (top) and corresponding racemic mixture (bottom).

IX. Synthetic Applicability

1. Multi-gram scale reaction

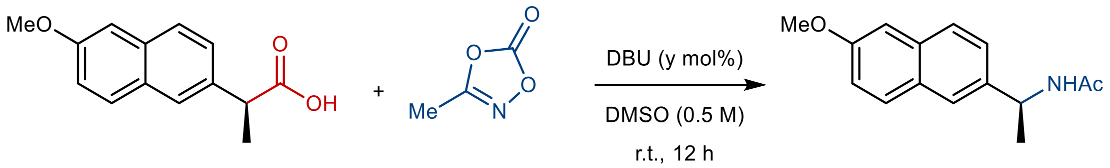

50, 10 mmol (2.3 g) **2e**, x equiv **52**

To an oven-dried 250 mL round bottom flask equipped with oval-shaped stirring bar were added (*S*)-Naproxen (**50**, 10.0 mmol, 2.30 g), 1,8-Diazabicyclo 5.4.0 undec-7-ene (DBU, 2 equiv., 20.0 mmol, 3.04 g), and anhydrous dimethylsulfoxide (DMSO, 20.0 mL, 0.500 M) under atmospheric conditions. To the reaction mixture was added 3-methyl-1,4,2-dioxazol-5-ones (20.0 mmol, 2.02 g) and stirred at for 12 h. After reaction completion, the crude reaction mixture was diluted with dichloromethane (DCM, 50 mL), added 1N HCl aqueous solution (50 mL), and extracted with DCM (50 mL x 3 times). The combined organic layer was dried over $MgSO_4$, filtered, and concentrated under the reduced pressure. The crude mixture was subjected to silica column chromatography to provide the purified desired N-alkylamide products (eluent: Dichloromethane/Acetone, 80:20). Values of enantiomeric ratio (e.r.) were determined by HPLC analysis. The authentic samples of racemic products were synthesized by our present decarboxylative amination method using as (±)-Naproxen.

Supplementary Table 5 | Results of gram-scale reaction of (*S*)-Naproxen **50** and methyl dioxazolone **2e**

Entry	x (equiv.)	y (mol%)	yield (%)	e.r.
1	2.0	200	92	>99:1
2	2.0	25	64	>99:1
3	1.1	25	53	>99:1

2. Access to ^{15}N-labeled alkylamine via transition-metal free decarboxylative amidation

Synthesis of 3-methyl-1,4,2-dioxazol-5-one-^{15}N (2e-^{15}N)

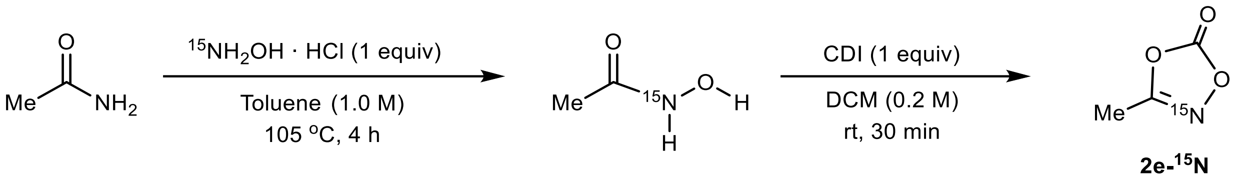

A ^{15}N-labeled acetohydroxamic acid was prepared according to the previously reported methods.[27] To a solution of acetamide (59.1 mg, 1.00 mmol) in toluene (1.00 mL, 1.00 M) was added $^{15}NH_2OH·HCl$ (1.00 equiv., 70.5 mg, 1.00 mmol). The reaction mixture was stirred at 105 °C for 4 h, and the crude mixture was cooled down to room temperature. Then, the solvent was removed under reduced pressure. The precipitated ^{15}N-labeled acetohydroxamic acid was used directly in the next step without further purification. To a solution of acetohydroxamic acid-^{15}N in DCM (5 mL, 0.2 M) was added 1,1-carbonyldiimidazole (CDI, 1.00 equiv., 162 mg, 1.00 mmol) and stirred at room temperature for 30 min. After the reaction completion, the crude mixture was added 1N HCl (aq) (10 mL) and extracted wtih DCM (10 mL x 3 times). The combined organic layers were dried over $MgSO_4$, filtered, and concentrated under reduced pressure. The crude mixture was then filtered through a pad of silica using DCM as an eluent. The filtrate was concentrated under reduced pressure to obtain the desired ^{15}N-labeled 3-methyl-1,4,2-dioxazol-5-one (**2e-^{15}N**).

3-methyl-1,4,2-dioxazol-5-one-2-^{15}N (2e-^{15}N)

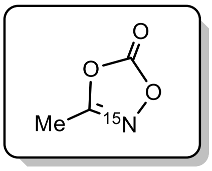

Colorless oil (31.4 mg, 31%); **^1H NMR** (600 MHz, $CDCl_3$) δ 2.35 (d, *J* = 3.1 Hz, 3H); **^{13}C NMR** (100 MHz, $CDCl_3$) δ 163.9 (d, *J* = 2.2 Hz), 154.2 (d, *J* = 1.8 Hz), 10.6 (d, *J* = 6.6 Hz). Data consistent with those previously reported except ^{15}N-coupling.[1]

***Decarboxylative ^{15}N-labeling of (S)-Naproxen using dioxazolone* 2e-^{15}N**

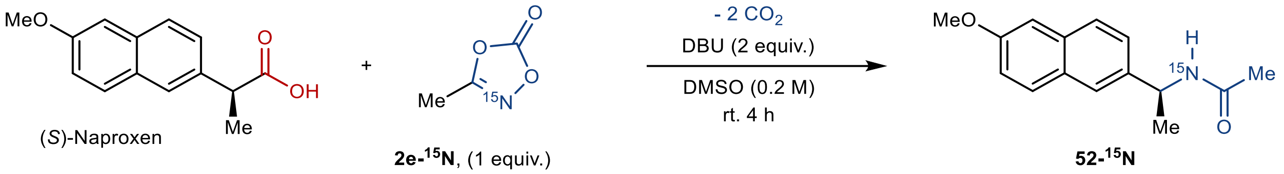

To an oven-dried 4 mL screw-capped vial equipped with oval-shaped stirring bar were added (*S*)-Naproxen (**50**, 0.200 mmol, 46.1 mg), 1,8-Diazabicyclo[5.4.0]undec-7-ene (DBU, 2.00 equiv., 0.400 mmol, 60.9 mg), and dimethylsulfoxide (DMSO, 1.00 mL, 0.200 M) under atmospheric conditions. To the reaction mixture was added 3-methyl-1,4,2-dioxazol-5-one-^{15}N (**2e-^{15}N**, 1.00 equiv., 0.200 mmol, 20.4 mg) and stirred for 4 h at room temperature. After reaction completion, the crude reaction mixture was diluted with dichloromethane (DCM, 5.0 mL), added 1N HCl aqueous solution (10 mL), and extracted with DCM (5 mL x 3 times). The combined organic layer was dried over $MgSO_4$, filtered, and concentrated under the reduced pressure. The crude mixture was subjected to silica column chromatography to provide the purified desired amide product **52-^{15}N** (eluent: DCM/Acetone, 85:15). Values of enantiomeric ratio (e.r.) were determined by HPLC analysis of **52-^{15}N** and corresponding racemic product except isotope labeling.

(*S*)-*N*-{1-(6-Methoxynaphthalen-2-yl)ethyl}acetamide-^{15}N (52-^{15}N)

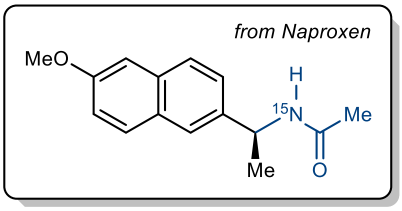

Colorless solid (24.2 mg, 50%); **m.p.** = 142 – 144 °C; **^{1}H NMR** (500 MHz, $CDCl_3$) δ 7.70 (d, J = 3.9 Hz, 1H), 7.69 (d, J = 4.3 Hz, 1H), 7.66 (d, J = 1.8 Hz, 1H), 7.39 (dd, J = 8.5, 1.9 Hz, 1H), 7.14 (dd, J = 8.9, 2.6 Hz, 1H), 7.10 (d, J = 2.5 Hz, 1H), 5.92 (dd, J = 89.5, 8.1 Hz, 1H), 5.25 (p, J = 6.4 Hz, 1H), 3.91 (s, 3H), 1.98 (d, J = 1.4 Hz, 3H), 1.55 (dd, J = 6.9, 2.9 Hz, 3H);. **^{13}C NMR** (125 MHz, $CDCl_3$) δ 169.3 (d, J = 13.7 Hz), 157.8, 138.3, 134.0, 129.5, 128.9, 127.4, 125.4, 124.6, 119.2, 105.7, 55.4, 48.8 (d, J = 9.7 Hz), 23.6 (d, J = 8.3 Hz), 21.7; **IR** (cm^{-1}) 3265, 2928, 1631, 1605, 1483, 1196, 957, 848, 609, 470; **HRMS** (EI) m/z calcd. For $C_{15}H_{17}{}^{15}NO_2$ $[M]^+$: 244.1230, found: 244.1229; **Specific Rotation** $[\alpha]_D^{29}$ = –134.5° (c 0.57, CH_2Cl_2); **HPLC Analysis**. CHIRALPAK AD-H, 32 °C; n-hexane:*i*-PrOH = 80:20, 0.5 mL/min, 250 nm, t_{R1} (major) = 10.7 min, t_{R2} (minor) = 9.2 min, >99:1 e.r.

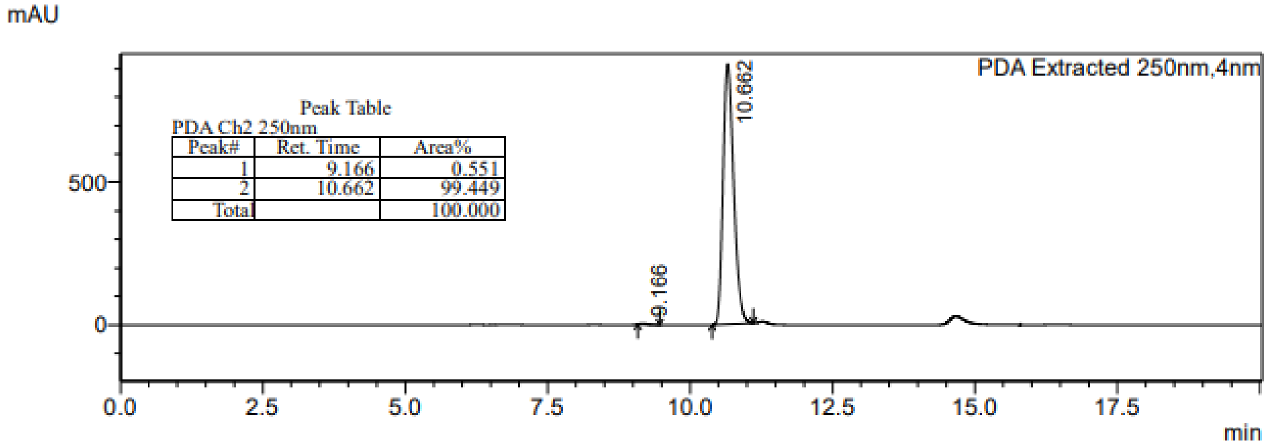

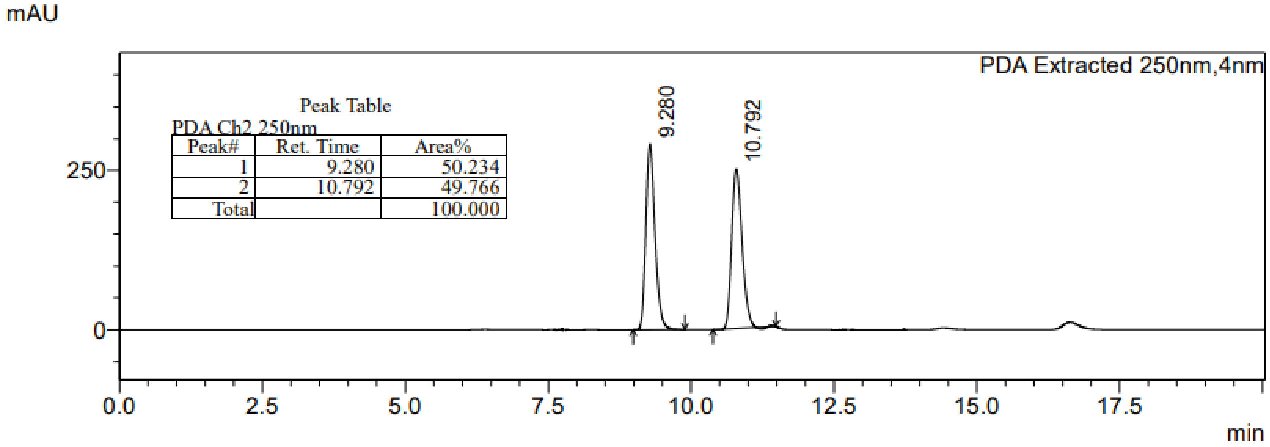

Supplementary Fig. 18 | HPLC traces of **52-^{15}N** (top) and corresponding racemic mixture (bottom).

X. Experimental Mechanistic Investigation

1. Control experiment using cyclohexanecarboxylic acid and cyclohexanecarboxylate potassium salt

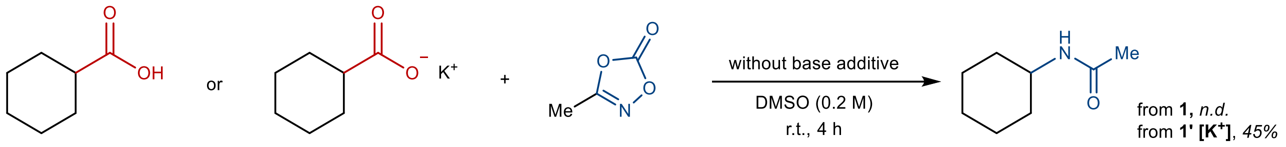

Cyclohexanecarboxylate potassium salt (**1′[K$^+$]**) was prepared according to reported procedure.[28] To an oven-dried 4 mL screw-capped vial equipped with oval-shaped stirring bar were added cyclohexanecarboxylic acid (**1**, 0.100 mmol, 12.8 mg) or cyclohexanecarboxylate potassium salt (**1′[K$^+$]**, 0.100 mmol, 16.6 mg) in dimethylsulfoxide (0.500 mL, 0.200 M). 3-Methyl-1,4,2-dioxazol-5-one (**2e**, 0.200 mmol, 20.2 mg) was added to the solution and stirred for 4 h at room temperature. Product yield was measured by ^{1}H-NMR analysis of the crude mixture in the presence of internal standard (1,3,5-trimethoxybenzene) in DMSO-d_6. While the carboxylic acid itself provided no corresponding amidated product, the carboxylate potassium salt yielded 45% of *N*-cyclohexyl acetamide product **3c**, suggesting deprotonation of carboxylic acid is essential to initiate the present decarboxylative amidation using dioxazolone.

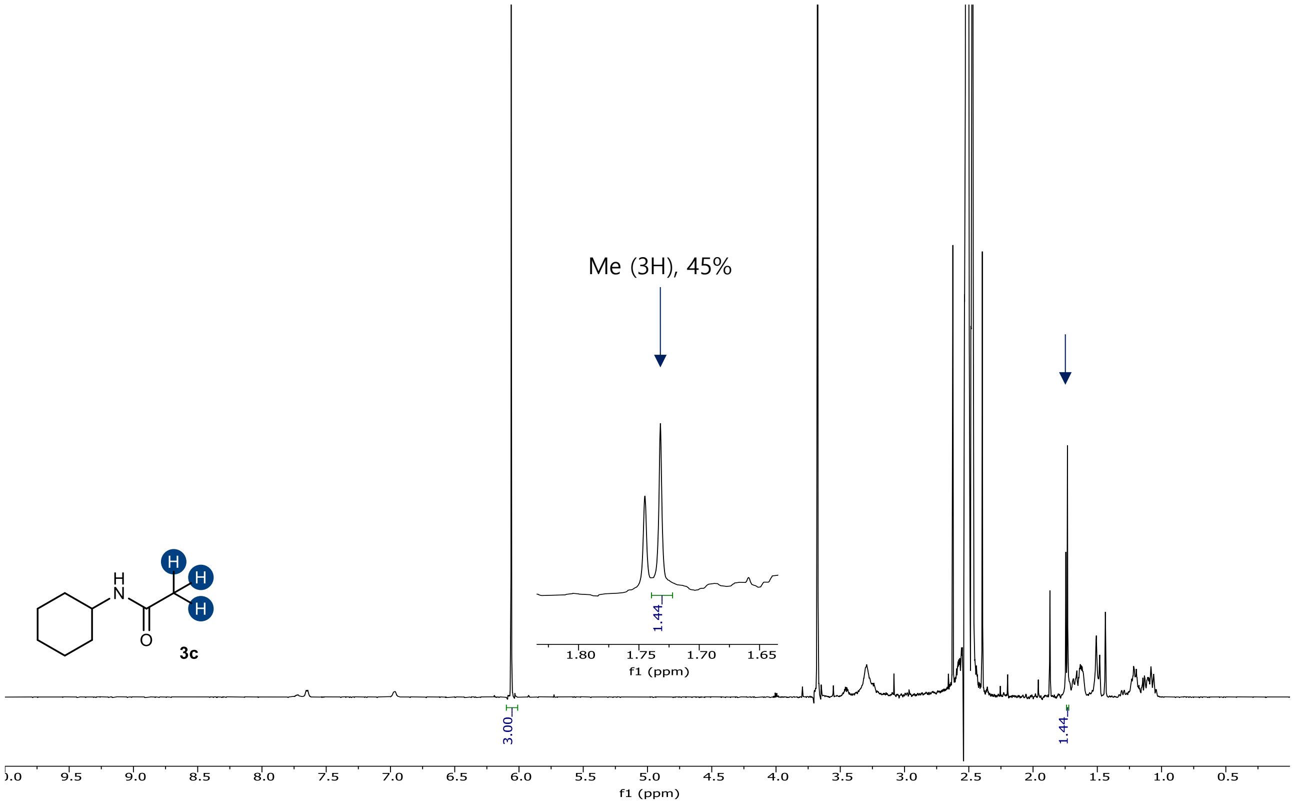

Supplementary Fig. 19 | ^{1}H NMR obtained from crude mixture of the reaction using **1′ [K+]** under standard reaction conditions without base additive.

2. Control experiment using methyl cyclohexanoate

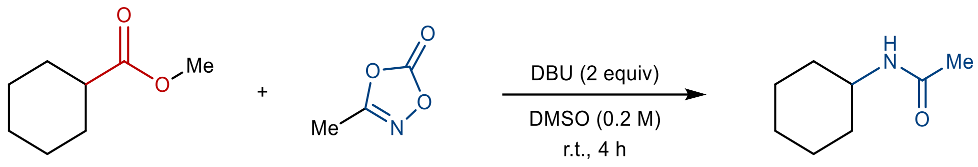

1-Me **2e** **3c,** n.d.

To an oven-dried 4 mL screw-capped vial equipped with oval-shaped stirring bar were added methyl cyclohexanecarboxylate (14.2 mg, 0.100 mmol), 1,8-Diazabicyclo[5.4.0]undec-7-ene (DBU, 2.00 equiv., 0.200 mmol, 30.4 mg), and dimethylsulfoxide (DMSO, 0.500 mL, 0.200 M) under atmospheric conditions. To the reaction mixture was added 3-methyl-1,4,2-dioxazol-5-one (**2e**, 1.00 equiv., 0.200 mmol, 10.3 mg) and stirred for 4 h at room temperature. Product yield was measured by ^{1}H-NMR analysis of the crude mixture in the presence of internal standard (1,3,5-trimethoxybenzene) in DMSO-d_6. The absence of detected product **3c** following the reaction suggests that the involvement of the nucleophilic oxygen in the carboxylic acid substrate is essential for the current decarboxylative amidation reactivity employing dioxazolone.

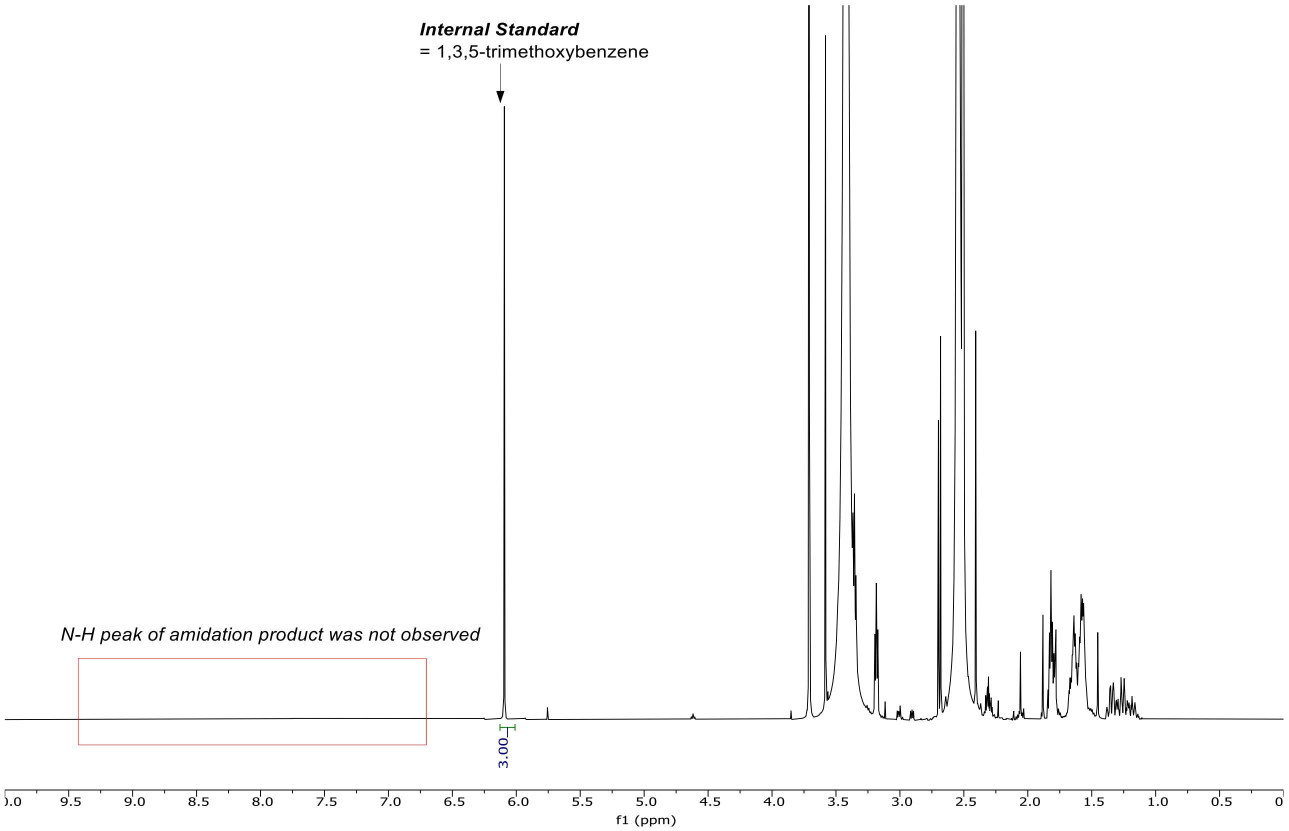

Supplementary Fig. 20 | ^{1}H NMR obtained from crude mixture of the reaction using **1-Me** under standard reaction conditions.

3. Alkyl group switching experiment

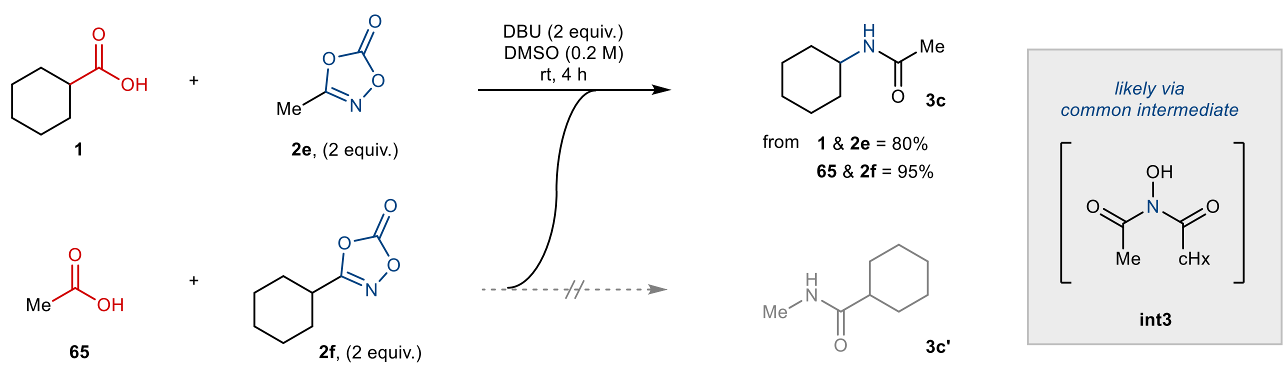

3-Cyclohexyl-1,4,2-dioxazol-5-one (**2f**) was preapred according to previously reported procedure.[1] To an oven-dried 4 mL screw-capped vial equipped with oval-shaped stirring bar were added cyclohexanecarboxylic acid **1** (or acetic acid **65**, 0.200 mmol, 25.6 mg or 12.0 mg, respectively), 1,8-Diazabicyclo[5.4.0]undec-7-ene (DBU, 2.00 equiv., 0.400 mmol, 60.9 mg), and dimethylsulfoxide (DMSO, 1.00 mL, 0.200 M) under atmospheric conditions. To the reaction mixture was added 3-methyl-1,4,2-dioxazol-5-one **2e** (or 3-cyclohexyl-1,4,2-dioxazol-5-one **2f**, 1.00 equiv., 0.200 mmol, 20.6 mg or 67.7 mg, respectively) and stirred for 4 h at room temperature. After reaction completion, the crude reaction mixture was diluted with dichloromethane (DCM, 5.0 mL), added 1N HCl aqueous solution (10 mL), and extracted with DCM (5 mL x 3 times). The combined organic layer was dried over $MgSO_4$, filtered, and concentrated under the reduced pressure. The crude mixture was subjected to silica column chromatography to provide the purified desired N-alkylamide products (eluent: Dichloromethane/Acetone, 90:10). As a result, both substrate combination (**1/2e** and **65/2f**) provided same product *N*-cylohexyl acetamide **3c** with high yield (80% and 95%, respectively), implies that symmetric intermediate **int3** likely involves during the course of the reaction.

4. Control expriement using *N*-acetyl-*N*-{(tert-butyldimethylsilyl)oxy}cyclohexanecarboxamide (Int3-TBS)

Synthetic procedure of sillyl-protected N,N-dicarbonyl-N-oxide intermediate (Int3-TBS)

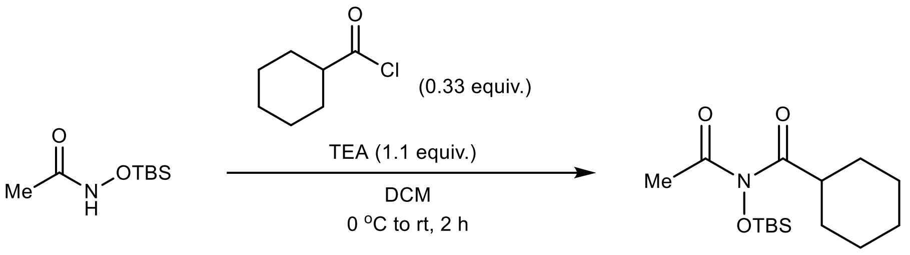

Int3-TBS

TBS-protected methyl hydroxamic acid was prepared following reported procedure.[29] Subsequently, to a 250 mL round-botton flask were added TBS-protected methyl hydroxamic acid (3.00 mmol, 568 mg), TEA (3.30 mmol, 334 mg) and dichloromethane (20.0 mL). The resulting solution was stirred for 10 min. To a reaction solution was added cyclohexanecarbonyl chloride (3.00 mmol, 440 mg) solution in dichloromethane (10.0 mL) as dropwise manner at 0 °C. After the dropwise addition, the solution was warmed up to room temperature and stirred for 2 h. After the reaction completion, the solvent was removed under the reduced pressure and purfied *N*-acetyl-*N*-{(tert-butyldimethylsilyl)oxy}cyclohexanecarboxamide **Int3-TBS** was obtained by column chromatography (eluent = *n*-hexane/EtOAc. 80:20).

_N_-Acetyl-_N_-{(_tert_-butyldimethylsilyl)oxy}cyclohexanecarboxamide (Int3-TBS)

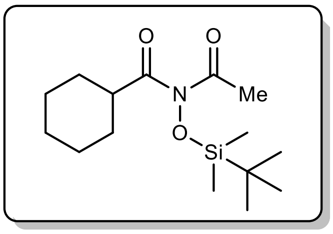

Colorless liquid (693 mg, 77%); **^{1}H NMR** (600 MHz, $CDCl_3$) δ 3.15 (tt, *J* = 11.5, 3.3 Hz, 1H), 2.37 (s, 3H), 1.89 (ddd, *J* = 15.3, 4.7, 2.4 Hz, 2H), 1.79 (dt, *J* = 13.2, 3.6 Hz, 2H), 1.69 (dqd, *J* = 10.6, 3.4, 1.8 Hz, 1H), 1.41 (qd, *J* = 12.2, 3.2 Hz, 2H), 1.35 – 1.18 (m, 3H), 1.01 (s, 9H), 0.17 (s, 6H); **^{13}C NMR** (150 MHz, $CDCl_3$) δ 176.9, 171.3, 44.0, 29.2, 26.1, 26.0, 25.8, 25.1, 18.6, -4.44; **IR** (cm^{-1}) 2930, 2856, 1714, 1365, 1242, 1156, 829, 784, 624; **HRMS** (FAB) m/z calcd. For $C_{15}H_{30}NO_3Si$ $[M+H]^+$: 300.1995, found: 300.1998.

Control expriement using N-acetyl-N-{(tert-butyldimethylsilyl)oxy}cyclohexanecarboxamide Int3-TBS

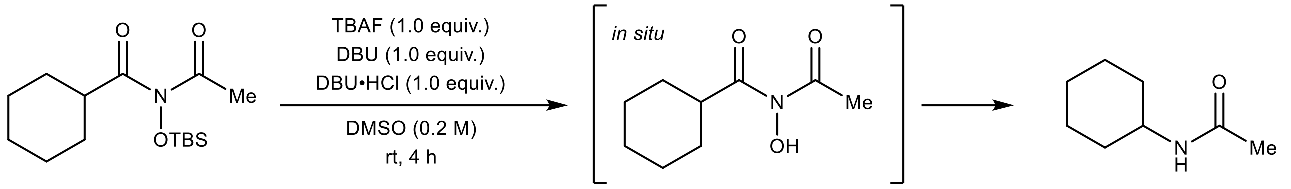

Int3-TBS **Int3** **3c**

To an oven-dried 4 mL screw-capped vial equipped with oval-shaped stirring bar were added *N*-acetyl-*N*-{(tert-butyldimethylsilyl)oxy}cyclohexanecarboxamide **Int3-TBS** (1.00 mmol, 299 mg) and dimethylsulfoxide (5.00 mL, 0.200 M) under atmospheric conditions. To the resulting solution were added 1,8-Diazabicyclo[5.4.0]undec-7-ene (DBU, 1.00 equiv, 1.00 mmol, 152 mg) and DBU•HCl (1.00 equiv, 1.00 mmol, 189 mg) to mimick the standard reaction conditions. Tetrabutylammonium fluoride (TBAF) 1.0 M solution in THF (1.00 mL, 1.00 mmol) was added to the reaction solution in dropwise manner for 30 min at room temperature. The resulting solution was stirred for 4 h at room temperature. After the reaction completion, the crude reaction mixture was diluted with dichlorimethane (DCM, 5.0 mL), added 1N HCl aqueous solution (10 mL), and extracted with DCM (5.0 mL x 3 times). The combined organic layer was dried over $MgSO_4$, filtered, and concentrated under the rduced pressure. The crude mixture was subjected to silica column chromatography (eluent = dichloromethane/acetone = 80:20) to provide the purfied *N*-cyclohexyl acetamide **3c** (62.1 mg, 44%).

5. Control experiment using hydroxamate intermediates

Synthesis of hydroxamate intermediate H[Int5-A]

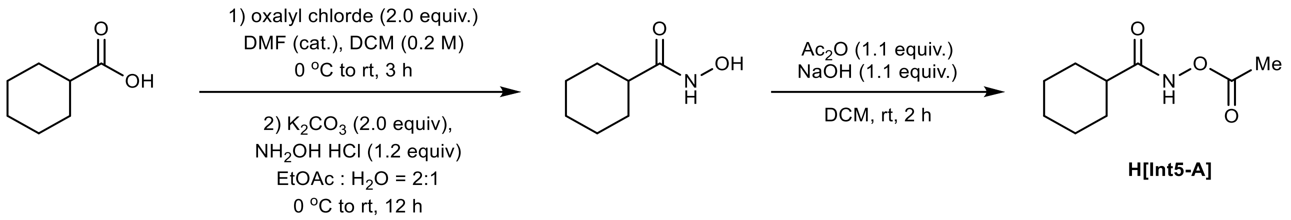

To an oven-dried 250 mL round-bottom flask equipped with oval-shaped stirring bar were added cyclohexyl carboxylic acid (20.0 mmol, 2.56 g) and dichloromethane (100 mL, 0.2 M). The oxalyl chloride (40.0 mmol, 5.08 g) and catalytic amount of *N*,*N*-dimethylformamide (DMF, 2~3 drops) were added at 0 °C. The resulting solution was warmed up to room temperature and stirred for 3 h. After the reaction, the solvent was removed under the reduced pressure to obtain crude cyclohexylcarbonyl chloride. To the two-phase solution of K_2CO_3 (2.00 equiv, 40.0 mmol, 5.53 g) and NH_2OH HCl (1.2 equiv, 24.0 mmol, 1.67 g) in EtOAc and H_2O (2:1, 0.2 M) was added crude cyclohexylcarbonyl chloride in dropwise manner at 0 °C. The resulting mixture was warmed up to room temperature and stirred for 12 h. After the reaction completion, the crude mixture was extracted with EtOAc (50 mL x 3 times) and washed with brine (100 mL). The collected organic layer was evaporated under the reduced pressure. The purified cyclohexyl hydroxamic acid was obtained by recrystallization (dichloromethane/*n*-pentane).

To an oven-dried 100 mL round-bottom flask equipped with oval-shaped stirring bar were added cyclcohexyl hydroxamic acid (5.00 mmol, 716 mg), NaOH (1.10 equiv., 5.50 mmol, 220 mg) and dichloromethane (25.0 mL, 0.200 M). To the resulting solution was added acetic anhydride (Ac_2O, 1.10 equiv., 5.50 mmol, 561 mg) and stirred for 2 h at room temperature. After the reaction completion, water (20 mL) was added to the resulting solution and extracted with dichloromethane (30 mL x 3 times). The combined organic layer was dried over $MgSO_4$, filtered and concentrated under reduced pressure. The crude mixture was recrystallized (dichloromethane/*n*-pentane) under -30 °C to obtain purifed N-acetoxycyclohexanecarboxamide (**H[Int5-A]**).

***N*-Hydroxycyclohexanecarboxamide**

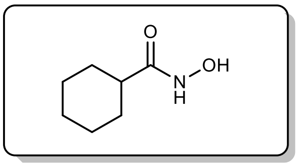

Colorless solid (1.95 g, 68%); **^{1}H NMR** (500 MHz, DMSO-d_6) δ 10.31 (s, 1H), 8.61 (s, 1H), 1.95 (tt, J = 11.7, 3.5 Hz, 1H), 1.75 – 1.64 (m, 2H), 1.64 – 1.54 (m, 3H), 1.35 (qd, J = 12.3, 3.3 Hz, 2H), 1.24 – 1.07 (m, 3H); **^{13}C NMR** (125 MHz, DMSO-d_6) δ 172.3, 41.2, 29.1, 25.4, 25.3; *Data consistent with those previously reported.*[1]

N-acetoxycyclohexanecarboxamide (H[int5-A]).

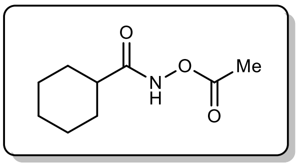

Colorless solid (746 mg, 80%); **^{1}H NMR** (500 MHz, $CDCl_3$) δ 8.90 (s, 1H), 2.26 – 2.16 (m, 4H), 1.91 – 1.84 (m, 2H), 1.84 – 1.77 (m, 2H), 1.70 – 1.65 (m, 1H), 1.59 – 1.46 (m, 2H), 1.36 – 1.17 (m, 3H); **^{13}C NMR** (125 MHz, $CDCl_3$) δ 174.5, 169.1, 42.4, 29.3, 25.5(9), 25.5(6), 18.4; *Data consistent with those previously reported.*[30]

Control experiement using hydroxamate H[Int5-A]

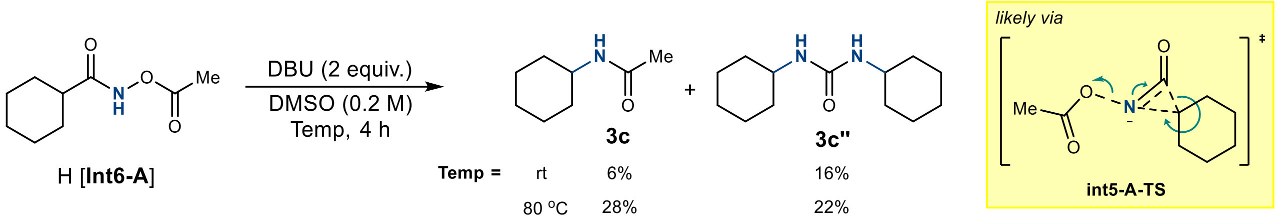

To an oven-dried 4 mL screw-capped vial equipped with oval-shaped stirring bar were added indicated hydroxamate substrate (**H[Int5-A]** and **H[Int5-B]**, 0.1 mmol, 18.5 mg) and anhydrous dimethylsulfoxide (DMSO, 0.500 mL, 0.200 M). To the resulting solution was added 1,8-Diazabicyclo[5.4.0]undec-7-ene (DBU, 1.00 equiv., 0.200 mmol, 30.5 mg) and stirred for 4 h at indicated temperature. Product yield was measured by ^{1}H-NMR analysis of the crude mixture in the presence of internal standard (1,3,5-trimethoxybenzene) in DMSO-d_6. In the reaction using **H[Int5-A]** at room temperature, only 6% of **3c** and 16% of **3c″** were obtained. However, an increased yield of the product (**3c** = 28%, **3c″** = 22%) was observed at an elevated temperature (80 °C). This result suggests that the desired amidated product **3c** can potentially be generated according to the computationally proposed mechanism following **Path A**, involving the Lossen-type rearrangement of **Int5-A**.

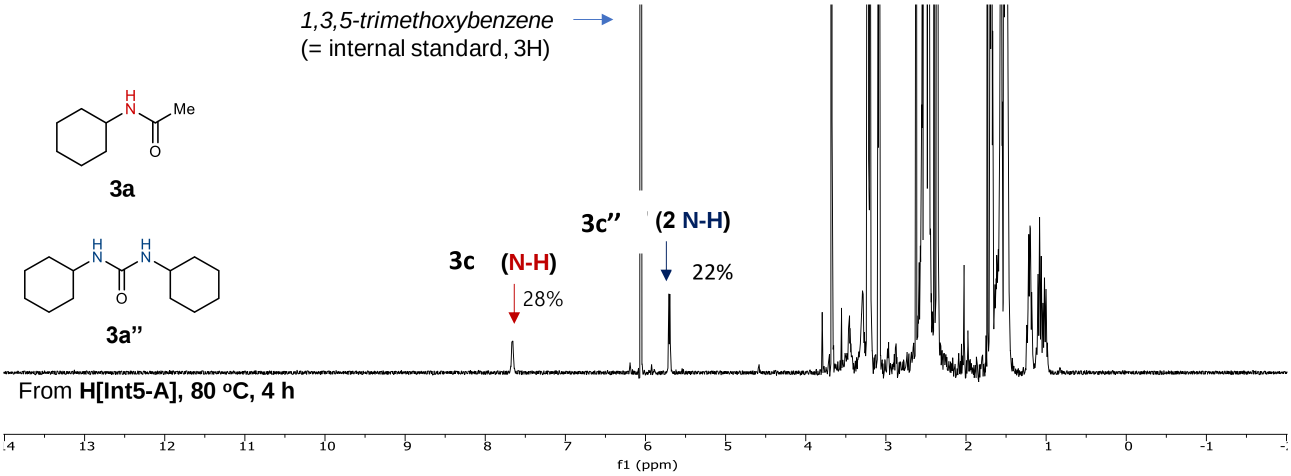

Supplementary Fig. 21 | ^{1}H-NMR obtained from crude reaction mixture using hydroxamate intermediate.

XI. Computation details

All calculations were performed using the density functional theory (DFT)[31] as implemented in the Gaussian 09 suite of programs.[32] All computations were performed on domestic servers at KAIST and High-Performance Computing Resources in the IBS Research Solution Center. Conformer search was conducted using CREST for local-minimum geometry.[33] Geometry optimizations were performed using the M06-2X functional[34] and a 6-31G** for all atoms. Vibrational frequency calculations were carried out at the same level of theory as that used for geometry optimizations, and zero-point energies and entropy correction terms were derived. Transition states were realized by the presence of single imaginary frequency and confirmed by intrinsic reaction coordinate calculations (IRC).[35,36] Single point energies of optimized structures were calculated with the same functional and a triple-zeta basis set 6-311+G**[37] for all atoms. Solvation correction energies were evaluated from the self-consistent reaction field (SCRF) approximations with dimethylsulfoxide (DMSO, ε = 46.826).[38–40] Final solution phase Gibbs free energies were calculated as follows:

$$G(Sol) = G(gas) + G^{solv} \quad (1)$$

$$G(gas) = H(gas) - TS(gas) \quad (2)$$

$$H(Gas) = E(SCF) + ZPE \quad (3)$$

$$\Delta G(Sol) = \Sigma G(Sol) \text{ for products} - \Sigma G(Sol) \text{ for reactants} \quad (4)$$

G(Sol) is the solvation-corrected Gibbs free energy; G(gas) is the gas phase free energy; H(gas) is the enthalpy in the gas phase; T is the temperature (298.15 K); S(gas) is the entropy in the gas phase; E(SCF) is the electronic energy converged from the self-consistent field method; ZPE is the vibrational zero-point energy; and S for the vibrational entropy correction. Note that here entropy refers specifically to the vibrational/rotational/translational entropy of the solute(s). The solvent entropies are implicitly included in the continuum model.

1. Potential energy surfaces (PES) toward generation of dicarbonyl N-hydroxy intermediate.

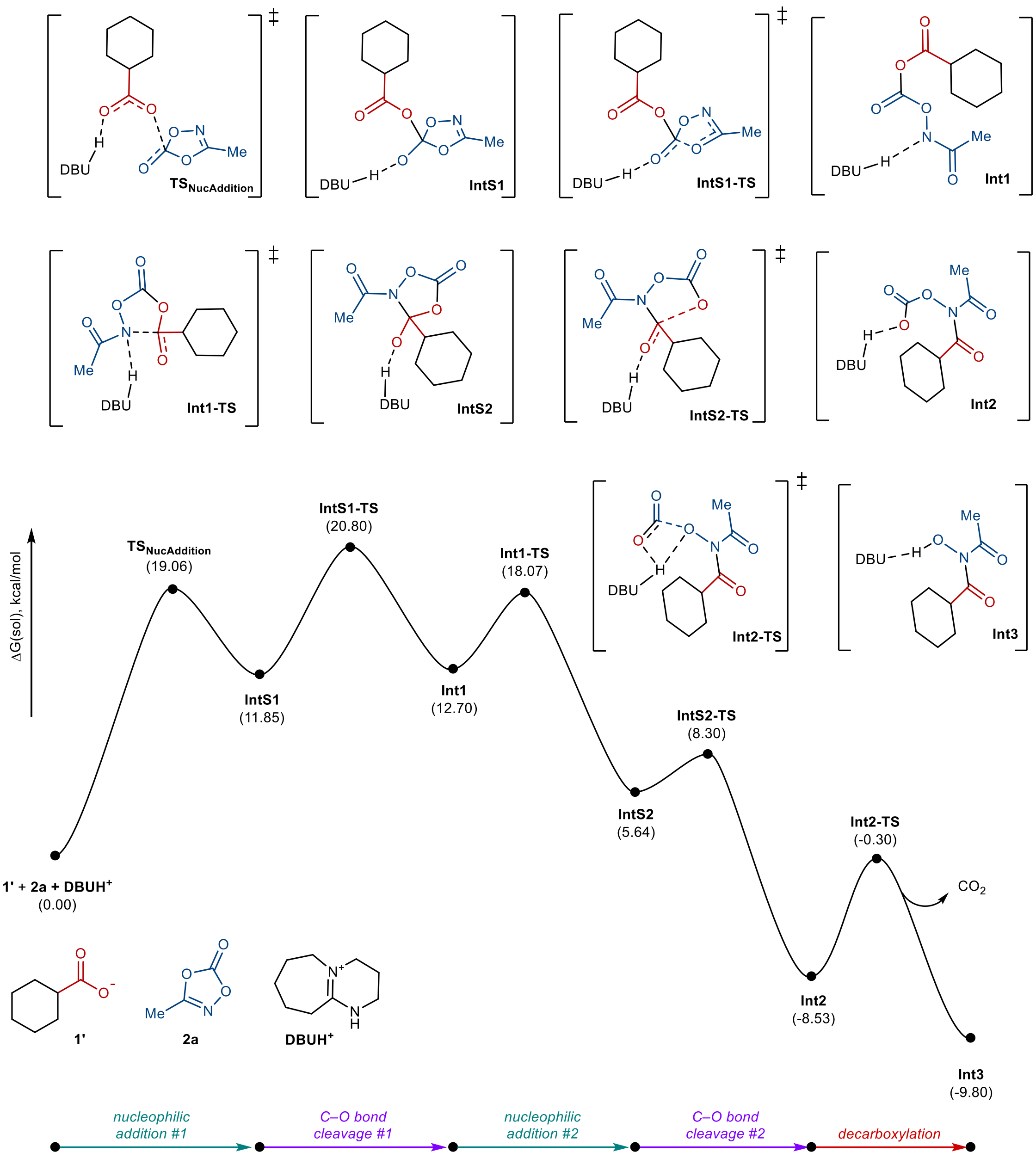

Supplementary Fig. 22 | Potential energy surfaces (PES) of formation of **Int3**

According to computational analysis, the nucleophilic attack of **1′** to the carbonyl position of dioxazolone **2e** (nucleophilic addition #1) was determined to be kinetically plausible, leading to the formation of the quaternary carbon intermediate **IntS1** ($\Delta G^{\ddagger}$ = 19.1 kcal/mol). Subsequently, the following C–O bond cleavage #1 (**IntS1-TS**), resulting in the ring-opened intermediate **Int1**, was also found to be kinetically possible ($\Delta G^{\ddagger}$ = 9.0 kcal/mol). Consequently, the second nucleophilic addition (**Int1-TS**) to provide the 5-membered-ring intermediate **IntS2** is both kinetically and thermodynamically favored (nucleophilic addition #2, $\Delta G^{\ddagger}$ = 5.4 kcal/mol, ΔG = –7.1 kcal/mol). Another C–O bond cleavage #2 (**IntS2-TS**) was also found to be readily available, resulting in the N-carbonate intermediate **Int2** ($\Delta G^{\ddagger}$ = 2.7 kcal/mol, ΔG = –14.2 kcal/mol). Subsequently, the decarboxylation of **Int2**, resulting in the crucial *N*,*N*-dicarbonyl-*N*-hydroxy intermediate, displayed a

low activation barrier (8.2 kcal/mol) with a negative free energy difference of –1.3 kcal/mol. In summary, starting from the nucleophilic addition of carboxylate to dioxazolone, the important intermediate Int3 is both kinetically and thermodynamically accessible.

2. Potential energy surfaces (PES) for oxazirine intermediate formation from N-oxide intermediate

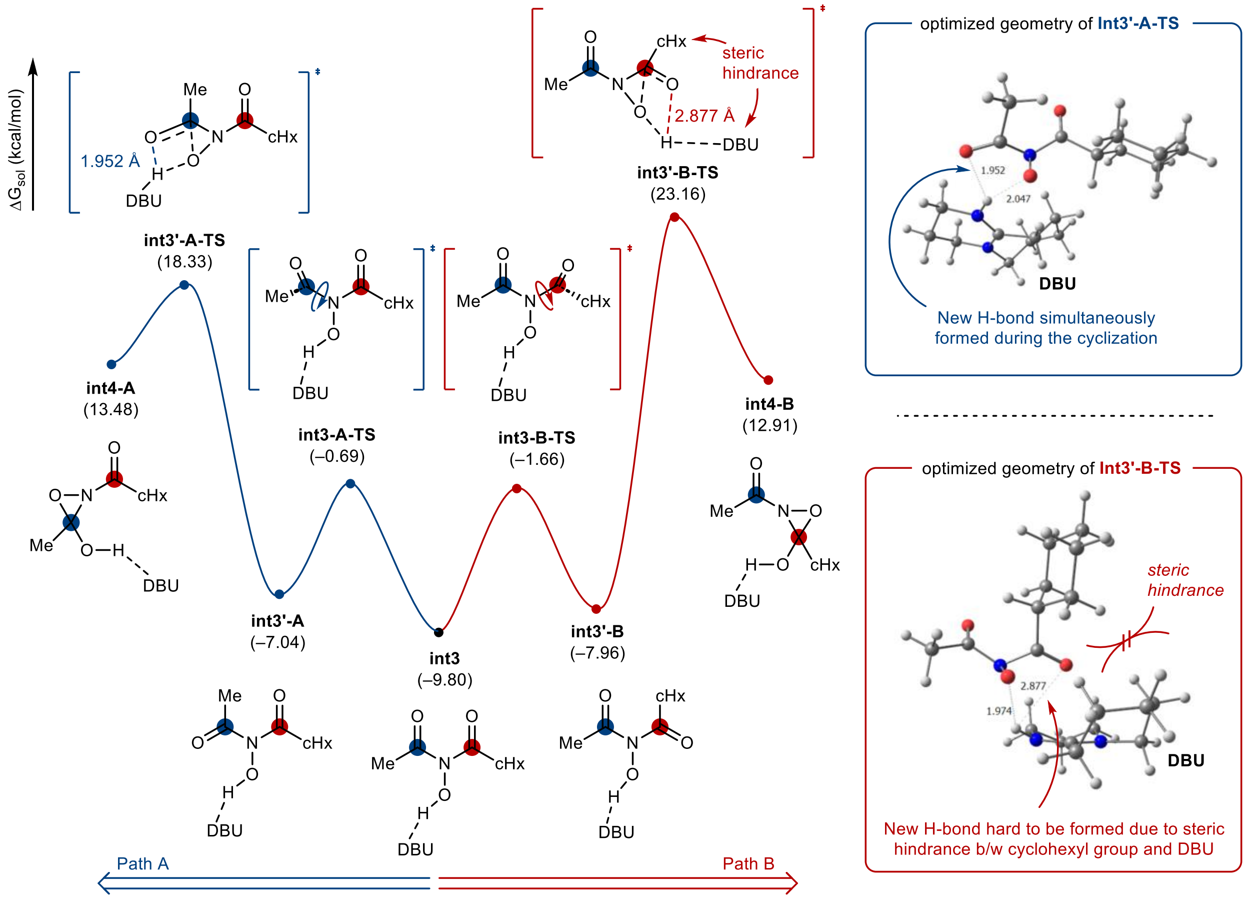

Supplementary Fig. 23 | Potential energy surfaces (PES) for oxazirine intermediate formation (**Int4**) from **Int3**

The cyclization of the N,N-dicarbonyl-N-hydroxy intermediate **Int3** can result in the formation of oxaziridine intermediates (**Int4-A** and **Int4-B**) through nucleophilic addition of the hydroxy group to the adjacent carbonyl group (-COMe: Path A, -COcHx: Path B) following proper rotation (**Int3′-A** and **Int3′-B**, respectively). Our computational findings indicate that the nucleophilic attack of the hydroxy group towards the acetyl group (Path A) is kinetically favored over Path B (addition to -COcHx). Structural analysis of **Int3′-A-TS** reveals the establishment of a new hydrogen bond with the carbonyl oxygen and DBUH+ during the reaction, whereas **Int3′-B-TS** does not due to steric hindrance between the cyclohexyl group and the DBU base pair. Based on these computational results, we conclude that the reaction likely follows Path A in Fig.S22.

3. Plausible formation of C(sp^3)–N bond coupled product from oxazirine intermediate

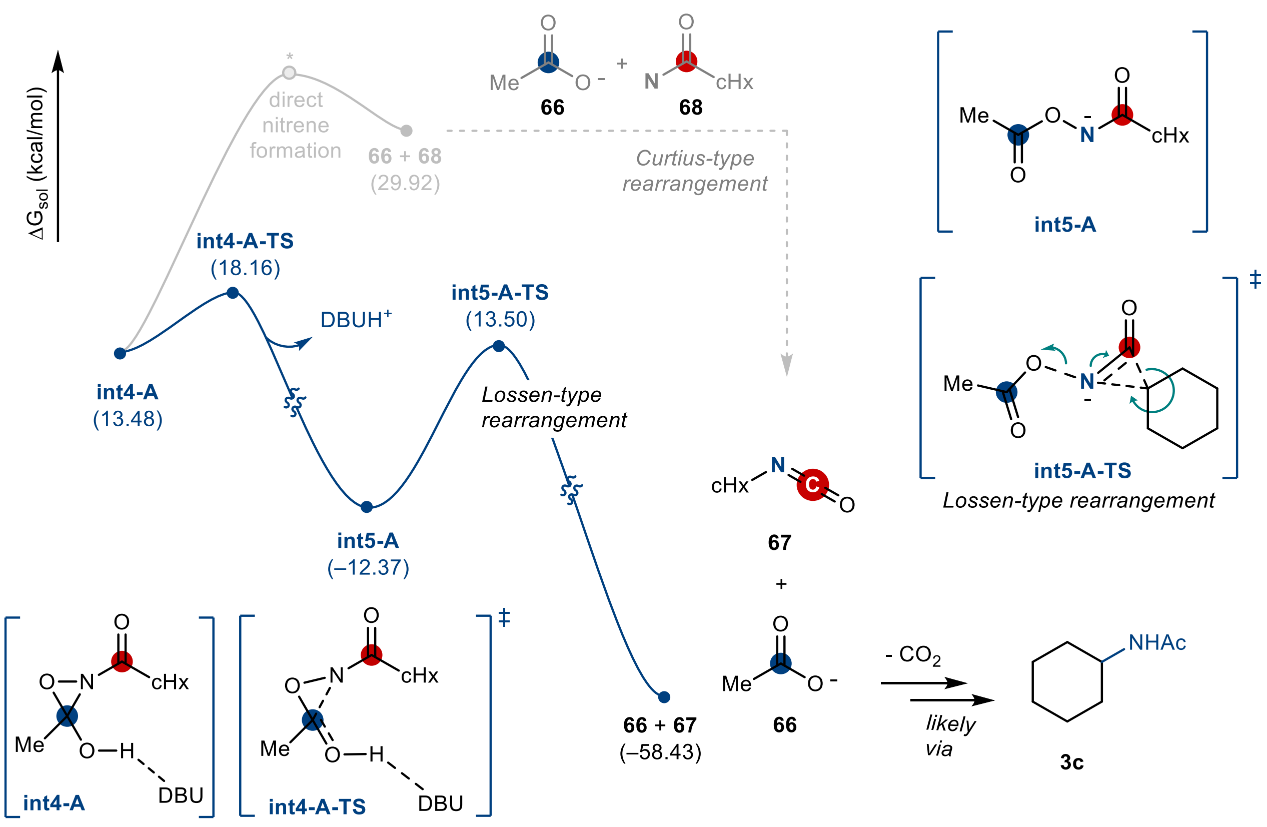

Supplementary Fig. 24 | Potential energy surfaces (PES) for C–N coupled product **3c** from **Int4-A**

Upon the formation of **Int4-A**, computational results indicated that the subsequent ring-opening of Int4-A to generate hydroxamate Int5-A is readily achievable, both kinetically and thermodynamically, with values of $\Delta G^{\ddagger}$ = 4.7 kcal/mol and ΔG = –25.9 kcal/mol, respectively. Furthermore, a Lossen-type rearrangement of **Int5-A** leading to **66** along with cyclohexyl isocyanate **67** was demonstrated to be accessible ($\Delta G^{\ddagger}$ = 25.9 kcal/mol, ΔG = –46.1 kcal/mol). It is noteworthy that the decarboxylative coupling of the isocyanate and carboxylate via a Hofmann-type rearrangement was previously reported in a reaction, resulting in the formation of alkylamides. In contrast, a direct conversion to acyl nitrene **68** along with acetate **66** was found to be thermodynamically unfavorable (ΔG = 16.44 kcal/mol) for the subsequent Curtius rearrangement.

4. Energy components from DFT calculation

Supplementary Table 6 | Computed energies of the optimized geometries (solvation = dimethylsulfoxide)

	**E(SCF)+G^{Solv} / (Hartree)**	**ZPE / (kcal/mol)**	**S(gas) / (cal/mol·K)**
	6-311+G**	6-31G**	6-31G**
**1a′**	-423.91978558	108.801	90.147
DBUH$^+$	-462.50436695	165.235	96.120
**2a**	-396.48741403	42.647	76.341
**$TS_{NucAddition}$**	-1282.92834207	319.139	171.627
**IntS1**	-1282.93073538	317.046	183.745
**IntS1-TS**	-1282.92311112	319.058	176.542
**Int1**	-1282.92715565	318.175	192.175
**Int1-TS**	-1282.92911196	318.947	172.668
**IntS2**	-1282.94715162	318.049	173.396
**IntS2-TS**	-1282.94410241	318.840	173.622
**Int2**	-1282.96754826	318.216	178.528
**Int2-TS**	-1282.95151554	317.122	181.010
**Int3**	-1094.36923007	308.714	172.050
**CO_2**	-188.57671233	7.494	51.056
**Int3-A-TS**	-1094.35488166	308.433	164.538
**Int3-B-TS**	-1094.35357094	308.454	164.118
**Int3′-A**	-1094.36776783	309.172	165.845
**Int3′-B**	-1094.36876959	309.253	167.085
**Int3′-A-TS**	-1094.32715011	308.480	163.904
**Int3′-B-TS**	-1094.32252348	308.977	159.112
**Int4-A**	-1094.33491873	308.499	163.908
**Int4-B**	-1094.33463788	308.18	165.341
**Acetate (66)**	-228.59447637	30.678	30.678
**Cyclohexyl acyl nitrene (68)**	-403.17875342	109.061	90.778
**Int4-A-TS**	-1094.3295144	308.108	158.268
**Int5-A**	-631.84950384	142.866	113.253
**Int5-A-TS**	-631.79941345	140.202	122.977
**Cyclohexyl isocyanate (67)**	-403.30219865	110.500	92.381

Spectral Copies of ^{1}H, ^{13}C, and ^{19}F NMR of Compounds Obtained in this Study

N-[{3,5-Bis(trifluoromethyl)benzoyl}oxy]acetamide (2d)

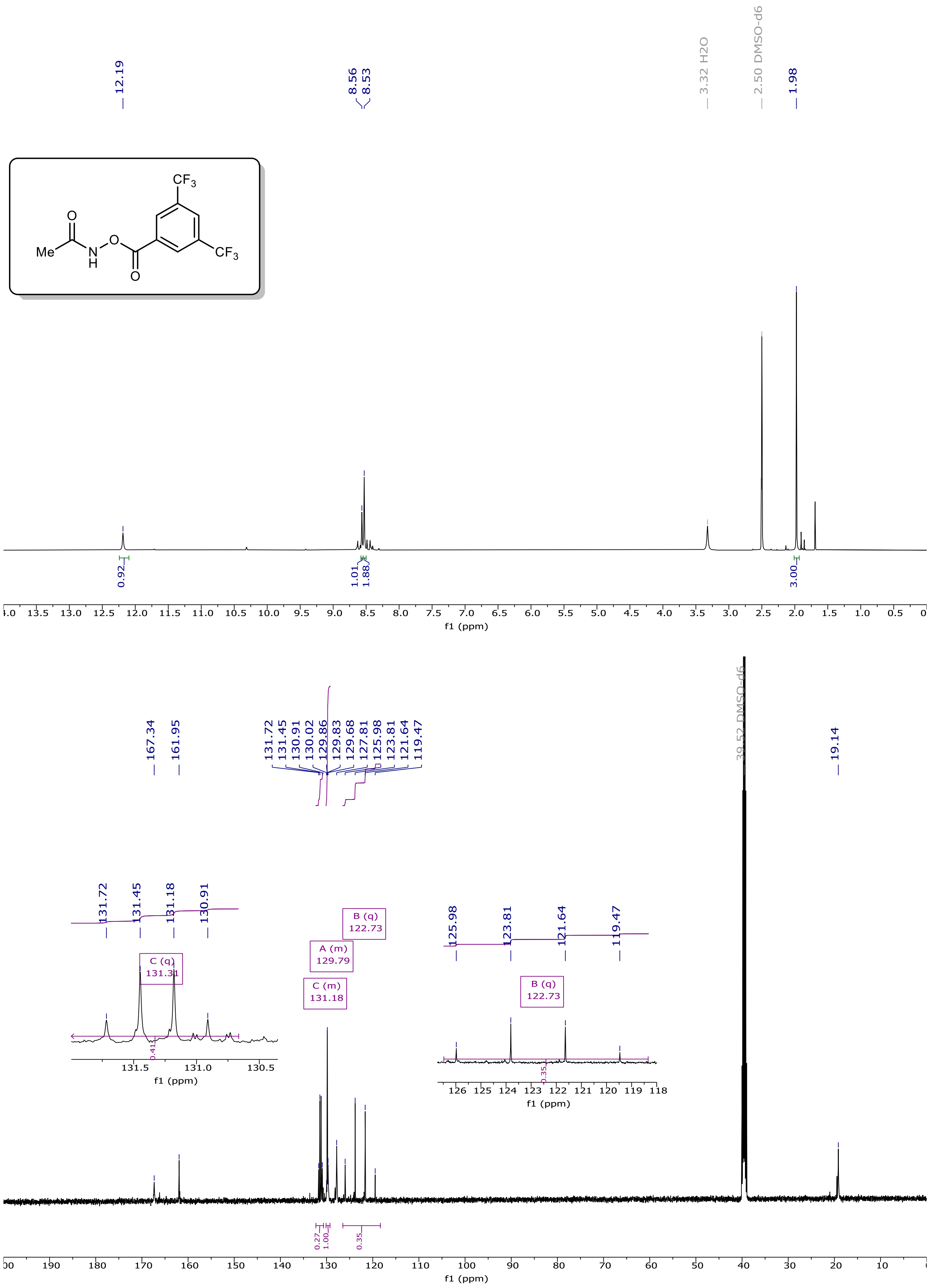

Supplementary Fig. 25 | ^{1}H (top) and ^{13}C (bottom) NMR spectra of **2d.**

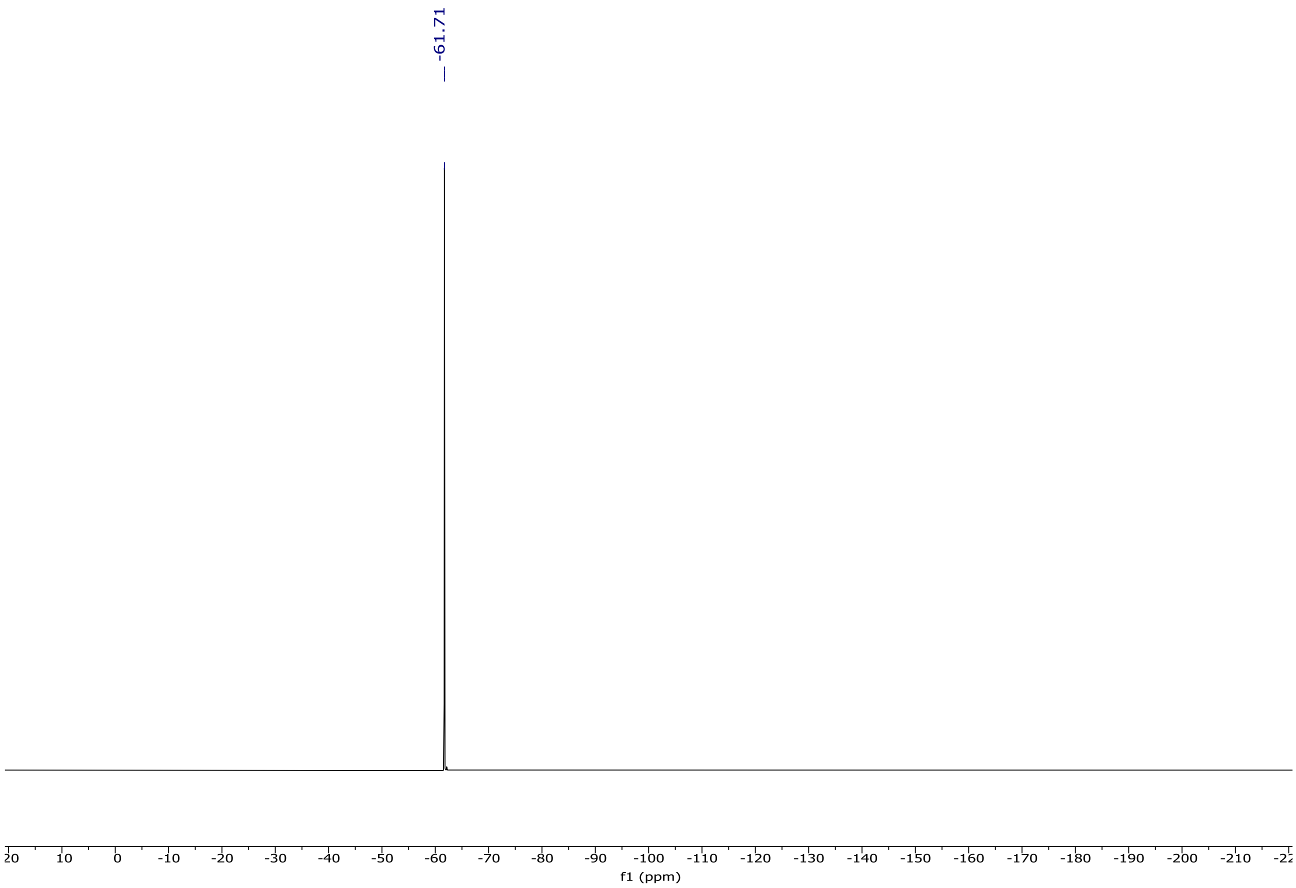

Supplementary Fig. 26 | ^{19}F NMR spectra of **2d.**

***N*-Cyclohexylacetamide (3c)**

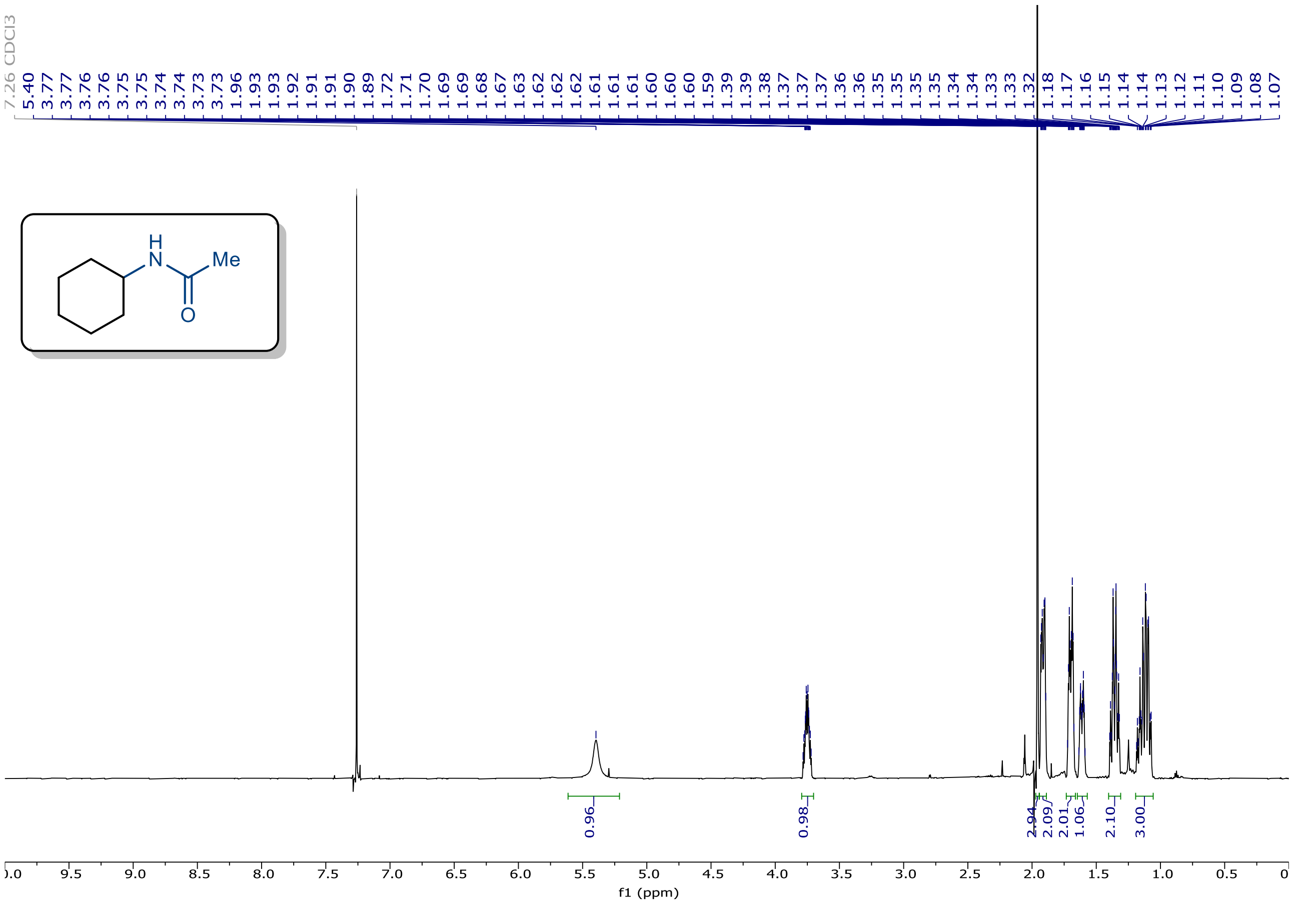

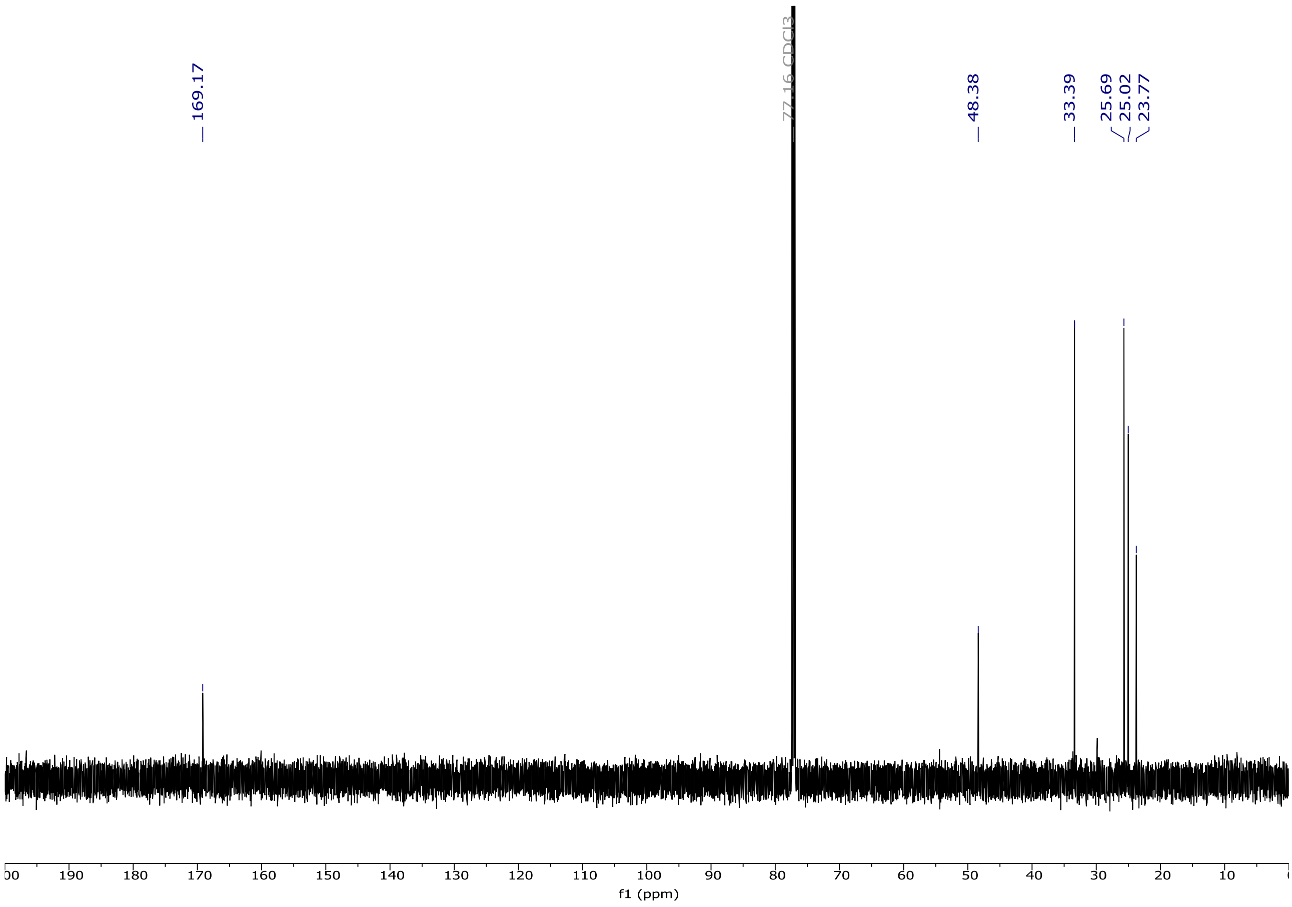

Supplementary Fig. 27 | ^{1}H (top) and ^{13}C (bottom) NMR spectra of **3c.**

***N*-Cyclopentylacetamide (4)**

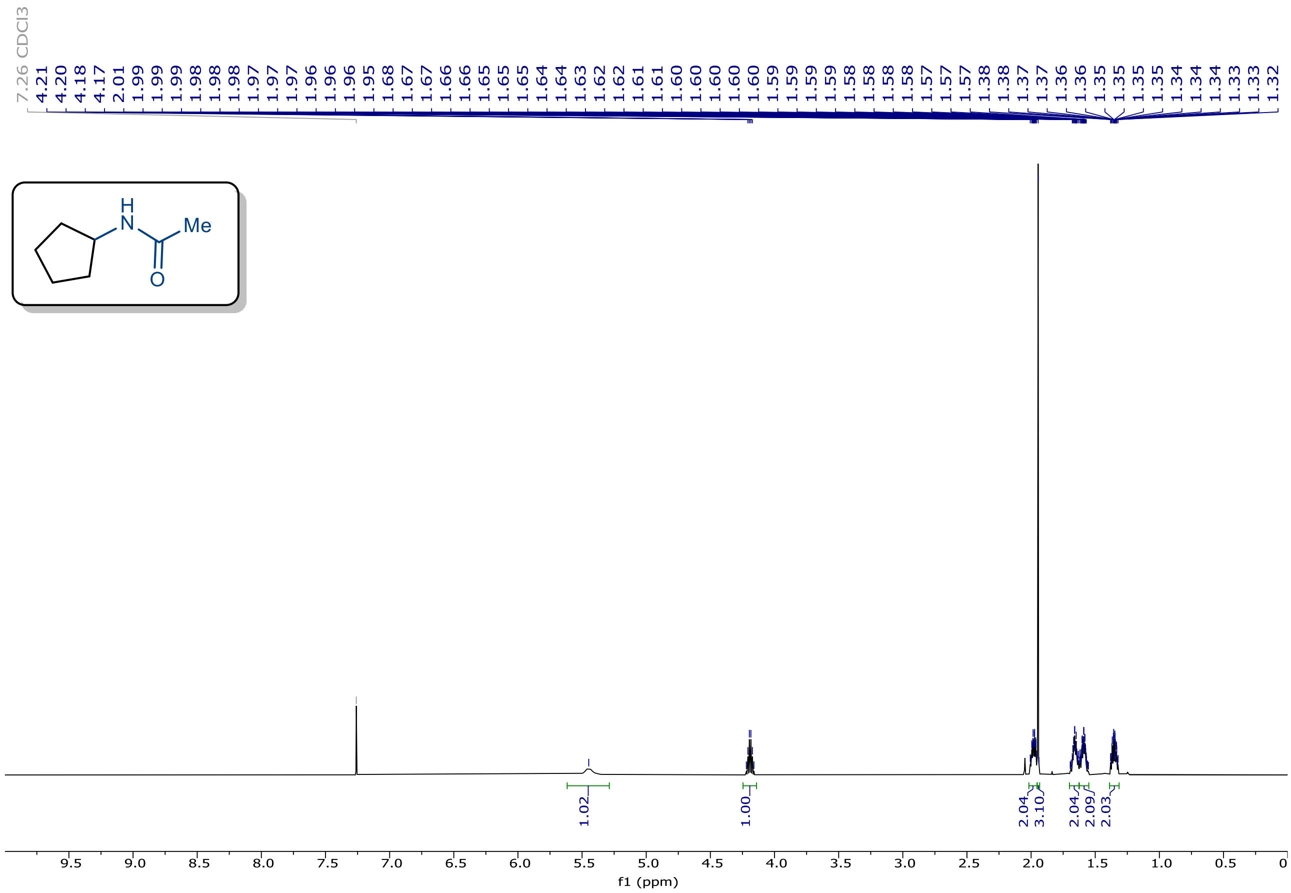

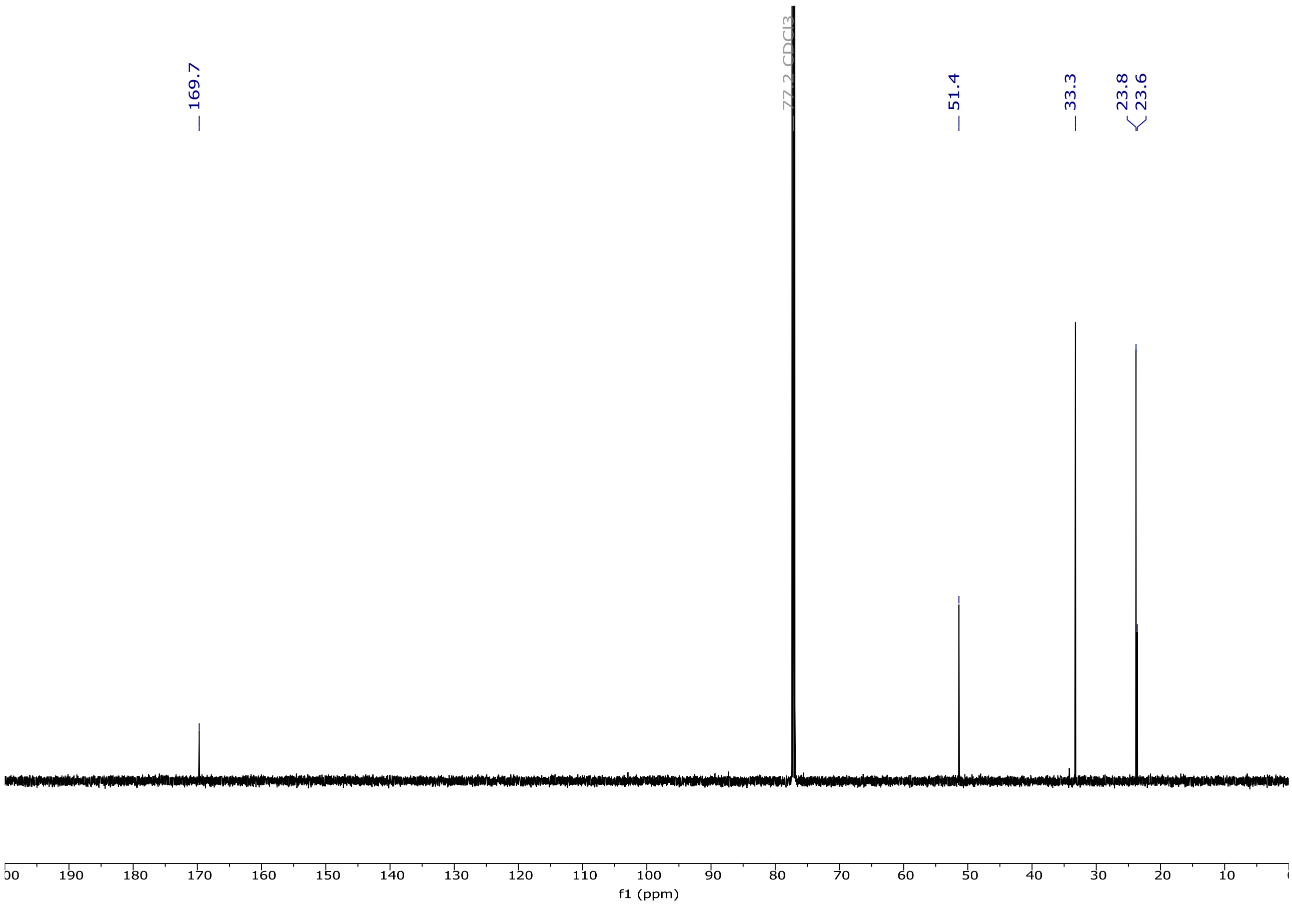

Supplementary Fig. 28 | ^{1}H (top) and ^{13}C (bottom) NMR spectra of **4.**

N-Cyclobutylacetamide (5)

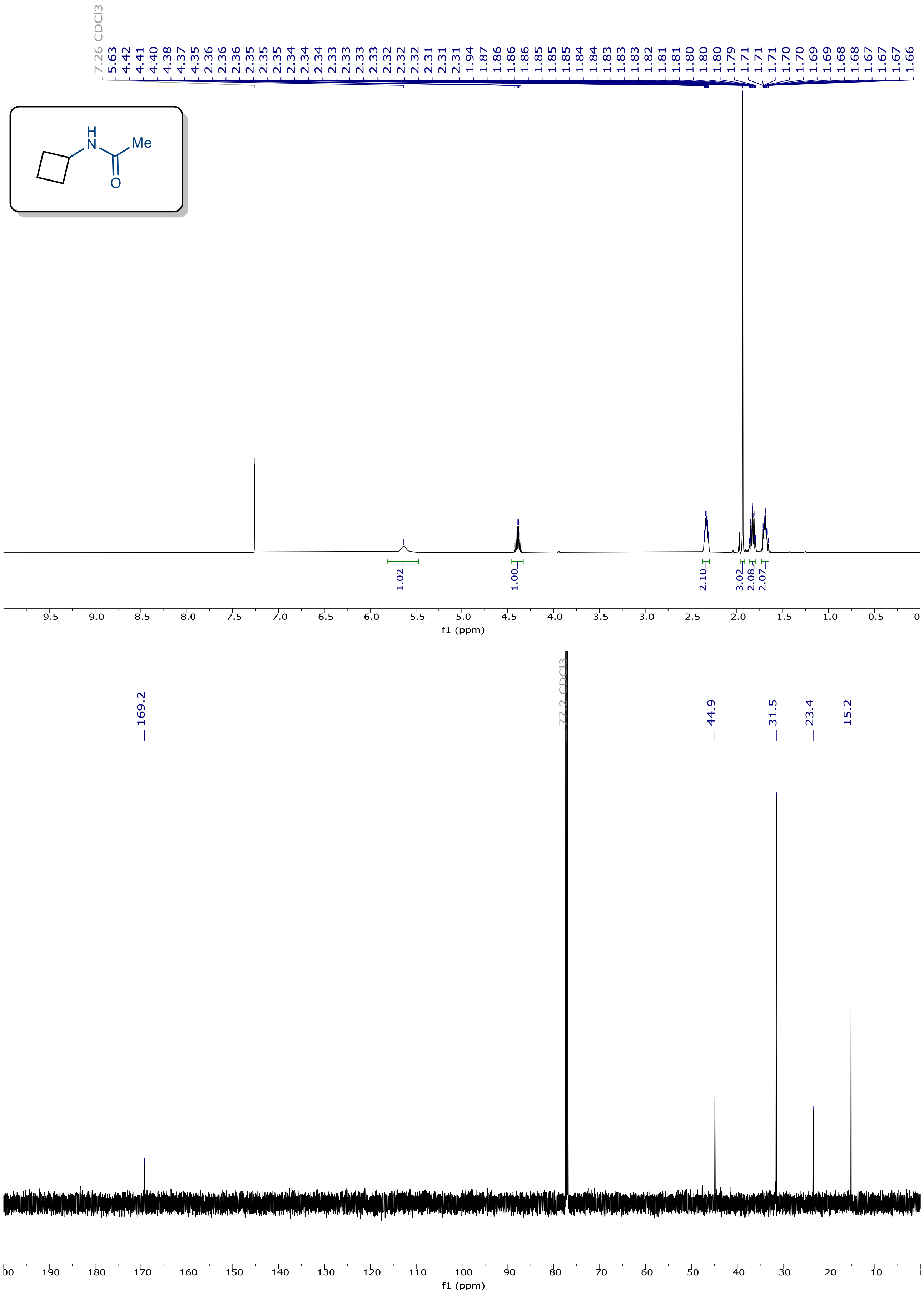

Supplementary Fig. 29 | ^{1}H (top) and ^{13}C (bottom) NMR spectra of **5.**

***N*-Cycloheptylacetamide (6)**

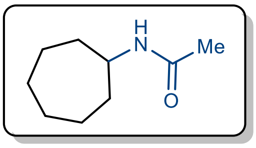

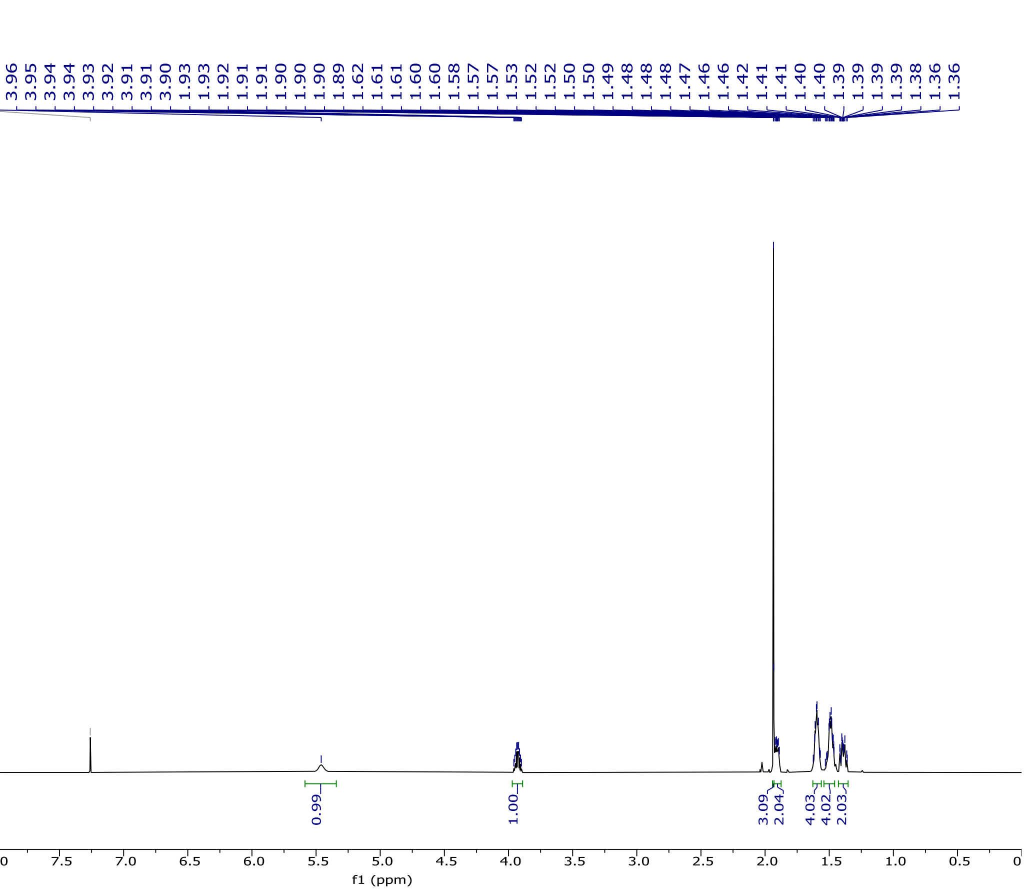

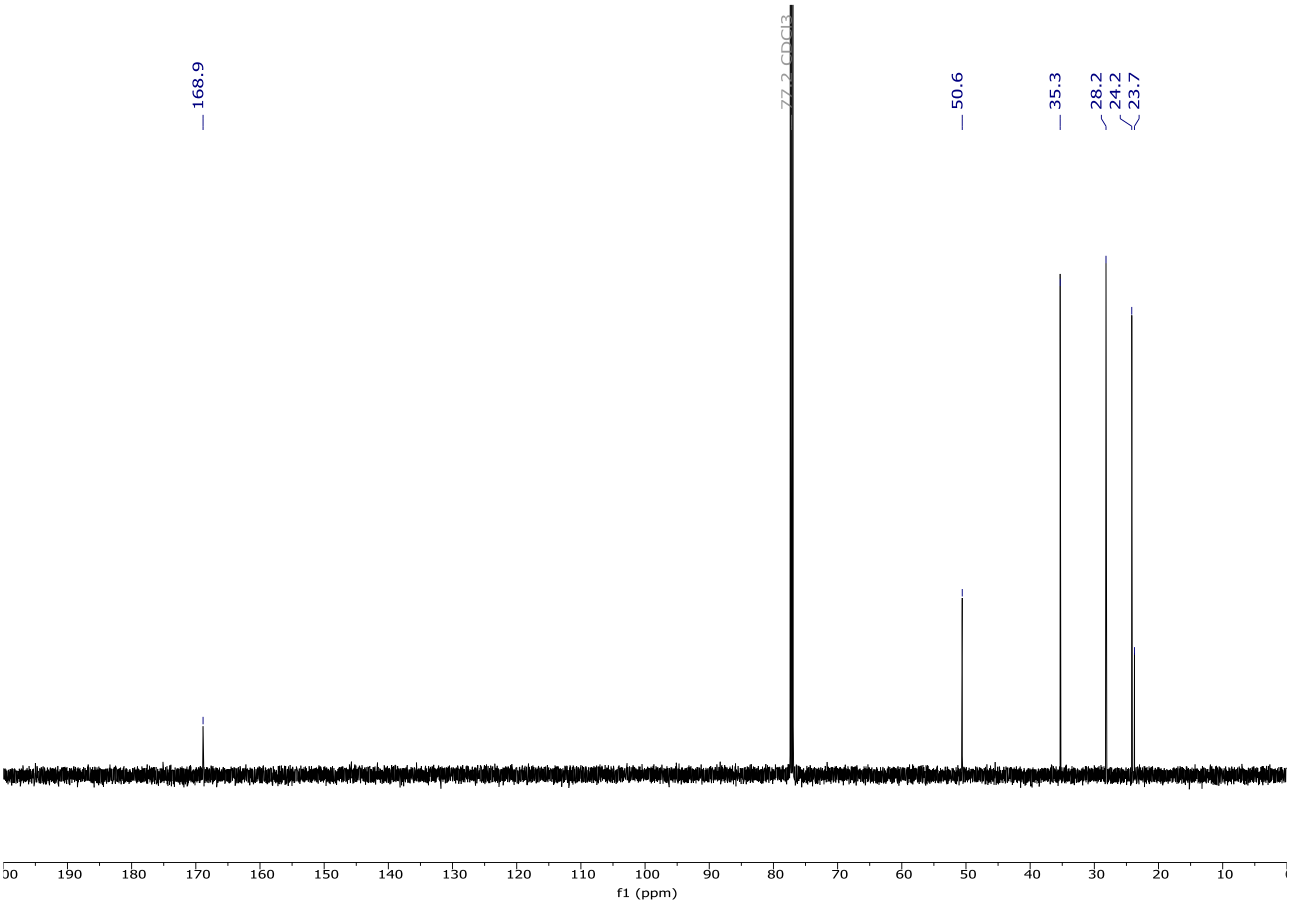

Supplementary Fig. 30 | ^{1}H (top) and ^{13}C (bottom) NMR spectra of **6.**

N-(Bicyclo[2.2.1]heptan-2-yl)acetamide (7)

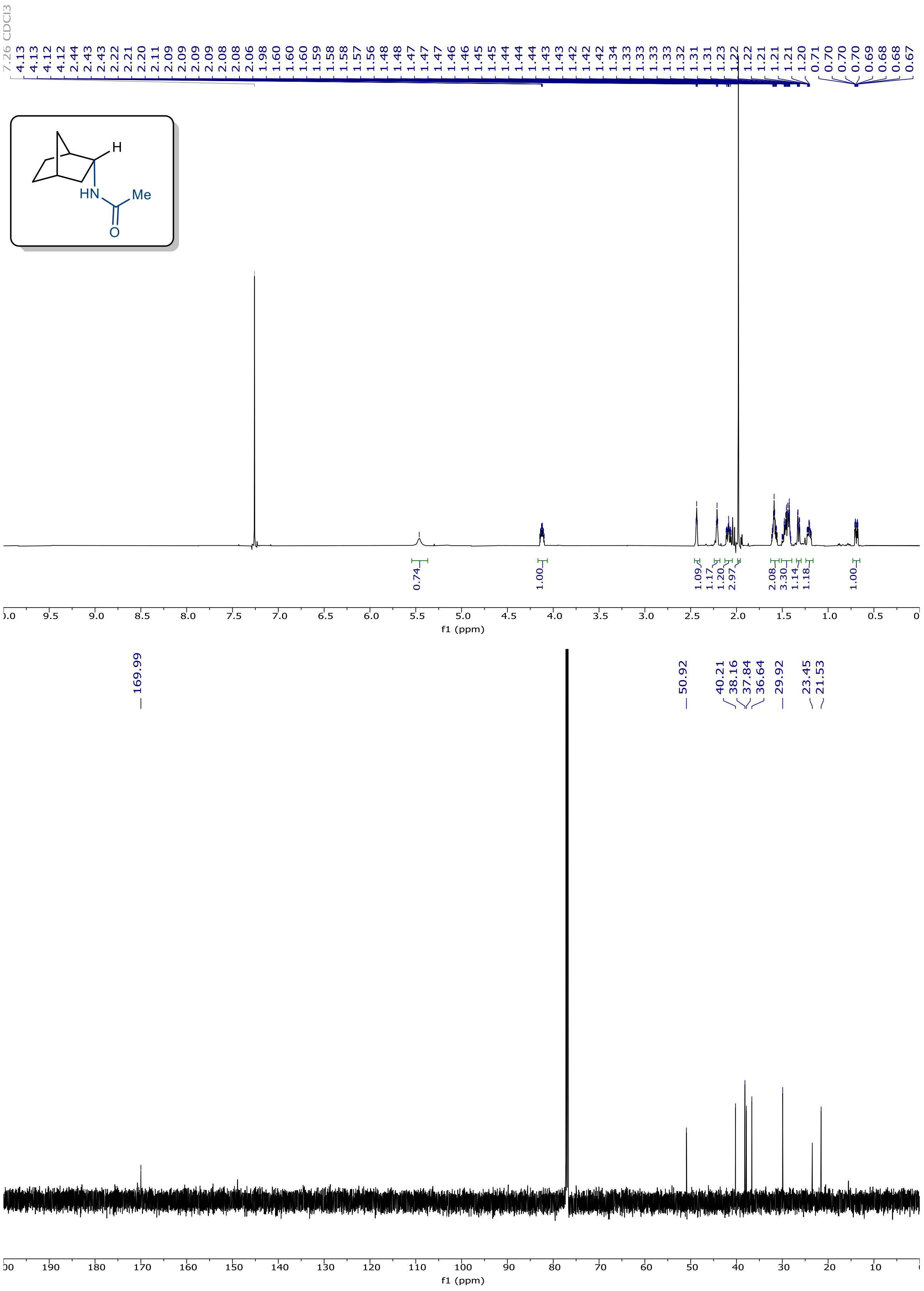

Supplementary Fig. 31 | ^{1}H (top) and ^{13}C (bottom) NMR spectra of **7.**

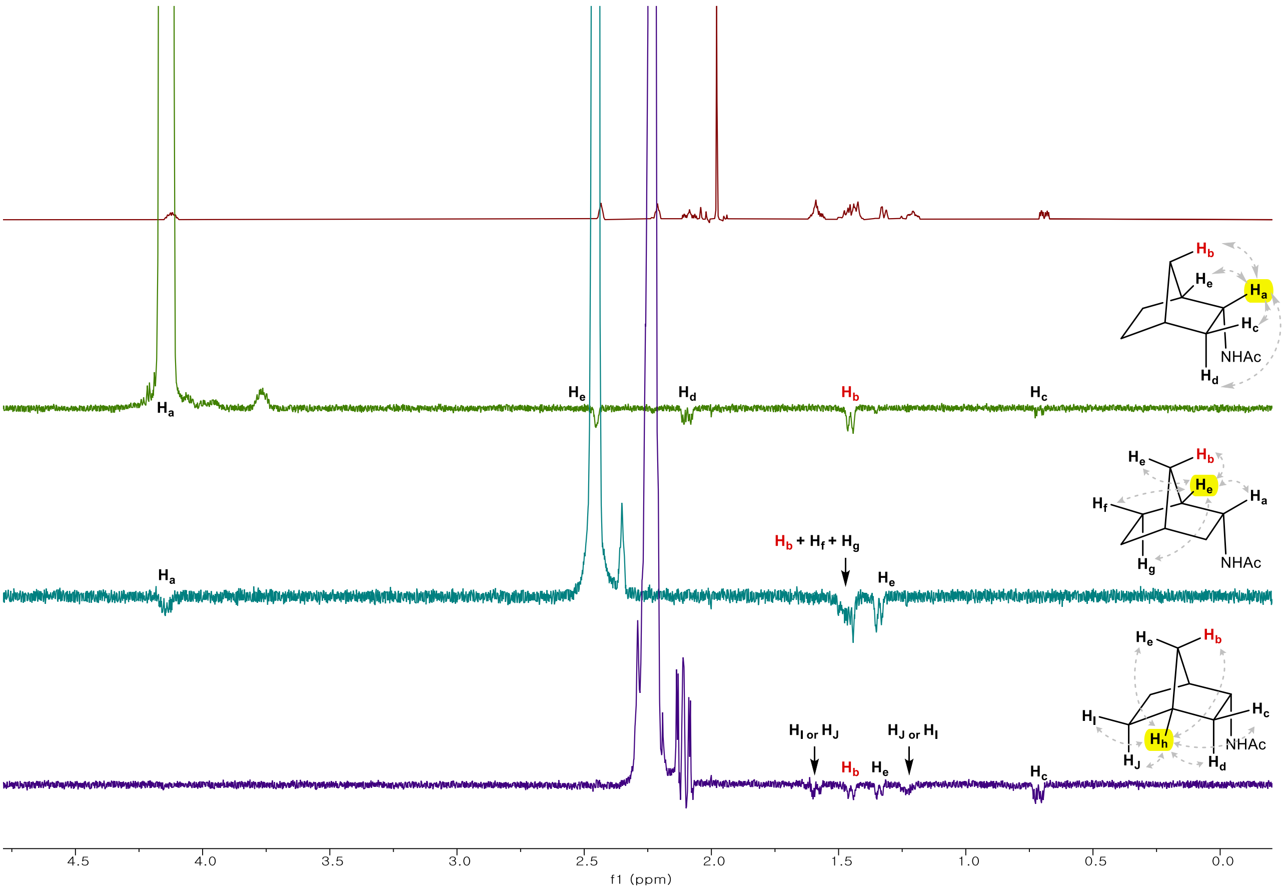

Supplementary Fig. 32 | 1D-NOE experiment of **7.**

N-(4,4-Difluorocyclohexyl)acetamide (8)

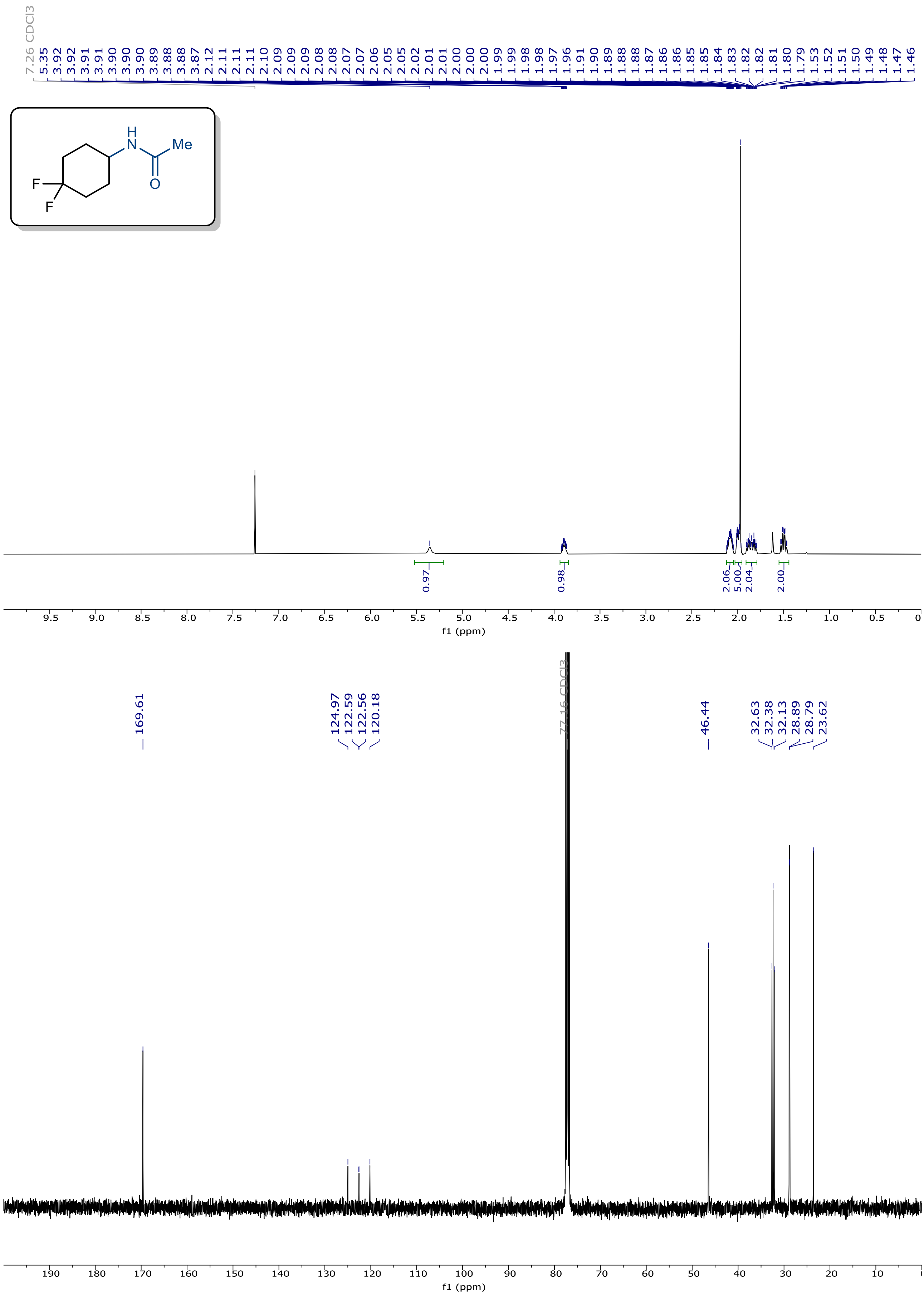

Supplementary Fig. 33 | ^{1}H (top) and ^{13}C (bottom) NMR spectra of **8.**

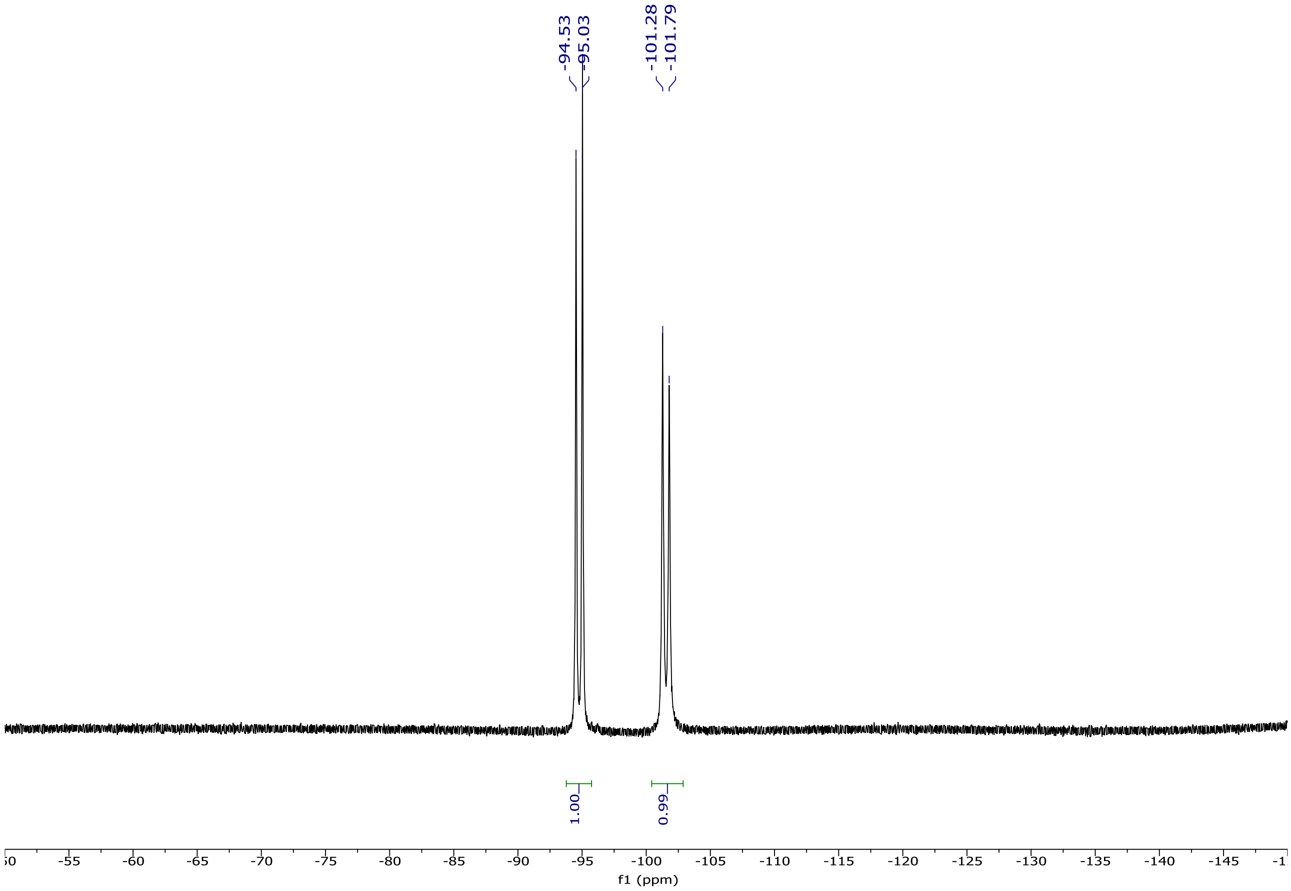

Supplementary Fig. 34 | ^{19}F NMR spectra of **8.**

***N*-(1,2,3,4-Tetrahydronaphthalen-2-yl)acetamide (9)**

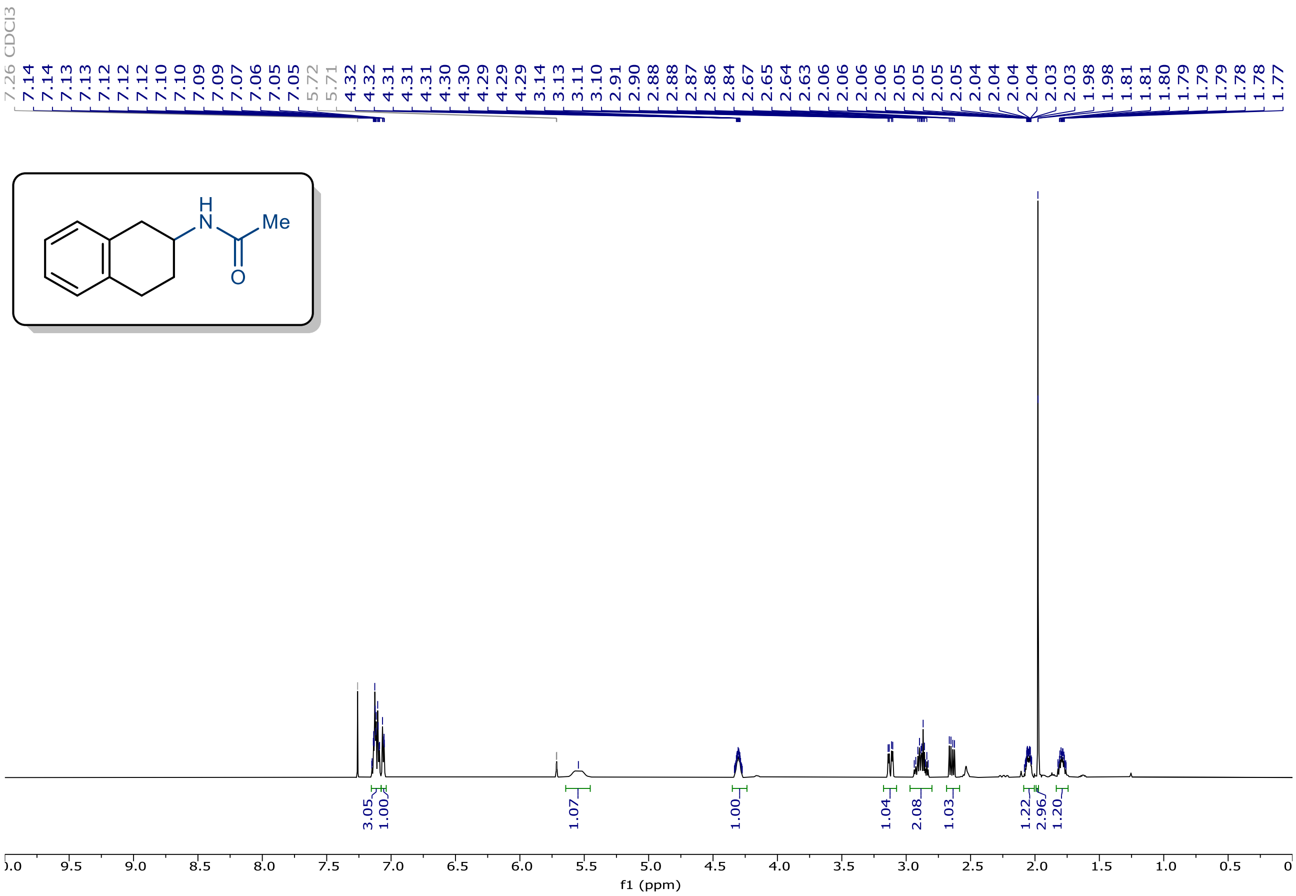

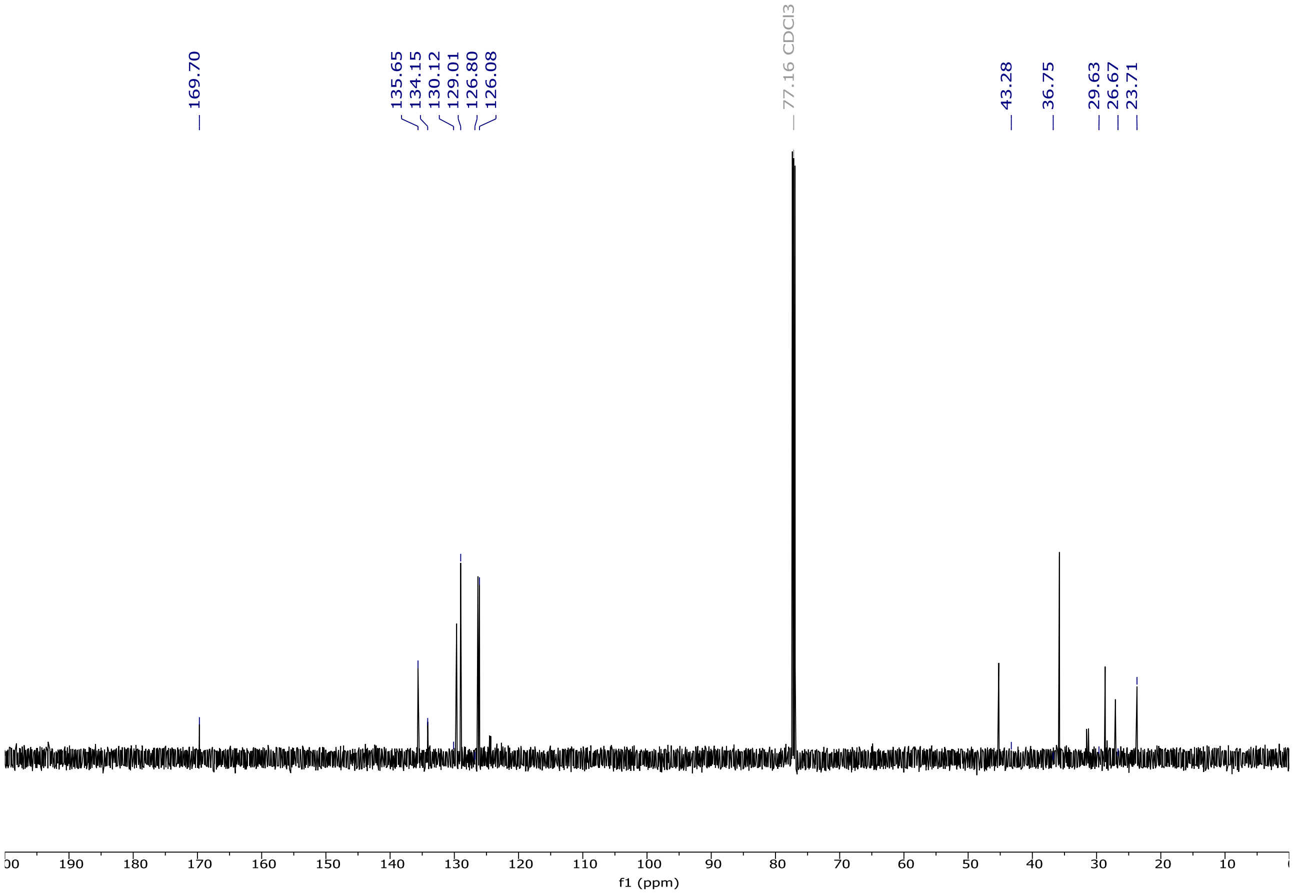

Supplementary Fig. 35 | ^{1}H (top) and ^{13}C (bottom) NMR spectra of **9.**

***N*-(2,3-Dihydro-1H-inden-2-yl)acetamide (10)**

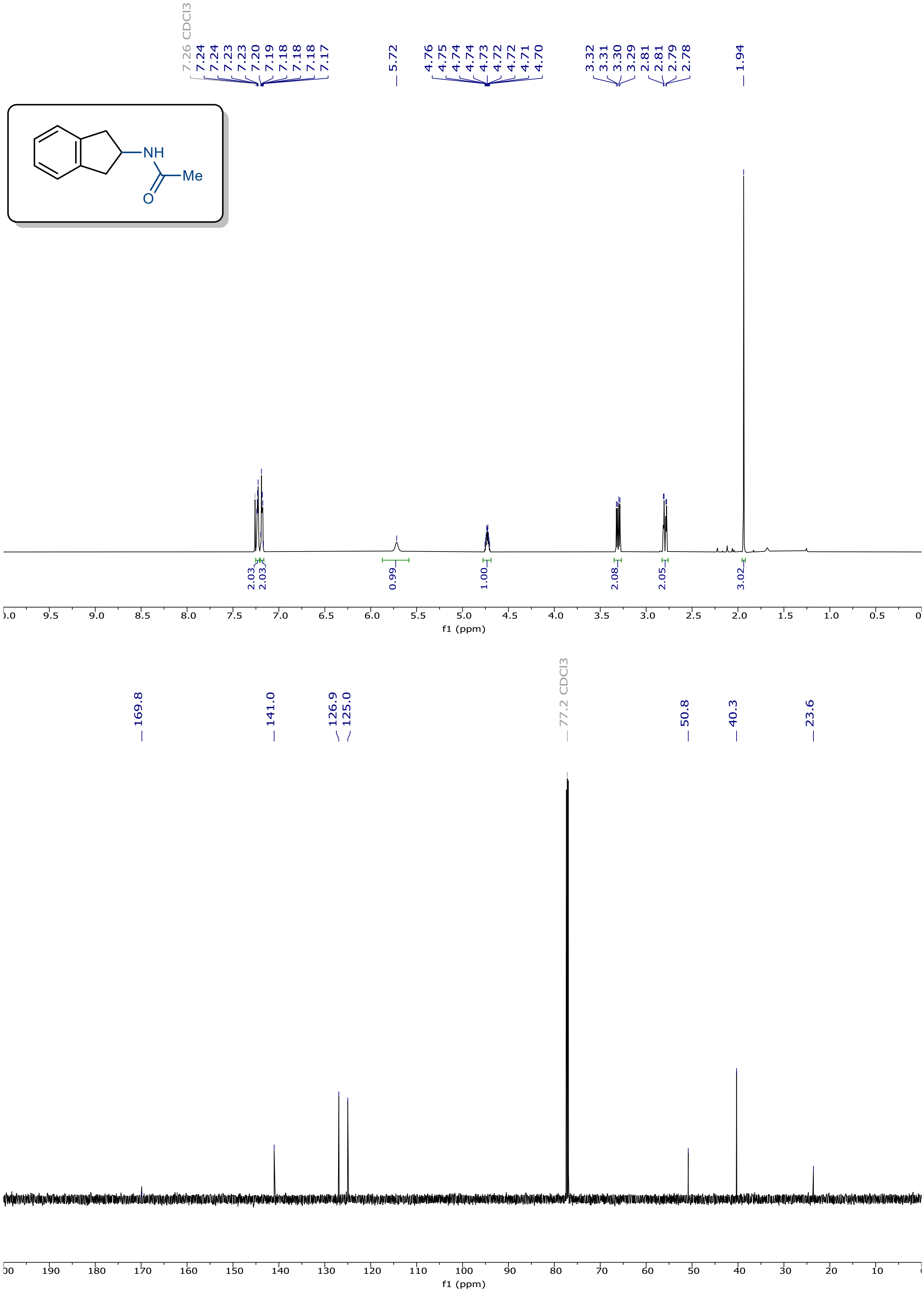

Supplementary Fig. 36 | ^{1}H (top) and ^{13}C (bottom) NMR spectra of **10.**

***tert*-Butyl-4-acetamidopiperidine-1-carboxylate (11)**

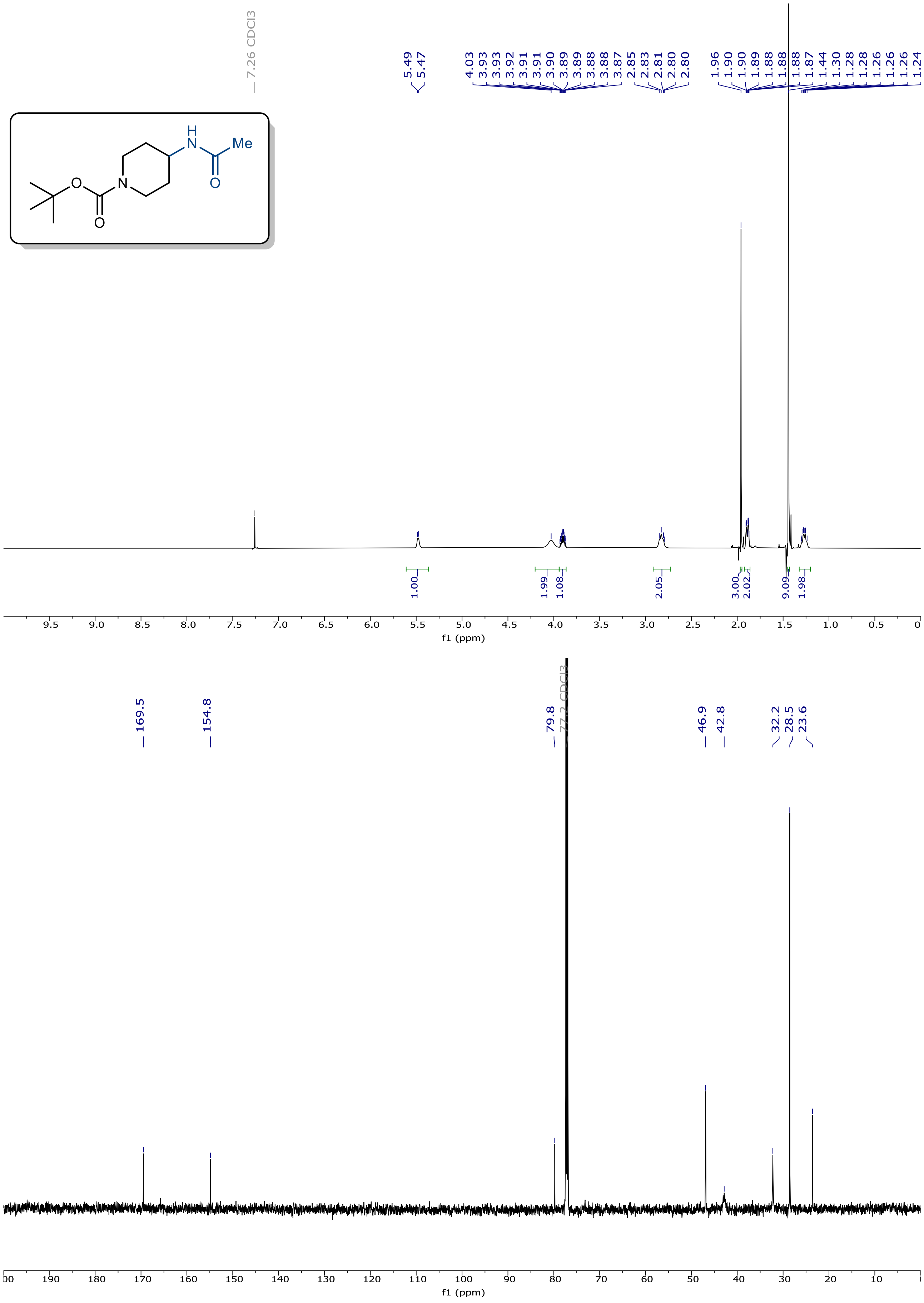

Supplementary Fig. 37 | ^{1}H (top) and ^{13}C (bottom) NMR spectra of **11.**

N-(1,4-Diphenylbutan-2-yl)acetamide (12)

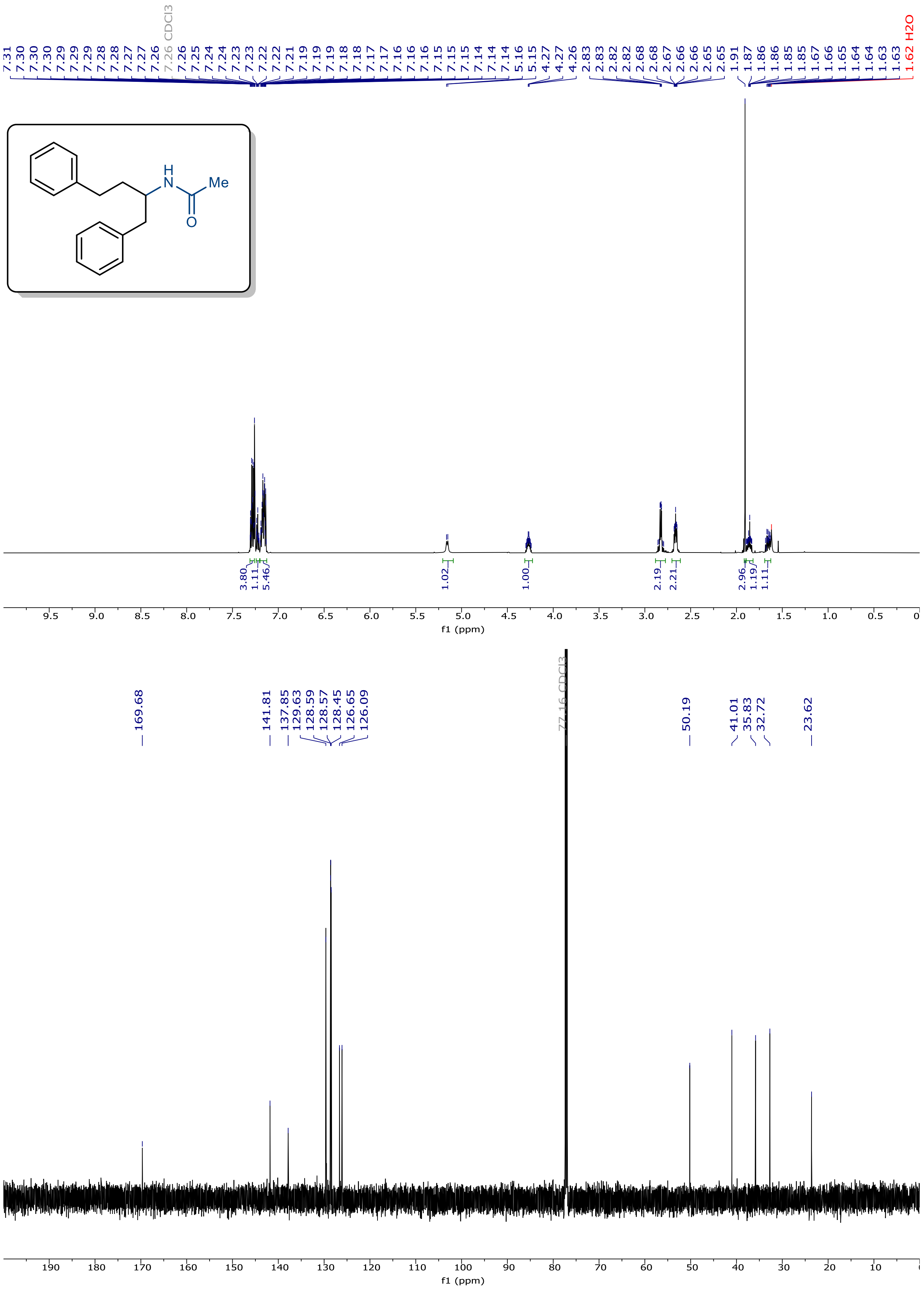

Supplementary Fig. 38 | ^{1}H (top) and ^{13}C (bottom) NMR spectra of **12.**

***N*-(Pentan-3-yl)acetamide (13)**

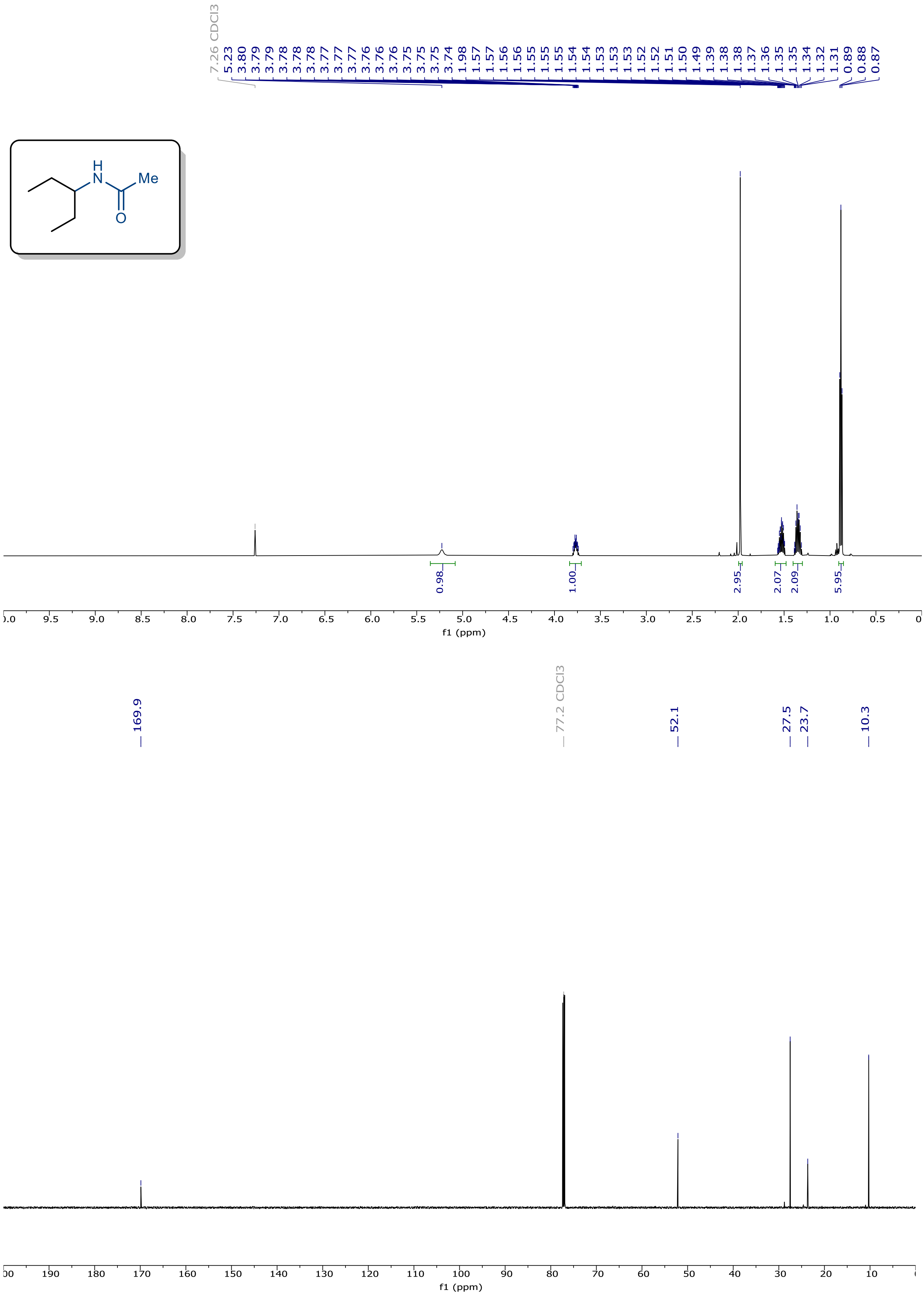

Supplementary Fig. 39 | ^{1}H (top) and ^{13}C (bottom) NMR spectra of **13.**

N-(Hex-5-yn-3-yl)acetamide (14)

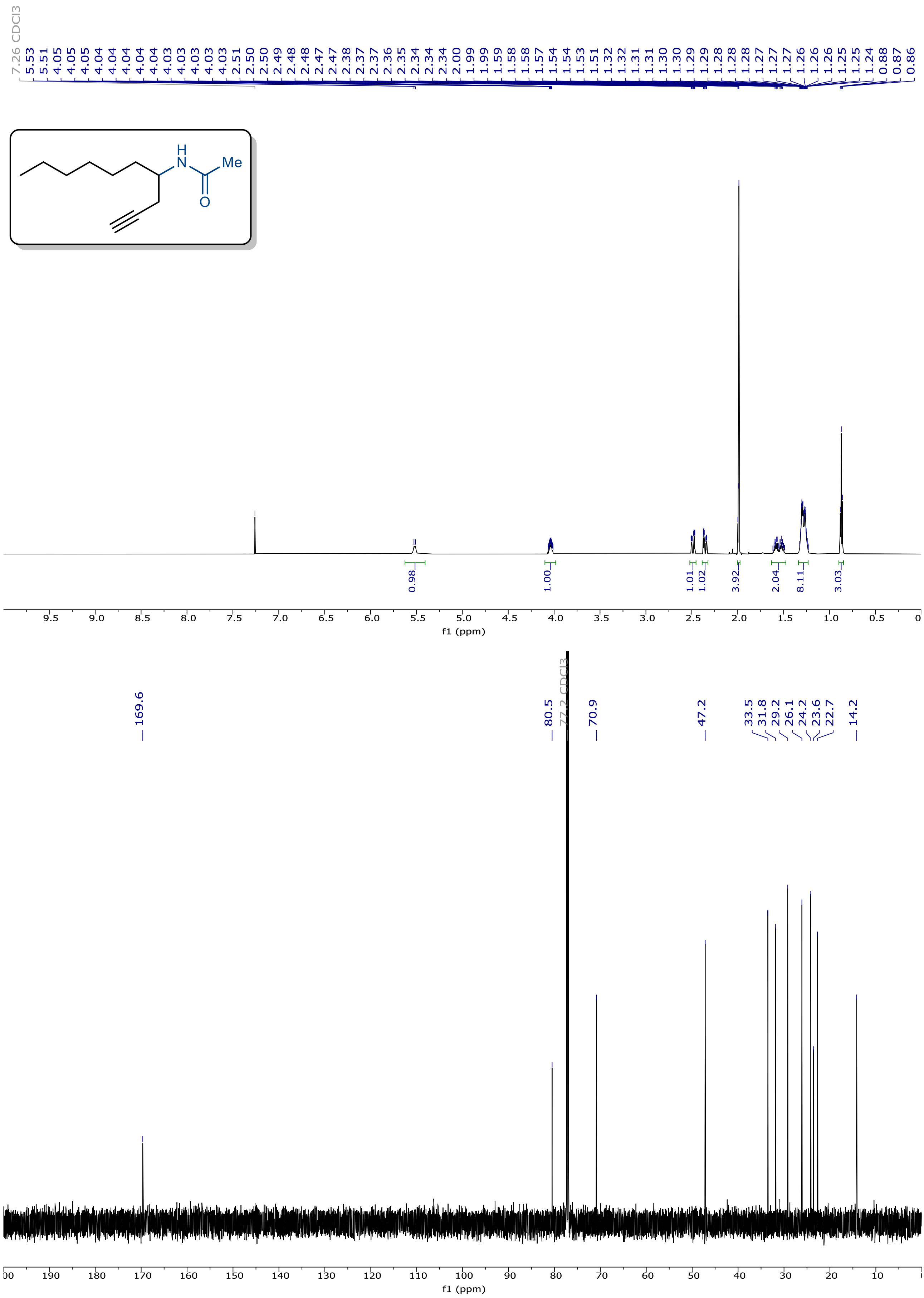

Supplementary Fig. 40 | ^{1}H (top) and ^{13}C (bottom) NMR spectra of **14.**

***N*-Benzylacetamide (15)**

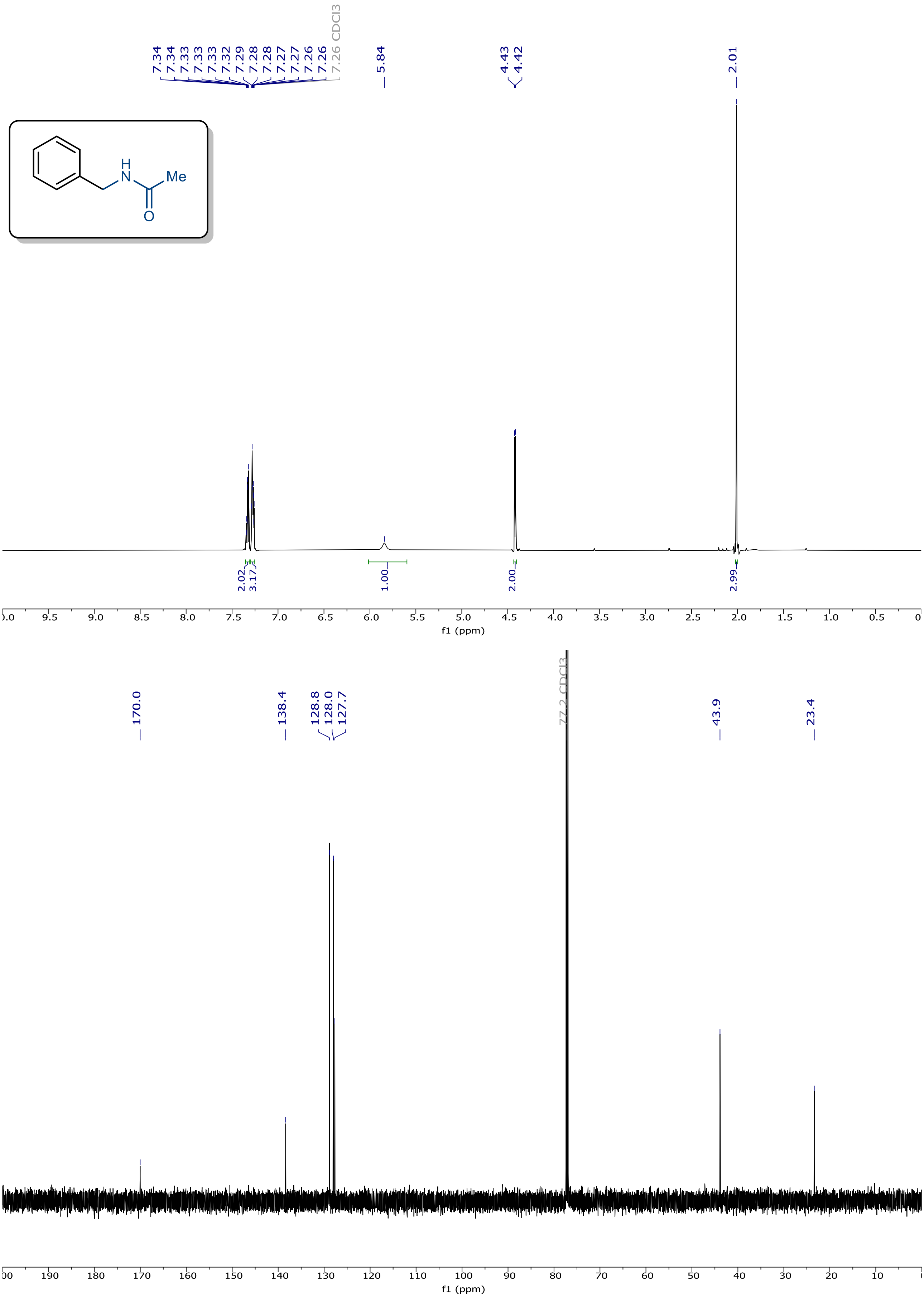

Supplementary Fig. 41 | ^{1}H (top) and ^{13}C (bottom) NMR spectra of **15.**

***N*-Phenethylacetamide (16)**

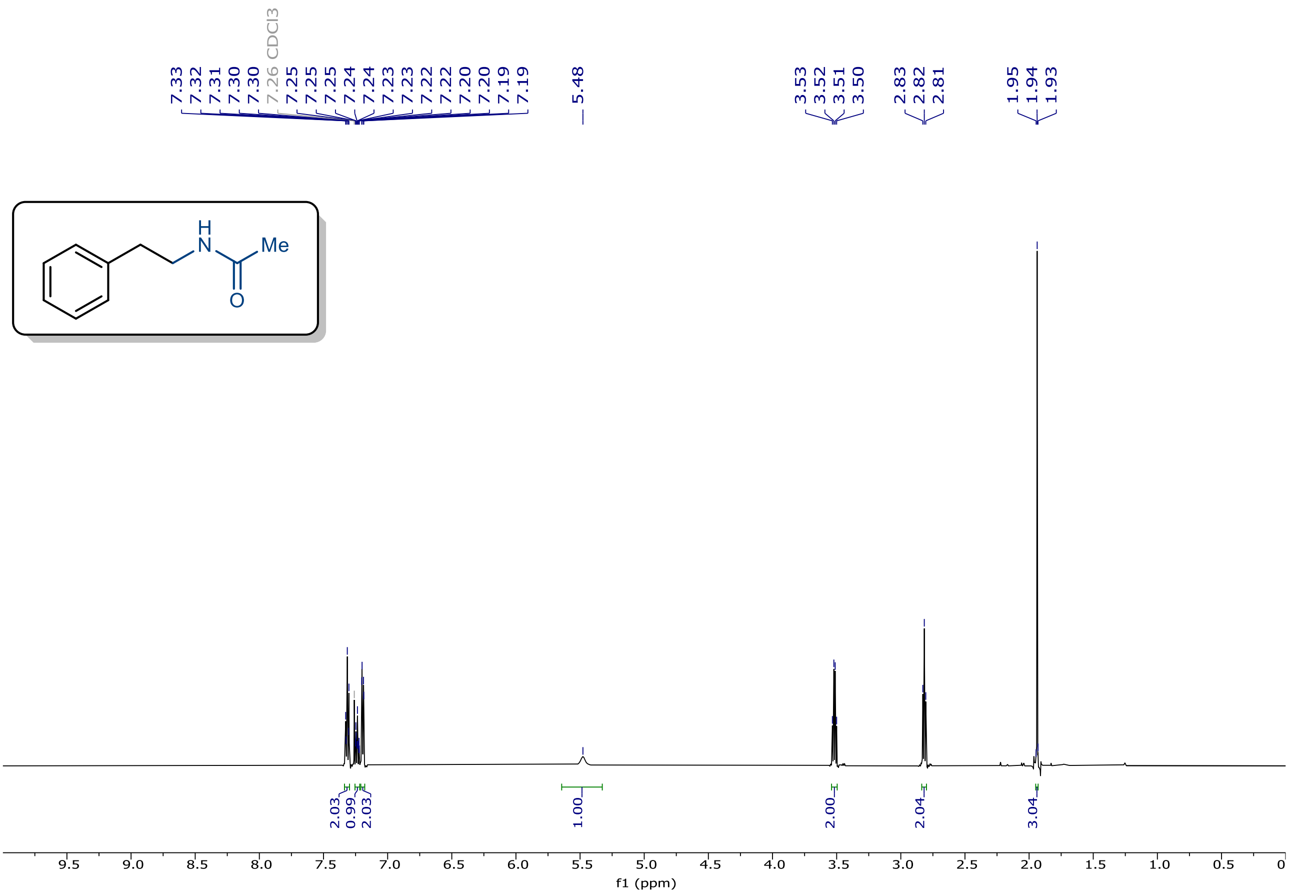

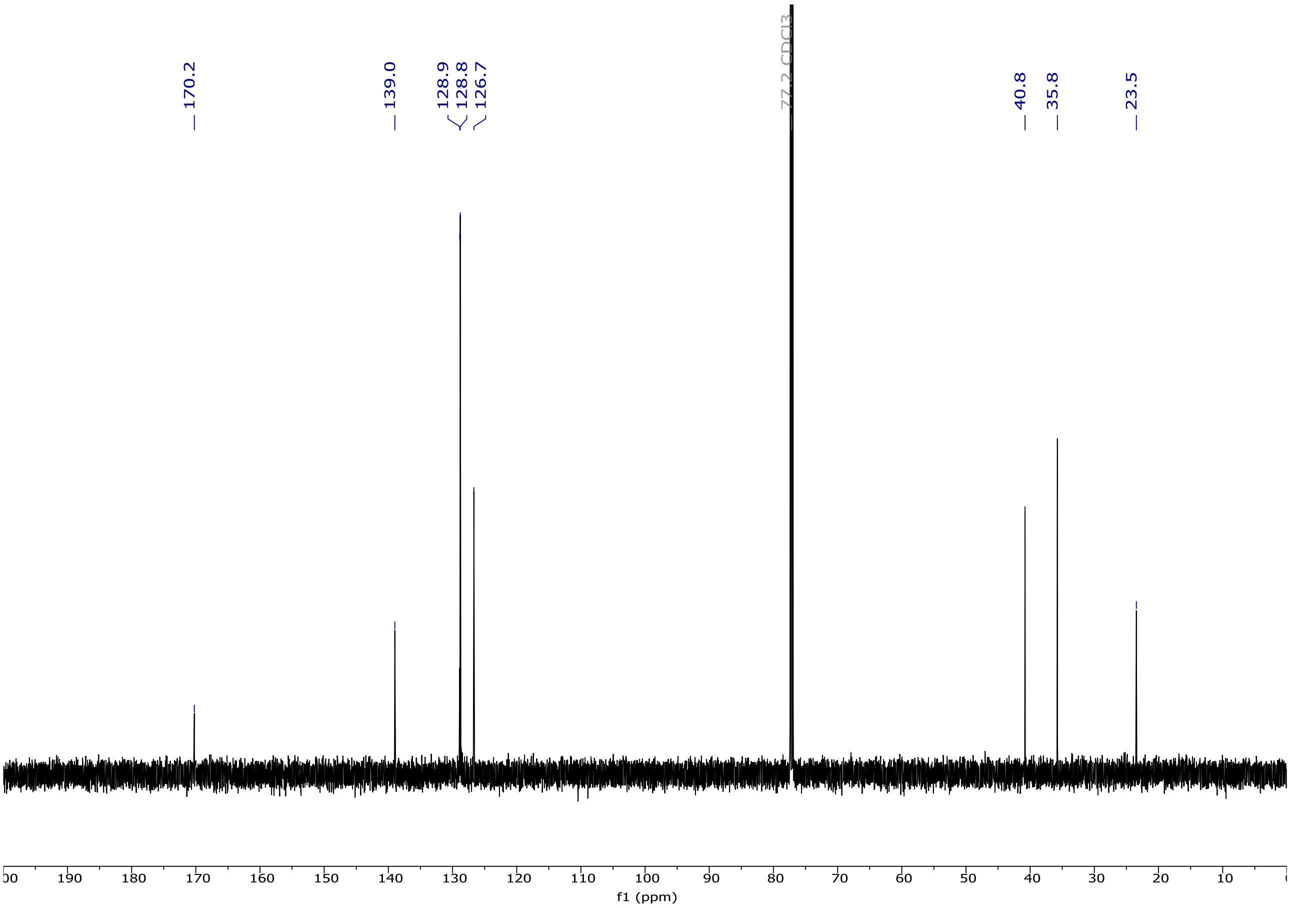

Supplementary Fig. 42 | ^{1}H (top) and ^{13}C (bottom) NMR spectra of **16.**

***N*-(3-Phenylpropyl)acetamide (17)**

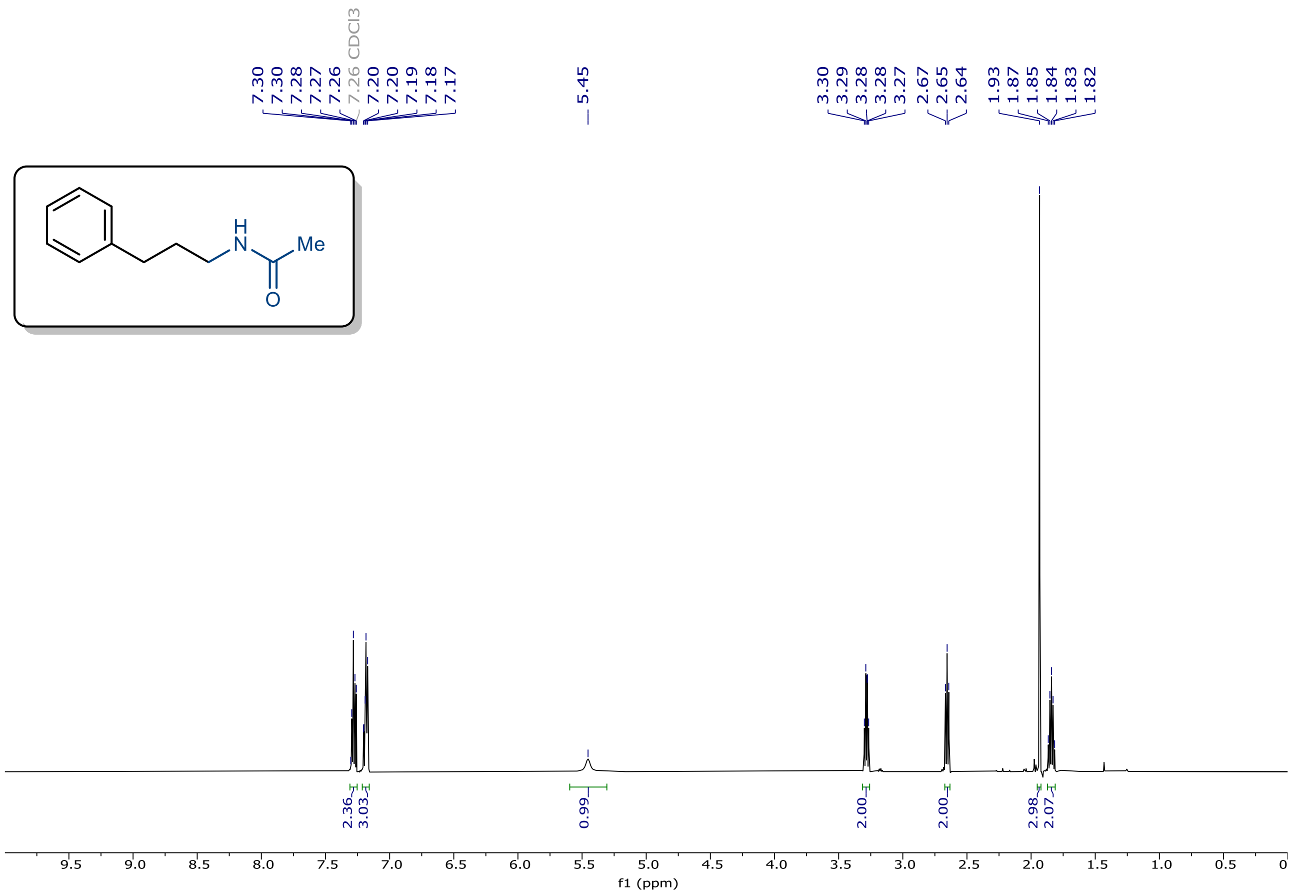

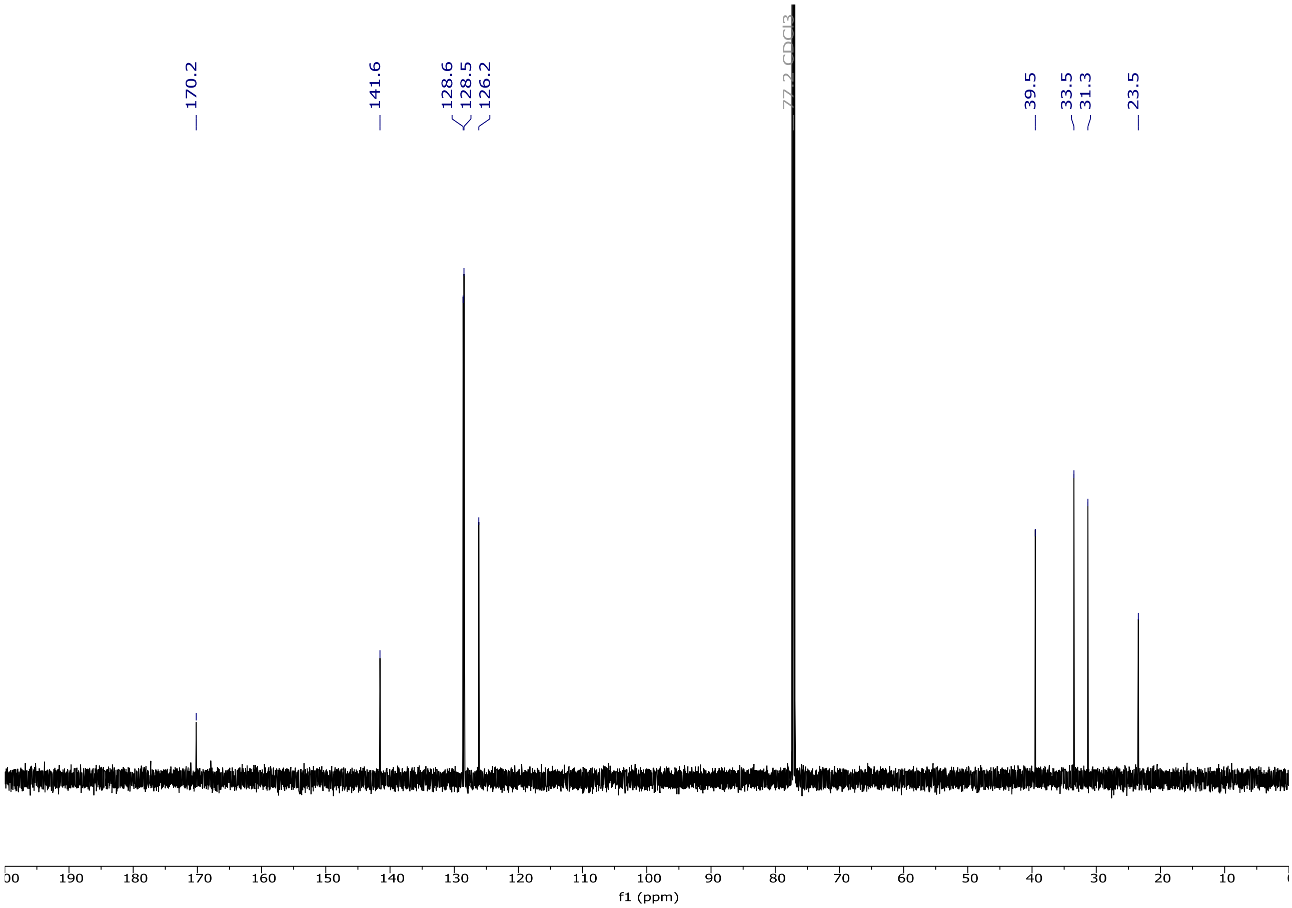

Supplementary Fig. 43 | ^{1}H (top) and ^{13}C (bottom) NMR spectra of **17.**

N-Neopentylacetamide (18)

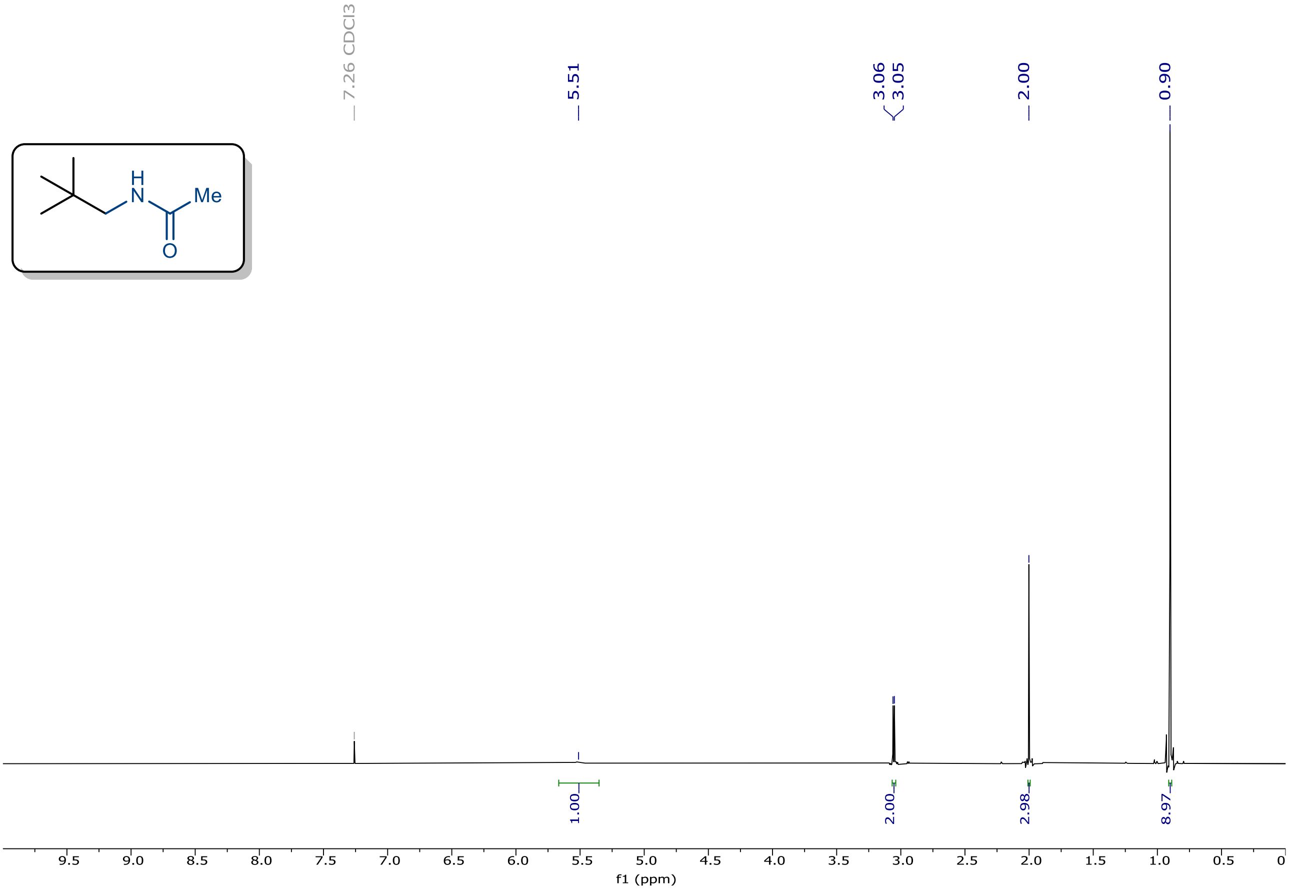

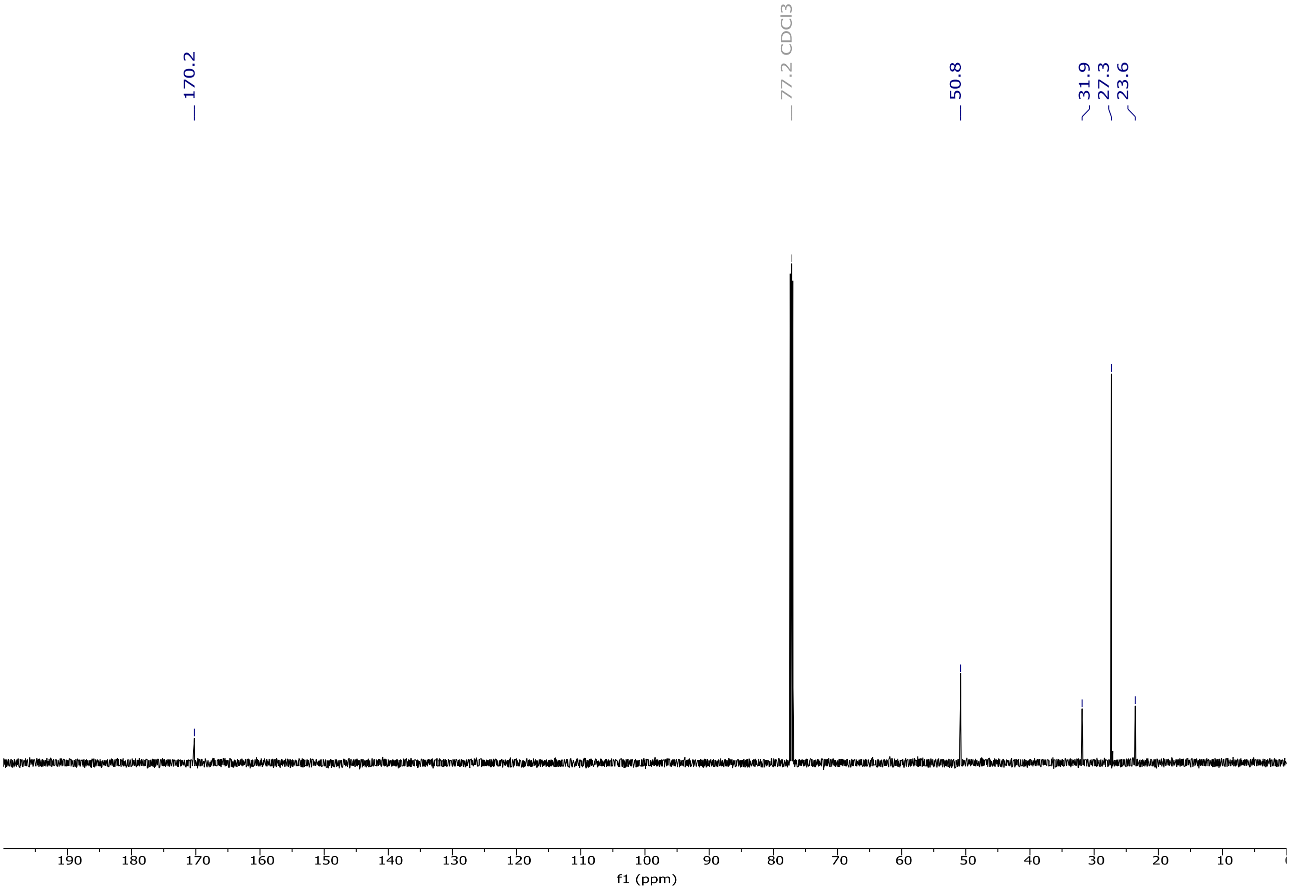

Supplementary Fig. 44 | ^{1}H (top) and ^{13}C (bottom) NMR spectra of **18.**

N-(5-Chloropentyl)acetamide (19)

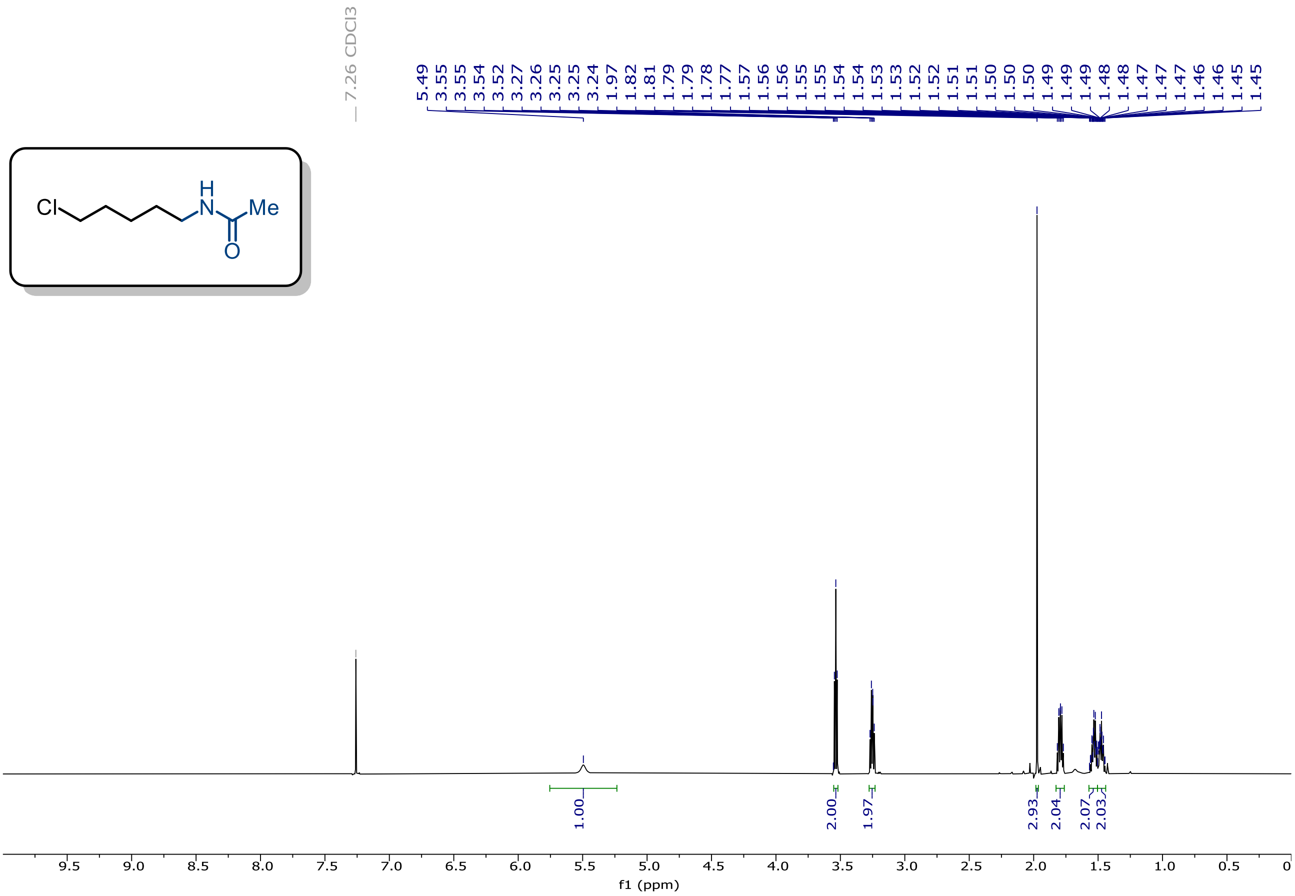

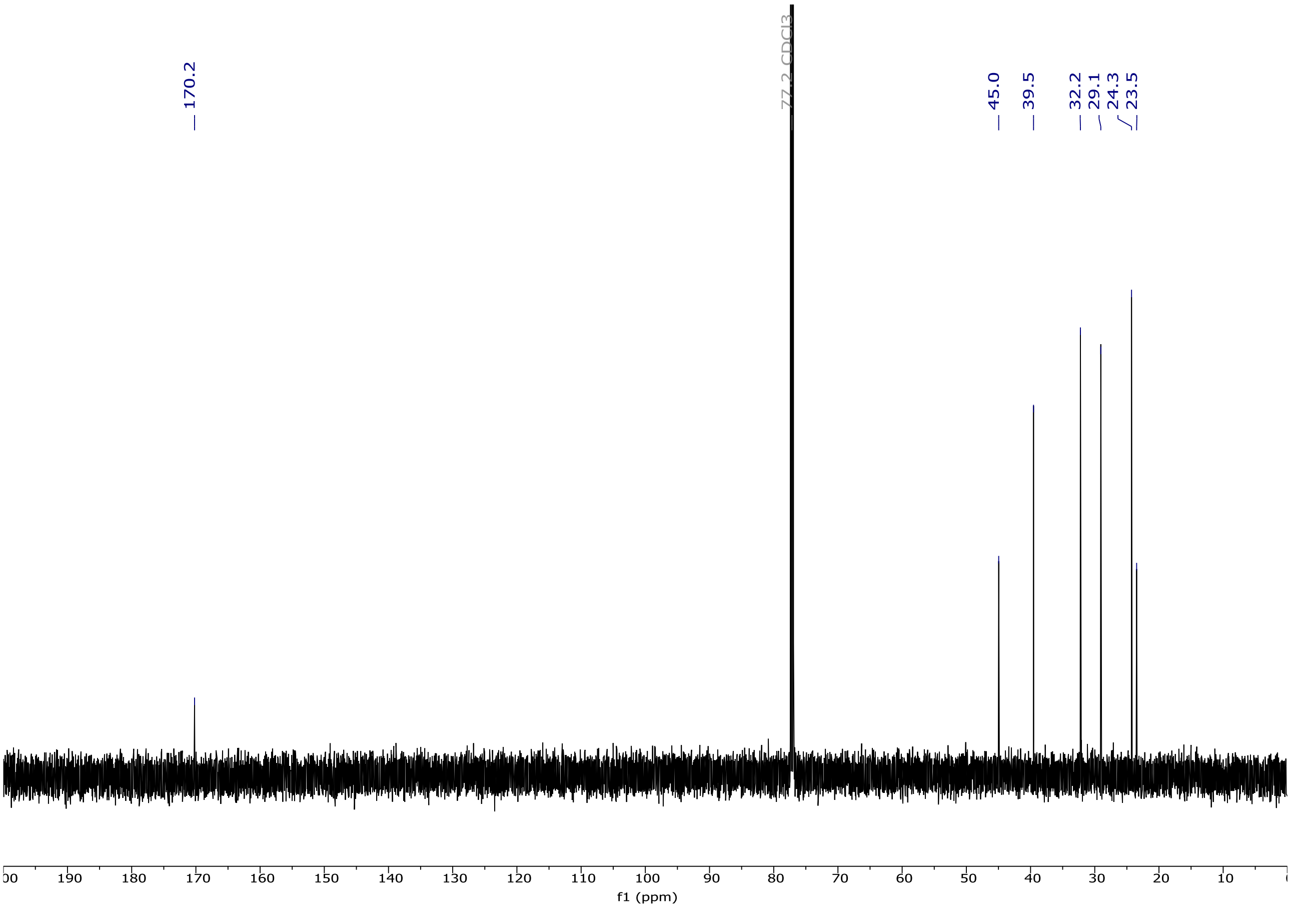

Supplementary Fig. 45 | ^{1}H (top) and ^{13}C (bottom) NMR spectra of **19.**

***N*-(Cyclopropylmethyl)acetamide (20)**

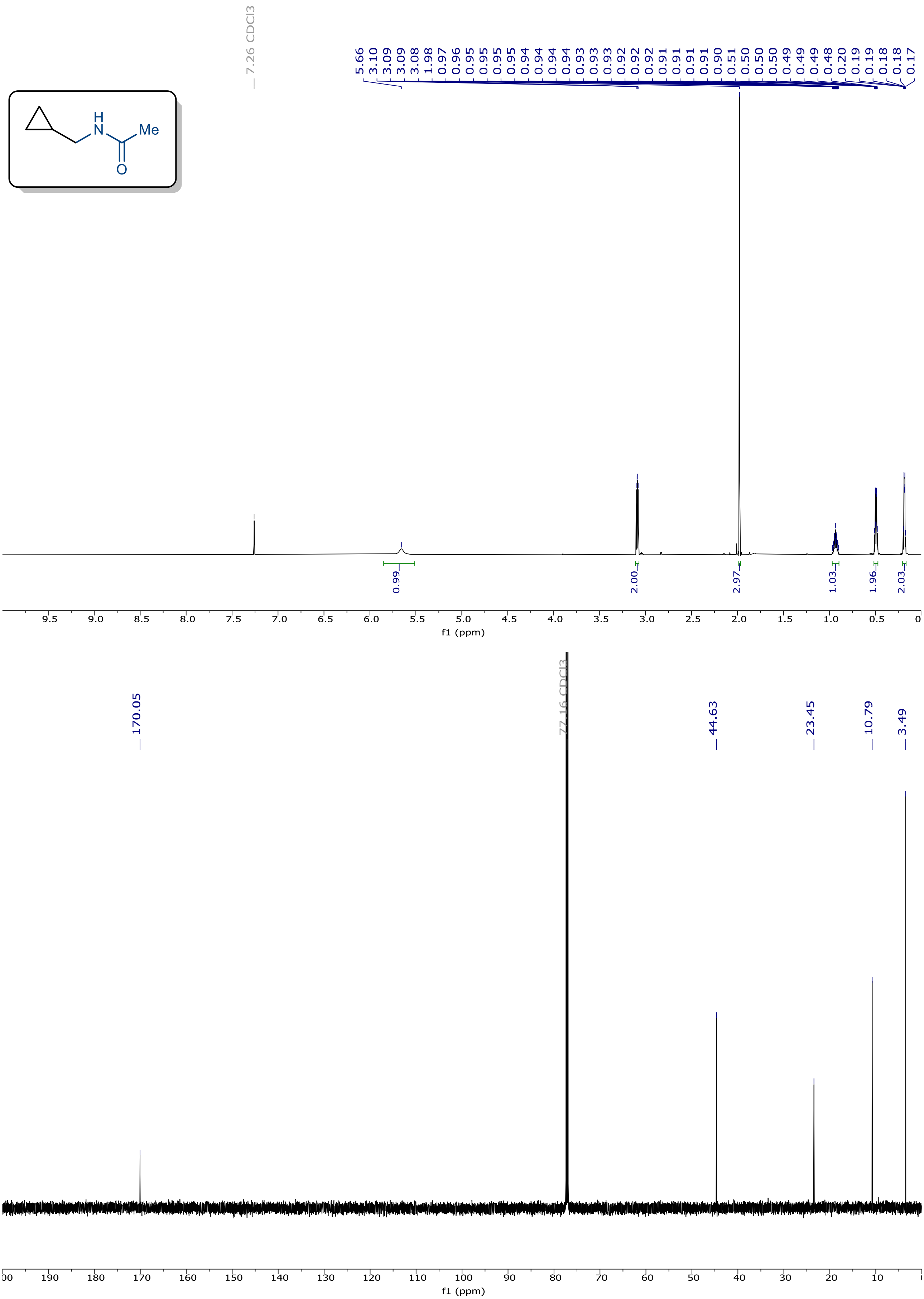

Supplementary Fig. 46 | ^{1}H (top) and ^{13}C (bottom) NMR spectra of **20.**

N,N′-(Pentane-1,5-diyl)diacetamide (21)

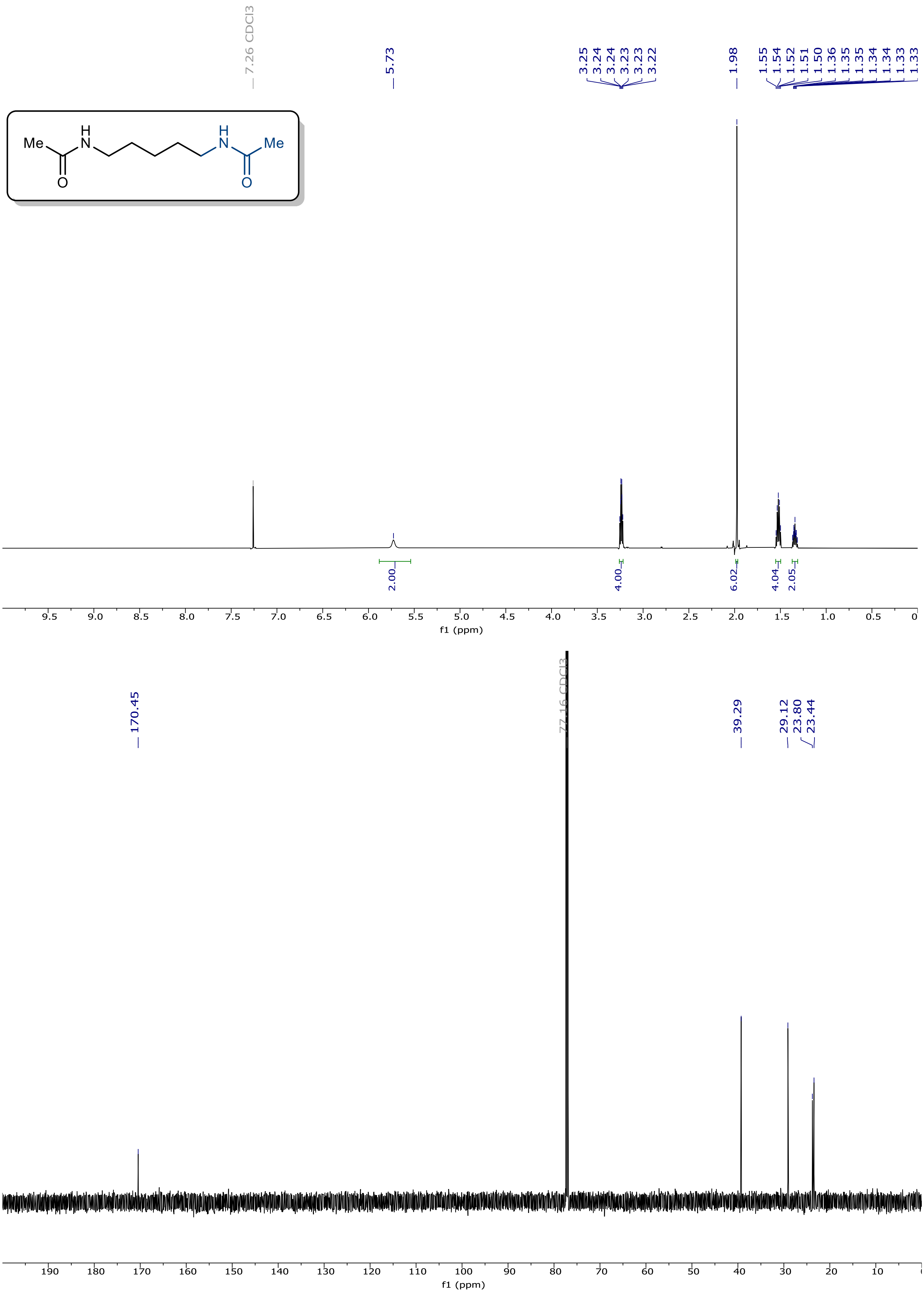

Supplementary Fig. 47 | ^{1}H (top) and ^{13}C (bottom) NMR spectra of **21.**

(*E*)-*N*-(Hept-2-en-1-yl)acetamide (22)

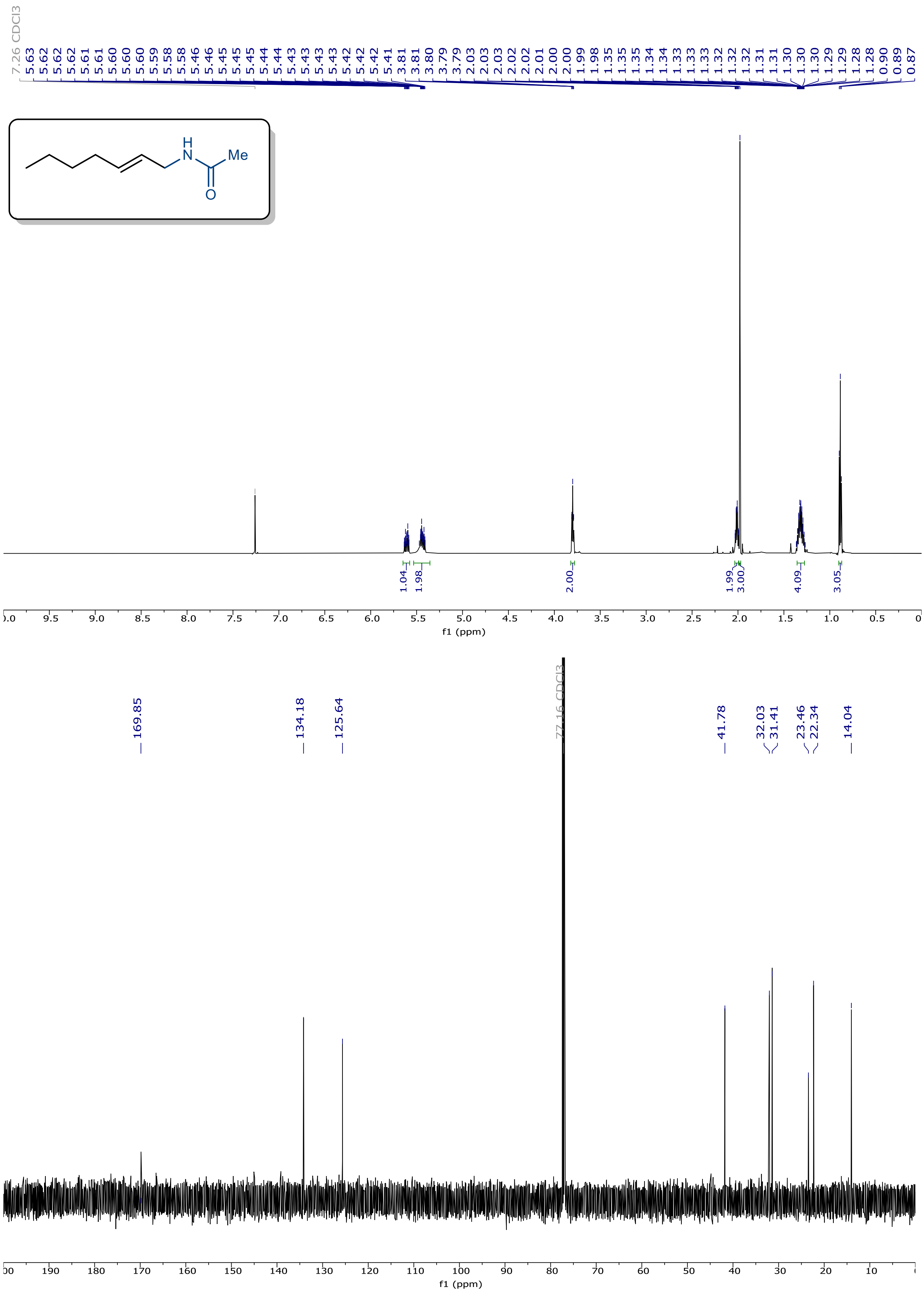

Supplementary Fig. 48 | ^{1}H (top) and ^{13}C (bottom) NMR spectra of **22.**

N-(Thiophen-3-ylmethyl)acetamide (23)

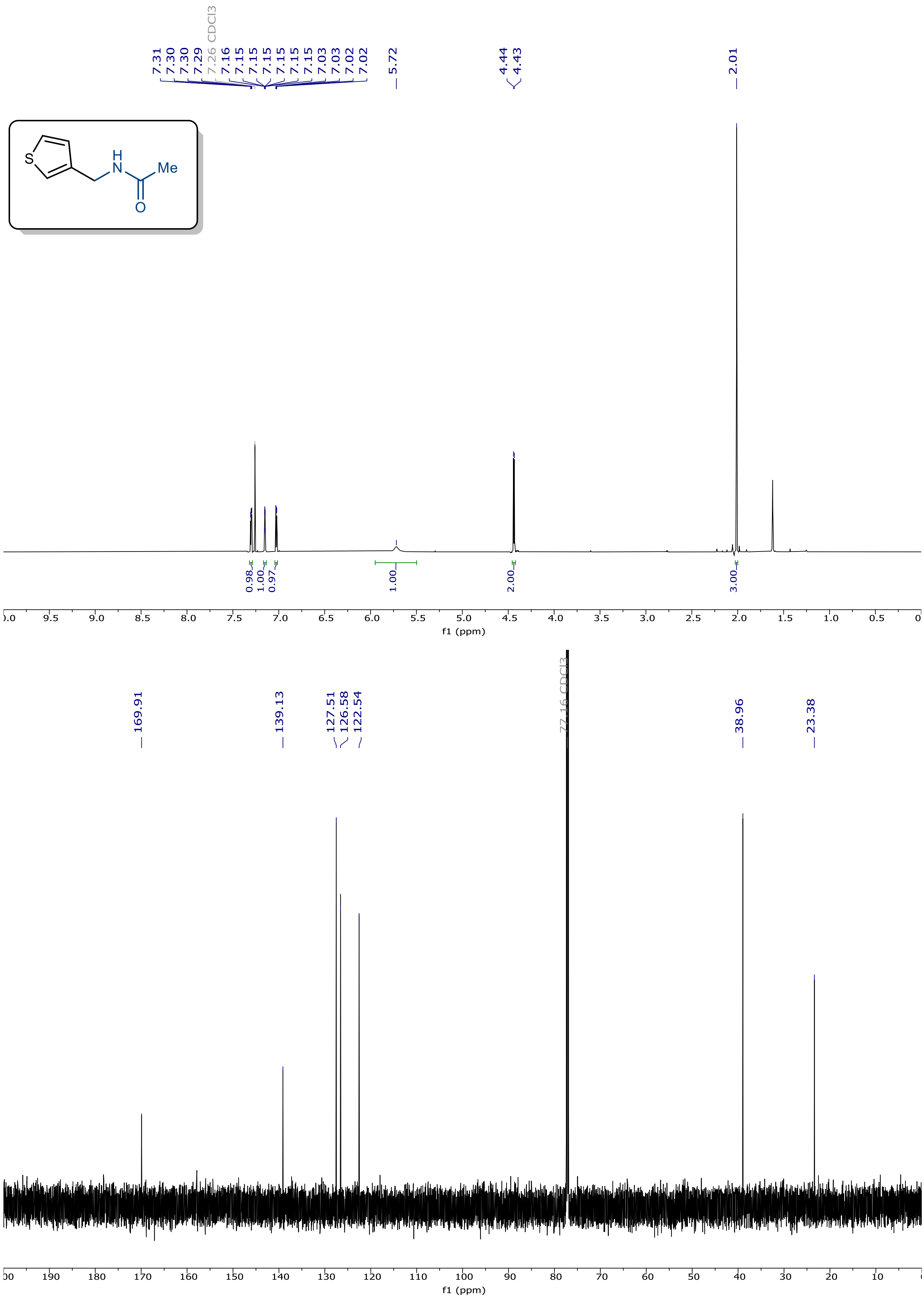

Supplementary Fig. 49 | ^{1}H (top) and ^{13}C (bottom) NMR spectra of **23.**

***N*-{3-(Benzyloxy)propyl}acetamide (24)**

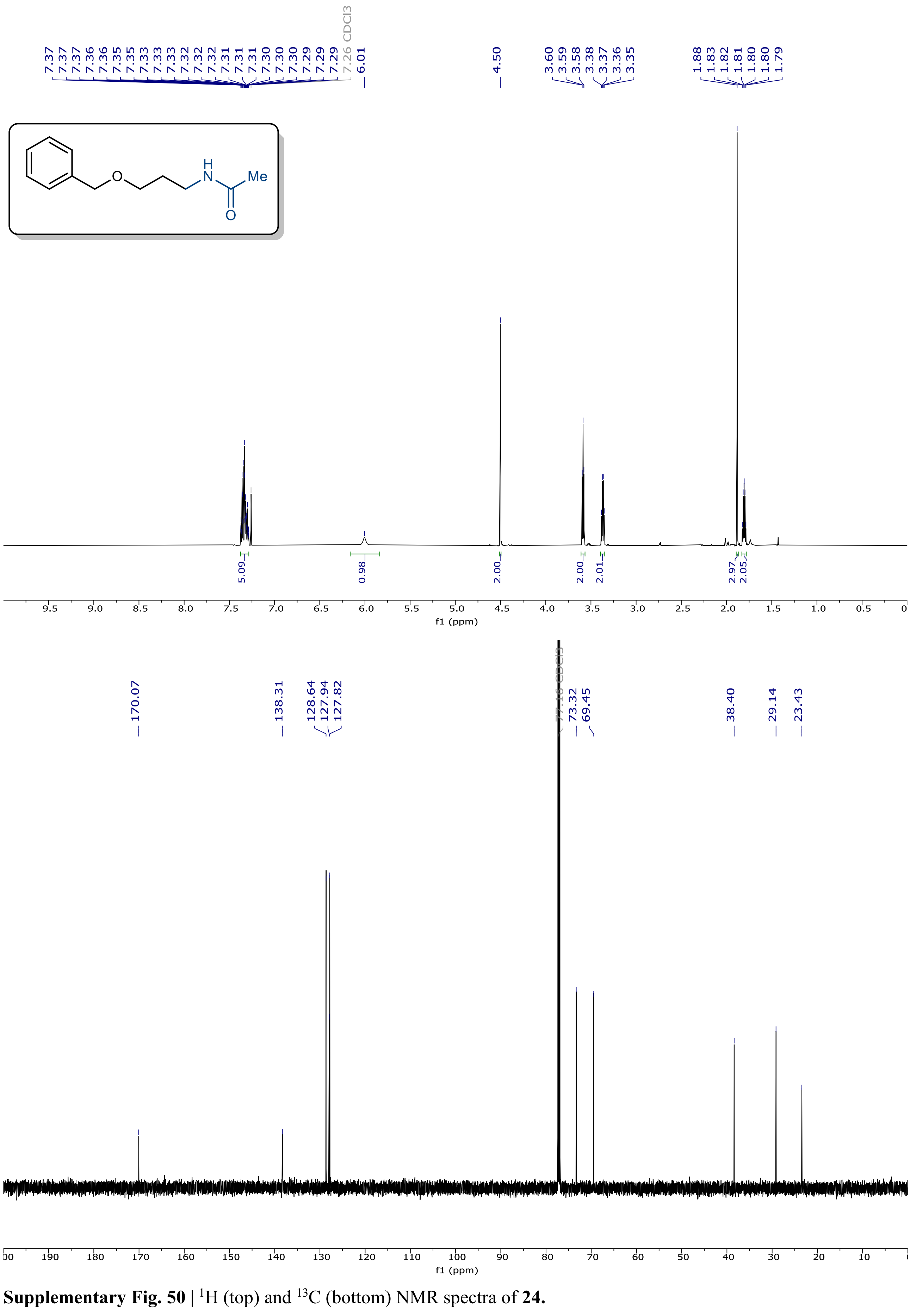

Supplementary Fig. 50 | ^{1}H (top) and ^{13}C (bottom) NMR spectra of **24.**

N-{3-(1,3-Dioxoisoindolin-2-yl)propyl}acetamide (25)

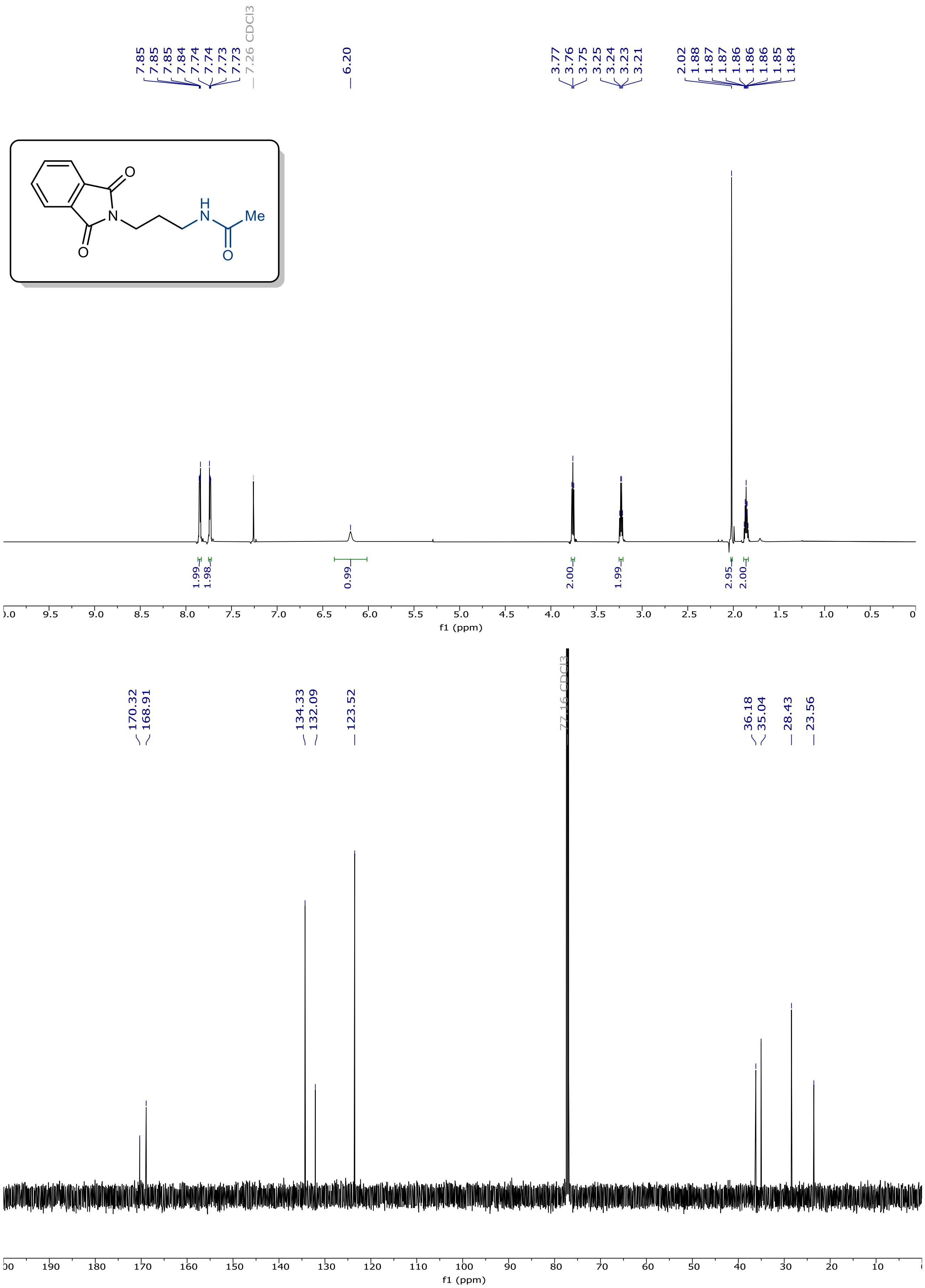

Supplementary Fig. 51 | ^{1}H (top) and ^{13}C (bottom) NMR spectra of **25.**

N-(2-Phenylpropan-2-yl)acetamide (26)

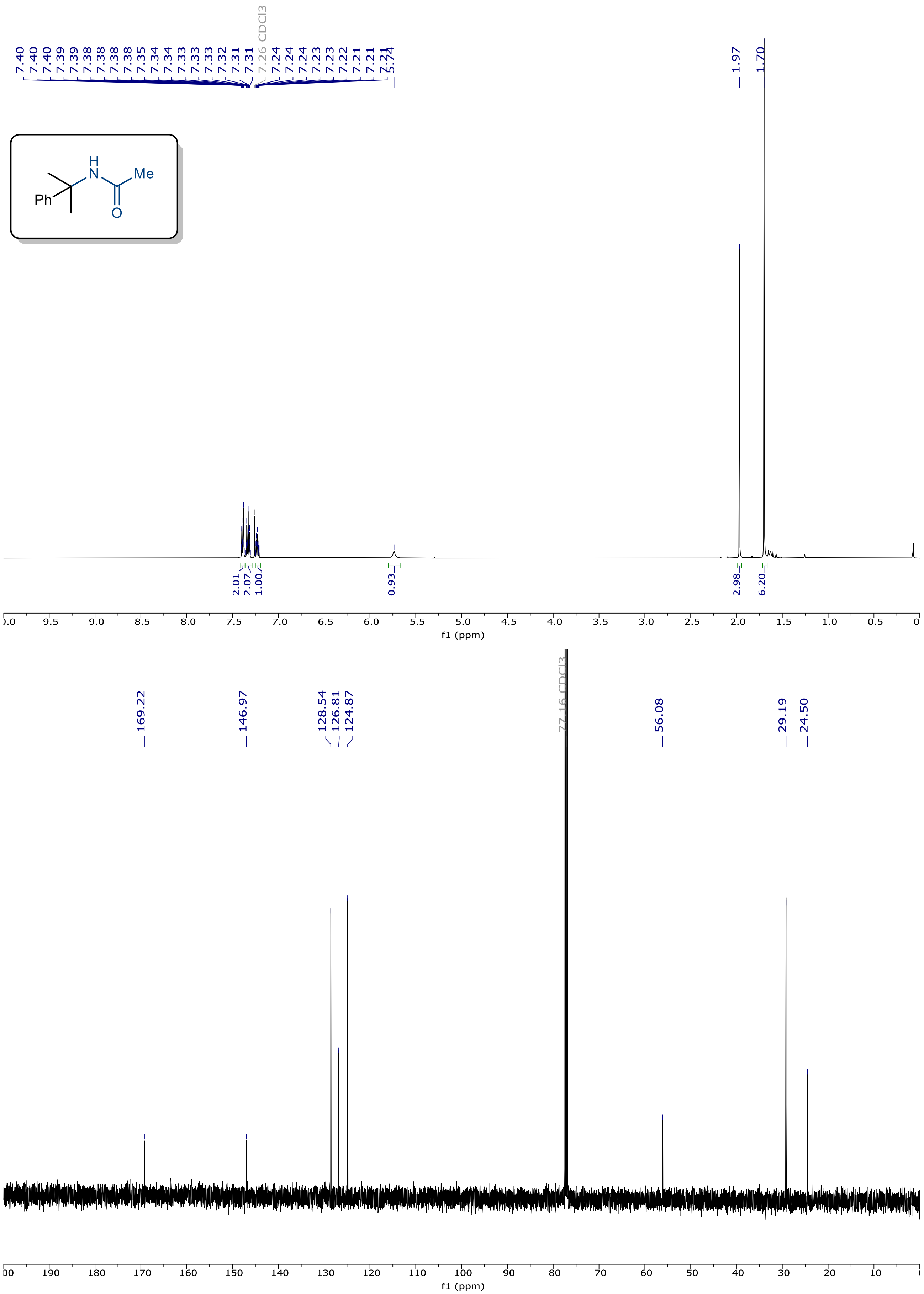

Supplementary Fig. 52 | ^{1}H (top) and ^{13}C (bottom) NMR spectra of **26.**

N-(1-Phenylcyclopentyl)acetamide (27)

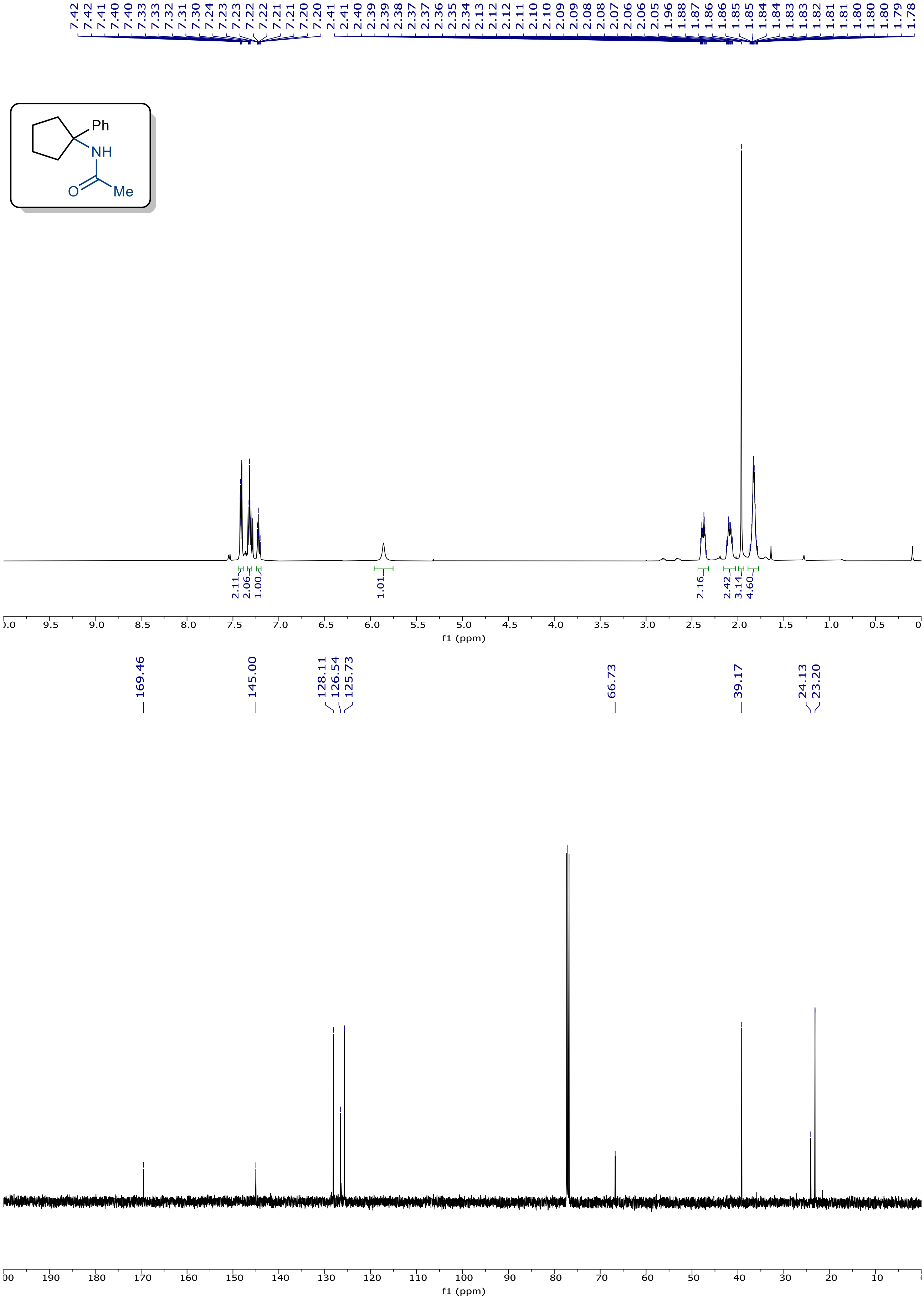

Supplementary Fig. 53 | ^{1}H (top) and ^{13}C (bottom) NMR spectra of **27.**

_N_-(1-Phenylcyclobutyl)acetamide (28)

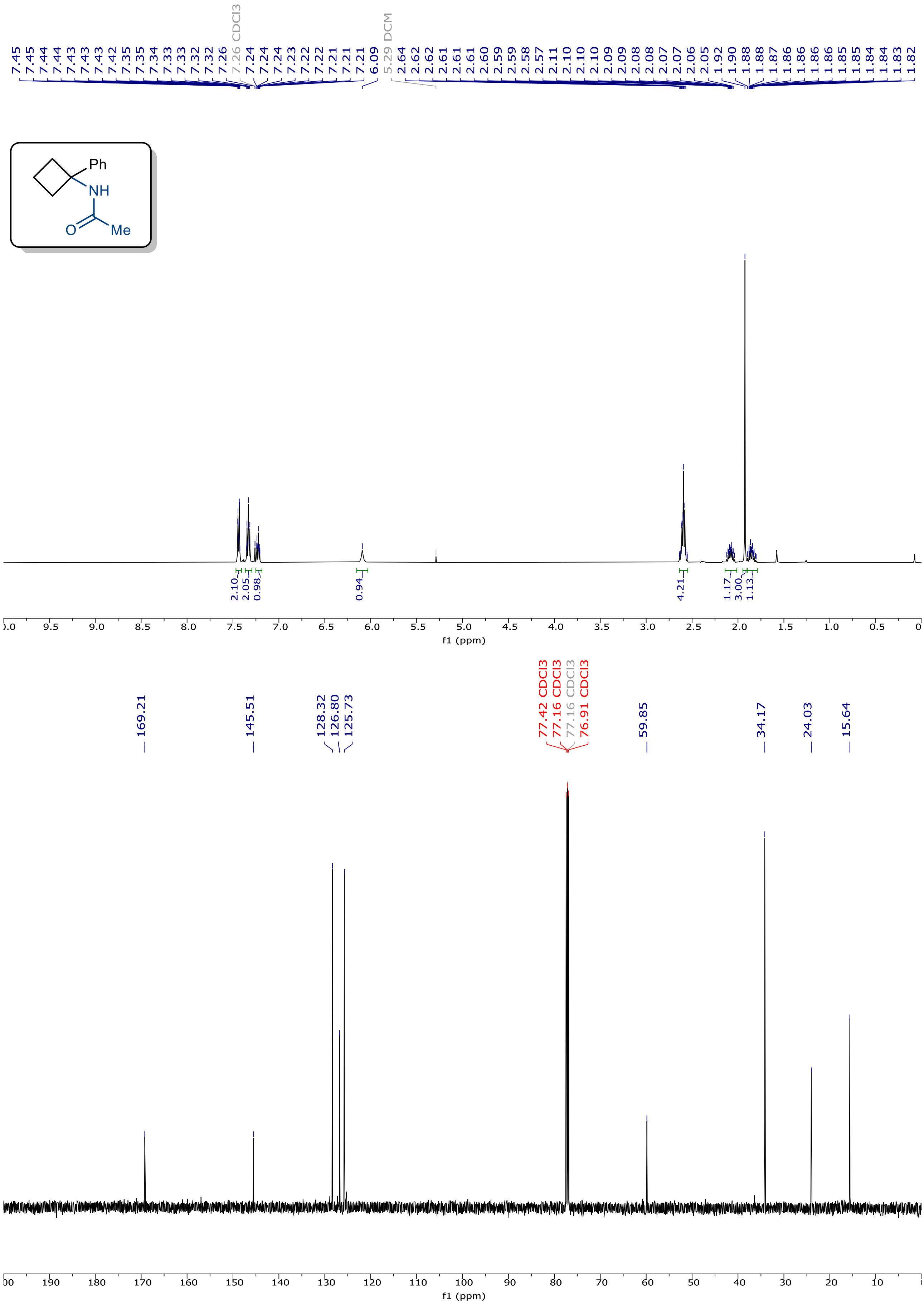

Supplementary Fig. 54 | ^{1}H (top) and ^{13}C (bottom) NMR spectra of **28.**

Methyl (1*r*,5*r*)-5-acetamidobicyclo[3.1.1]heptane-1-carboxylate (29)

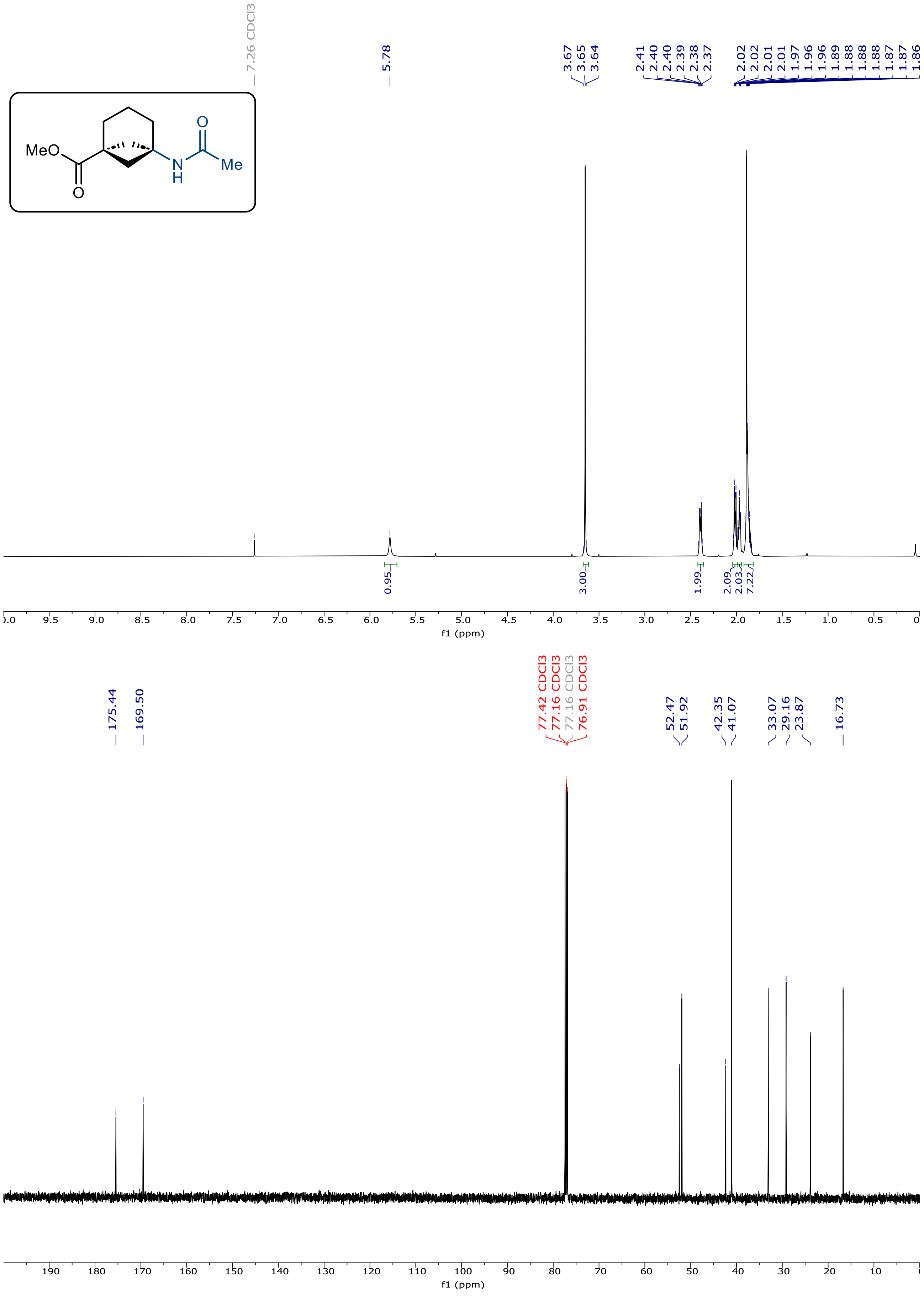

Supplementary Fig. 55 | ^{1}H (top) and ^{13}C (bottom) NMR spectra of **29.**

***N*-(Bicyclo[1.1.1]pentan-1-yl)acetamide (30)**

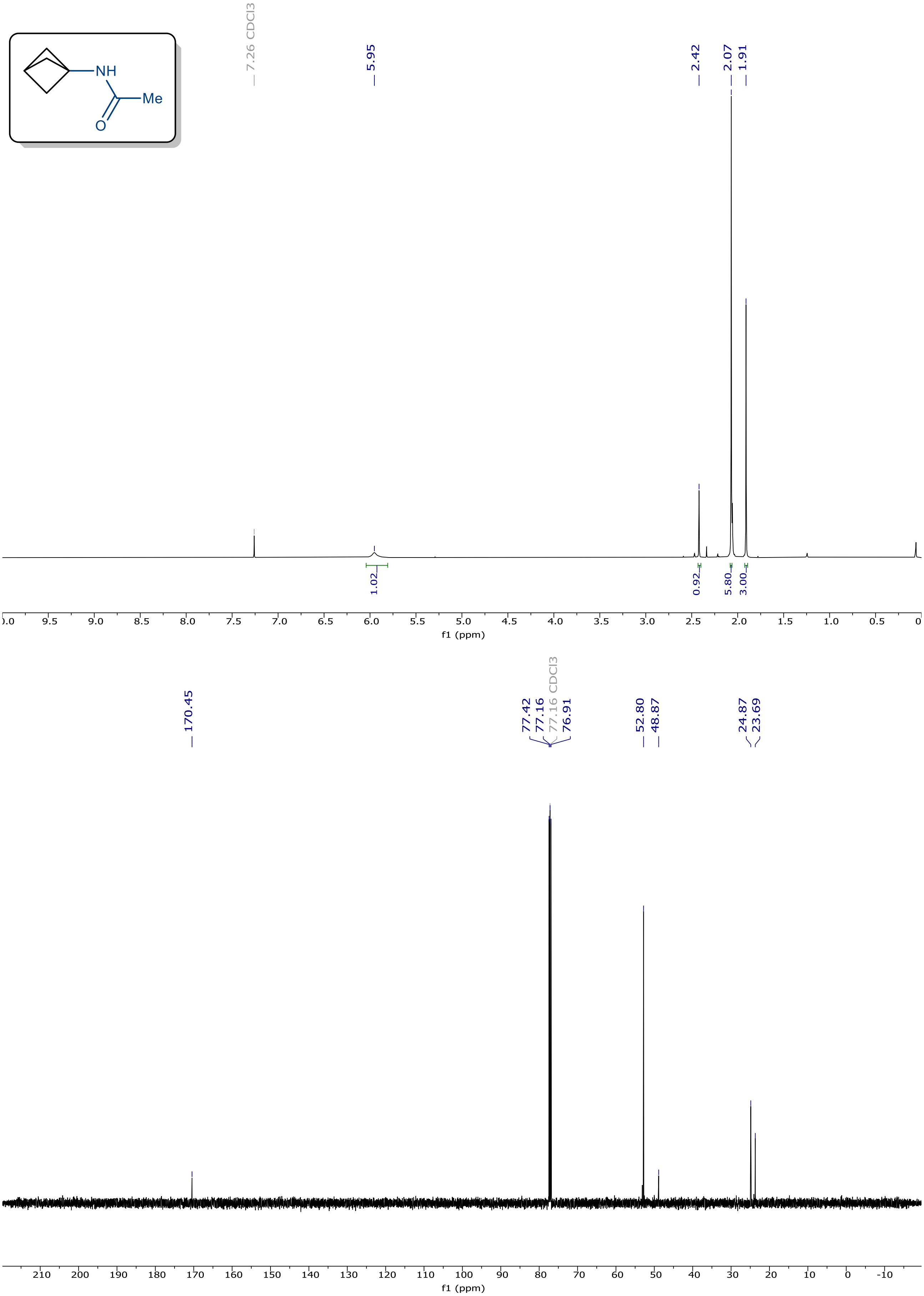

Supplementary Fig. 56 | ^{1}H (top) and ^{13}C (bottom) NMR spectra of **30.**

Methyl (2*r*,3*R*,4*s*,5*S*)-4-acetamidocubane-1-carboxylate (31)

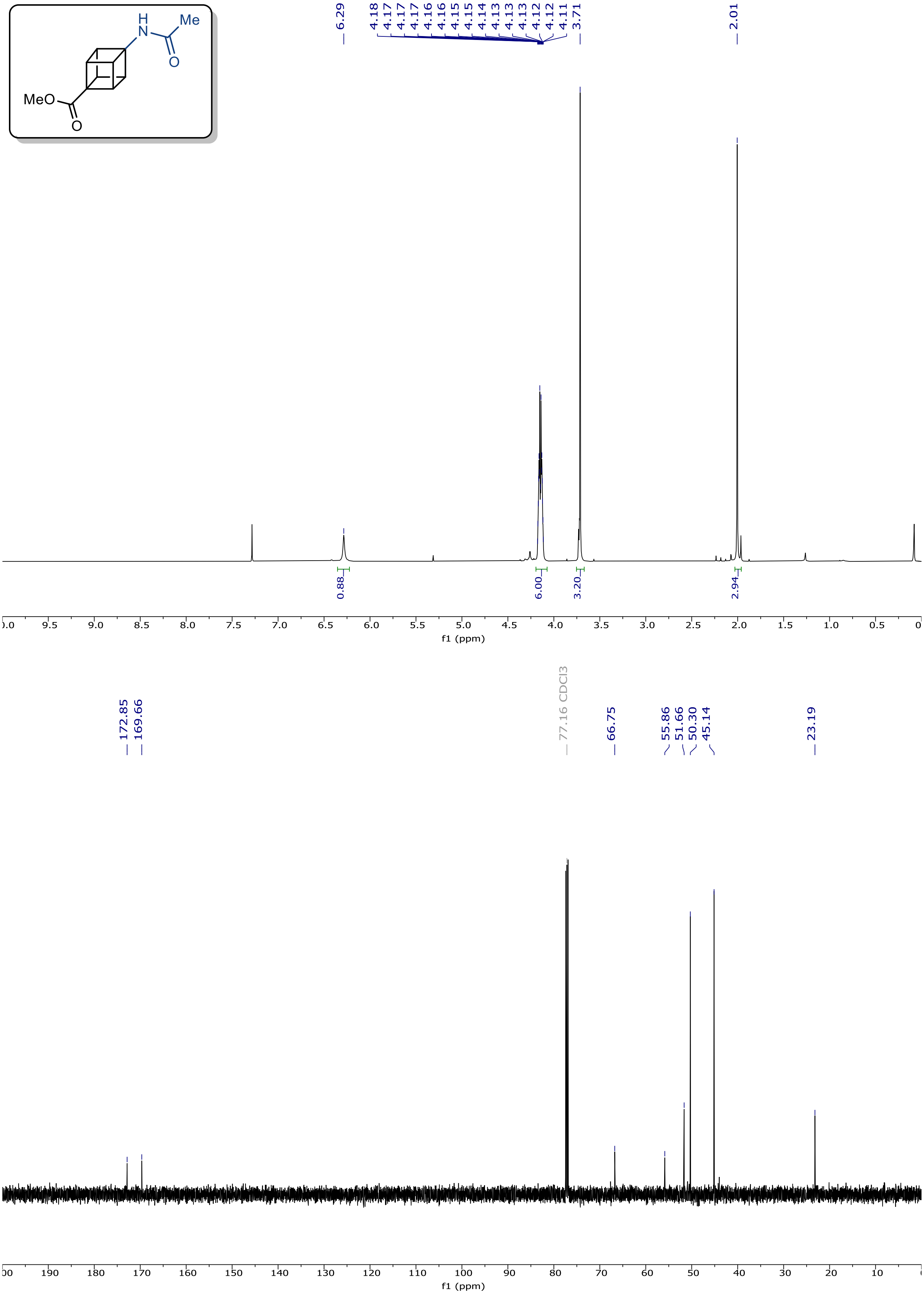

Supplementary Fig. 57 | ^{1}H (top) and ^{13}C (bottom) NMR spectra of **31.**

N-[{1-(4-Chlorobenzoyl)-5-methoxy-2-methyl-1H-indol-3-yl}methyl]acetamide (32)

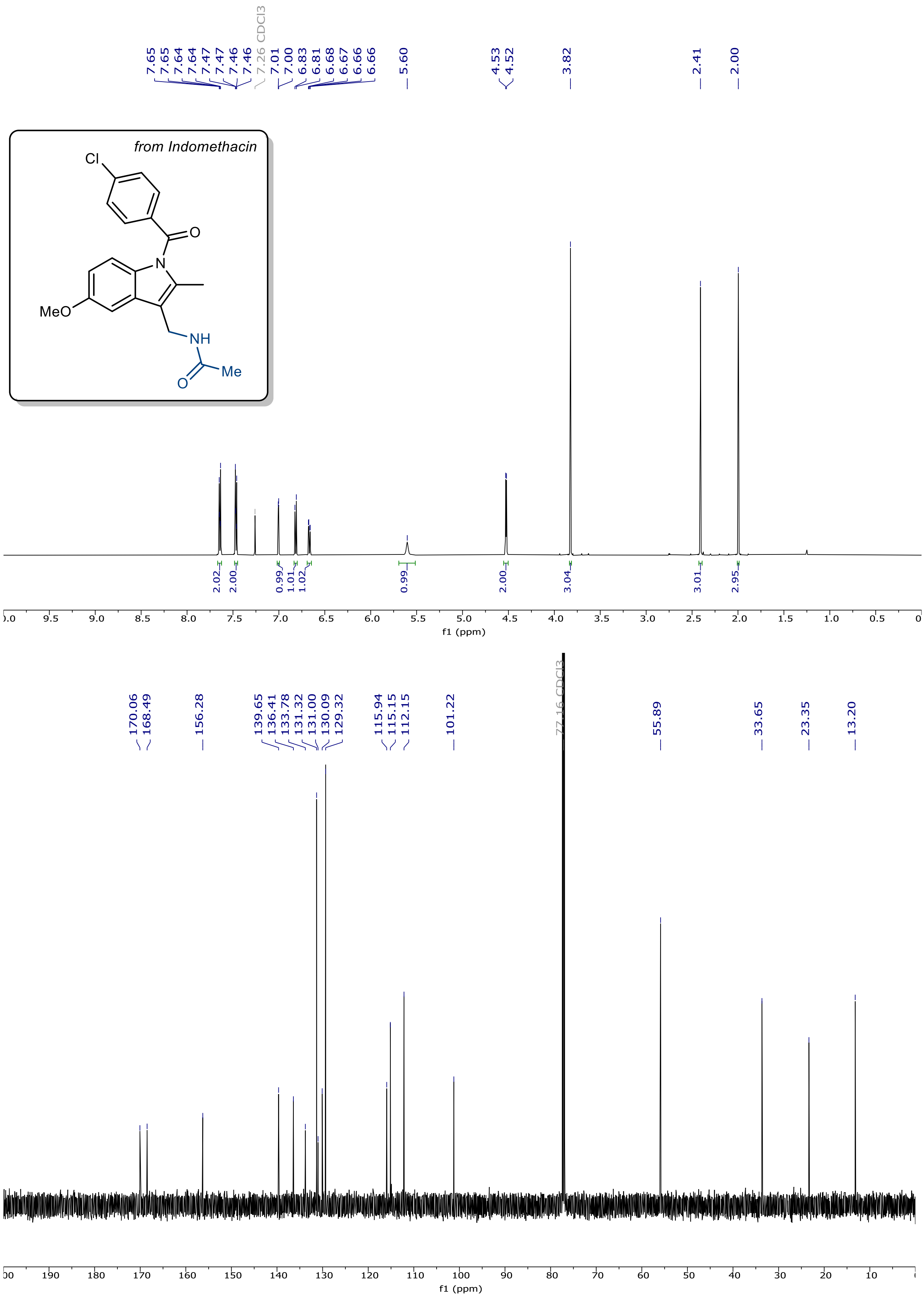

Supplementary Fig. 58 | ^{1}H (top) and ^{13}C (bottom) NMR spectra of **32.**

N-(1-[4-{(2-Oxocyclopentyl)methyl}phenyl]ethyl)acetamide (33)

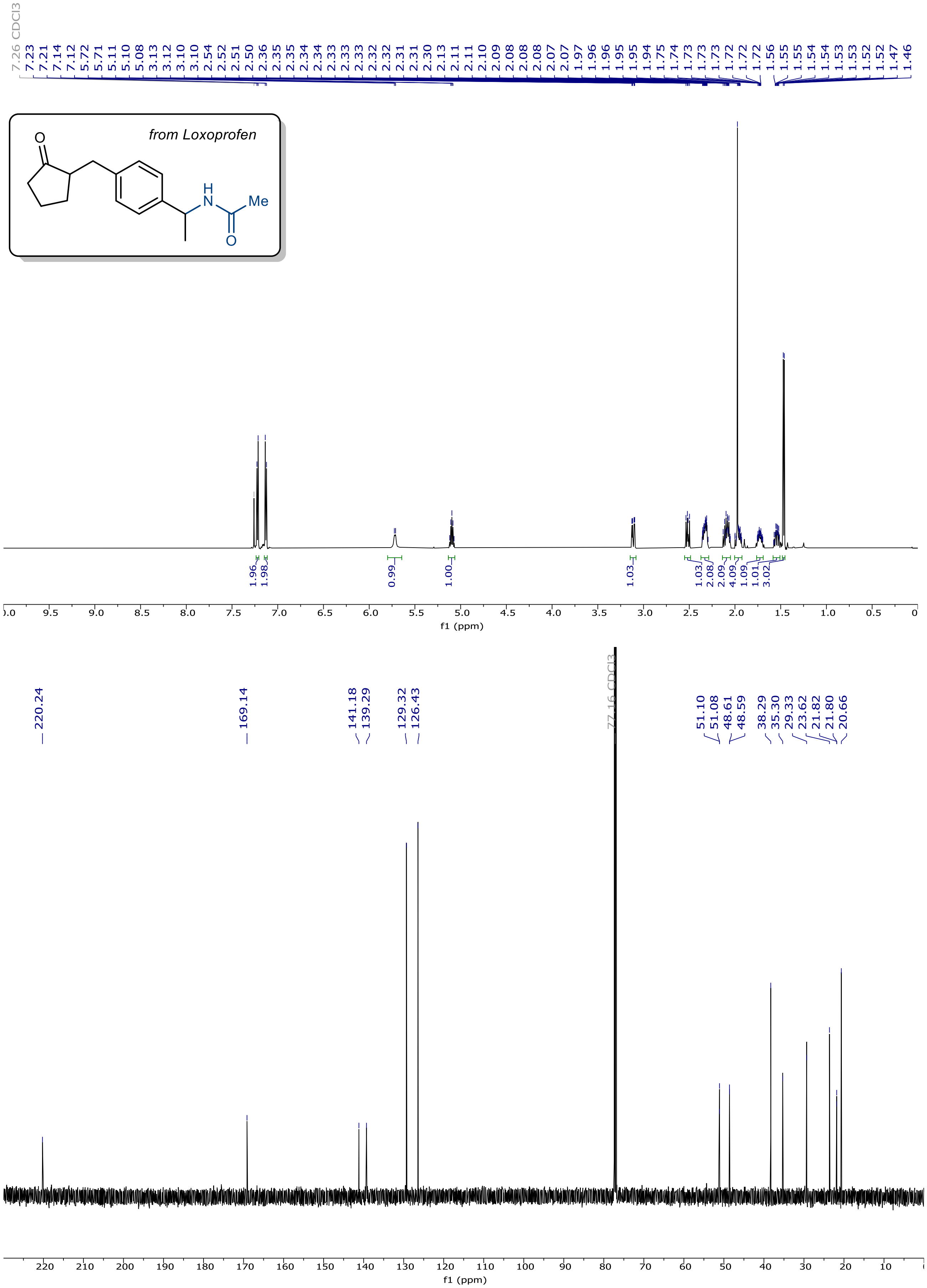

Supplementary Fig. 59 | ^{1}H (top) and ^{13}C (bottom) NMR spectra of **33.**

N-{1-(10-Oxo-10,11-dihydrodibenzo[b,f]thiepin-2-yl)ethyl}acetamide (34)

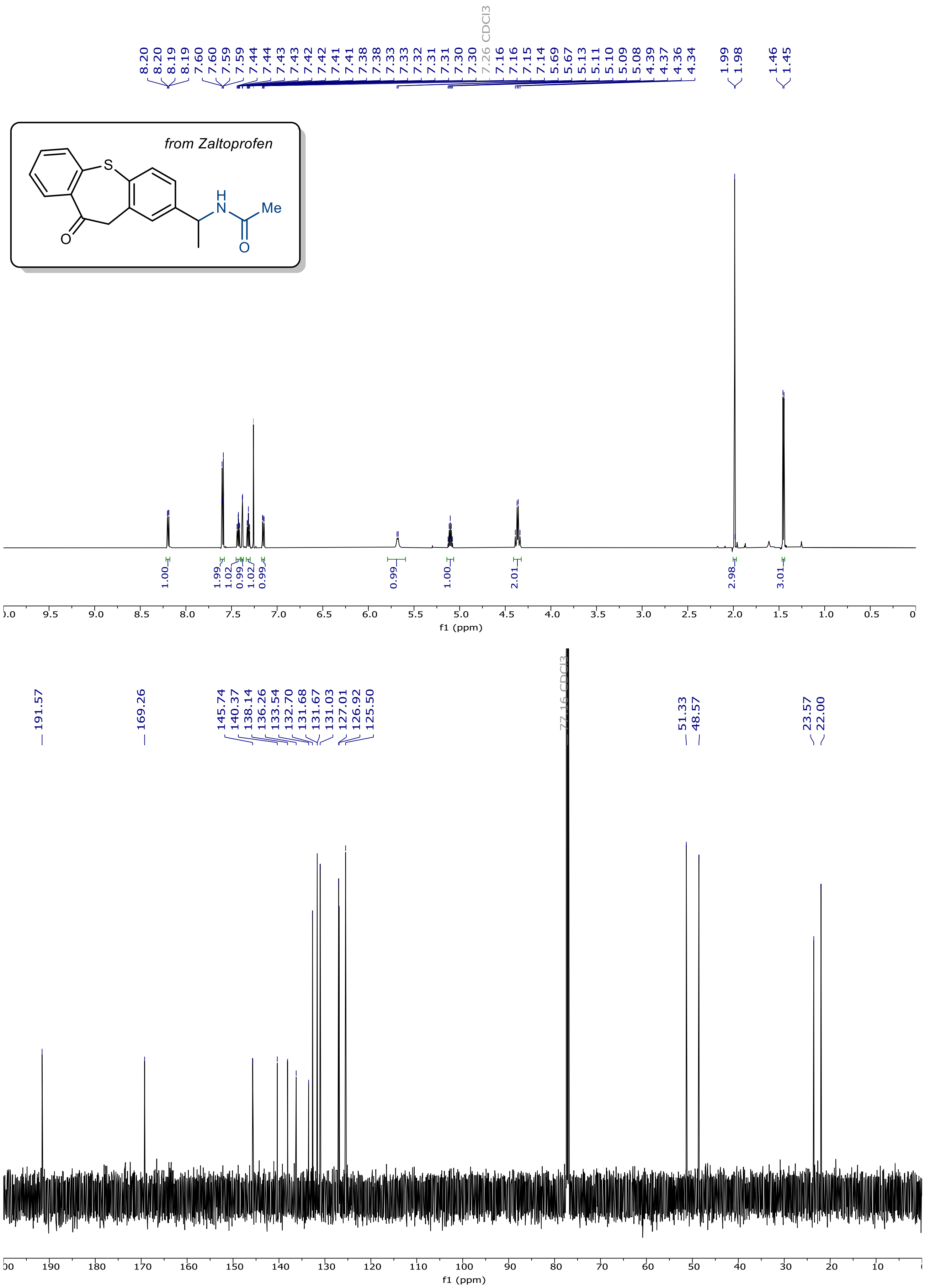

Supplementary Fig. 60 | ^{1}H (top) and ^{13}C (bottom) NMR spectra of **34.**

(*Z*)-*N*-([5-Fluoro-2-methyl-1-{4-(methylsulfinyl)benzylidene}-1H-inden-3-yl]methyl)acetamide (35)

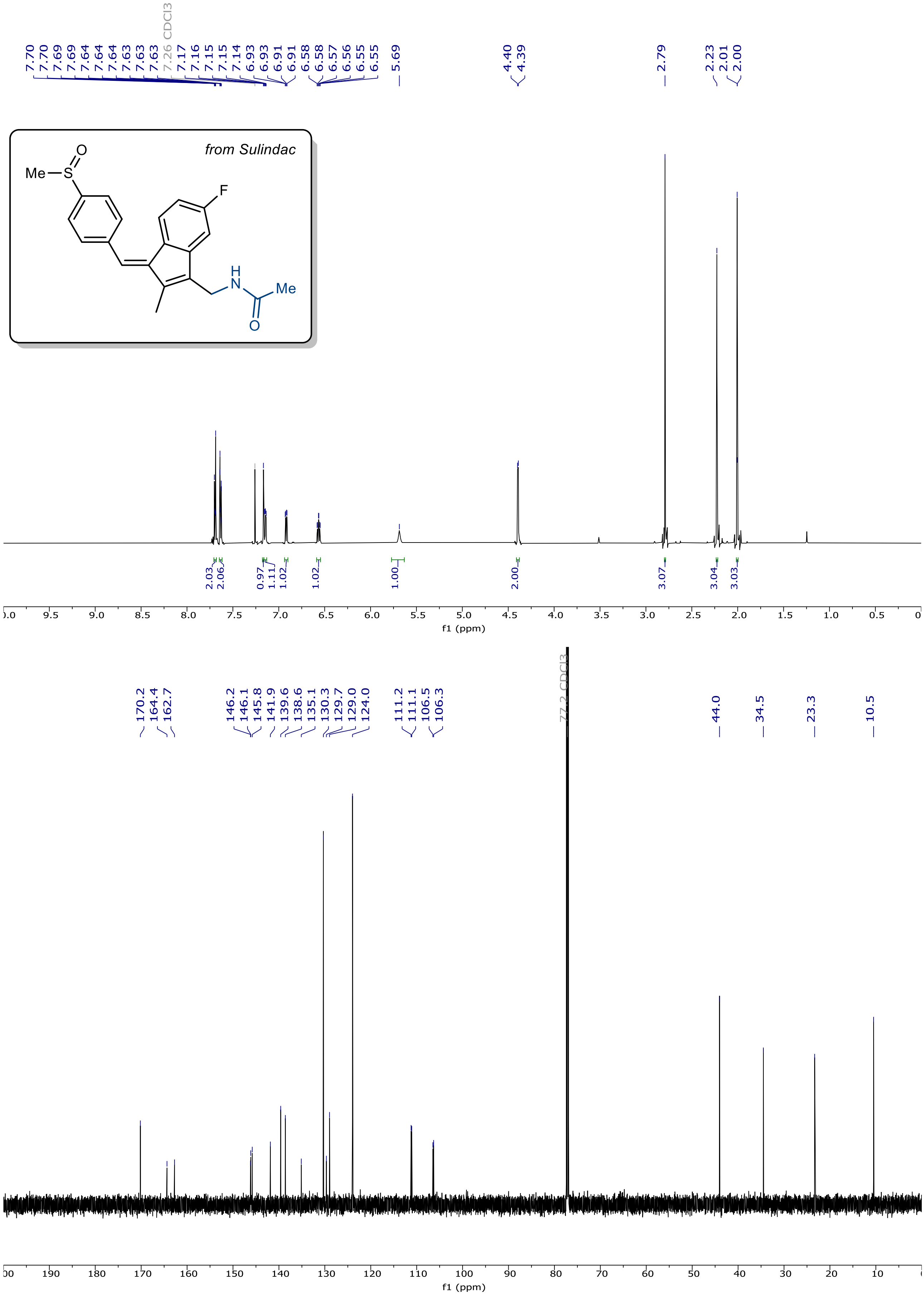

Supplementary Fig. 61 | ^{1}H (top) and ^{13}C (bottom) NMR spectra of **35.**

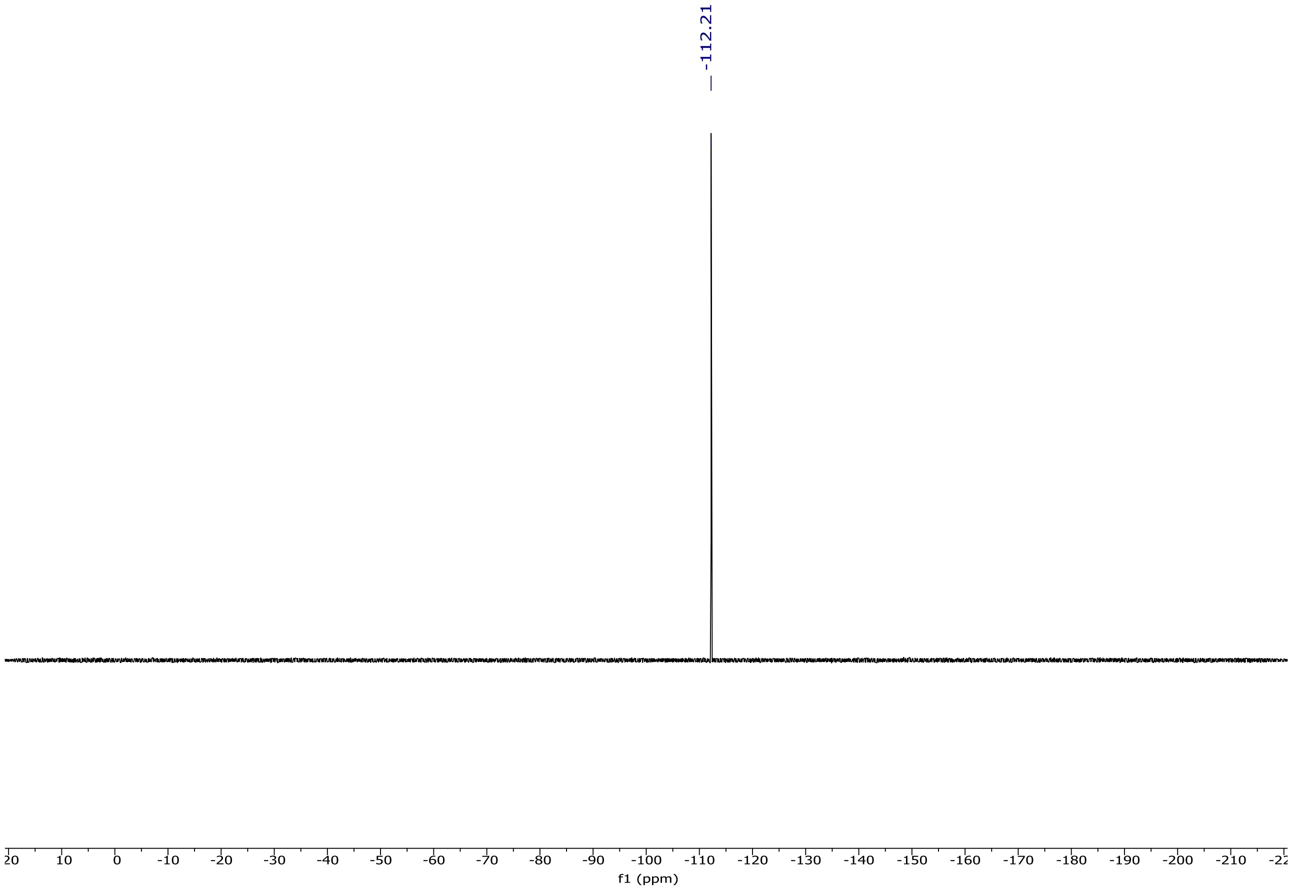

Supplementary Fig. 62 | ^{19}F NMR spectra of **35.**

***N*-[(*R*)-3-{(3*R*,5*R*,8*R*,9*S*,10*S*,13*R*,14*S*,17*R*)-3-Hydroxy-10,13-dimethylhexadecahydro-1H-cyclopenta[a]phenanthren-17-yl}butyl]acetamide (36)**

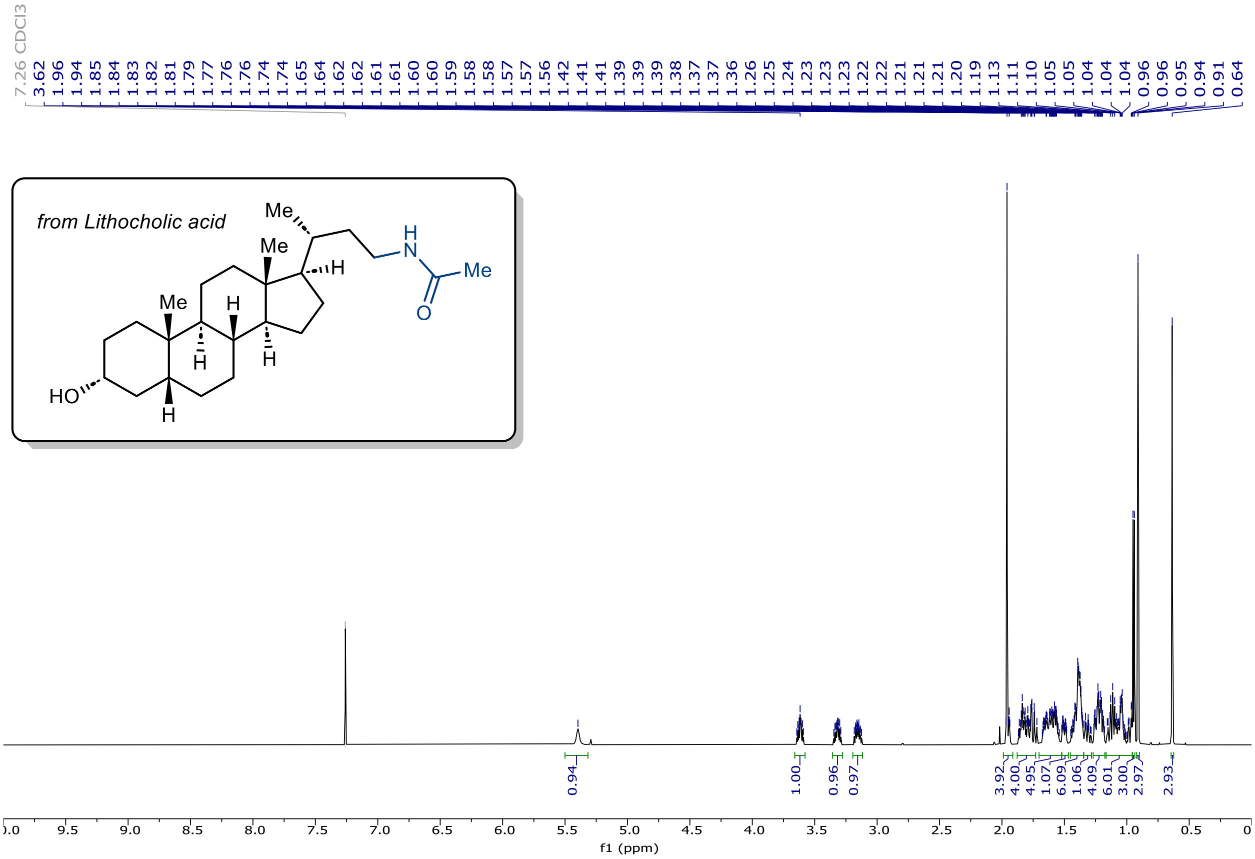

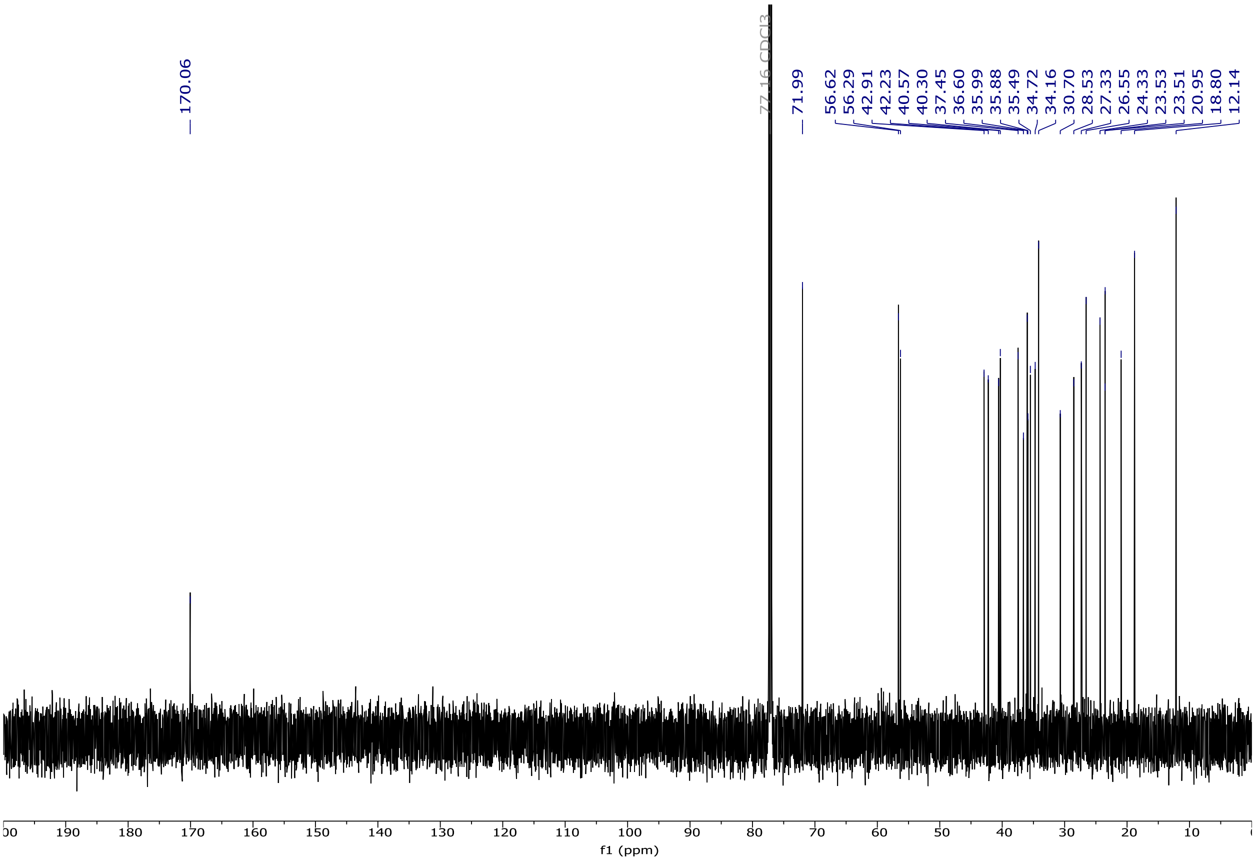

Supplementary Fig. 63 | ^{1}H (top) and ^{13}C (bottom) NMR spectra of **36.**

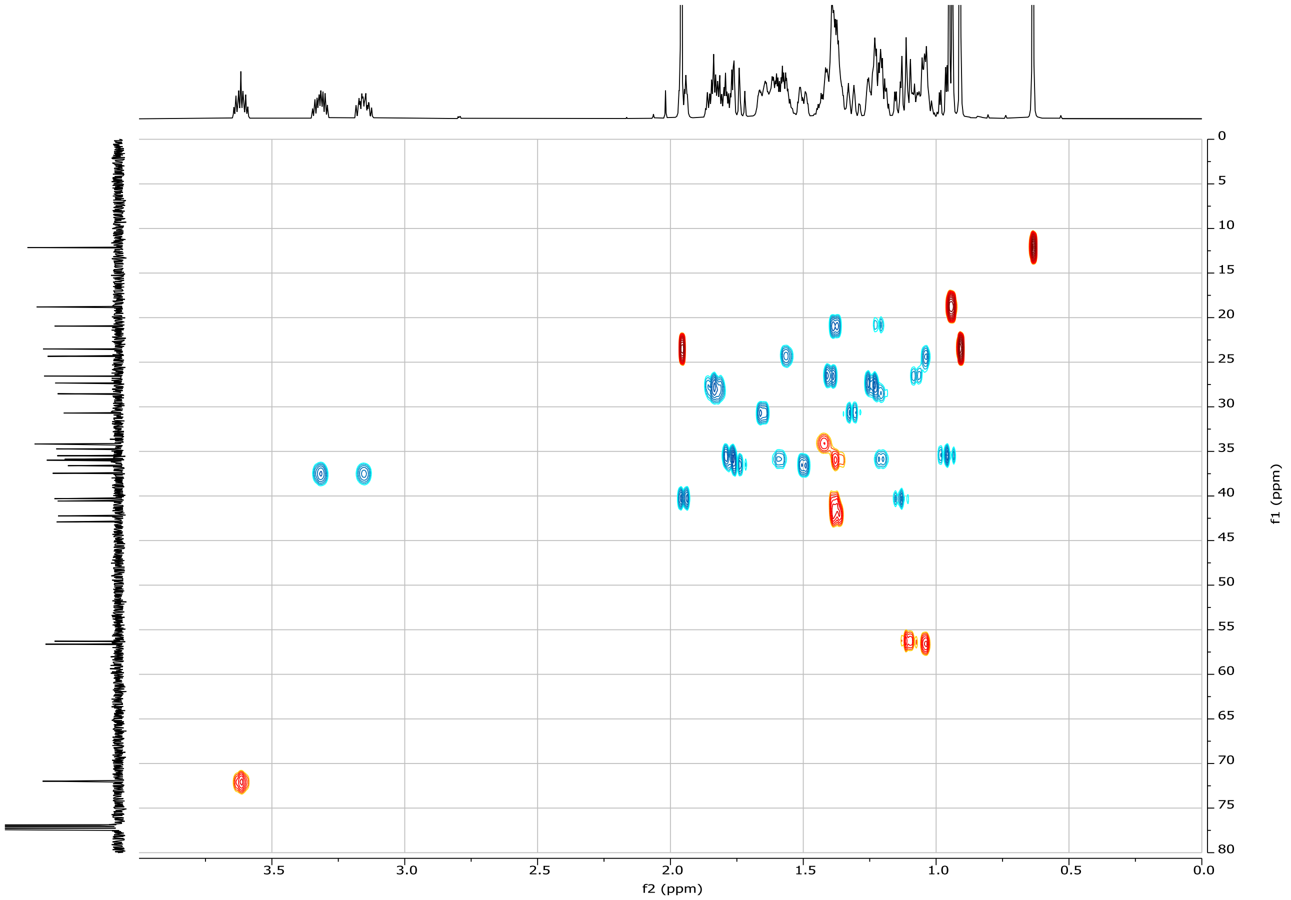

Supplementary Fig. 64 | HSQC spectra of **36.**

(*E*)-*N*-(1-Phenylprop-1-en-2-yl)acetamide (37)

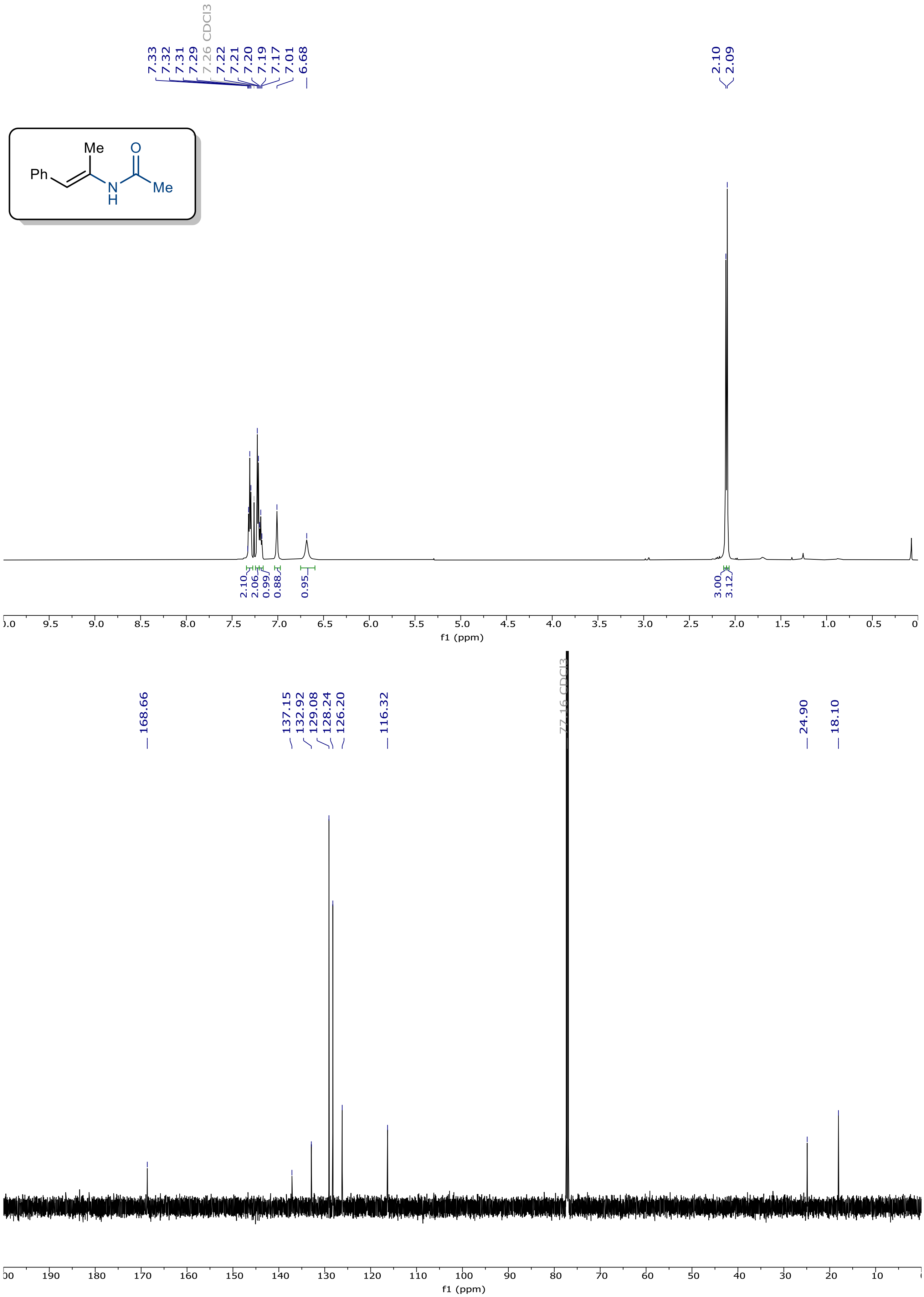

Supplementary Fig. 65 | ^{1}H (top) and ^{13}C (bottom) NMR spectra of **37.**

N-Phenylacetamide (38)

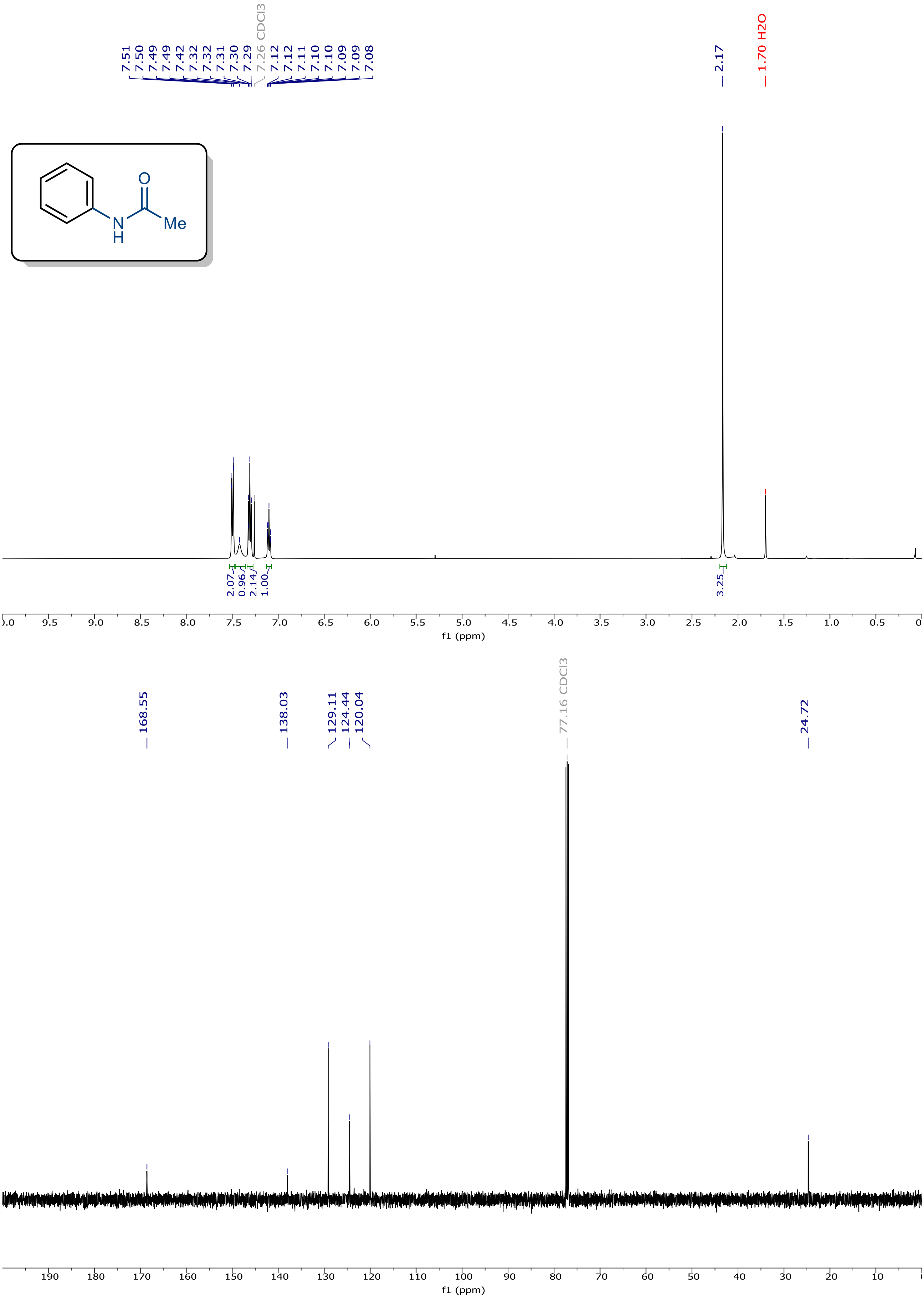

Supplementary Fig. 66 | ^{1}H (top) and ^{13}C (bottom) NMR spectra of **38.**

_N_-(_p_-Tolyl)acetamide (39)

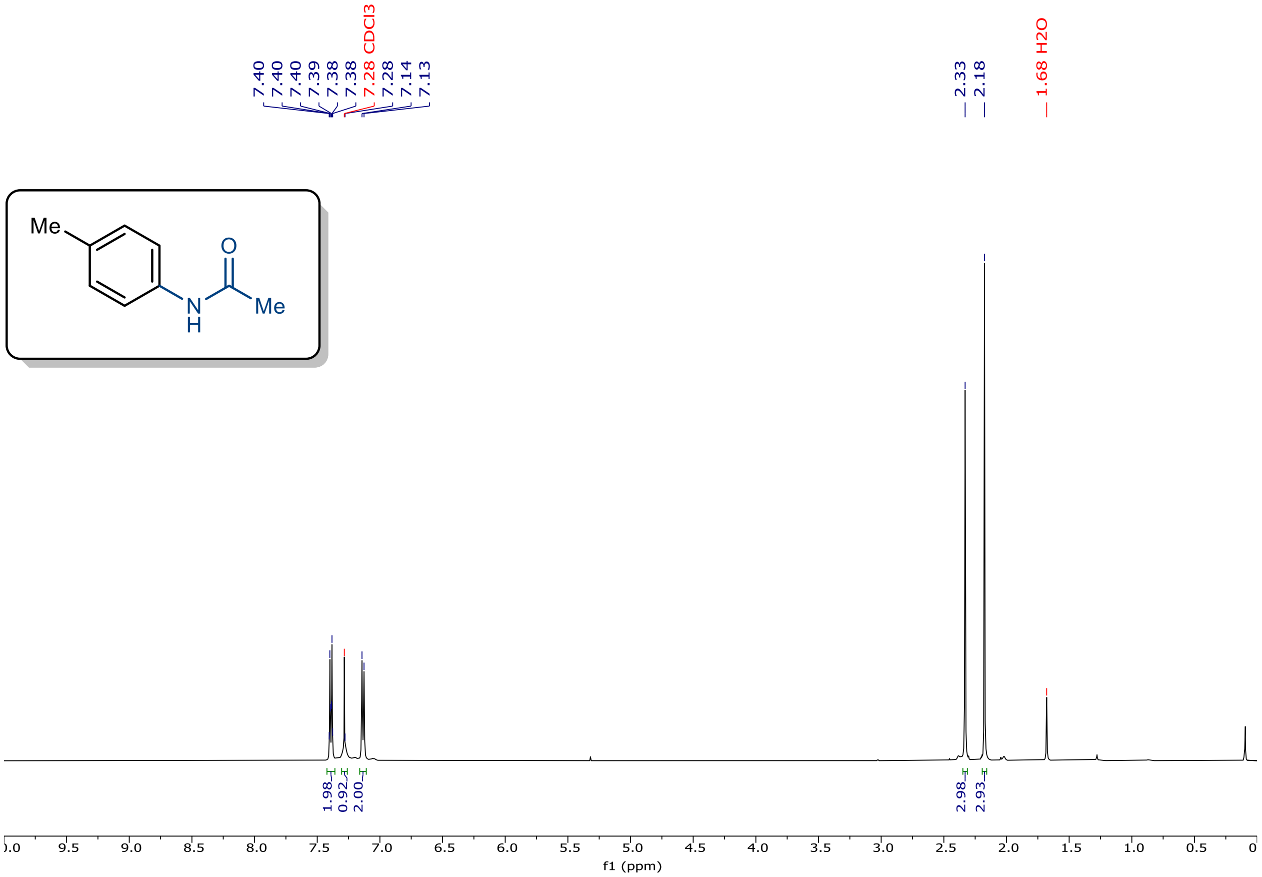

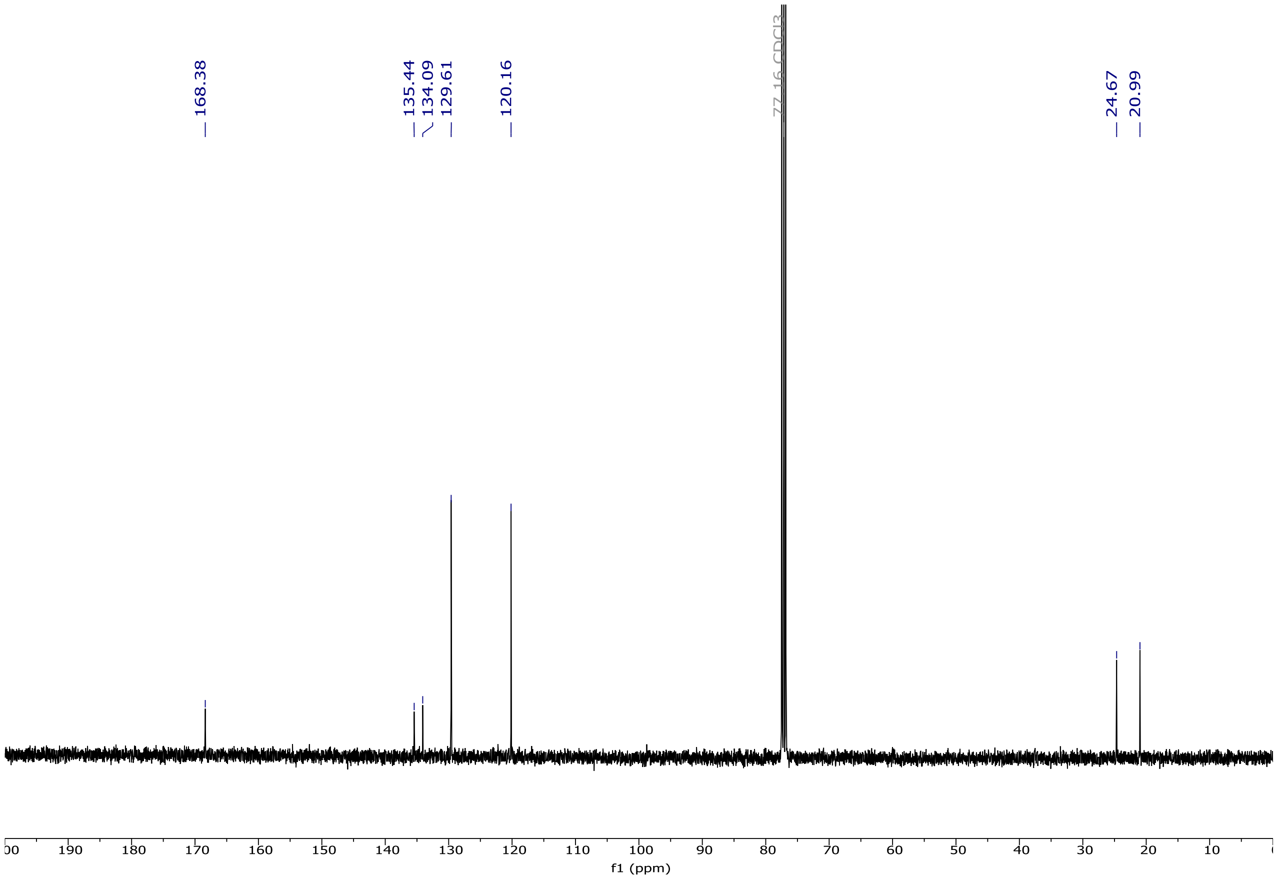

Supplementary Fig. 67 | ^{1}H (top) and ^{13}C (bottom) NMR spectra of **39.**

N-(3-Methoxyphenyl)acetamide (40)

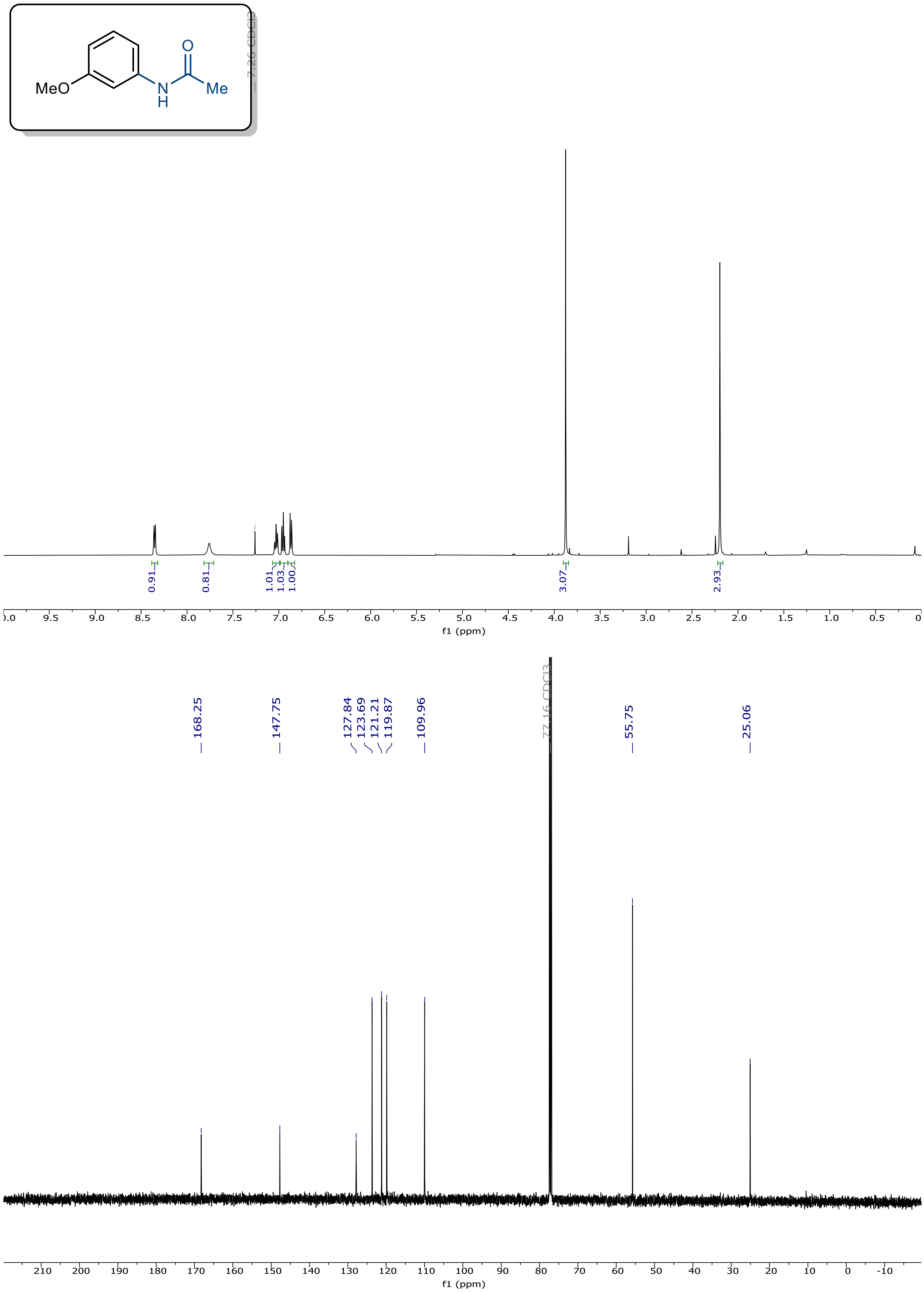

Supplementary Fig. 68 | ^{1}H (top) and ^{13}C (bottom) NMR spectra of **40.**

***N*-{4-(4,4,5,5-Tetramethyl-1,3,2-dioxaborolan-2-yl)phenyl}acetamide (41)**

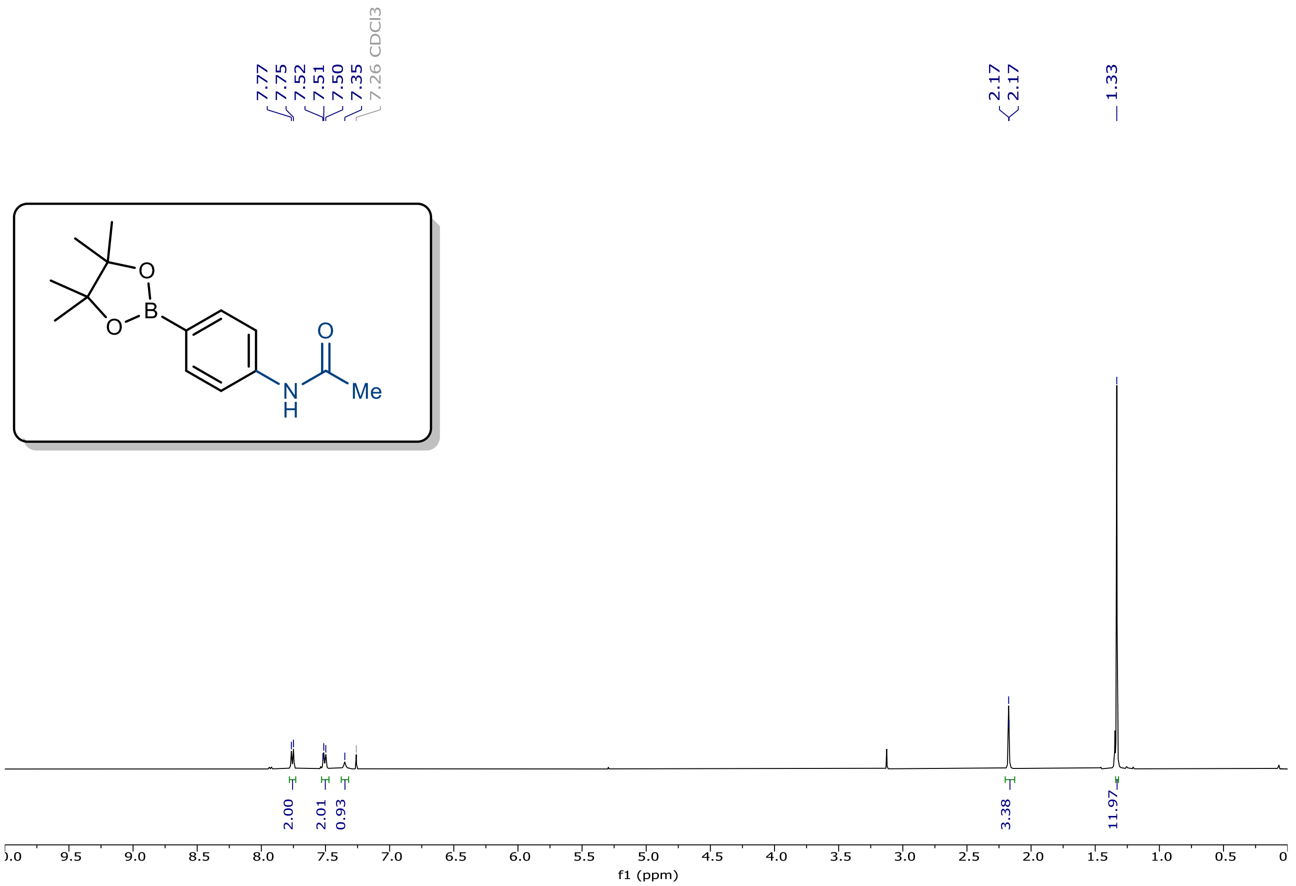

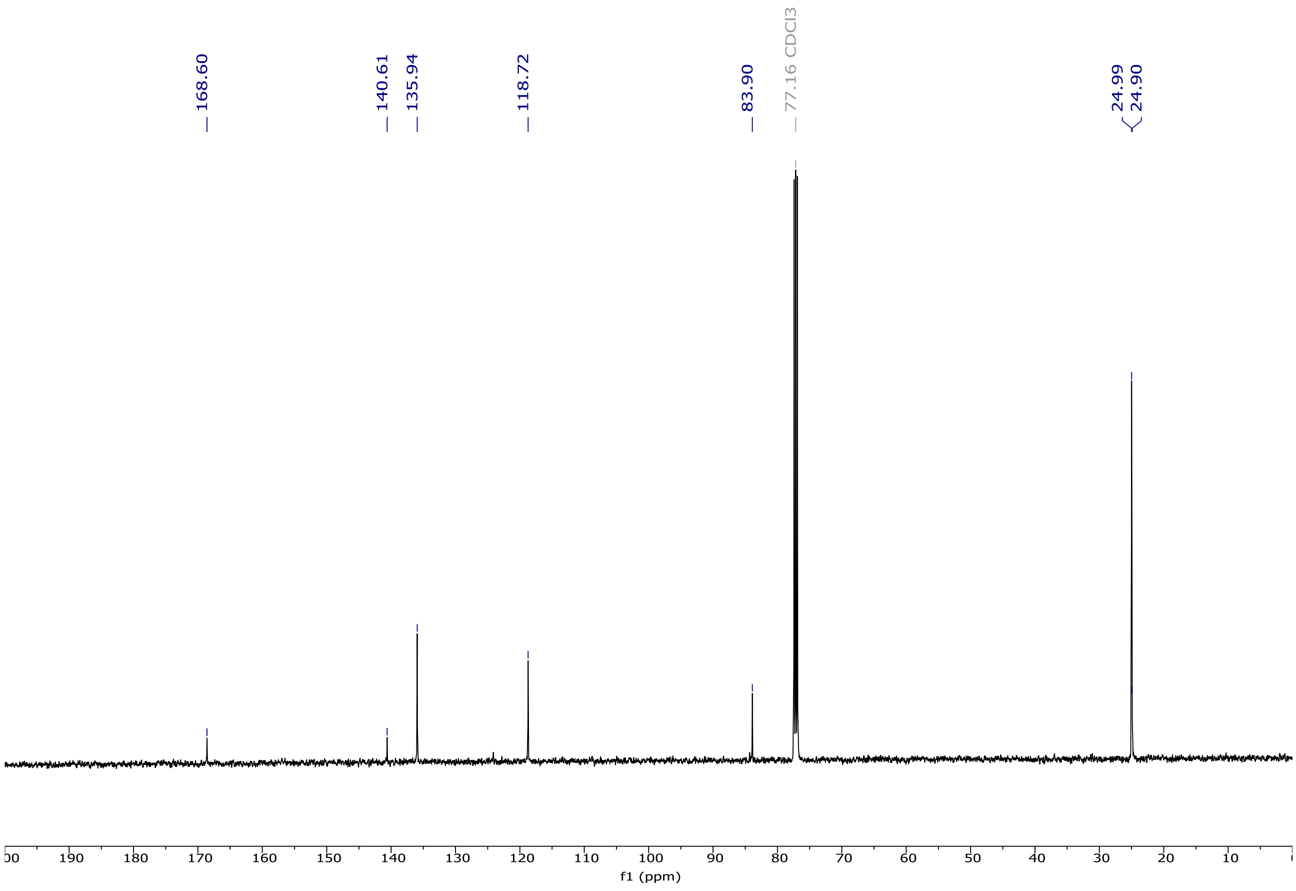

Supplementary Fig. 69 | ^{1}H (top) and ^{13}C (bottom) NMR spectra of **41.**

***N*-[6-{3-(Adamantan-1-yl)-4-methoxyphenyl}naphthalen-2-yl]acetamide (42)**

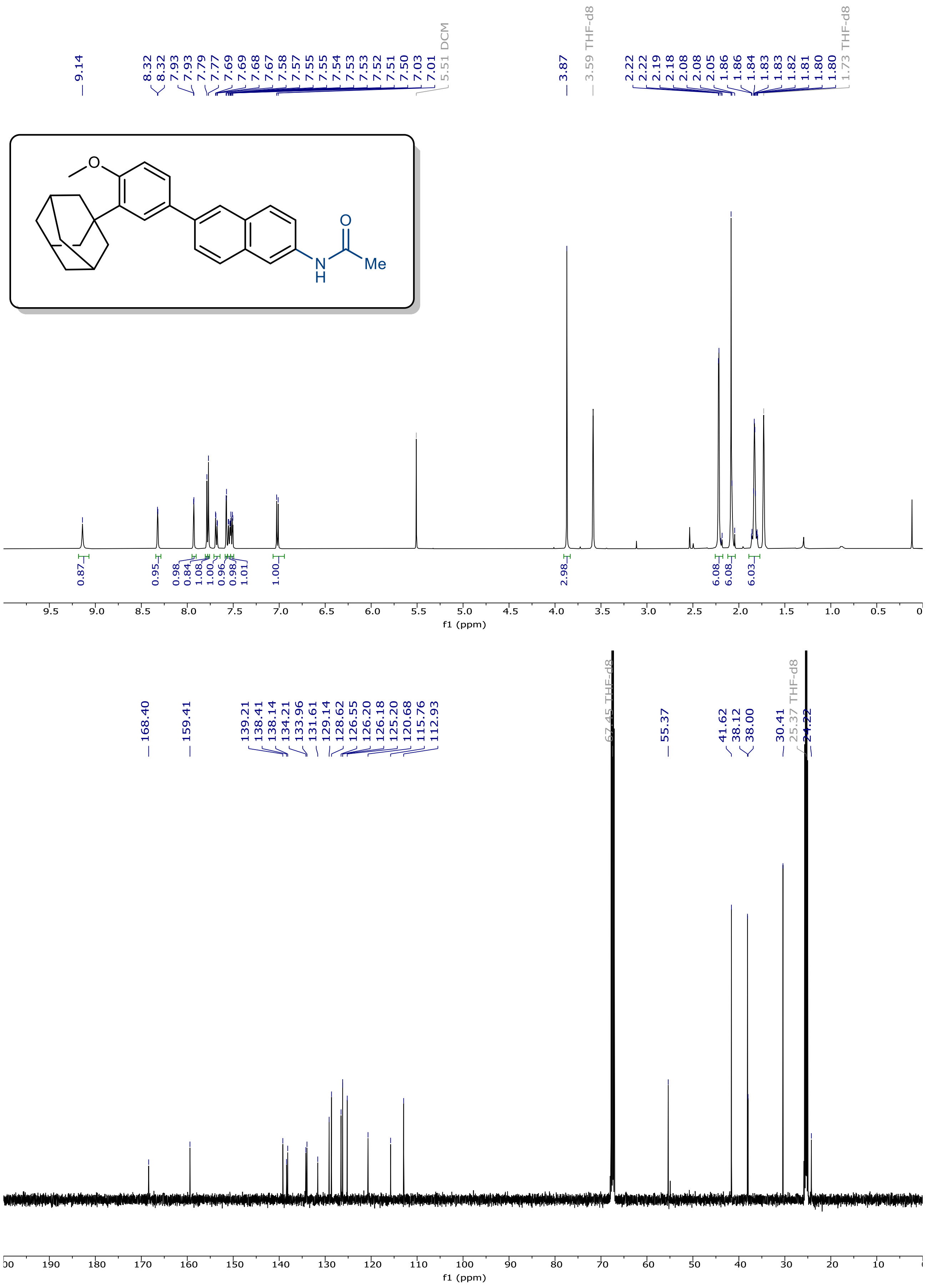

Supplementary Fig. 70 | ^{1}H (top) and ^{13}C (bottom) NMR spectra of **42.**

(*S*)-*N*-(1-Phenylethyl)acetamide (43)

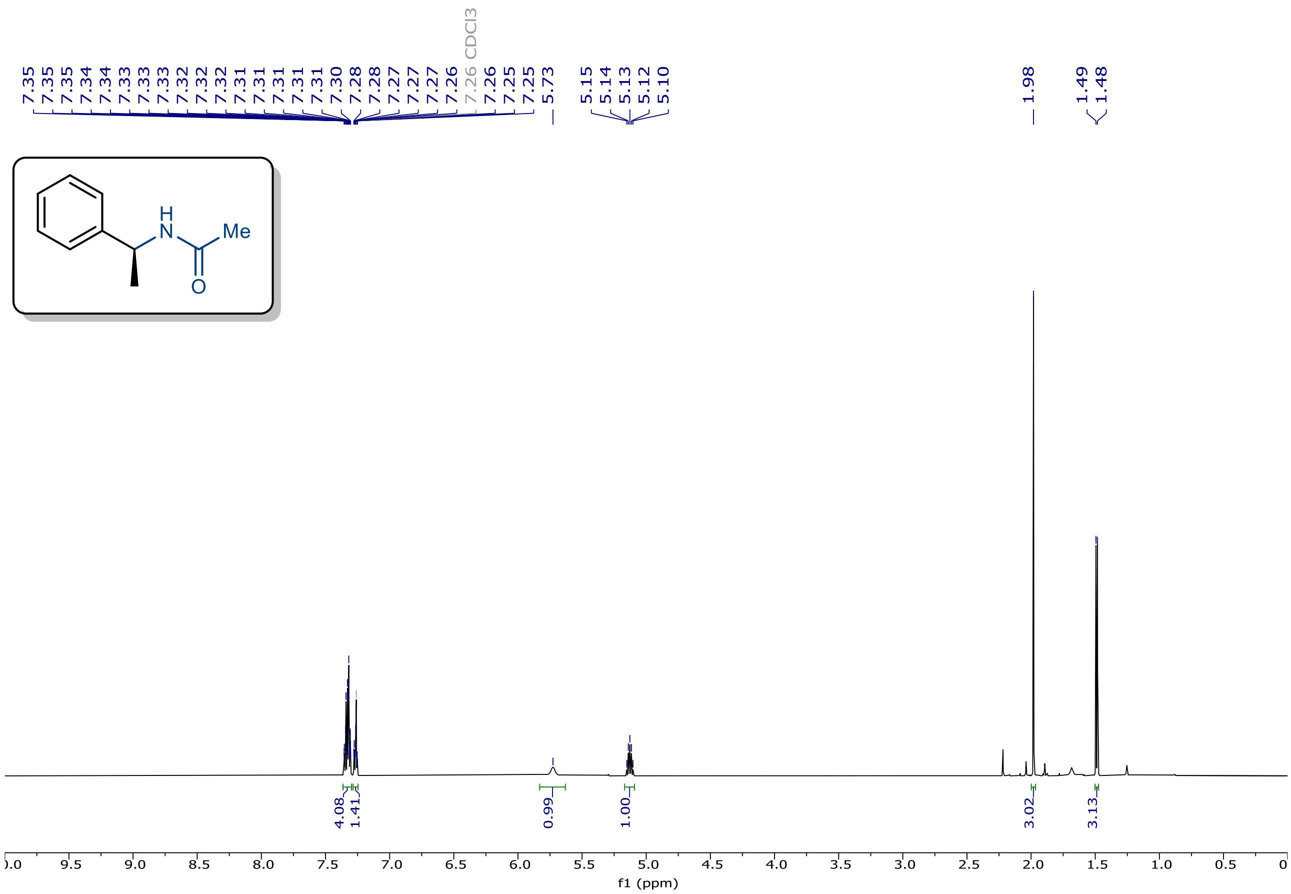

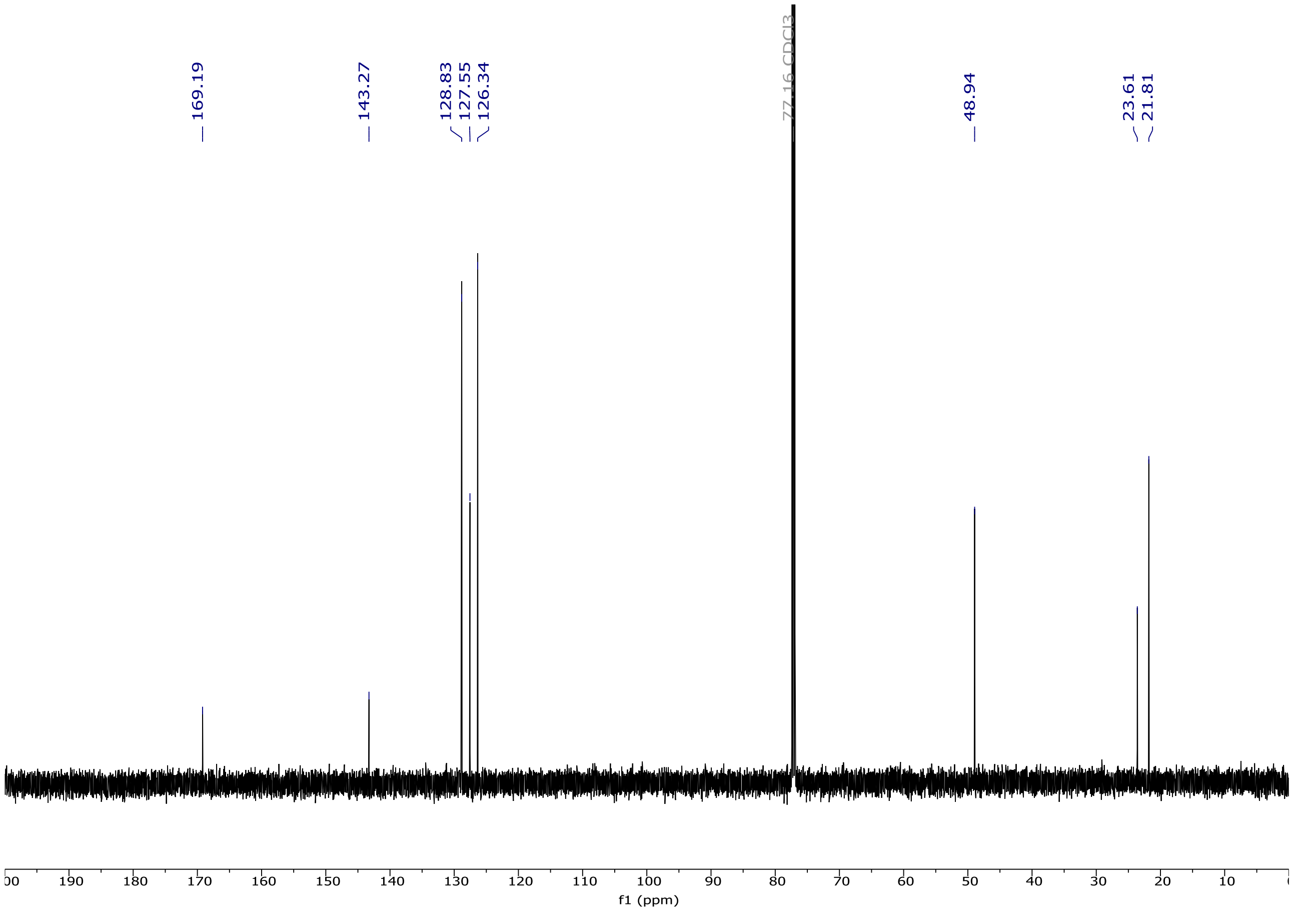

Supplementary Fig. 71 | ^{1}H (top) and ^{13}C (bottom) NMR spectra of **43.**

(*R*)-*N*-(1,2,3,4-Tetrahydronaphthalen-1-yl)acetamide (44)

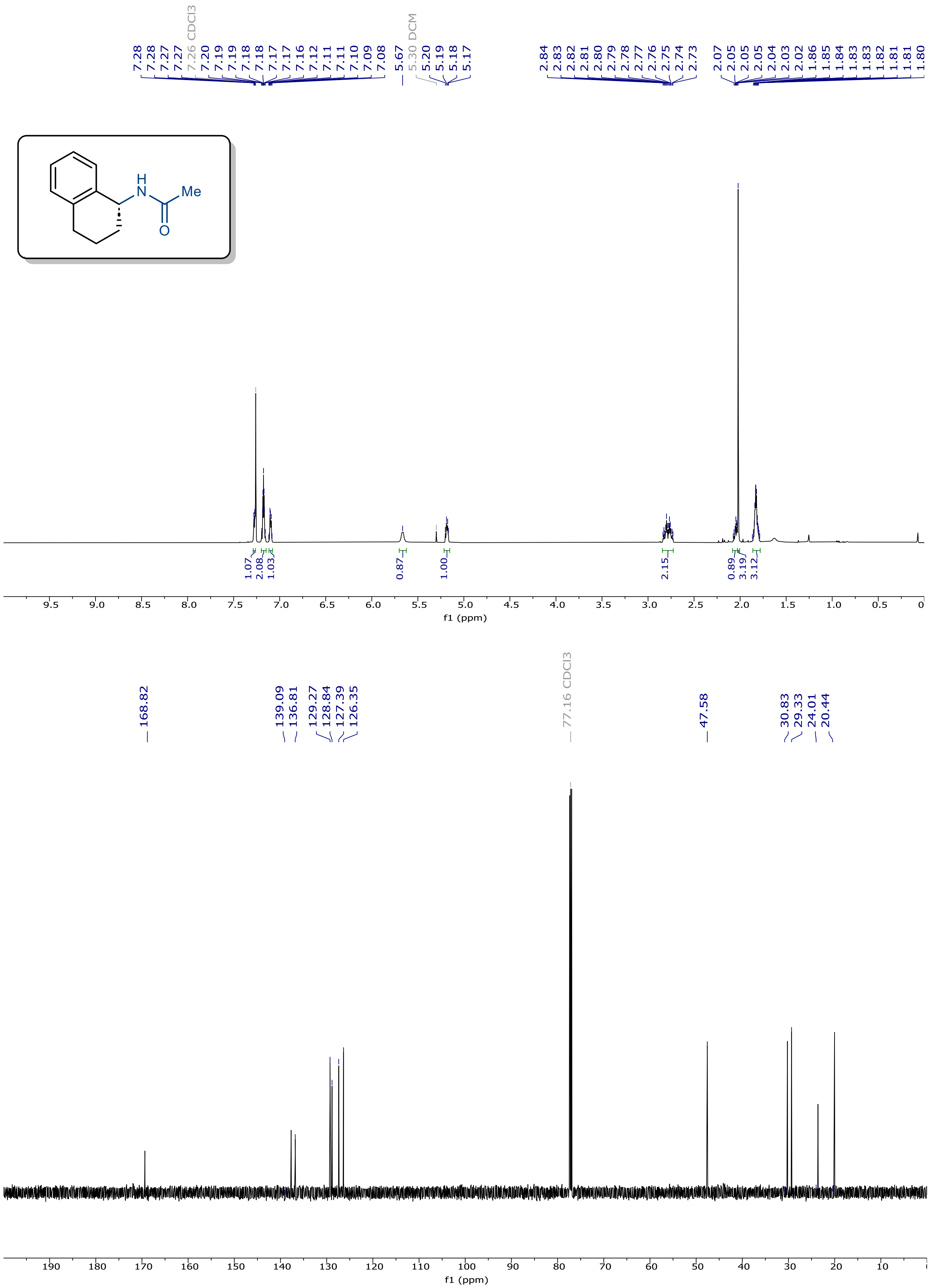

Supplementary Fig. 72 | ^{1}H (top) and ^{13}C (bottom) NMR spectra of **44.**

(*S*)-*N*-(Cyclohex-3-en-1-yl)acetamide (45)

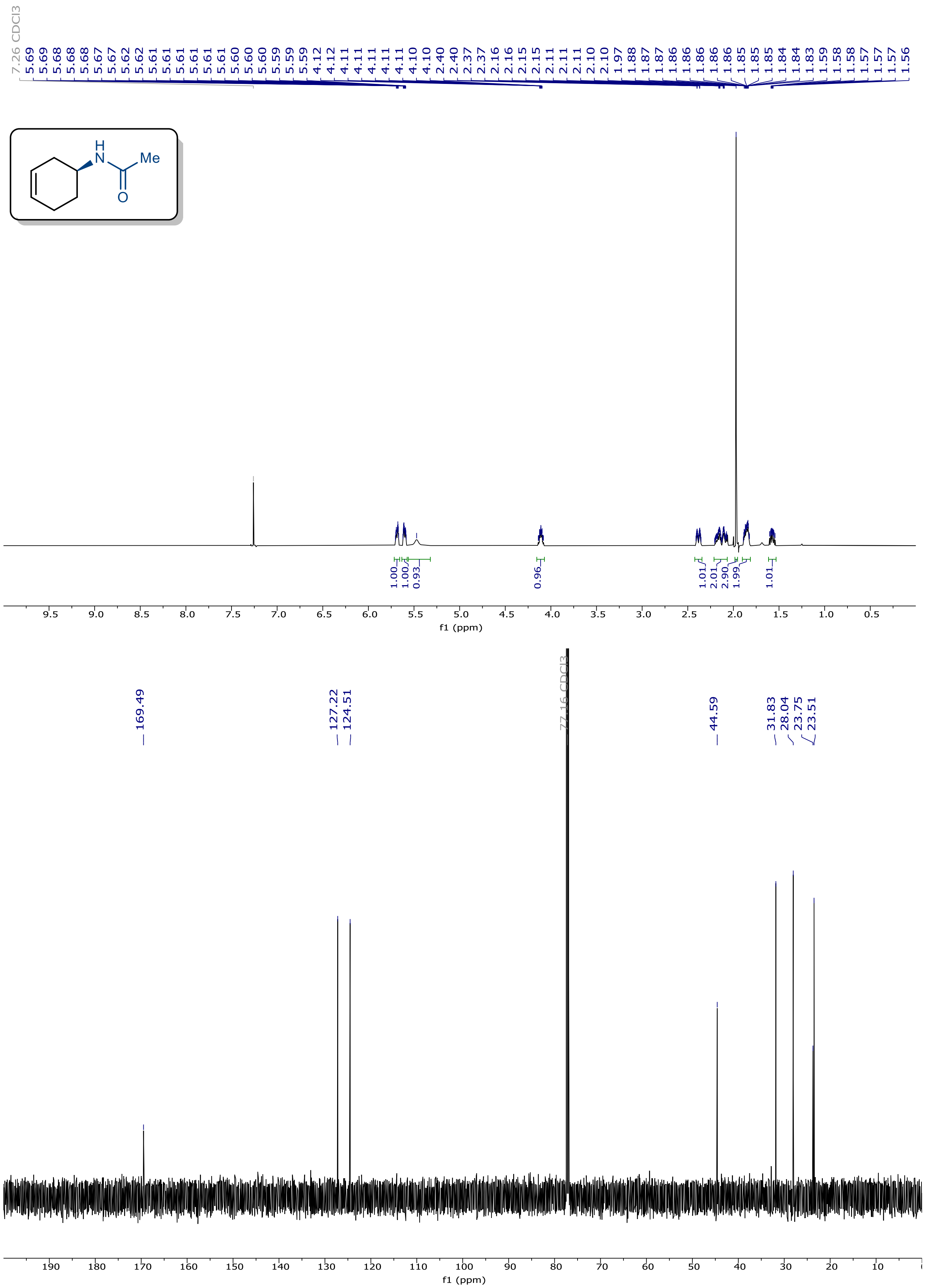

Supplementary Fig. 73 | ^{1}H (top) and ^{13}C (bottom) NMR spectra of **45.**

(*R*)-*N*-{Methoxy(phenyl)methyl}acetamide (46)

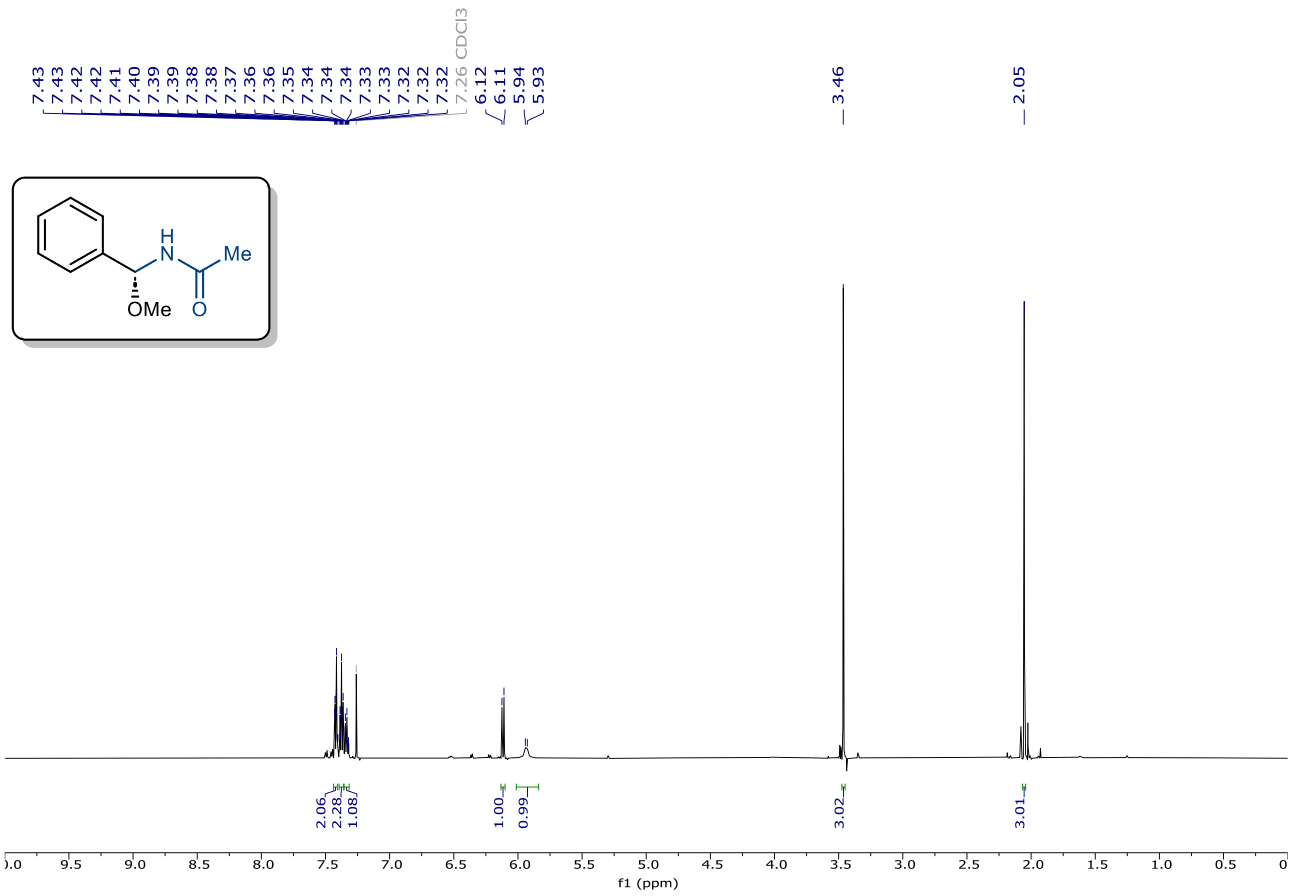

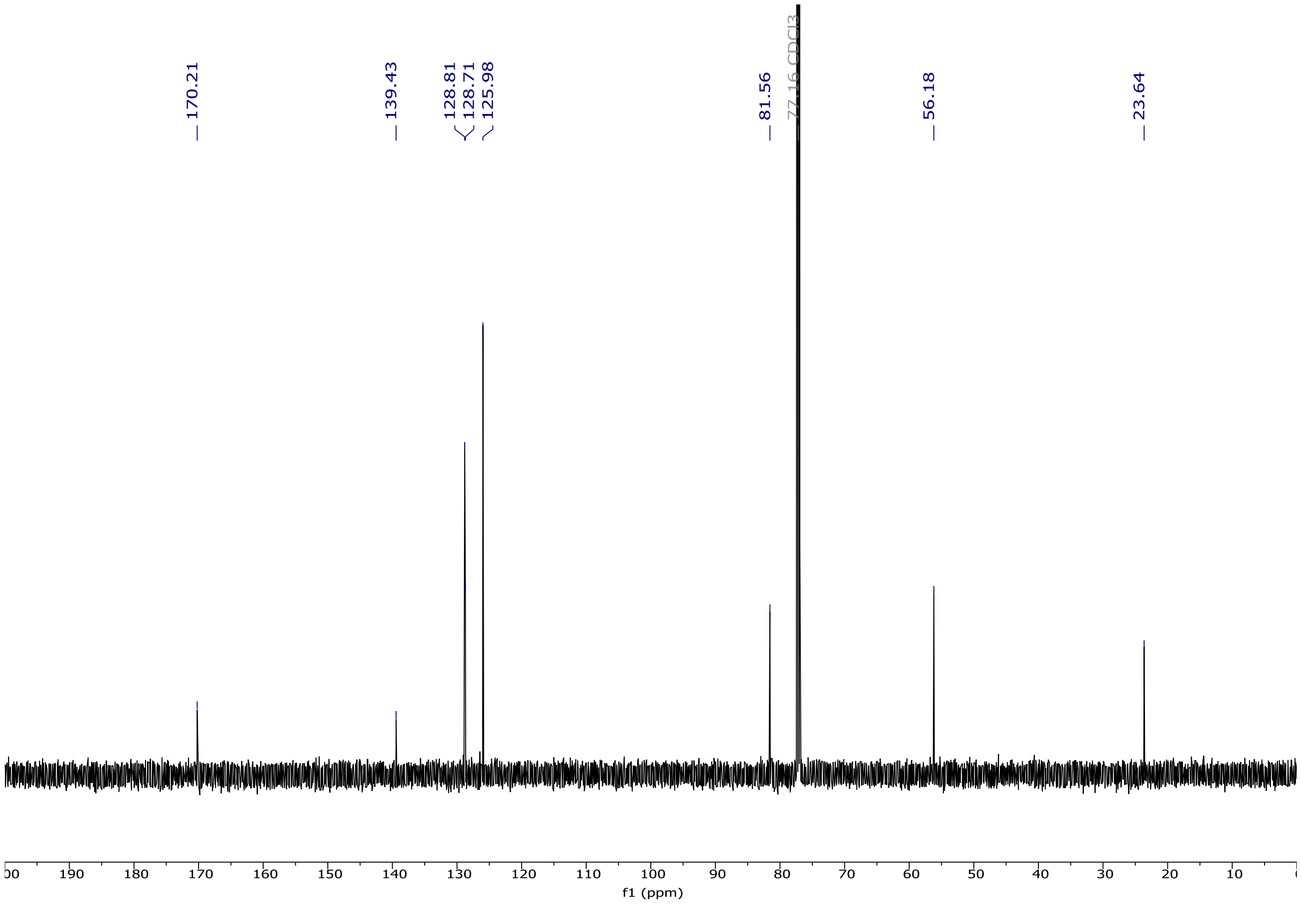

Supplementary Fig. 74 | ^{1}H (top) and ^{13}C (bottom) NMR spectra of **46.**

(*S*)-*N*-(Tetrahydrofuran-2-yl)acetamide (47)

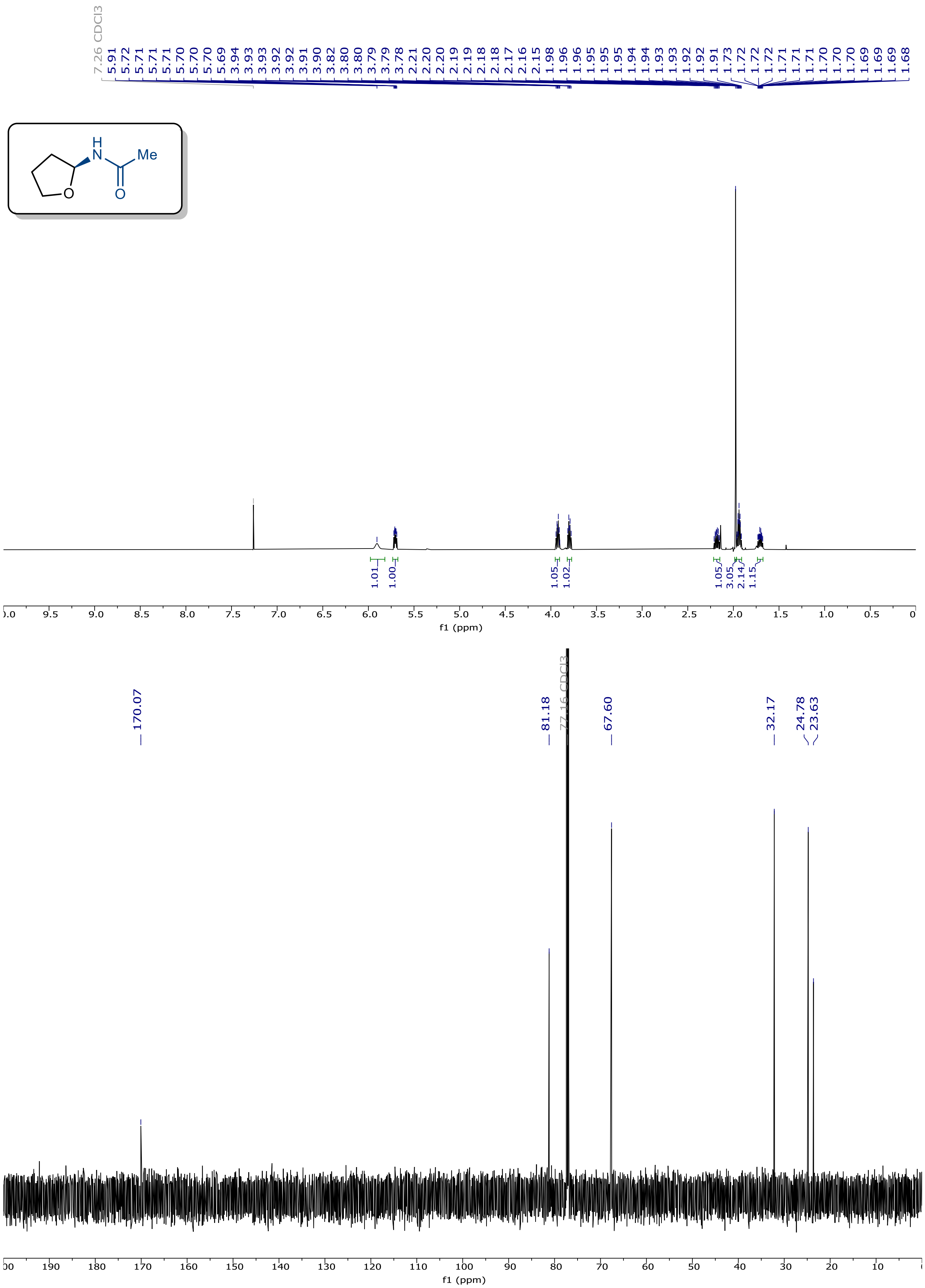

Supplementary Fig. 75 | ^{1}H (top) and ^{13}C (bottom) NMR spectra of **47.**

***tert*-Butyl (*R*)-2-acetamidopyrrolidine-1-carboxylate (48)**

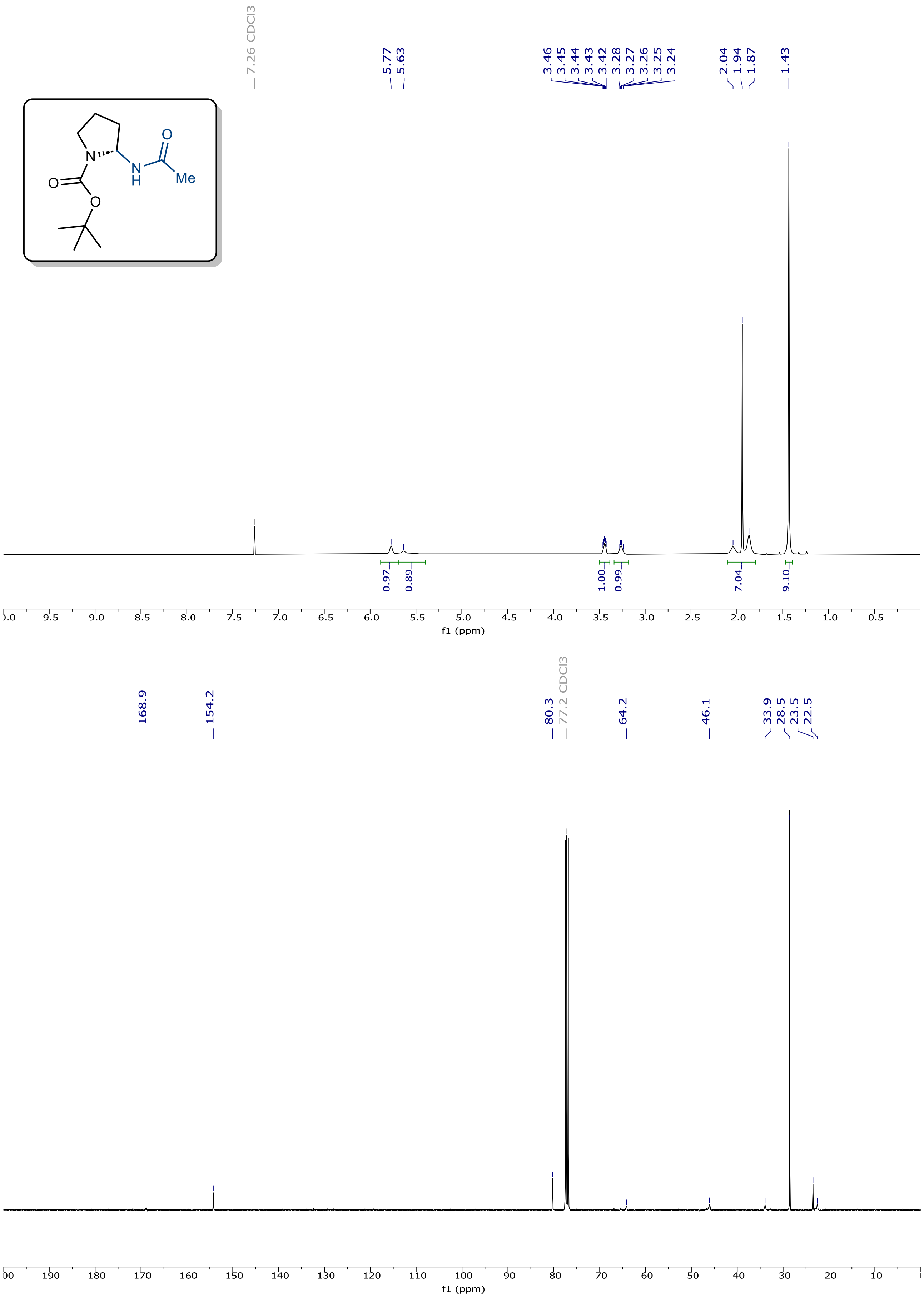

Supplementary Fig. 76 | ^{1}H (top) and ^{13}C (bottom) NMR spectra of **48.**

***tert*-Butyl {(*1R,3S*)-3-acetamidocyclohexyl}carbamate (49)**

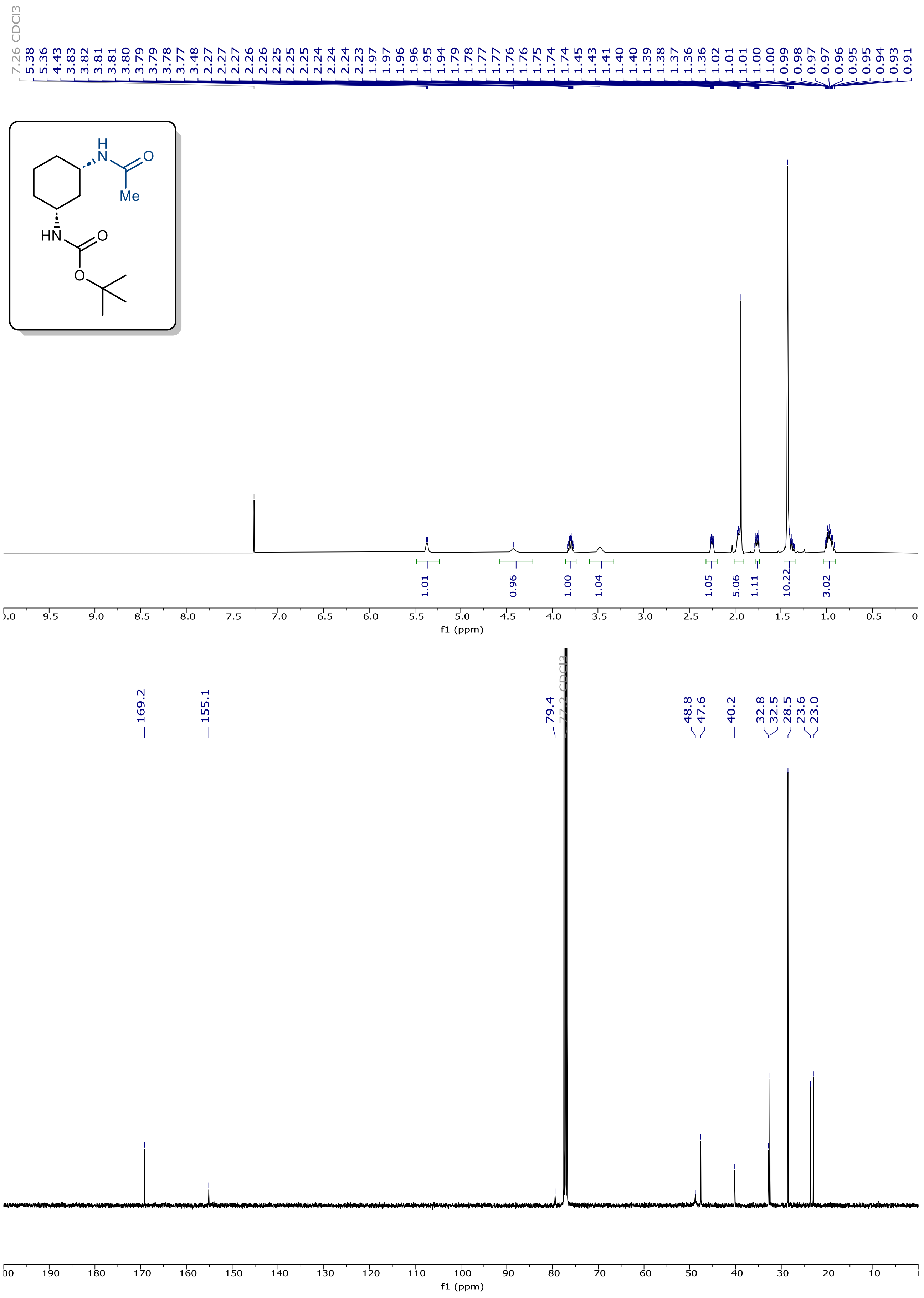

Supplementary Fig. 77 | ^{1}H (top) and ^{13}C (bottom) NMR spectra of **49.**

(*S*)-*N*-{1-(6-Methoxynaphthalen-2-yl)ethyl}acetamide (52)

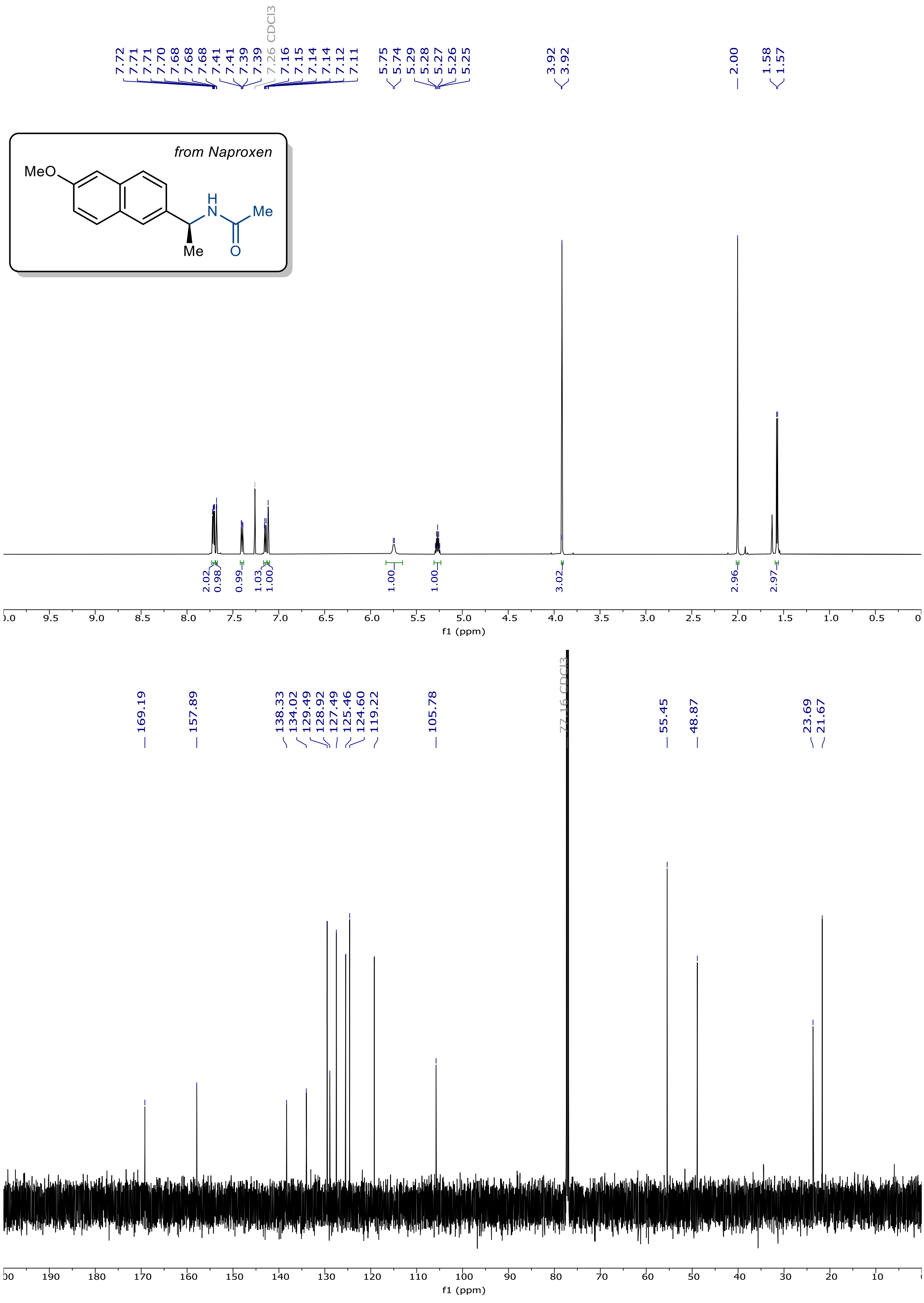

Supplementary Fig. 78 | ^{1}H (top) and ^{13}C (bottom) NMR spectra of **52.**

(S)-*N*-{1-(5-Bromo-6-methoxynaphthalen-2-yl)ethyl}acetamide (53)

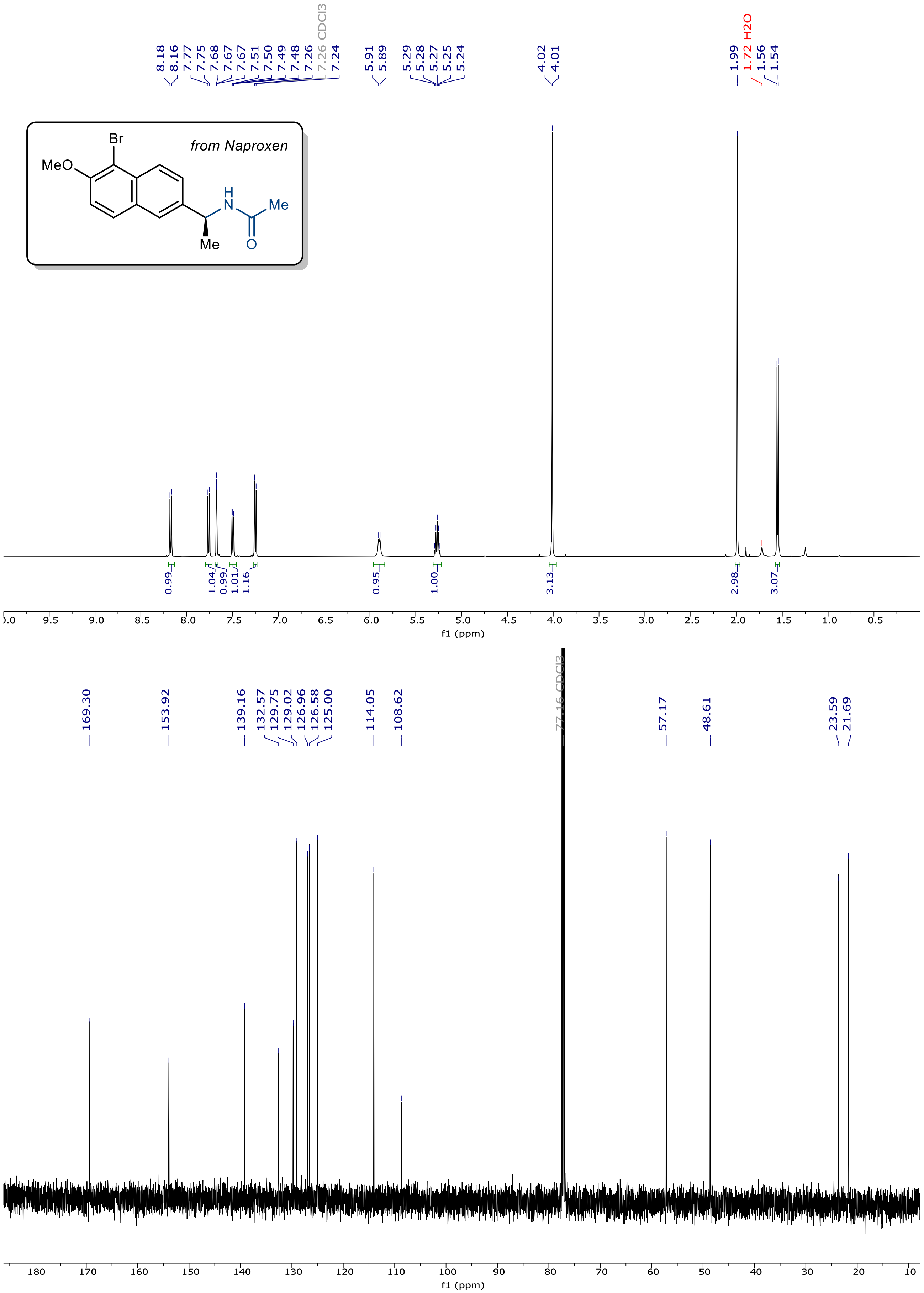

Supplementary Fig. 79 | ^{1}H (top) and ^{13}C (bottom) NMR spectra of **53.**

(*S*)-*N*-{1-(4-Isobutylphenyl)ethyl}acetamide (55)

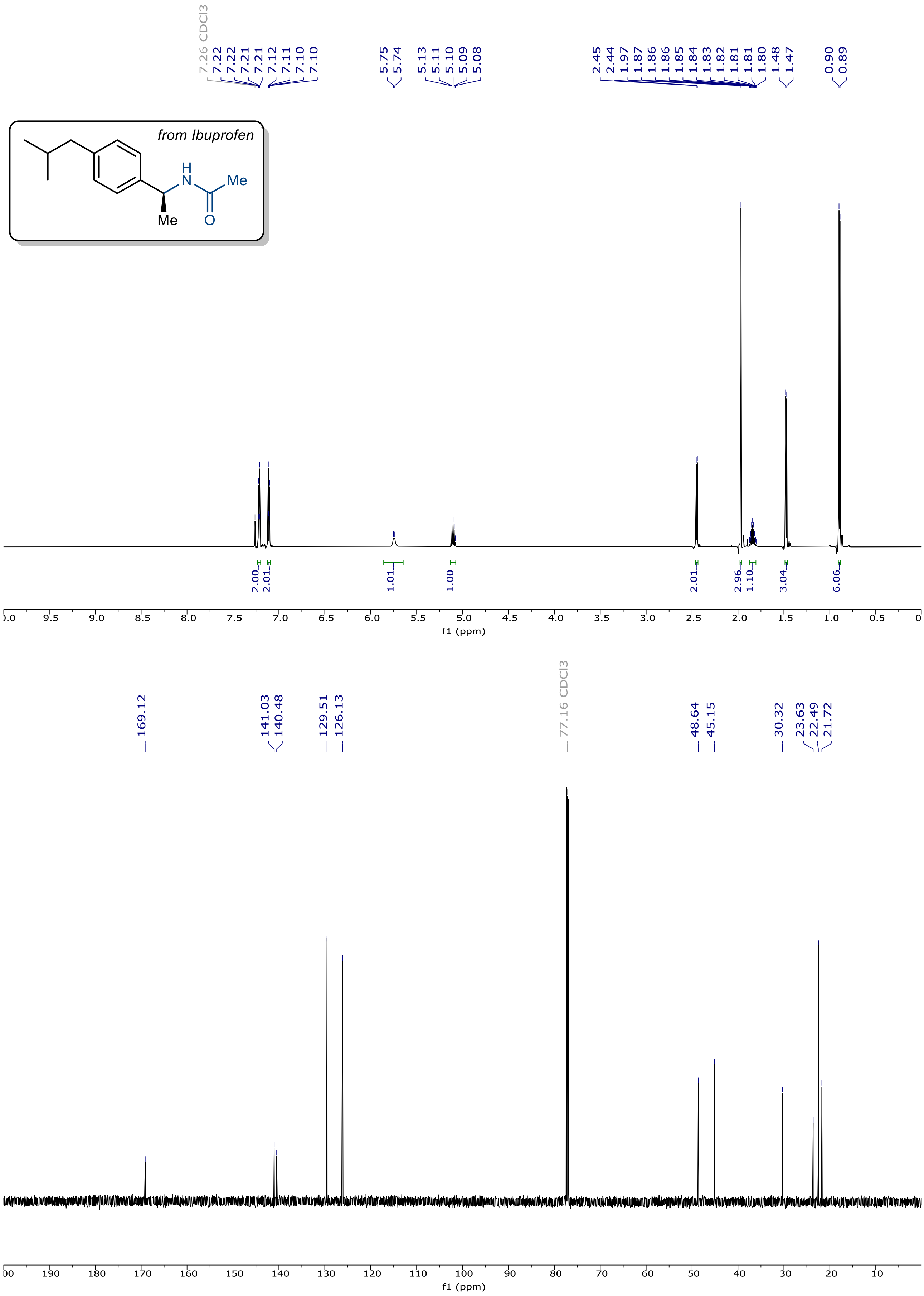

Supplementary Fig. 80 | ^{1}H (top) and ^{13}C (bottom) NMR spectra of **55.**

***tert*-Butyl [(2*S*,4*S*)-1-{(1,1'-biphenyl)-4-yl}-4-acetamidopentan-2-yl]carbamate (57)**

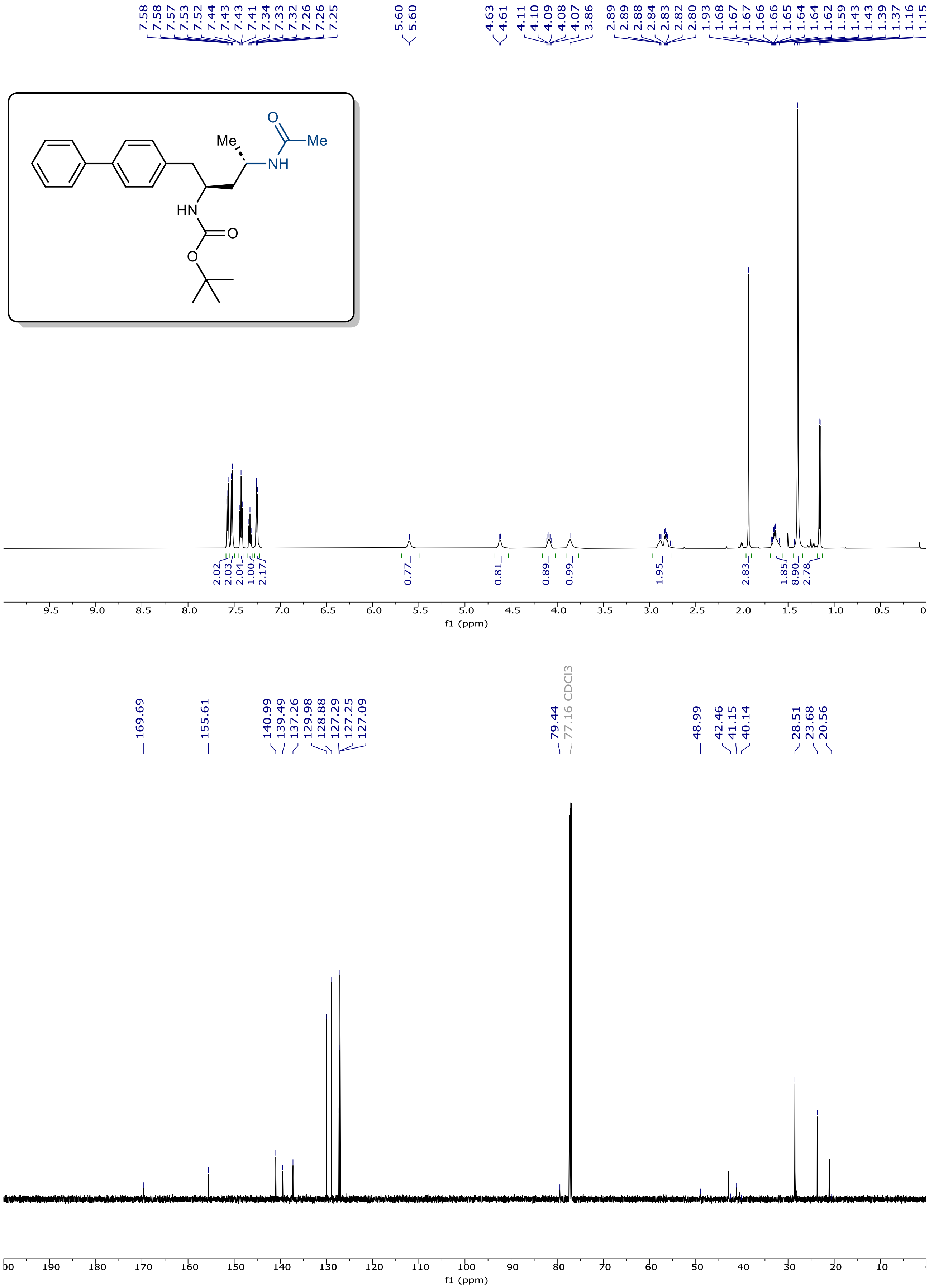

Supplementary Fig. 81 | ^{1}H (top) and ^{13}C (bottom) NMR spectra of **57.**

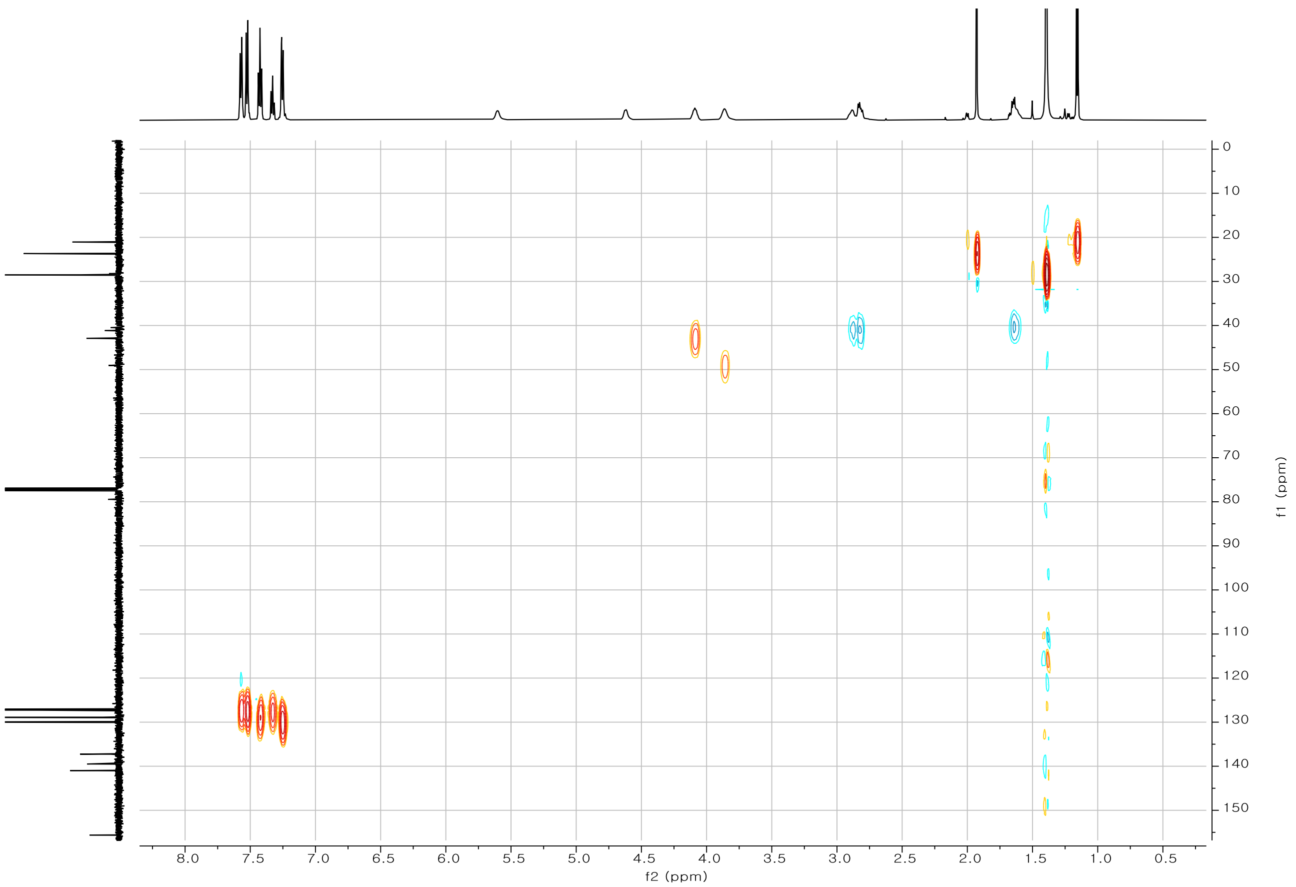

Supplementary Fig. 82 | HSQC spectra of **57.**

N-[(R)-1-{(1R,4R,4aS,8aR)-4,7-Dimethyl-1,2,3,4,4a,5,6,8a-octahydronaphthalen-1-yl}ethyl]acetamide (59)

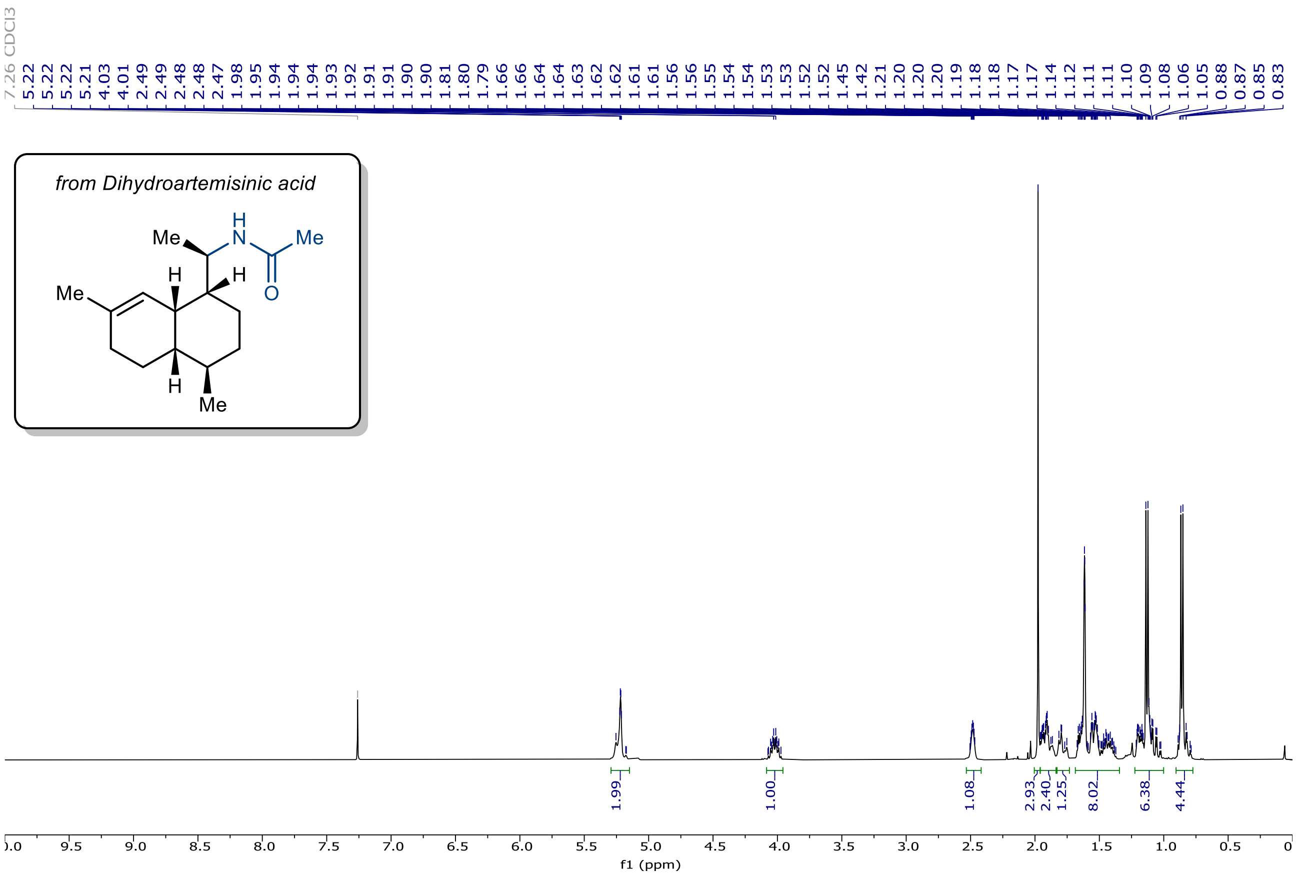

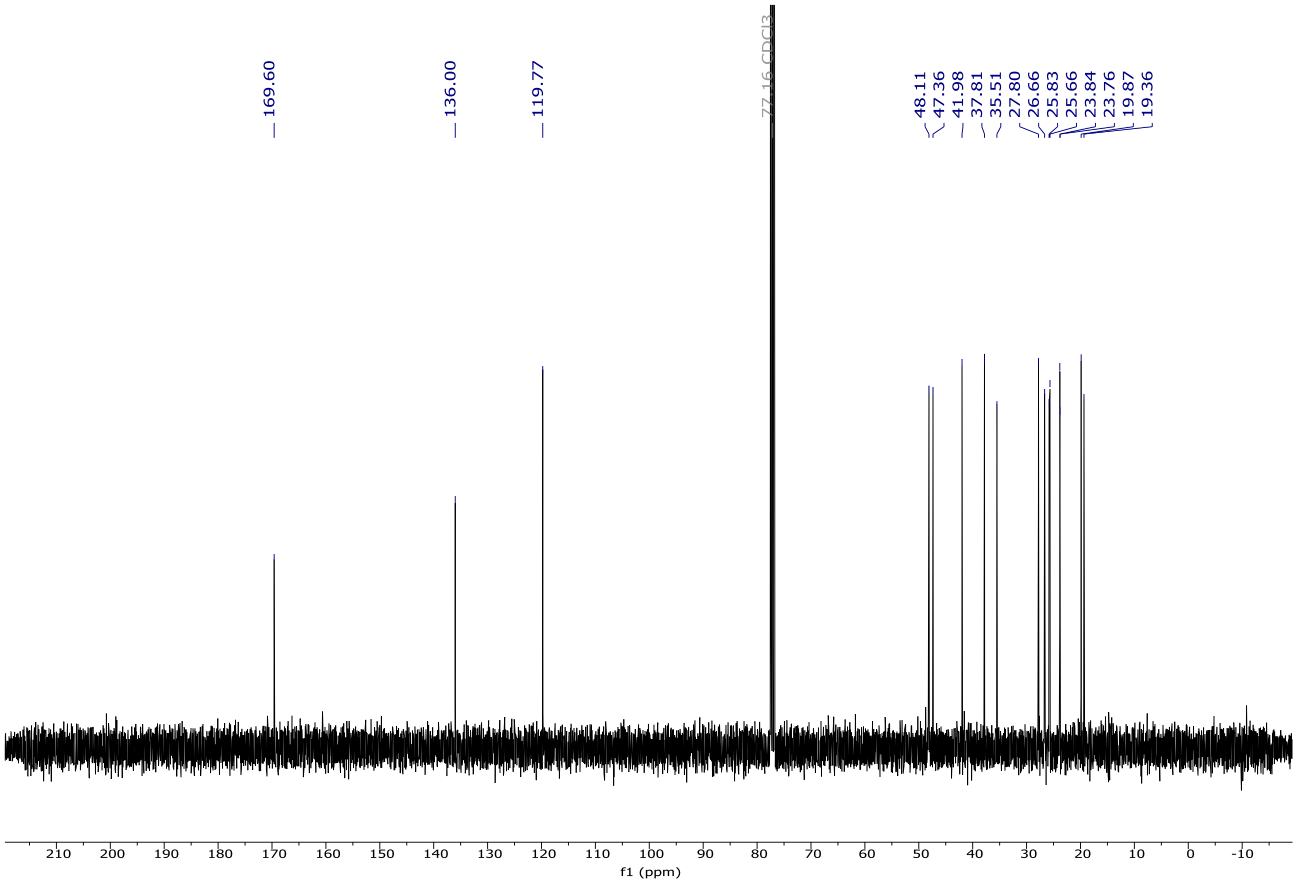

Supplementary Fig. 83 | ^{1}H (top) and ^{13}C (bottom) NMR spectra of **59.**

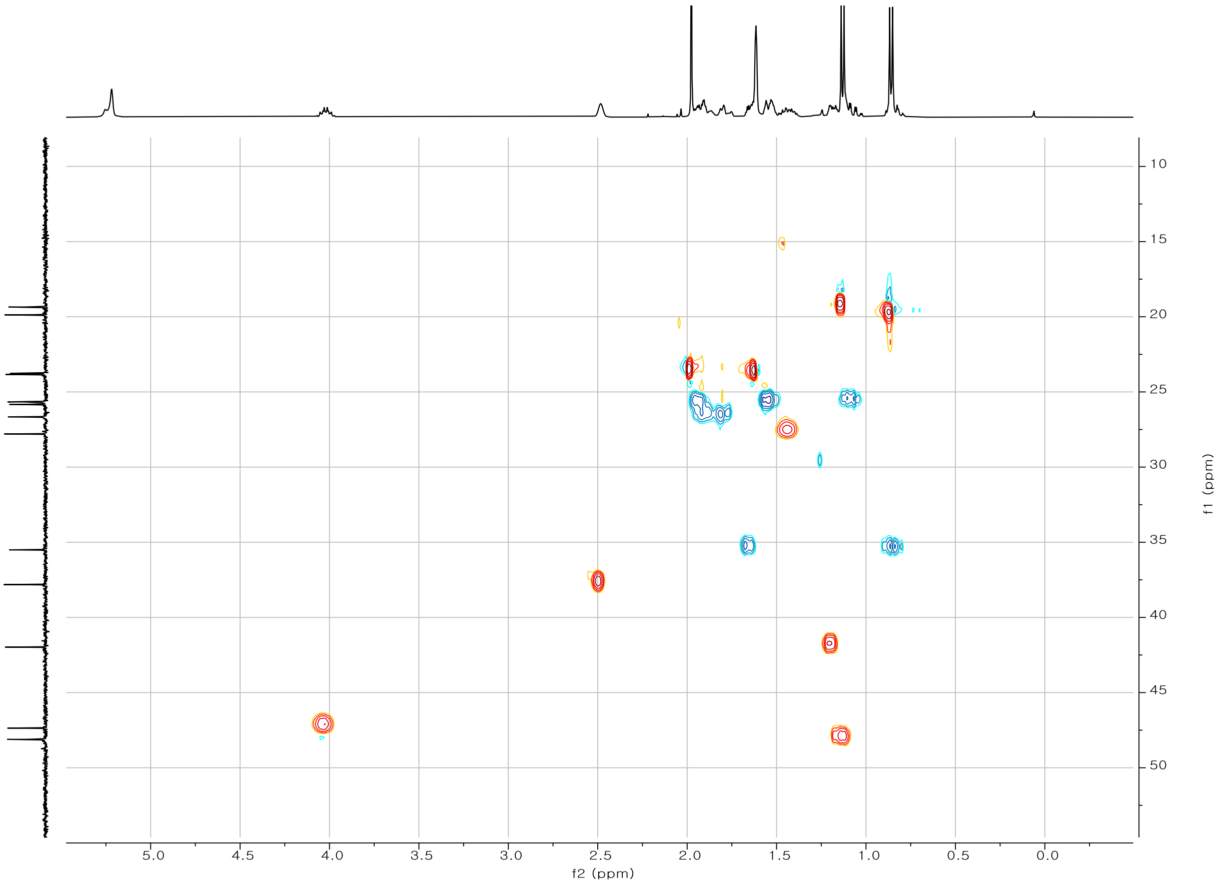

Supplementary Fig. 84 | HSQC spectra of **59.**

(*S*)-*N*-{1-(6-Methoxynaphthalen-2-yl)ethyl}-4-phenylbutanamide (60)

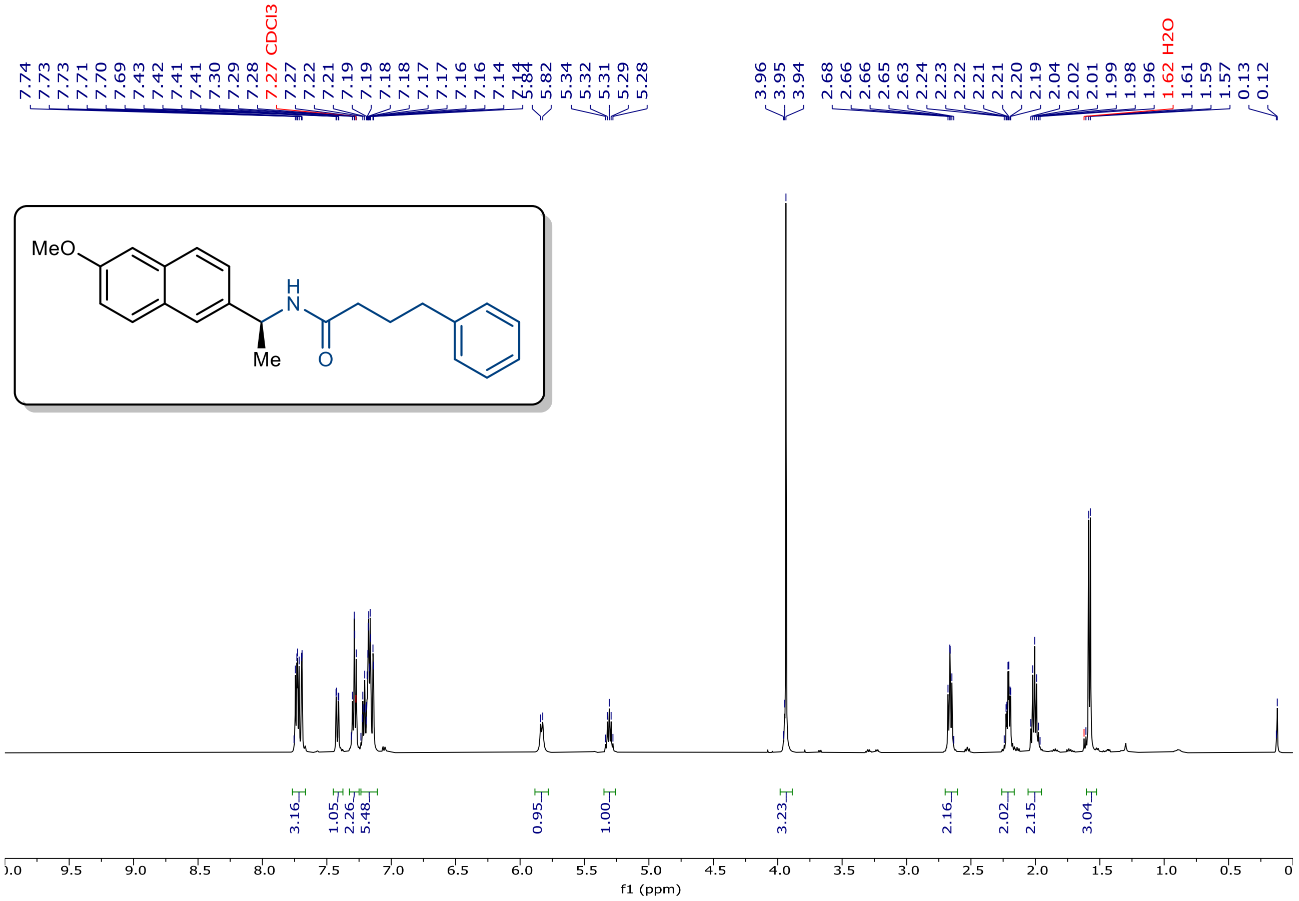

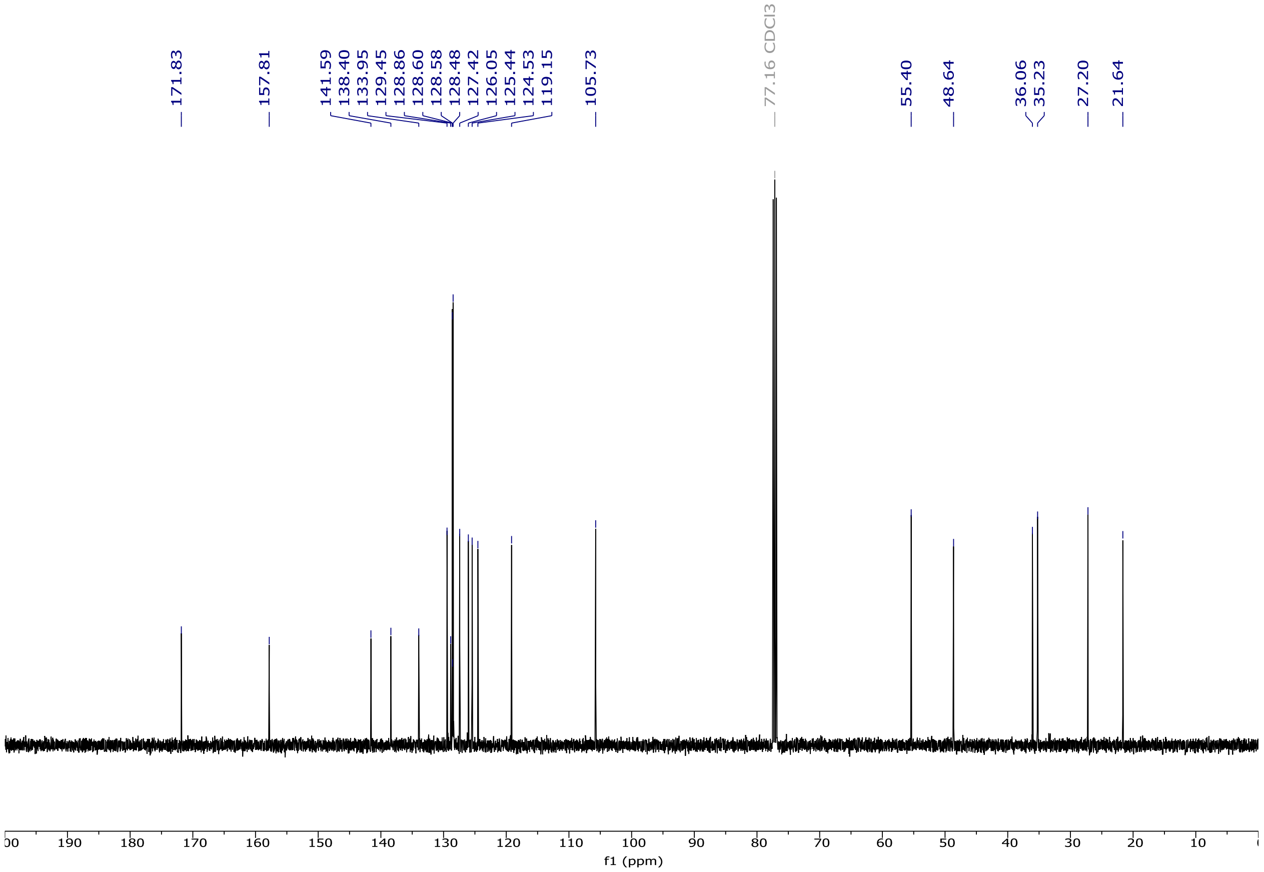

Supplementary Fig. 85 | ^{1}H (top) and ^{13}C (bottom) NMR spectra of **60.**

(*S*,*E*)-*N*-{1-(6-Methoxynaphthalen-2-yl)ethyl}-5-phenylpent-4-enamide (61)

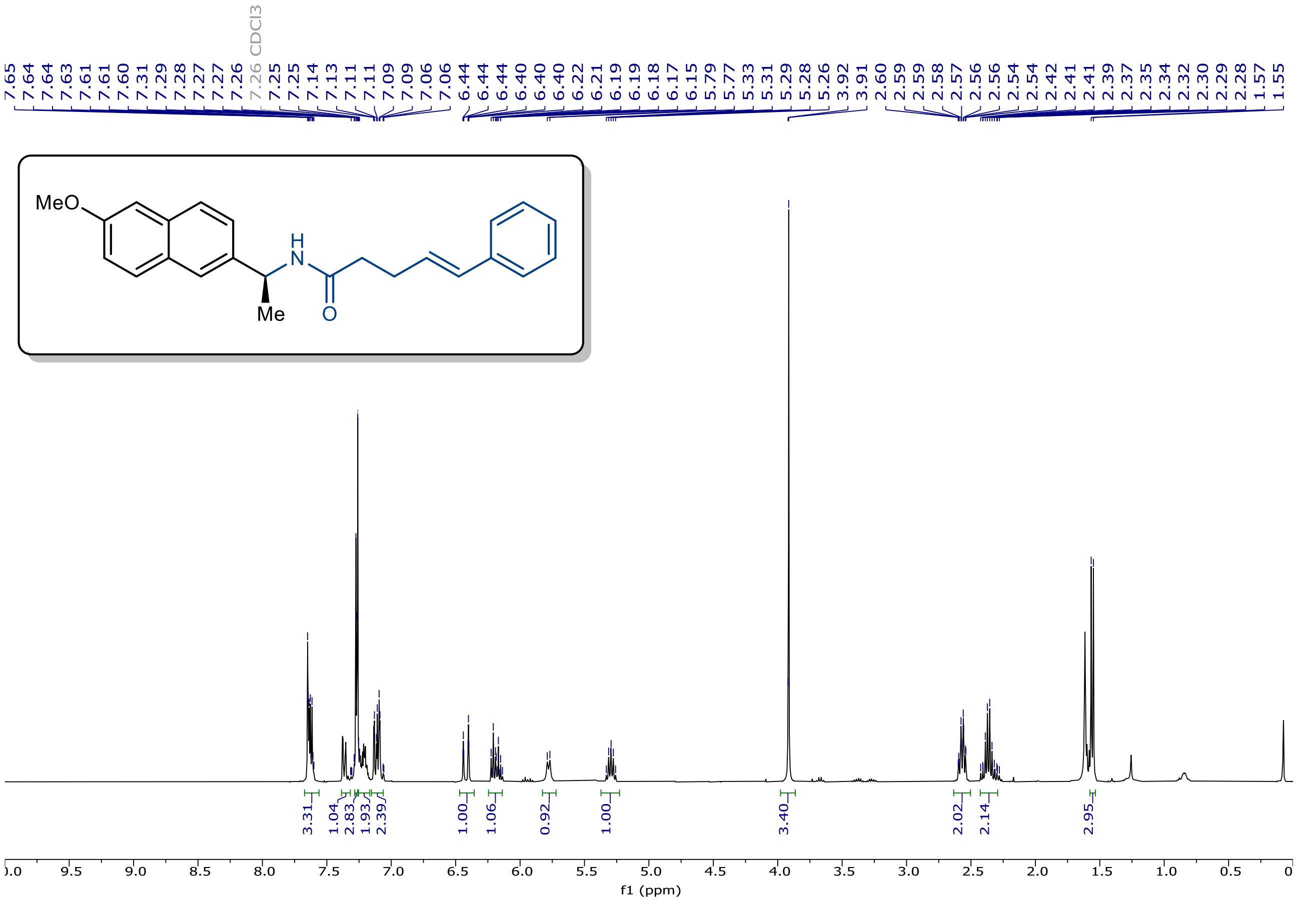

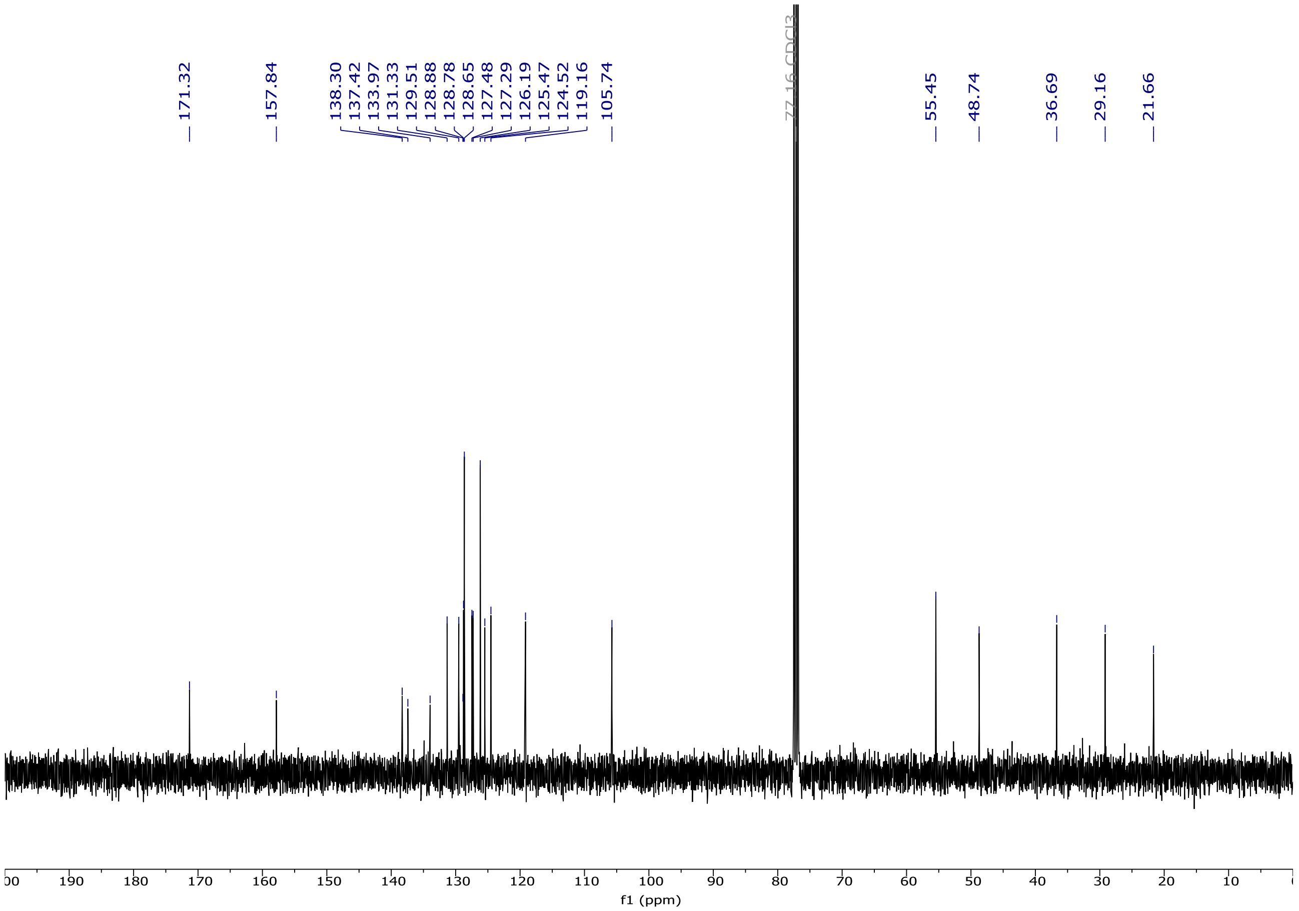

Supplementary Fig. 86 | ^{1}H (top) and ^{13}C (bottom) NMR spectra of **61.**

(*S*)-4-(5-[{1-(6-Methoxynaphthalen-2-yl)ethyl}amino]-5-oxopent-1-yn-1-yl)benzoate (62)

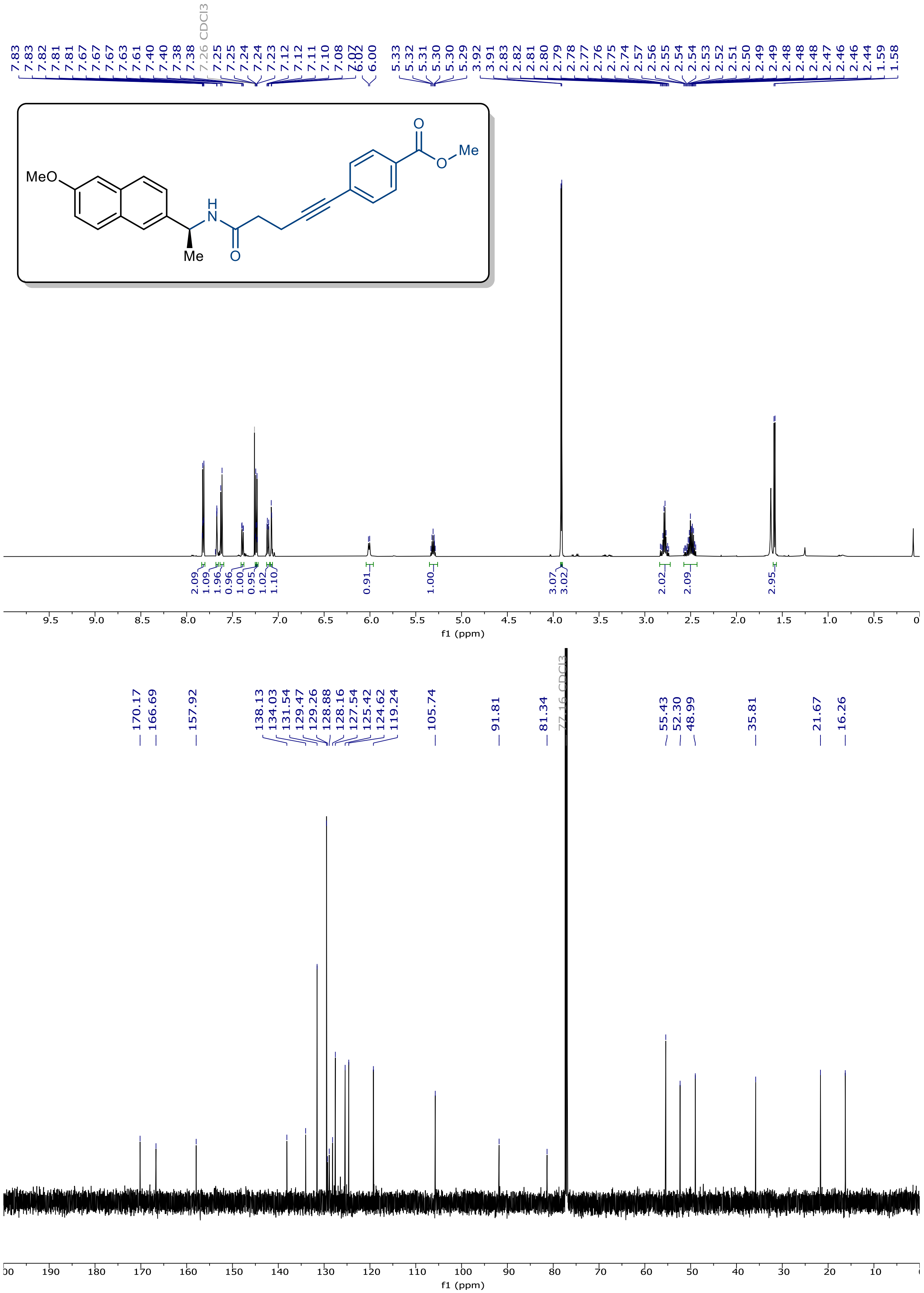

Supplementary Fig. 87 | ^{1}H (top) and ^{13}C (bottom) NMR spectra of **62.**

(*S*)-*N*-{1-(6-Methoxynaphthalen-2-yl)ethyl}cinnamamide (63)

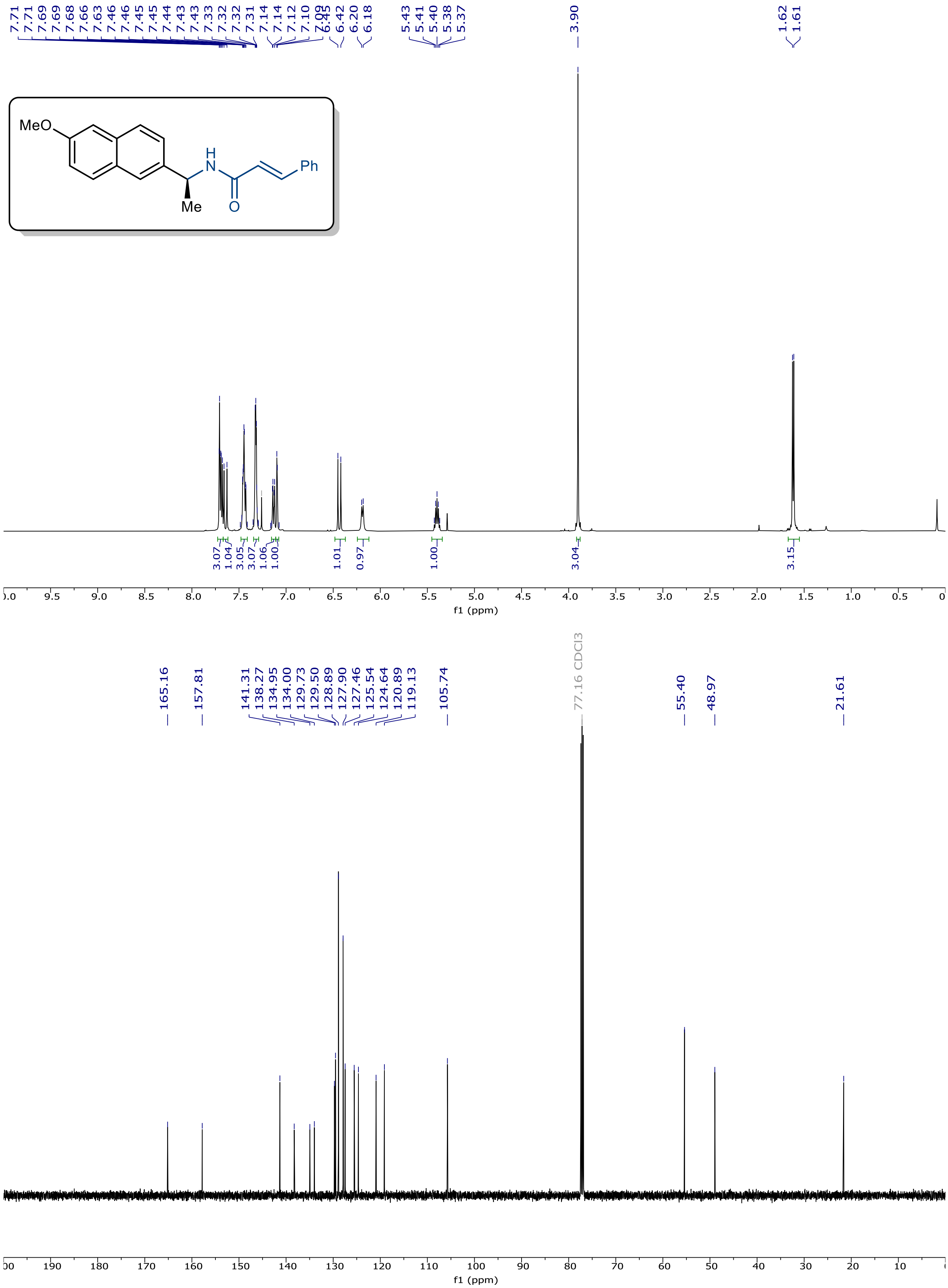

Supplementary Fig. 88 | ^{1}H (top) and ^{13}C (bottom) NMR spectra of **63.**

3-(4,5-Diphenyl-4,5-dihydrooxazol-2-yl)-*N*-{(*S*)-1-(6-methoxynaphthalen-2-yl)ethyl}propanamide (64)

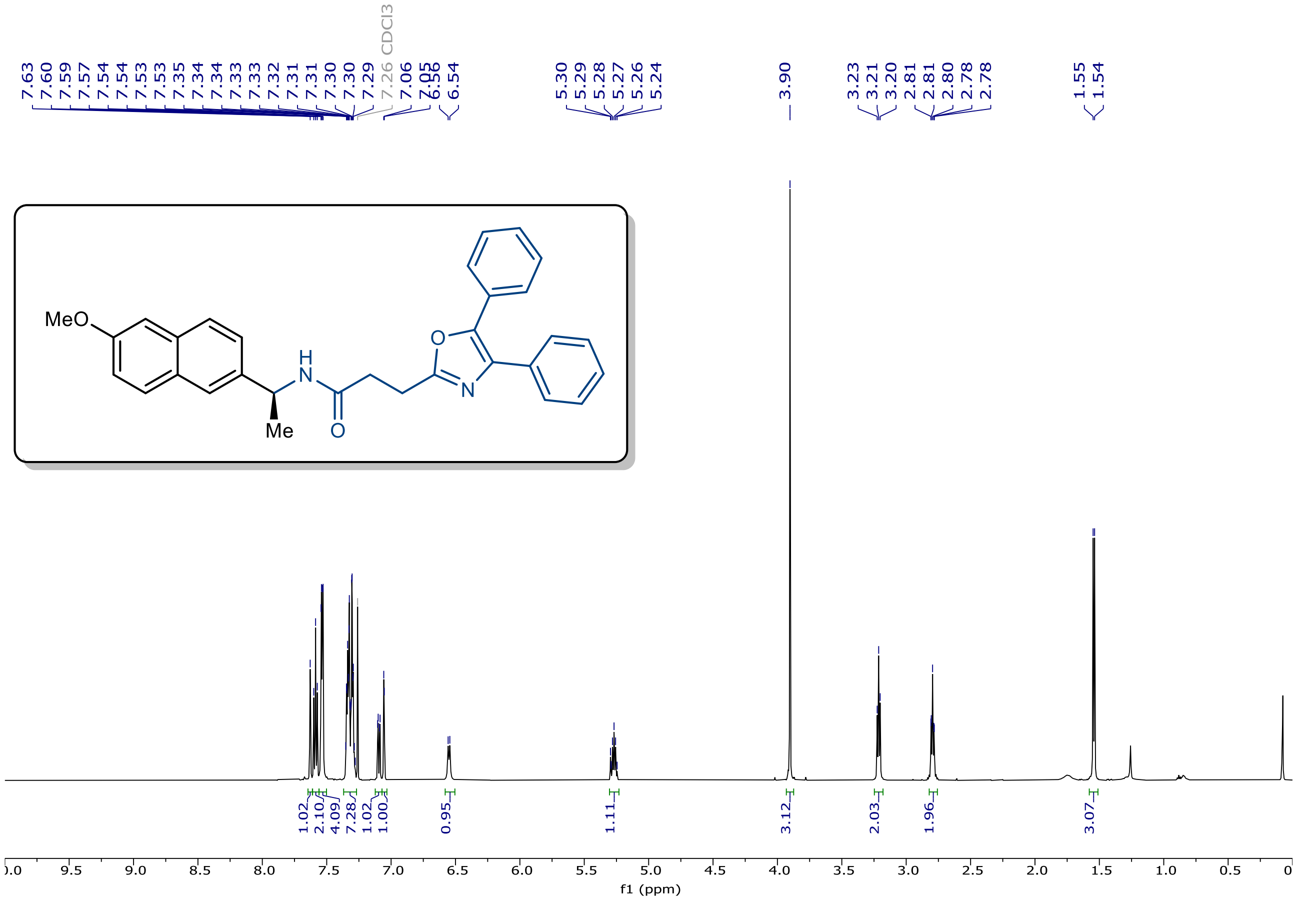

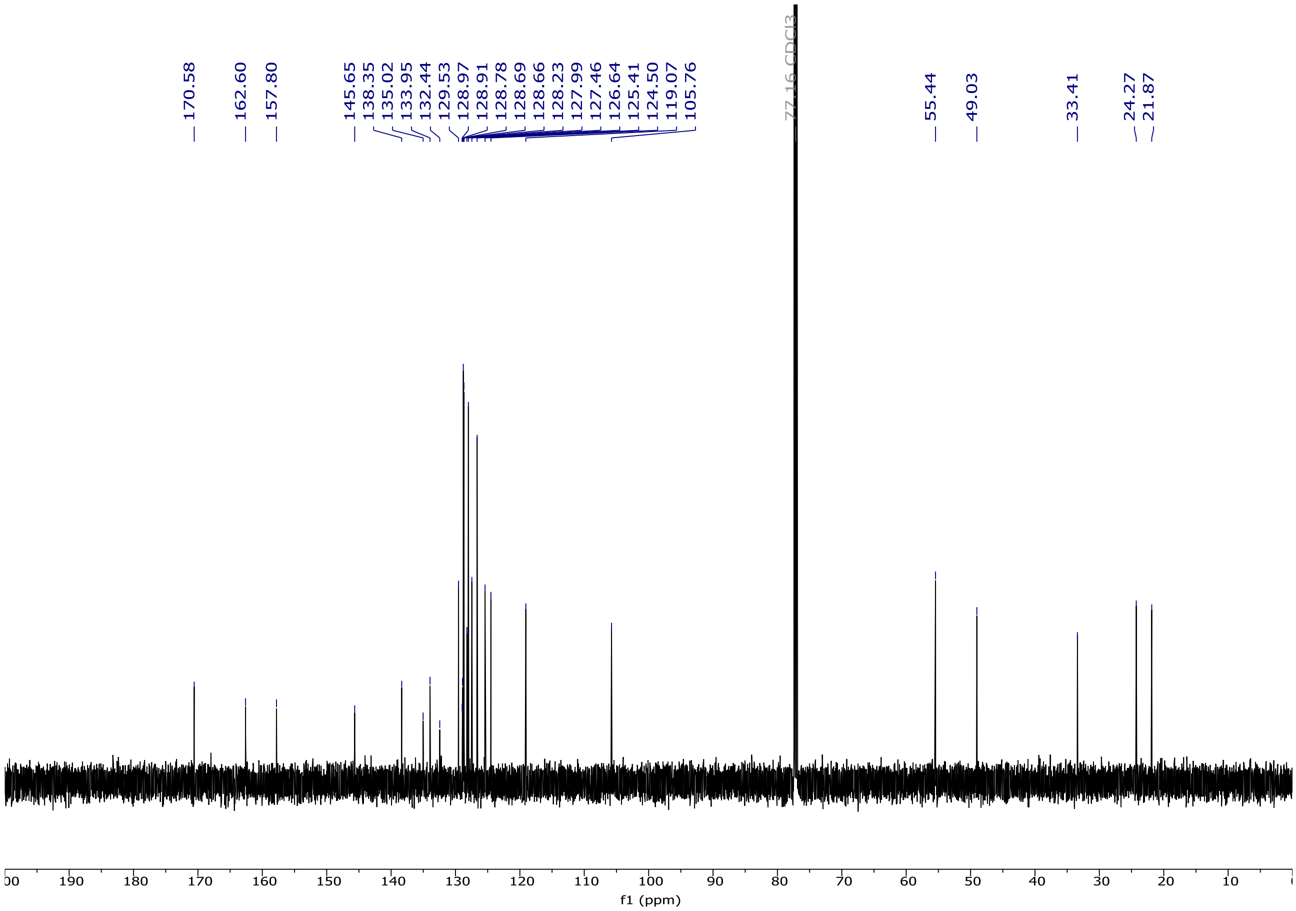

Supplementary Fig. 89 | ^{1}H (top) and ^{13}C (bottom) NMR spectra of **64.**

3-Methyl-1,4,2-dioxazol-5-one-2-^{15}N (2a-^{15}N)

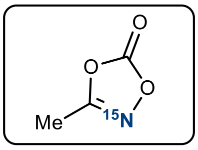

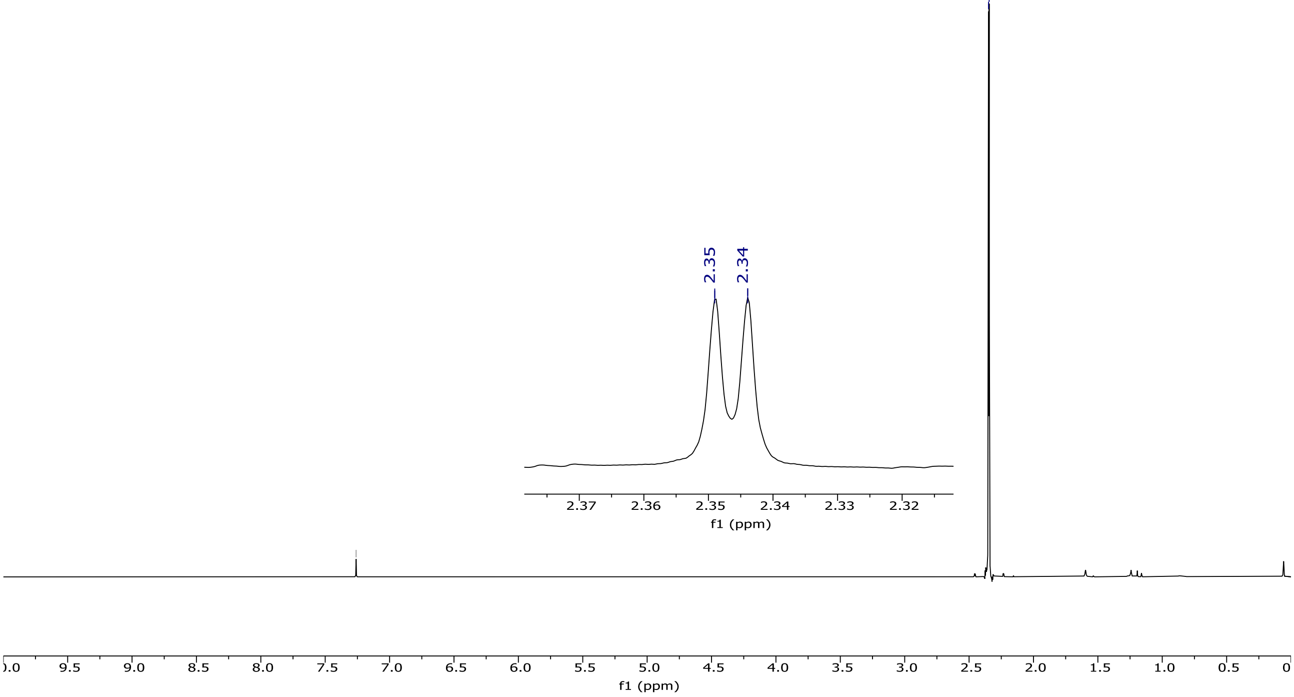

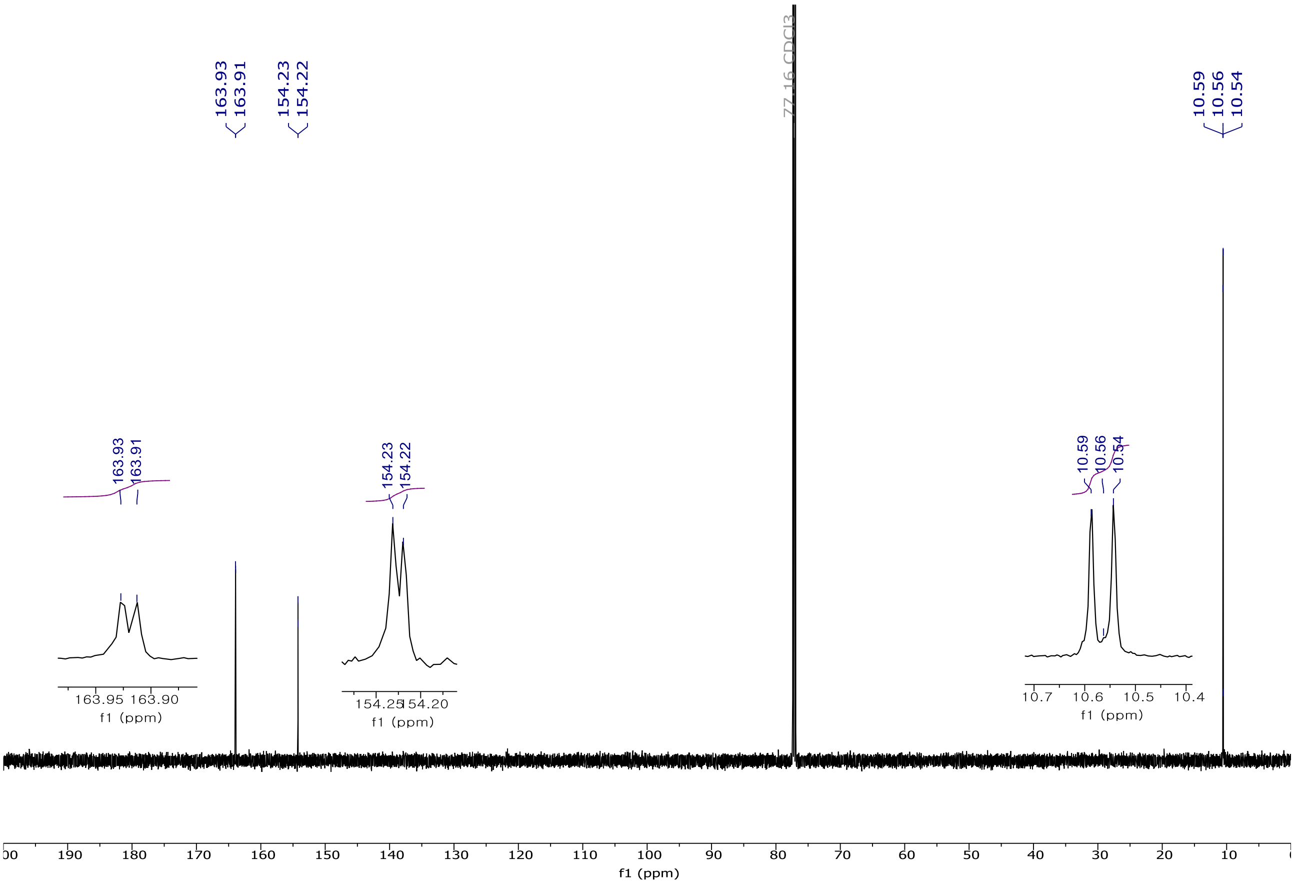

Supplementary Fig. 90 | ^{1}H (top) and ^{13}C (bottom) NMR spectra of **2a-^{15}N.**

(*S*)-*N*-{1-(6-Methoxynaphthalen-2-yl)ethyl}acetamide-^{15}N (52-^{15}N)

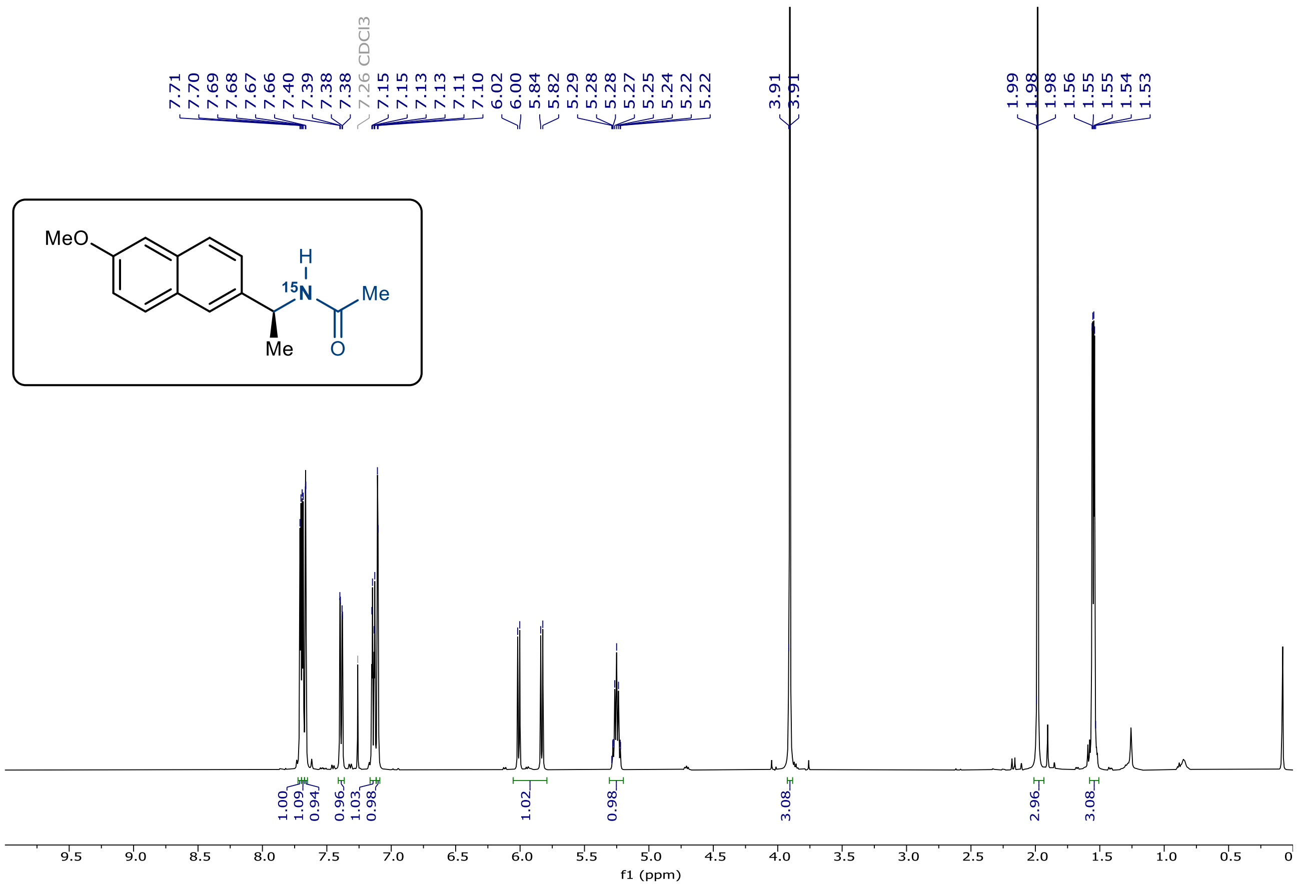

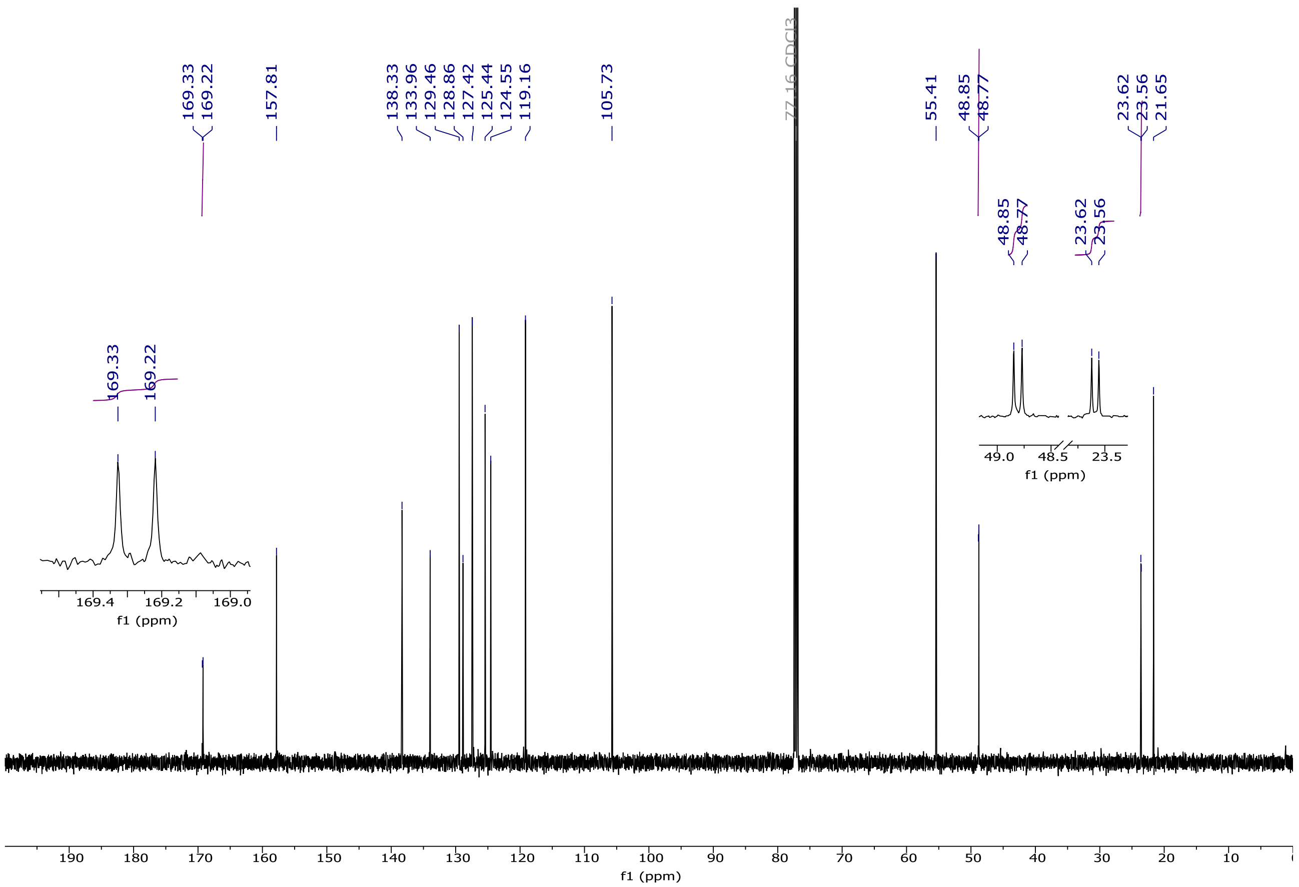

Supplementary Fig. 91 | ^{1}H (top) and ^{13}C (bottom) NMR spectra of **52-^{15}N.**

***N*-Acetyl-*N*-{(*tert*-butyldimethylsilyl)oxy}cyclohexanecarboxamide (Int3-TBS)**

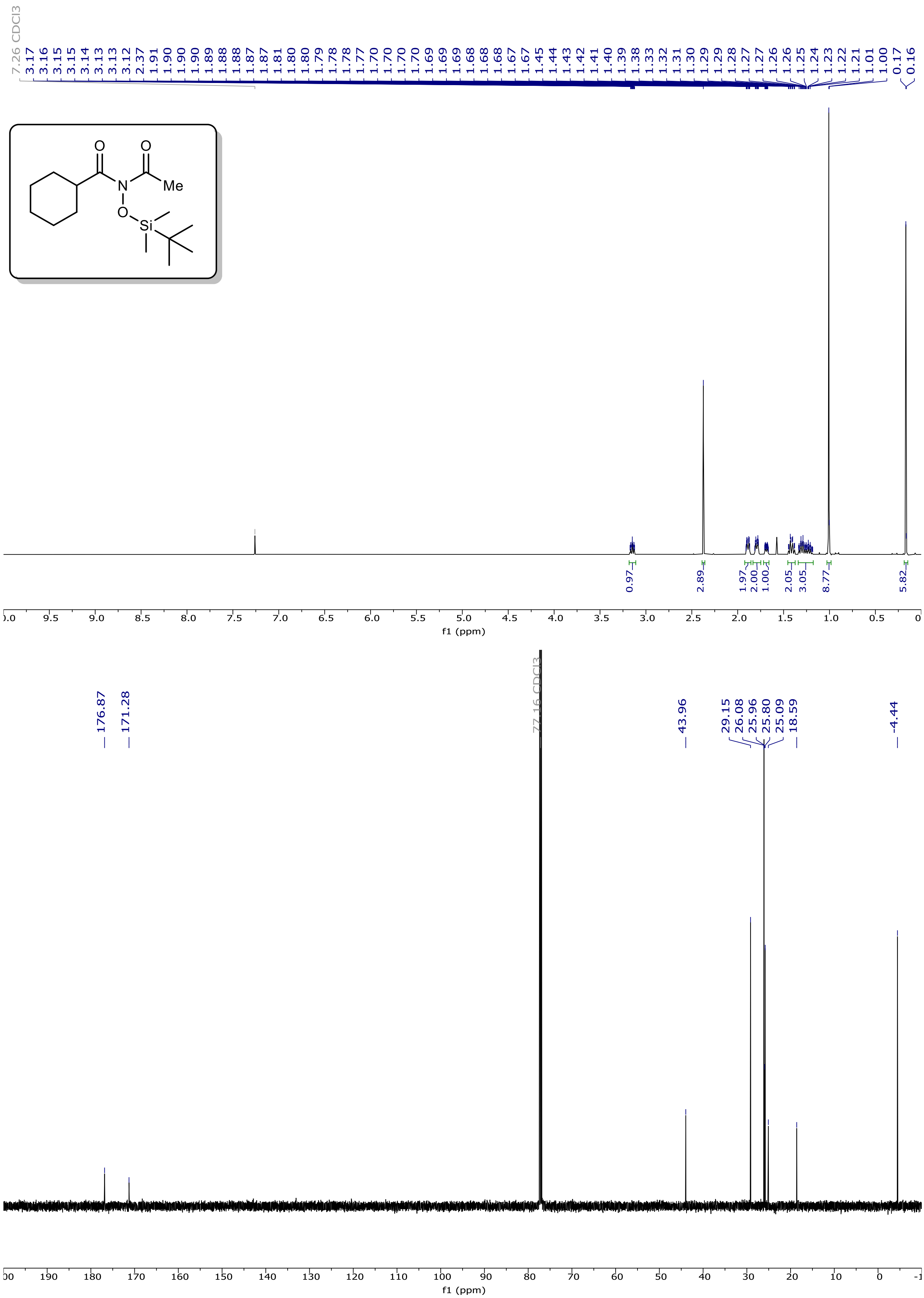

Supplementary Fig. 92 | ^{1}H (top) and ^{13}C (bottom) NMR spectra of **Int3-TBS**.

N-Hydroxycyclohexanecarboxamide

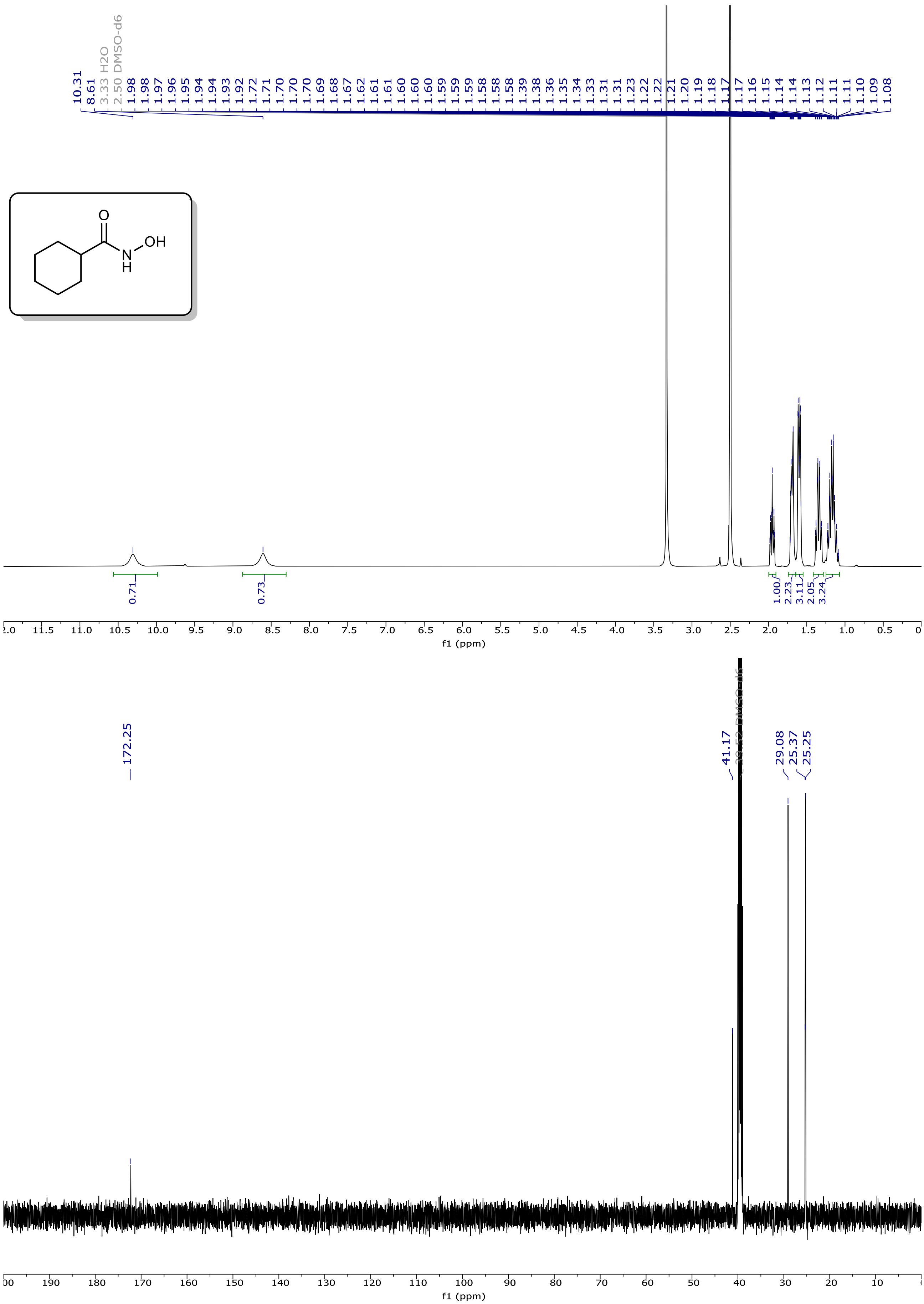

Supplementary Fig. 93 | ^{1}H (top) and ^{13}C (bottom) NMR spectra of *N*-hydroxycyclohexanecarboxamide.

***N*-Acetoxycyclohexanecarboxamide (H[int5-A]).**

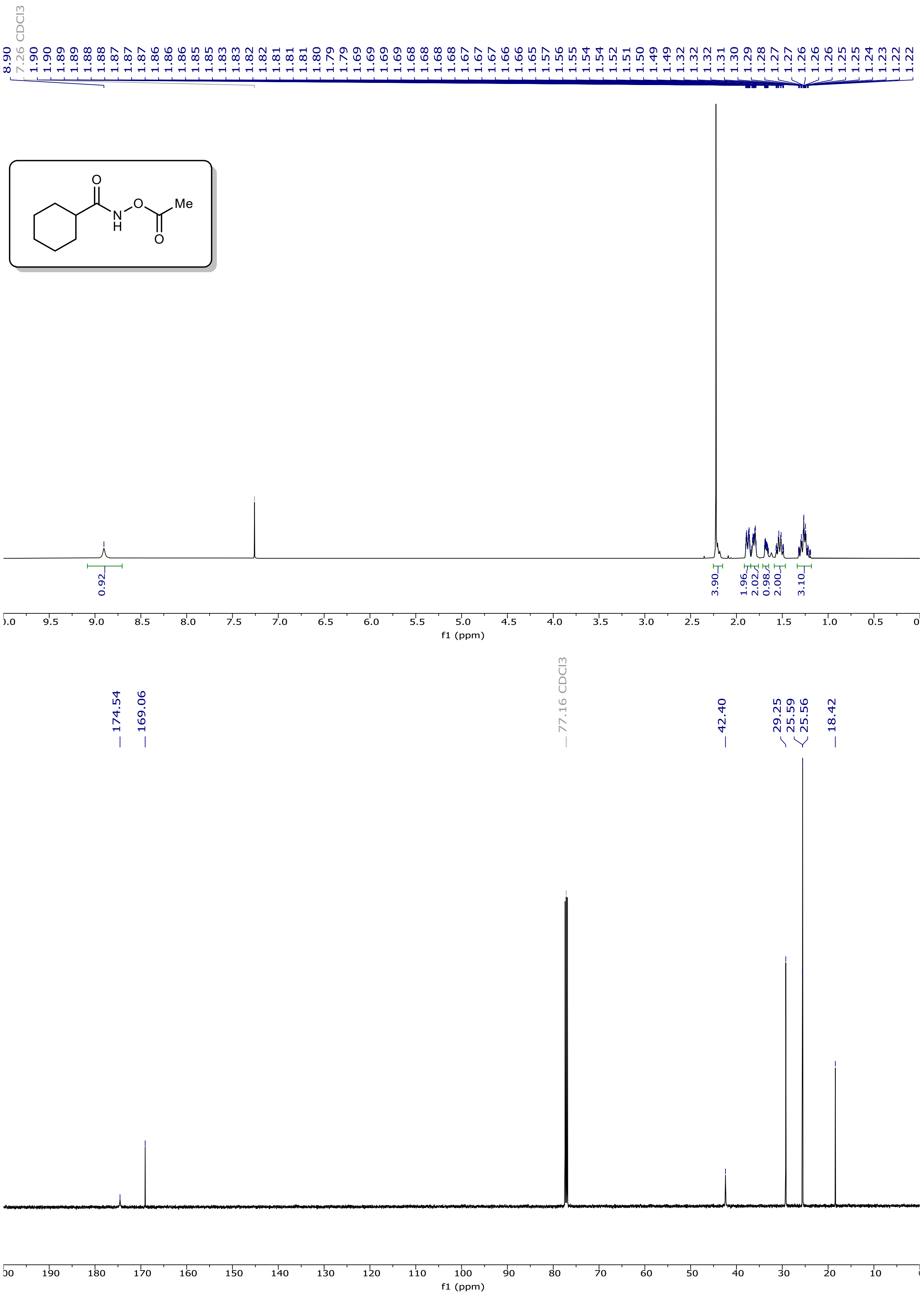

Supplementary Fig. 94 | ^{1}H (top) and ^{13}C (bottom) NMR spectra of **H[int5-A]**.

Single Crystal X-Ray Diffraction Data of
27**, **31**, **36**, **49**, **53**, **57**, and **59

Crystallographic data for 27 (CCDC 2321815)

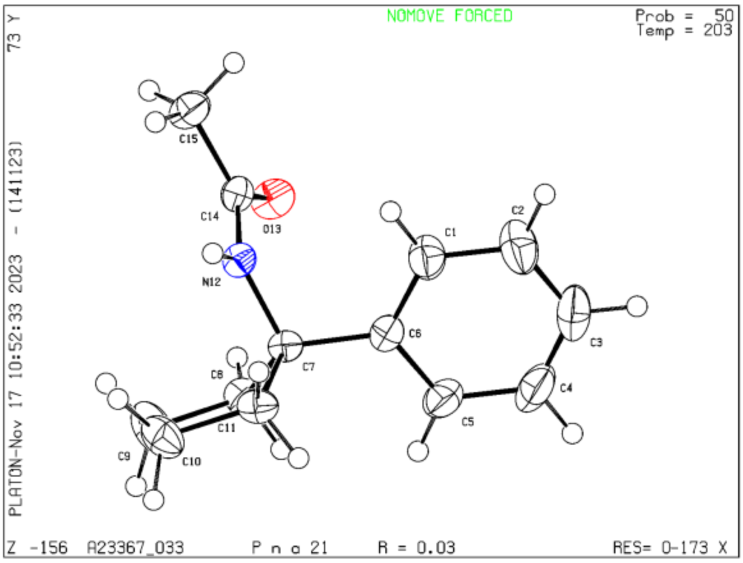

Supplementary Table 7 | Crystal data and structure refinement for **27**

Empirical formula	C_{13} H_{17} N O	
Formula weight	203.27	
Temperature	203(2) K	
Wavelength	0.71073 Å	
Crystal system	Orthorhombic	
Space group	*Pna*2_1	
Unit cell dimensions	a = 9.6363(4) Å	α = 90°
	b = 7.8110(3) Å	β = 90°
	c = 15.4153(7) Å	γ = 90°
Volume	1160.30(8) Å^3	
Z	4	
Density (calculated)	1.164 Mg/m^3	
Absorption coefficient	0.073 mm^{-1}	
F(000)	440	
Crystal size	0.153 x 0.046 x 0.034 mm^3	
Theta range for data collection	3.357 to 27.089°.	
Index ranges	–12<=h<=12, –10<=k<=9, –19<=l<=19	
Reflections collected	32469	
Independent reflections	2544 [R(int) = 0.0536]	
Completeness to theta = 25.242°	99.6 %	
Absorption correction	Semi-empirical from equivalents	
Max. and min. transmission	0.7455 and 0.6721	
Refinement method	Full-matrix least-squares on F^2	
Data / restraints / parameters	2544 / 1 / 140	
Goodness-of-fit on F^2	1.064	
Final R indices [I>2sigma(I)]	R1 = 0.0343, wR2 = 0.0858	
R indices (all data)	R1 = 0.0363, wR2 = 0.0879	
Absolute structure parameter	0.1(3)	
Largest diff. peak and hole	0.223 and –0.156 e·Å^{-3}	

Crystallographic data for 31 (CCDC 2321816)

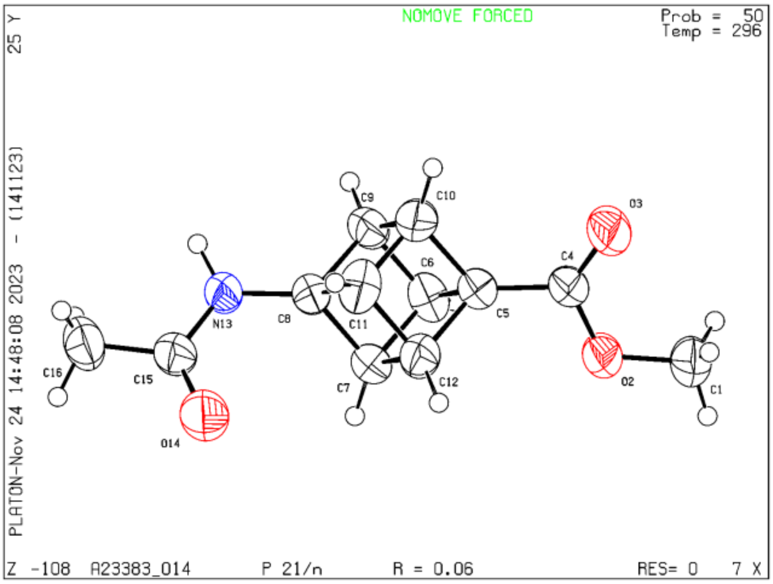

Supplementary Table S8 | Crystal data and structure refinement for **31**

Empirical formula	$C_{12}\,H_{13}\,N\,O_3$	
Formula weight	219.23	
Temperature	296(2) K	
Wavelength	0.71073 Å	
Crystal system	Monoclinic	
Space group	*P*2$_1$/*n*	
Unit cell dimensions	a = 5.8002(6) Å	α = 90°
	b = 27.722(3) Å	β = 94.178(3)°
	c = 6.9287(7) Å	γ = 90°
Volume	1111.1(2) Å^3	
Z	4	
Density (calculated)	1.311 Mg/m^3	
Absorption coefficient	0.095 mm^{-1}	
F(000)	464	
Crystal size	0.112 x 0.052 x 0.022 mm^3	
Theta range for data collection	2.939 to 26.015°.	
Index ranges	–6<=h<=7, –34<=k<=34, –8<=l<=8	
Reflections collected	12921	
Independent reflections	2182 [R(int) = 0.1056]	
Completeness to theta = 25.242°	99.7 %	
Absorption correction	Semi-empirical from equivalents	
Max. and min. transmission	0.7453 and 0.6530	
Refinement method	Full-matrix least-squares on F^2	
Data / restraints / parameters	2182 / 0 / 150	
Goodness-of-fit on F^2	1.009	
Final R indices [I>2sigma(I)]	R1 = 0.0565, wR2 = 0.1185	
R indices (all data)	R1 = 0.1111, wR2 = 0.1435	
Largest diff. peak and hole	0.161 and –0.208 e·Å^{-3}	

Crystallographic data for 36 (CCDC 2321817)

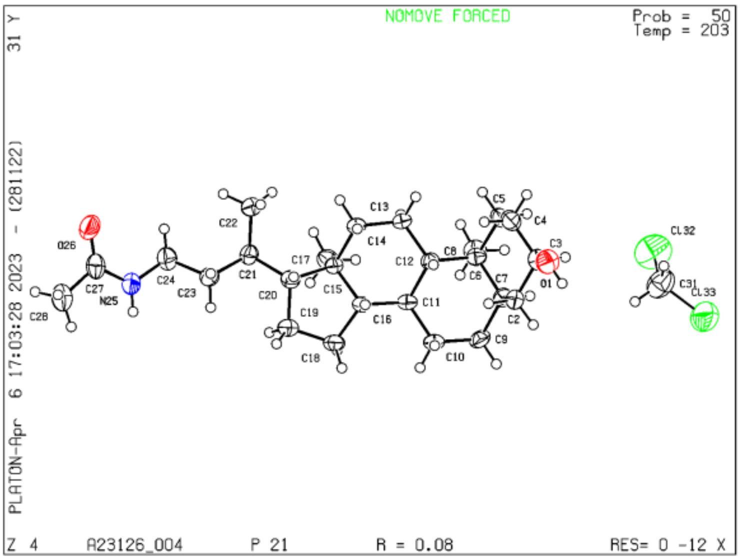

Supplementary Table 9 | Crystal data and structure refinement for **36**

Empirical formula	$C_{25.63}$ $H_{44.26}$ $Cl_{1.26}$ N O_2	
Formula weight	443.10	
Temperature	203(2) K	
Wavelength	0.71073 Å	
Crystal system	Monoclinic	
Space group	$P2_1$	
Unit cell dimensions	a = 12.8619(10) Å	α = 90°
	b = 7.5367(7) Å	β = 115.895(2)°
	c = 14.6629(11) Å	γ = 90°
Volume	1278.65(18) $Å^3$	
Z	2	
Density (calculated)	1.151 Mg/m^3	
Absorption coefficient	0.197 mm^{-1}	
F(000)	485	
Crystal size	0.261 x 0.031 x 0.029 mm^3	
Theta range for data collection	3.089 to 27.047°.	
Index ranges	–14<=h<=16, –9<=k<=9, –16<=l<=18	
Reflections collected	14768	
Independent reflections	5409 [R(int) = 0.0955]	
Completeness to theta = 25.242°	99.2 %	
Absorption correction	Semi-empirical from equivalents	
Max. and min. transmission	0.7455 and 0.6416	
Refinement method	Full-matrix least-squares on F^2	
Data / restraints / parameters	5409 / 1 / 285	
Goodness-of-fit on F^2	1.053	
Final R indices [I>2sigma(I)]	R1 = 0.0813, wR2 = 0.1793	
R indices (all data)	R1 = 0.1433, wR2 = 0.2096	
Absolute structure parameter	0.14(9)	
Largest diff. peak and hole	0.606 and –0.235 e·$Å^{-3}$	

Crystallographic data for 49 (CCDC 2321818)

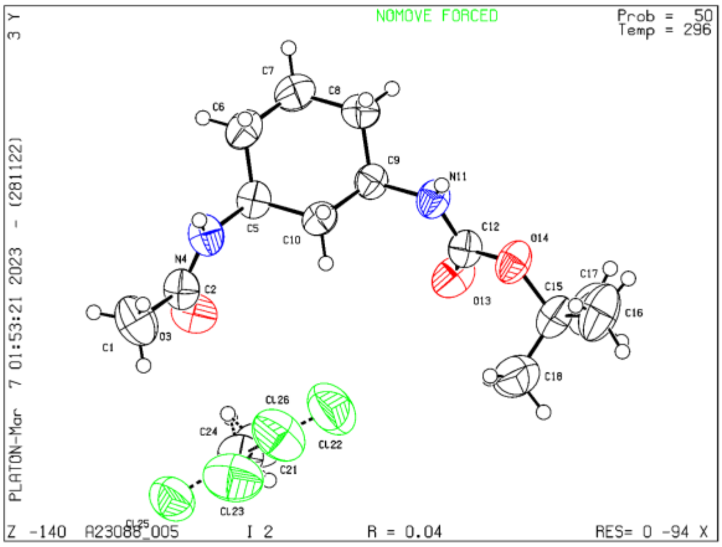

Supplementary Table 10 | Crystal data and structure refinement for **49**

Empirical formula	$C_{27} H_{50} Cl_2 N_4 O_6$	
Formula weight	597.61	
Temperature	296(2) K	
Wavelength	0.71073 Å	
Crystal system	Monoclinic	
Space group	*I*2	
Unit cell dimensions	a = 11.9383(6) Å	α = 90°
	b = 8.5803(4) Å	β = 103.5446(14)°
	c = 17.5385(11) Å	γ = 90°
Volume	1746.57(16) $Å^3$	
Z	2	
Density (calculated)	1.136 Mg/m^3	
Absorption coefficient	0.226 mm^{-1}	
F(000)	644	
Crystal size	0.154 x 0.034 x 0.028 mm^3	
Theta range for data collection	2.657 to 27.470°.	
Index ranges	–15<=h<=15, –11<=k<=11, –22<=l<=22	
Reflections collected	22119	
Independent reflections	3992 [R(int) = 0.0817]	
Completeness to theta = 25.242°	99.7 %	
Absorption correction	Semi-empirical from equivalents	
Max. and min. transmission	0.7456 and 0.6439	
Refinement method	Full-matrix least-squares on F^2	
Data / restraints / parameters	3992 / 97 / 228	
Goodness-of-fit on F^2	1.017	
Final R indices [I>2sigma(I)]	R1 = 0.0408, wR2 = 0.0941	
R indices (all data)	R1 = 0.0737, wR2 = 0.1090	
Absolute structure parameter	-0.01(5)	
Largest diff. peak and hole	0.096 and –0.109 e·$Å^{-3}$	

Crystallographic data for 53 (CCDC 2321819)

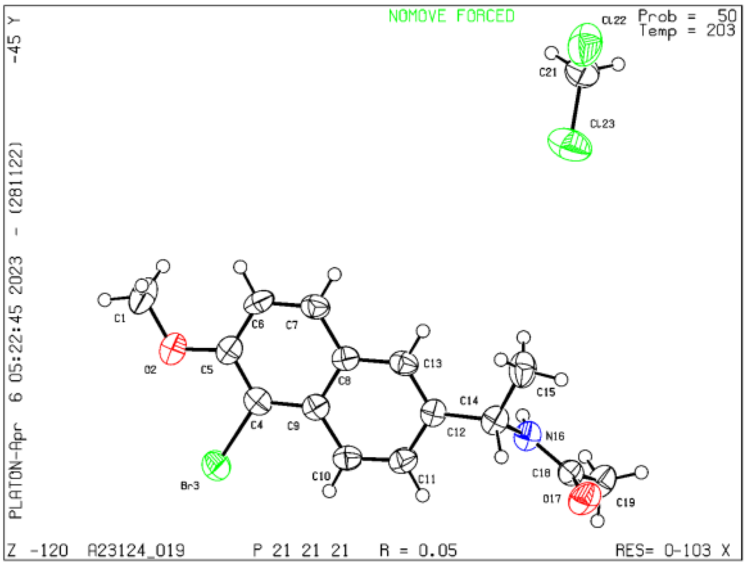

Supplementary Table 11 | Crystal data and structure refinement for **53**

Empirical formula	C_{16} H_{18} Br Cl_2 N O_2	
Formula weight	407.12	
Temperature	203(2) K	
Wavelength	0.71073 Å	
Crystal system	Orthorhombic	
Space group	$P2_12_12_1$	
Unit cell dimensions	a = 4.8463(5) Å	α = 90°
	b = 12.8625(13) Å	β = 90°
	c = 27.987(3) Å	γ = 90°
Volume	1744.6(3) $Å^3$	
Z	4	
Density (calculated)	1.550 Mg/m^3	
Absorption coefficient	2.668 mm^{-1}	
F(000)	824	
Crystal size	0.131 x 0.025 x 0.023 mm^3	
Theta range for data collection	2.697 to 27.044°.	
Index ranges	–6<=h<=6, –16<=k<=16, –35<=l<=35	
Reflections collected	19468	
Independent reflections	3807 [R(int) = 0.0901]	
Completeness to theta = 25.242°	99.8 %	
Absorption correction	Semi-empirical from equivalents	
Max. and min. transmission	0.7455 and 0.5577	
Refinement method	Full-matrix least-squares on F^2	
Data / restraints / parameters	3807 / 0 / 209	
Goodness-of-fit on F^2	1.046	
Final R indices [I>2sigma(I)]	R1 = 0.0524, wR2 = 0.0816	
R indices (all data)	R1 = 0.0919, wR2 = 0.0900	
Absolute structure parameter	0.064(18)	
Largest diff. peak and hole	0.392 and –0.443 e·$Å^{-3}$	

Crystallographic data for 57 (CCDC 2321820)

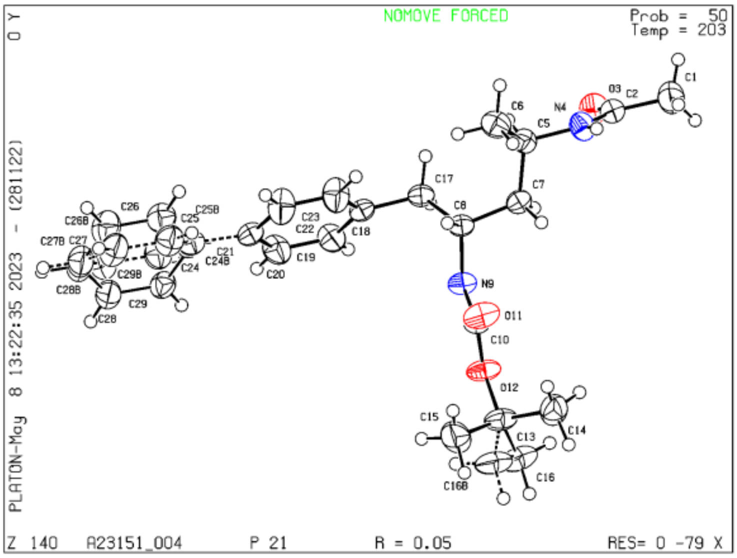

Supplementary Table 12 | Crystal data and structure refinement for **57**

Empirical formula	$C_{24} H_{32} N_2 O_3$	
Formula weight	396.51	
Temperature	203(2) K	
Wavelength	0.71073 Å	
Crystal system	Monoclinic	
Space group	$P2_1$	
Unit cell dimensions	a = 10.8852(7) Å	α = 90°
	b = 5.2166(3) Å	β = 96.6862(18)°
	c = 19.7769(11) Å	γ = 90°
Volume	1115.37(11) $Å^3$	
Z	2	
Density (calculated)	1.181 Mg/m^3	
Absorption coefficient	0.078 mm^{-1}	
F(000)	428	
Crystal size	0.164 x 0.031 x 0.028 mm^3	
Theta range for data collection	2.634 to 27.034°.	
Index ranges	–10<=h<=13, –6<=k<=6, –25<=l<=25	
Reflections collected	21769	
Independent reflections	4866 [R(int) = 0.0940]	
Completeness to theta = 25.242°	99.6 %	
Absorption correction	Semi-empirical from equivalents	
Max. and min. transmission	0.7455 and 0.6678	
Refinement method	Full-matrix least-squares on F^2	
Data / restraints / parameters	4866 / 271 / 327	
Goodness-of-fit on F^2	1.037	
Final R indices [I>2sigma(I)]	R1 = 0.0485, wR2 = 0.0927	
R indices (all data)	R1 = 0.0804, wR2 = 0.1036	
Absolute structure parameter	0.5(8)	
Largest diff. peak and hole	0.132 and –0.150 $e·Å^{-3}$	

Crystallographic data for 59 (CCDC 2321821)

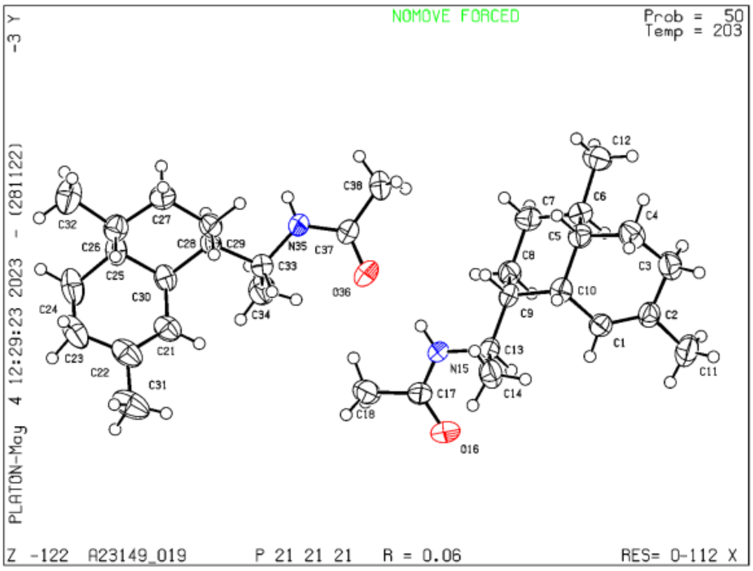

Supplementary Table 13 | Crystal data and structure refinement for **59**

Empirical formula	$C_{16}H_{27}NO$	
Formula weight	249.38	
Temperature	203(2) K	
Wavelength	0.71073 Å	
Crystal system	Orthorhombic	
Space group	$P2_12_12_1$	
Unit cell dimensions	a = 9.4770(5) Å	α = 90°
	b = 9.7795(5) Å	β = 90°
	c = 33.4952(17) Å	γ = 90°
Volume	3104.3(3) $Å^3$	
Z	8	
Density (calculated)	1.067 Mg/m^3	
Absorption coefficient	0.065 mm^{-1}	
F(000)	1104	
Crystal size	0.078 x 0.059 x 0.023 mm^3	
Theta range for data collection	2.469 to 27.088°.	
Index ranges	–11<=h<=12, –12<=k<=12, –31<=l<=42	
Reflections collected	25176	
Independent reflections	6816 [R(int) = 0.1378]	
Completeness to theta = 25.242°	99.8 %	
Absorption correction	Semi-empirical from equivalents	
Max. and min. transmission	0.7455 and 0.6586	
Refinement method	Full-matrix least-squares on F^2	
Data / restraints / parameters	6816 / 0 / 339	
Goodness-of-fit on F^2	0.987	
Final R indices [I>2sigma(I)]	R1 = 0.0616, wR2 = 0.1022	
R indices (all data)	R1 = 0.1284, wR2 = 0.1217	
Absolute structure parameter	0.1(10)	
Largest diff. peak and hole	0.158 and –0.170 e·$Å^{-3}$	

Supplementary References

1. Wang, H. *et al.* Nitrene-Mediated Intermolecular N–N Coupling for Efficient Synthesis of Hydrazides. *Nat. Chem.* **13**, 378–385 (2021).
2. Hwang, Y., Baek, S. B., Kim, D. & Chang, S. Chain Walking as a Strategy for Iridium-Catalyzed Migratory Amidation of Alkenyl Alcohols to Access α-Amino Ketones. *J. Am. Chem. Soc.* **144**, 4277–4285 (2022).
3. Hwang, Y., Wisniewski, S. R. & Engle, K. M. Ligand-Enabled Carboamidation of Unactivated Alkenes Through Enhanced Organonickel Electrophilicity. *J. Am. Chem. Soc.* **145**, 25293–25303 (2023).
4. Keum, H., Jung, H., Jeong, J., Kim, D. & Chang, S. Visible-Light Induced $C(sp^2)$−H Amidation with an Aryl–Alkyl σ-Bond Relocation via Redox-Neutral Radical–Polar Crossover. *Angew. Chem., Int. Ed.* **60**, 25235–25240 (2021).
5. Zhang, L. *et al.* Ritter-Type Amination of $C(sp^3)$-H Bonds Enabled by Electrochemistry with SO_4^{2-}. *Nat. Commun.* **13**, 4138 (2022).
6. Guin, J., Mück-Lichtenfeld, C., Grimme, S. & Studer, A. Radical Transfer Hydroamination with Aminated Cyclohexadienes using Polarity Reversal Catalysis: Scope and Limitations. *J. Am. Chem. Soc.* **129**, 4498–4503 (2007).
7. Chakrabortty, S. *et al.* Cobalt-Catalyzed Enantioselective Hydrogenation of Trisubstituted Carbocyclic Olefins: An Access to Chiral Cyclic Amides. *Angew. Chem., Int. Ed.* **62**, e202301329 (2023).
8. Liang, K. *et al.* Deprotection of Benzyl-Derived Groups via Photochemically Mesolytic Cleavage of C–N and C–O Bonds. *Chem* (2022) **9**, 511-522.
9. Kiely-Collins, H. J., Sechi, I., Brennan, P. E. & McLaughlin, M. G. Mild, Calcium Catalysed Beckmann Rearrangements. *Chem. Commun.* **54**, 654–657 (2018).
10. Roice, M., Christensen, S. F. & Meldal, M. ULTRAMINE: A High-Capacity Polyethylene-Imine-Based Polymer and Its Application as a Scavenger Resin. *Chem. -Eur. J.* **10**, 4407–4415 (2004).
11. Goswami, N. *et al.* Distal meta-Alkenylation of Formal Amines Enabled by Catalytic Use of Hydrogen-Bonding Anionic Ligands. *Chem* **9**, 989–1003 (2023).
12. Morisset, E., Chardon, A., Rouden, J. & Blanchet, J. Phenysilane and Silicon Tetraacetate: Versatile Promotors for Amide Synthesis. *Eur. J. Org. Chem.* **2020**, 388–392 (2020).
13. Beyeh, N. K. *et al.* Cooperative Binding of Divalent Diamides by N-alkyl Ammonium Resorcinarene Chlorides. *Chem. -Eur. J.* **21**, 9556–9562 (2015).
14. Schnapperelle, I. & Bach, T. C4-Selective Oxidative Coupling of Thiophenes with Arylboron Compounds: Influence of Solvent Acidity and Boron Substitution. *ChemCatChem* **5**, 3232–3236 (2013).
15. Zhou, Z. *et al.* Photoinduced Transition-Metal-Free Chan-Evans-Lam-Type Coupling: Dual Photoexcitation Mode with Halide Anion Effect. *J. Am. Chem. Soc.* **144**, 9161–9171 (2022).
16. Gao, Y. *et al.* Dichloroimidazolidinedione-Activated Beckmann Rearrangement of Ketoximes for Accessing Amides and Lactams. *J. Org. Chem.* **83**, 2040–2049 (2018).
17. Minami, H., Otsuka, S., Nogi, K. & Yorimitsu, H. Palladium-Catalyzed Borylation of Aryl Sulfoniums with Diborons. *ACS Catal.* **8**, 579–583 (2018).
18. Zupancic, B., Mohar, B. & Stephan, M. Impact on Hydrogenation Catalytic Cycle of the R Groups' Cyclic Feature in "R-SMS-Phos." *Org. Lett.* **12**, 3022–3025 (2010).
19. Huang, X. *et al.* Asymmetric Synthesis of Primary Amines via the Spiroborate-Catalyzed Borane Reduction of Oxime Ethers. *Org. Lett.* **9**, 1793–1795 (2007).

20. Mazurkiewicz, R. *et al.* A Amidoalkylating Agents from *N*-Acyl-α-Amino Acids: 1-(*N*-Acylamino)alkyltriphenylphosphonium Salts. *J. Org. Chem.* **77**, 1952–1960 (2012).
21. Yi, Z.-J. *et al.* Cu(OTf)$_2$-Catalyzed C3 Aza-Friedel-Crafts Alkylation of Indoles with N,O-Acetals. *Org. Biomol. Chem.* **20**, 2261–2270 (2022).
22. Hong, S. Y. *et al.* Selective Formation of γ-Lactams via C–H Amidation Enabled by Tailored Iridium Catalysts. *Science* **359**, 1016–1021 (2018).
23. Kim, S., Kim, D., Hong, S. Y. & Chang, S. Tuning Orbital Symmetry of Iridium Nitrenoid Enables Catalytic Diastereo- and Enantioselective Alkene Difunctionalizations. *J. Am. Chem. Soc.* **143**, 3993–4004 (2021).
24. Choi, H., Lyu, X., Kim, D., Seo, S. & Chang, S. *endo*-Selective Intramolecular Alkyne Hydroamidation Enabled by NiH Catalysis Incorporating Alkenylnickel Isomerization. *J. Am. Chem. Soc.* **144**, 10064–10074 (2022).
25. Bai, Z. *et al.* Synthesis of N-Acyl Sulfenamides via Copper Catalysis and Their Use as S-Sulfenylating Reagents of Thiols. *Nat. Commun.* **13**, 6445 (2022).
26. Keum, H., Ryoo, H., Kim, D. & Chang, S. Amidative β-scission of Alcohols Enabled by Dual Catalysis of Photoredox Proton-Coupled Electron Transfer and Inner-Sphere Ni-Nitrenoid Transfer. *J. Am. Chem. Soc.* (2023) doi:10.1021/jacs.3c11813.
27. Allen, C. L., Atkinson, B. N. & Williams, J. M. J. Transamidation of Primary Amides with Amines using Hydroxylamine Hydrochloride as an InorganicCcatalyst. *Angew. Chem., Int. Ed.* **51**, 1383–1386 (2012).
28. Li, C.-G. *et al.* Silver-Catalyzed Decarboxylative Alkylfluorination of Alkenes. *Org. Lett.* **21**, 8496–8500 (2019).
29. Torruellas, C., Hsu, F.-L. & Walz, A. J. Synthesis of N-Pyridyl hydroxylamines via Copper-Catalyzed Cross-Coupling. *Synthesis* **51**, 2891–2896 (2019).
30. Ghosh, H. & Patel, B. K. Hypervalent Iodine(III)-Mediated Oxidation of Aldoximes to N-Acetoxy or N-Hydroxy Amides. *Org. Biomol. Chem.* **8**, 384–390 (2010).
31. Parr, R. G. & Weitao, Y. *Density-functional theory of atoms and molecules*. (Oxford University Press, 1995).
32. Frisch, M. J. *Gaussian 09, Revision A.02*. (Gaussian, Inc, 2016).
33. Pracht, P., Bohle, F. & Grimme, S. Automated Exploration of the Low-Energy Chemical Space with Fast Quantum Chemical Methods. *Phys. Chem. Chem. Phys.* **22**, 7169–7192 (2020).
34. Zhao, Y. & Truhlar, D. G. The M06 suite of density functionals for main group thermochemistry, thermochemical kinetics, noncovalent interactions, excited states, and transition elements: two new functionals and systematic testing of four M06-class functionals and 12 other functionals. *Theor. Chem. Acc.* **120**, 215–241 (2008).
35. Fukui, K. Formulation of the Reaction Coordinate. *J. Phys. Chem.* **74**, 4161–4163 (1970).
36. Fukui, K. The Path of Chemical Reactions - the IRC Approach. *Acc. Chem. Res.* **14**, 363–368 (1981).
37. Marenich, A. V., Cramer, C. J. & Truhlar, D. G. Universal Solvation Model Based on Solute Electron Density and on a Continuum Model of the Solvent Defined by the Bulk Dielectric Constant and Atomic Surface Tensions. *J. Phys. Chem. B* **113**, 6378–6396 (2009).
38. Marten, B. *et al.* New Model for Calculation of Solvation Free Energies: Correction of Self-Consistent Reaction Field Continuum Dielectric Theory for Short-Range Hydrogen-Bonding Effects. *J. Phys. Chem.* **100**, 11775–11788 (1996).
39. Friedrichs, M., Zhou, R., Edinger, S. R. & Friesner, R. A. Poisson−Boltzmann Analytical Gradients for Molecular Modeling Calculations. *J. Phys. Chem. B* **103**, 3057–3061 (1999).
40. Edinger, S. R., Cortis, C., Shenkin, P. S. & Friesner, R. A. Solvation Free Energies of Peptides: Comparison of Approximate Continuum Solvation Models with Accurate Solution of the Poisson−Boltzmann Equation. *J. Phys. Chem. B* **101**, 1190–1197 (1997).
