# Supplementary material for: Decarboxylative stereoretentive C–N coupling by harnessing aminating reagent
Source: Nat Commun. 2024 May 6;15:3788. doi: 10.1038/s41467-024-48075-w (PMC11074145; doi:10.1038/s41467-024-48075-w)
Supplement: Supplementary file 3 — Description of Additional Supplementary Files [file 41467_2024_48075_MOESM3_ESM.docx]

**Supplementary data legends**

- Supplementary Information
- Supplementary Data 1 – Cartesian coordinates of Optimized Geometry (excel file)
